# Radical cascade synthesis of azoles *via* tandem hydrogen atom transfer†

**DOI:** 10.1039/c9sc06239d

**Published:** 2020-01-31

**Authors:** Andrew D. Chen, James H. Herbort, Ethan A. Wappes, Kohki M. Nakafuku, Darsheed N. Mustafa, David A. Nagib

**Affiliations:** Department of Chemistry and Biochemistry, The Ohio State University Columbus OH 43210 USA nagib.1@osu.edu

## Abstract

A radical cascade strategy for the modular synthesis of five-membered heteroarenes (*e.g.* oxazoles, imidazoles) from feedstock reagents (*e.g.* alcohols, amines, nitriles) has been developed. This double C–H oxidation is enabled by *in situ* generated imidate and acyloxy radicals, which afford regio- and chemo-selective β C–H bis-functionalization. The broad synthetic utility of this tandem hydrogen atom transfer (HAT) approach to access azoles is included, along with experiments and computations that provide insight into the selectivity and mechanism of both HAT events.

## Abstract 2

A radical cascade enables rapid, modular access to five-membered heteroarenes, including oxazoles and imidazoles, through a regio- and chemo-selective β C–H bis-functionalization.

## Graphical abstract

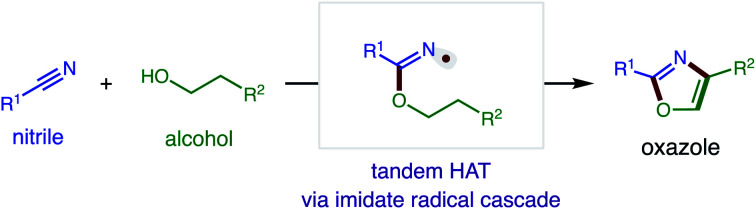

## Introduction

**Fig. 1:**
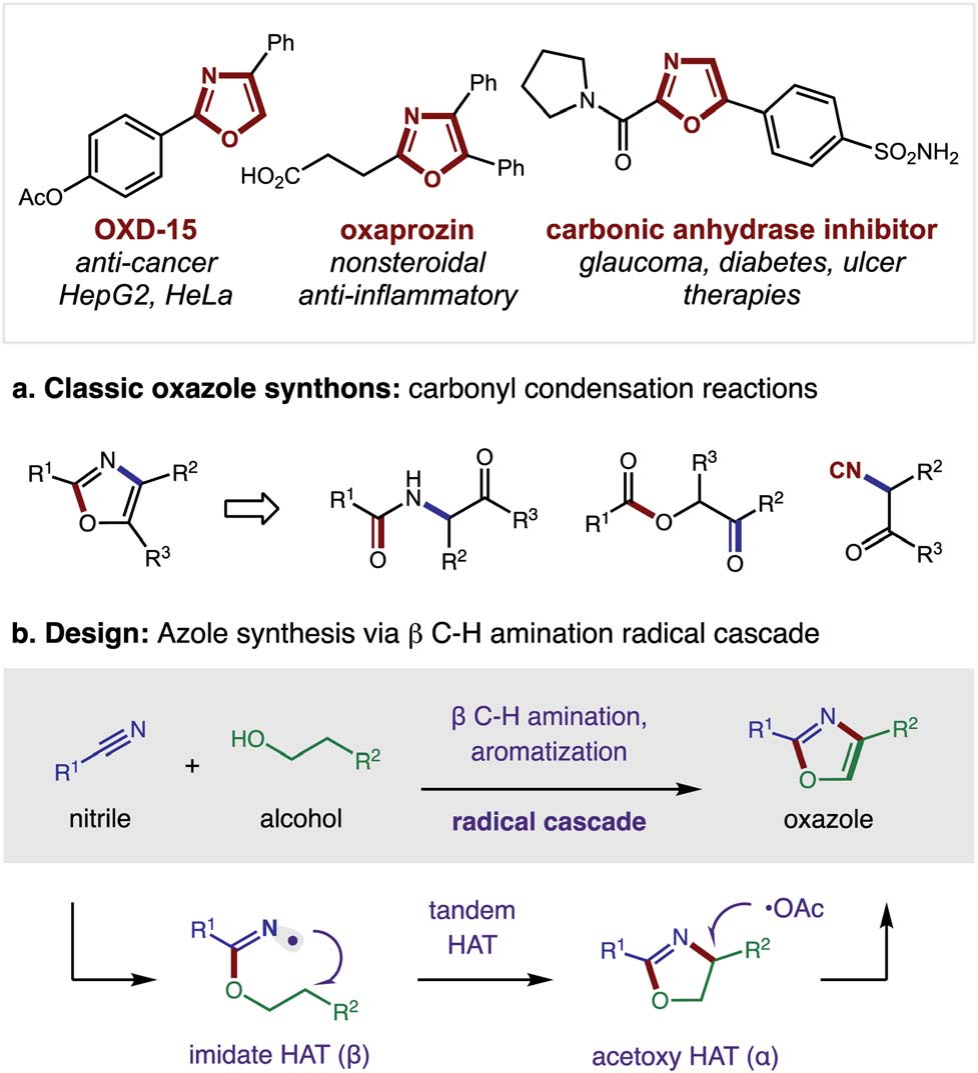
Azole synthesis *via* (a) classic strategies, or (b) tandem hydrogen atom transfer.

Nitrogen-containing heterocycles are prevalent in medicinal chemistry.^1^ Specifically, five-membered heteroaromatics, such as oxazoles, imidazoles, and thiazoles, are among the most common structures found in drugs.^2^ These azoles mimic biological interactions while also providing increased metabolic stability.^3^ Such privileged medicinal motifs are typically synthesized from analogs of amines and carbonyls (Fig. 1a).^4^ Alternatively, we envisioned a complementary synthetic route might entail direct combination of alcohols and nitriles through a double hydrogen atom transfer (HAT) mechanism (Fig. 1b). In this proposal, an imidate may undergo a tandem HAT sequence, which includes β C–H amination and subsequent aromatization of a transient oxazoline, to afford an oxazole. Recently, we disclosed a radical-mediated β C–H amination of imidates^5,6^ to afford oxazolines *via* regioselective HAT.^7,8^ Subsequently, the oxazoline was oxidized to a heteroarene (*i.e.* oxazole) in a post-synthetic operation. Given the pharmacological value of azoles, as well as the broad availability^9^ of the feedstock reagents used in this C–H amination strategy, we questioned if we could streamline the multi-step, imidate radical-mediated strategy into a single, tandem HAT sequence.

**Fig. 2:**
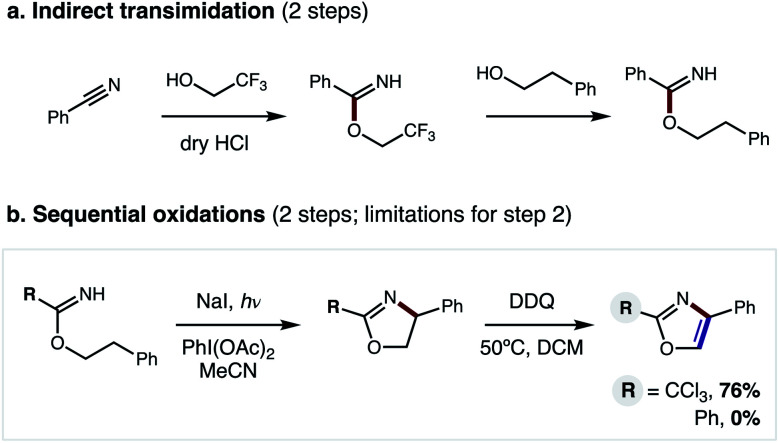
Previous 4-step approach entails: (a) 2-step imidate synthesis, and (b) 2-step oxidation, limited to R = CCl_3_.

In our previous approach, we prepared aryl imidates by a two-step protocol (Fig. 2a), entailing temporary addition of trifluoroethanol to benzonitrile followed by transimidation with the desired alcohol.^5^ The imidate was then converted to an oxazole *via* a second, two-step protocol (Fig. 2b), entailing β C–H amination to form an oxazoline, followed by oxidative aroma-tization with DDQ. A limitation of this strategy is each of these four steps was carried out sequentially, and involved discrete isolation and purification by column chromatography.

Moreover, although β C–H amination is viable with electronically distinct classes of imidates (R = CCl_3_, Ph), only the former variant may be converted to an oxazole (76% yield). Conversely, the more generalizable, aryl oxazolines are oxidatively decomposed by DDQ – affording 0% oxazole (see ESI for details†). Thus, to improve this strategy and significantly expand its scope, we sought to prepare benzimidates in a single step (*versus* the two-step, transimidation route). Importantly, we also postulated that the two C–H oxidations could be performed in a single, tandem oxidative transformation (*cf.*Fig. 1b).

## Results and discussion

**Fig. 3:**
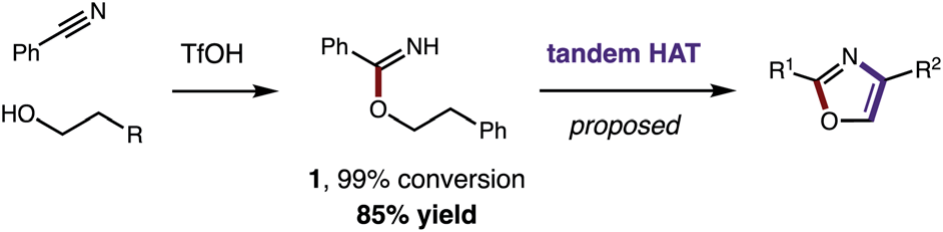
Streamlined access to azoles from alcohols and nitriles (by direct synthesis of benzimidates and tandem HAT).

Toward a more streamlined approach (Fig. 3), we developed a method for preparing benzimidates directly by combining alcohols, nitriles, and triflic acid. This route is significantly more efficient (**1**, 99% conversion, 85% yield) and no longer requires gaseous HCl or sacrificial trifluoroethanol. As an added benefit, these triflate salts are isolable by crystallization (and basic work-up) – precluding the need for column chromatography.

With this one-step protocol for accessing imidates in hand, we then tested our proposal for direct conversion of the imidate to an oxazole *via* tandem C–H oxidation. One of our key findings was that solvent plays a crucial role in promoting double HAT (Table 1). For example, β C–H amination of benzimidate **1** affords oxazoline **2** quantitatively in MeCN. To our surprise, less polar solvents (*e.g.* DCE, PhMe) facilitate a *second* HAT as well, to enable direct formation of oxazole **3** (entries 1–3). Given the limited solubility of alkali iodide salts, other cations were investigated (entries 4–6) with the larger CsI emerging as the most efficient reagent. Control reactions indicate the double oxidation may be initiated with other visible light sources or in the dark – albeit with decreased efficiency (entries 7–9). Interestingly, while our previous catalytic I_2_ conditions^5*b*^ exclusively provide oxazoline in polar solvents (DMF, MeCN), 5% I_2_ affords a 2 : 1 ratio of oxazole : oxazoline in PhMe (entry 10).

**Table 1:** Development of a tandem HAT-mediated synthesis of an oxazole^a^

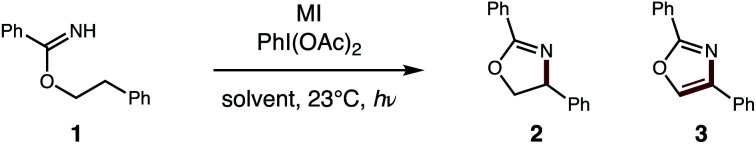					
Entry	MI	Solvent	*hν*	Yield **2**	Yield **3**
**1**	**NaI**	**MeCN**	**23 W CFL**	**99%**	**0%**
2	NaI	DCE	23 W CFL	35%	66%
3	NaI	PhMe	23 W CFL	15%	85%
4	KI	PhMe	23 W CFL	5%	76%
**5**	**CsI**	**PhMe**	**23 W CFL**	**0%**	**98%**
6	^*n*^Bu_4_I	PhMe	23 W CFL	25%	55%
7	CsI	PhMe	Blue LED	0%	82%
8	CsI	PhMe	Dark	75%	12%
9	CsI	PhMe	Dark, 50 °C	50%	35%
10	5% I_2_	PhMe	23 W CFL	58%	24%

a Conditions: imidate (0.2 mmol), MI (3 equiv.), PhI(OAc)_2_ (3 equiv.), solvent (2 mL), 23 W compact fluorescent light (CFL), 23 °C, 24 h. ^1^H NMR yields *vs.* standard.

### Synthetic scope

**Fig. 4:**
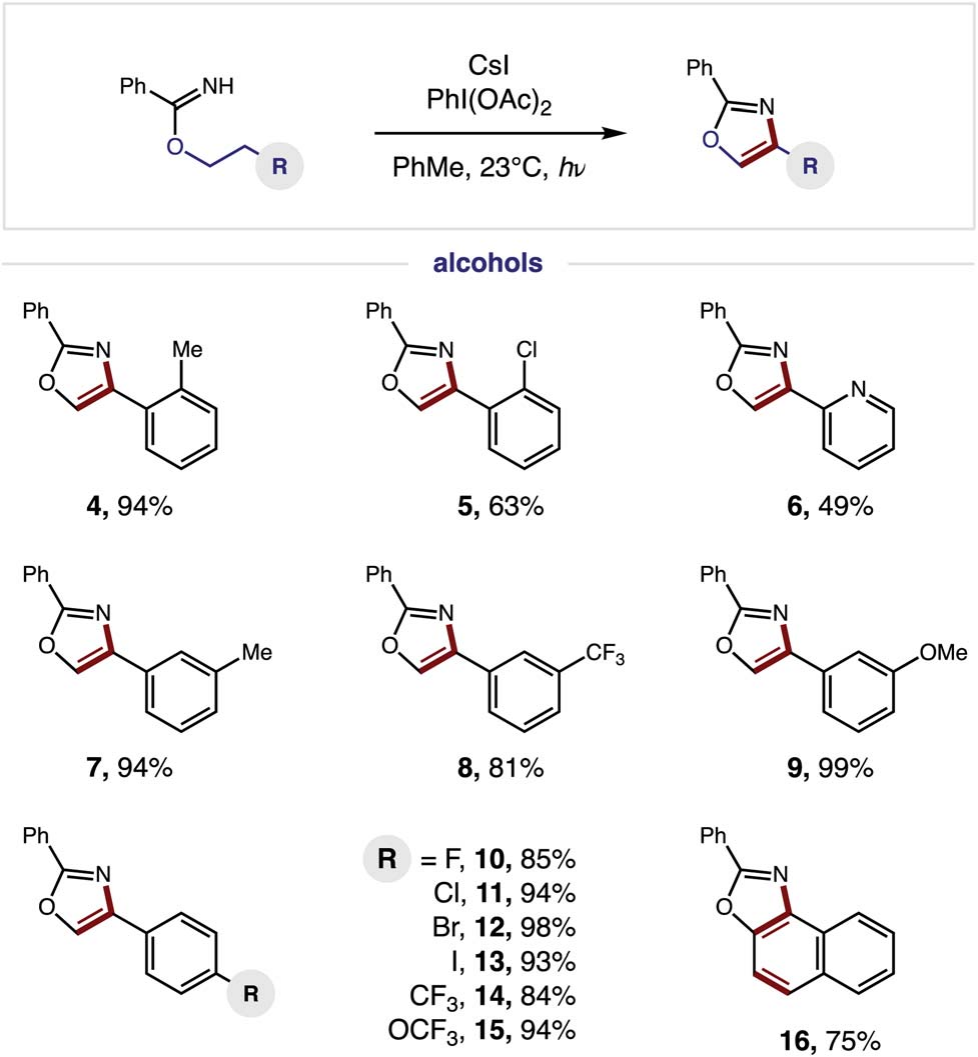
Tandem HAT-mediated oxazole synthesis: alcohol scope.

Having developed an efficient, tandem oxidative method to enable β C–H amination and ensuing HAT to convert imidates to oxazoles, we next investigated the generality of this radical cascade. As shown in Fig. 4, a broad range of alcohols can be employed, including functional groups that are both electronically withdrawing and releasing. These diverse substituents may be at the *ortho* (**4–5**), *meta* (**7–9**), or *para* (**10–15**) positions of various 2-phenylethanol analogs.

Additionally, a pyridyl alcohol affords the privileged bis-heteroarene **6**, commonly employed as a chelating ligand in catalysis or as a drug fragment. Interestingly, the secondary alcohol, tetrahydronaphthalen-2-ol, undergoes a triple C–H oxidation, wherein formation of both a second and third aromatic ring affords naphthyl-fused oxazole **16**.

**Fig. 5:**
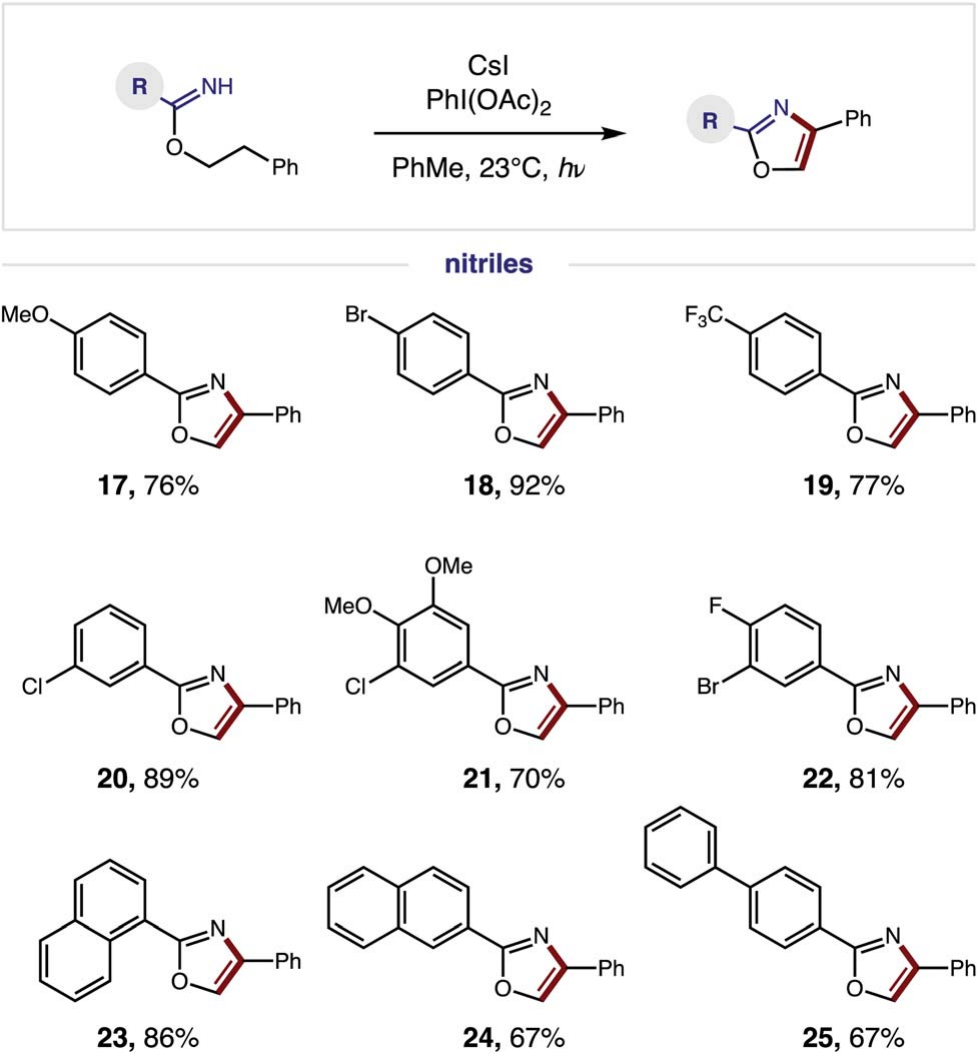
Radical cascade oxazole synthesis: nitrile scope.

We next explored the generality of the nitrile component (Fig. 5). Since a broad range of sterically and electronically diverse nitriles are commercially available, we tested a variety of substitution patterns on the benzonitrile fragment. Again, OMe, CF_3_, and various halide substituents are well tolerated (**17–22**), indicating minimal effect of electronics on efficiency of the tandem oxidative protocol – albeit longer reaction times are required for electron-deficient imidates. Polyaromatic nitriles, including 1- and 2-naphthylenes as well as 4-biphenyl, are also amenable to this oxidative cascade reaction to afford unique (hetero)polyarenes (**23–25**). Notably, this β C–H amination strategy does not afford over-halogenation – in contrast to the benzylic bromination of phenethyl amides, which exclusively yields bromo-oxazoles.^10^

### Synthetic applications

**Fig. 6:**
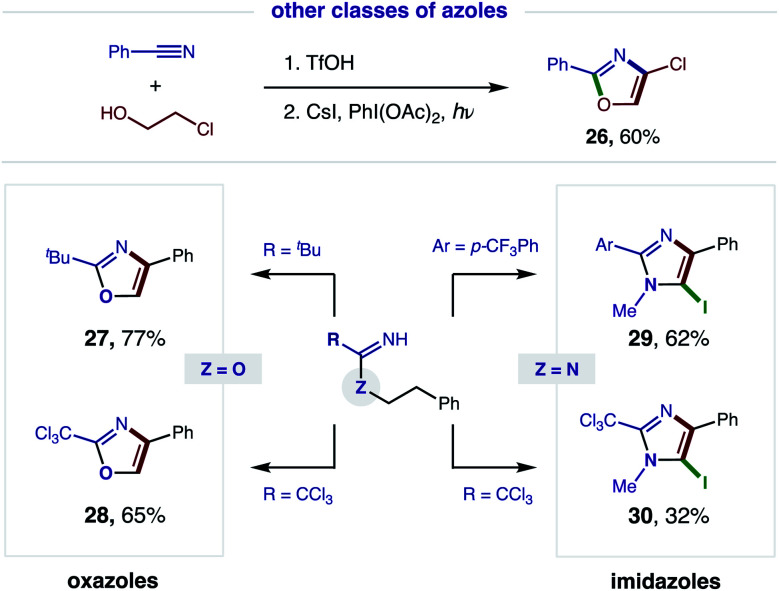
Accessing a family of azoles *via* tandem HAT.

Having demonstrated broad synthetic utility of this radical cascade to access 2,4-bis-aryl-oxazoles, we questioned if this approach could also streamline entry to other classes of azoles (Fig. 6). To this end, we subjected 2-Cl ethanol to our two-step sequence and were pleased to find the intermediate imidate is readily converted to the synthetically versatile 4-Cl oxazole (**26**) *via* double HAT. Next, we examined the aliphatic, pivalonitrile-derived imidate and found it cleanly affords 2-^*t*^Bu-oxazole (**27**). We also developed conditions for tandem β C–H oxidation of a trichloroacetimidate to its oxazole (**28**), precluding our previous need for oxazoline isolation and subsequent oxidation with DDQ.^5*a*^

We next sought to access imidazoles by subjecting amidines (Z = N) to this double HAT, wherein the key β C–H amination step was inspired by Chiba's Cu-catalyzed conversion of amidines to imidazolines.^11^ To this end, we were pleased to find our imidate protocol also converts 2-Ar and 2-CCl_3_ amidines to imidazoles (**29–30**). Interestingly, electrophilic aromatic iodination is also observed yielding 5-iodo-imidazoles *via* a triple C–H oxidation cascade. This third oxidation likely occurs in this case because imidazoles are more easily oxidized than oxazoles (by ∼0.6 V).^12^

**Fig. 7:**
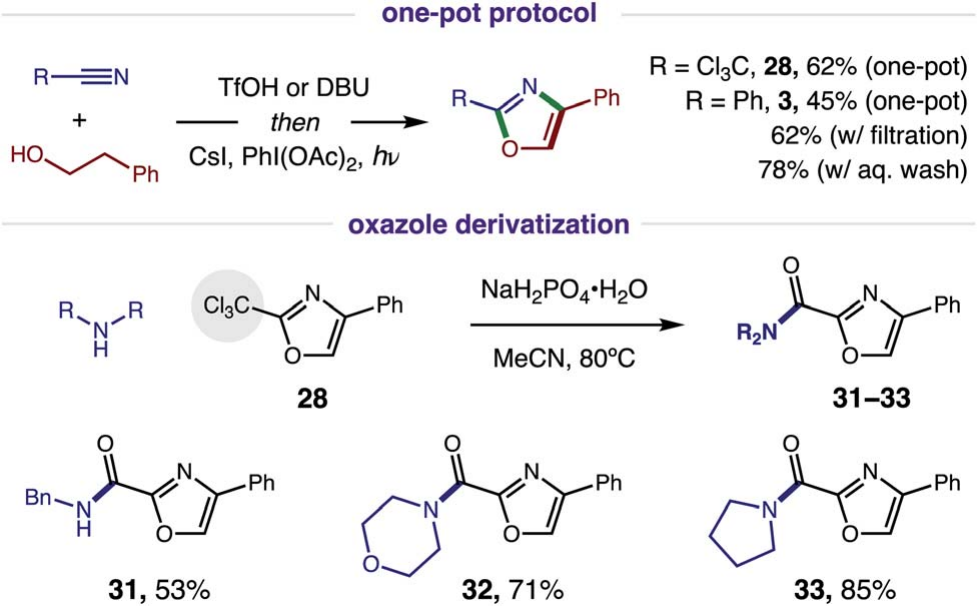
Streamlined synthesis *via* one-pot protocol, and utility of trichloromethyl oxazoles.

Since this new, two-step method affords a more rapid and modular route to azoles, which is ideal for medicinal chemistry applications, we tested the viability of a one-pot protocol for combining these modular, feedstock components (*e.g.* alcohol, nitrile) and converting the resulting imidate to oxazoles (Fig. 7). To our delight, this further streamlined method readily affords both classes of oxazoles (R = CCl_3_**28**, 62%; Ph, **3**, 45%) directly from an alcohol and nitrile. In the less efficient case (**3**), we found the one-pot procedure can be improved by filtration of the imidate hydrotriflate salt intermediate to remove excess TfOH (62%). Alternatively, basic, aqueous wash of the imidate affords greatest efficiency (78%). Given a maximum, theoretical yield of 80% for imidate formation, these tandem oxidations are achieved in 78% and 98% yields, respectively. We anticipate the latter approach will be the preferred method for constructing oxazole libraries for medicinal chemistry applications.

Intrigued by the possibility of derivatizing 2-CCl_3_ oxazoles to other drug-like motifs, we subjected **28** to nucleophilic addition by a variety of amines. Although expecting an addition–elimination mechanism^13^ to displace CCl_3_ by amines, we were surprised to find that amides **31–33** were instead obtained *via* Cl-displacement and ensuing hydrolysis of the remaining *gem*-dichloride. We expect these electron-deficient oxazole amides to exhibit greater metabolic stability as potential drug fragments.^3^ Notably, these motifs have already shown efficacy as carbonic anhydrase inhibitors (*cf.*Fig. 1).

### Mechanistic investigations

**Fig. 8:**
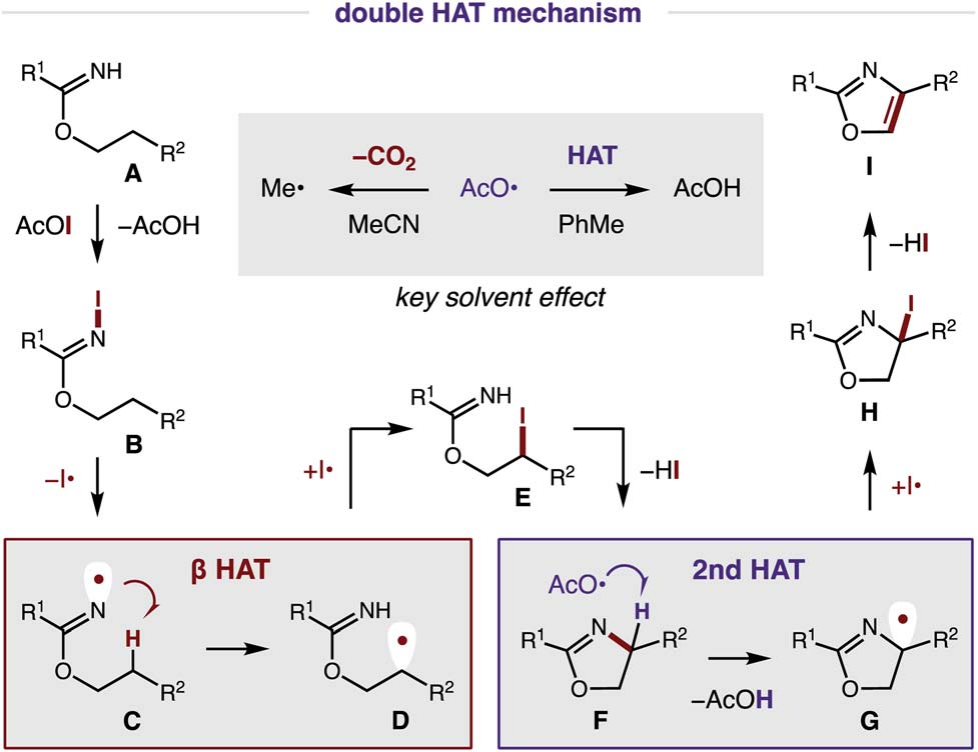
Proposed mechanism and rationale for solvent effect.

Our proposed mechanism for the radical cascade conversion of imidate **A** (from addition of an alcohol and nitrile) to an oxazole is shown in Fig. 8. To start, *in situ* combination of CsI and PhI(OAc)_2_ affords AcOI, which serves as an electrophilic iodine source.^14^ Upon combination with imidate **A**, an *N*-iodo imidate **B** is transiently formed. In the presence of visible light, homolysis of the weak N–I bond (N–I BDFE; 23.0 kcal mol^−1^)^15^ generates N-centered imidate radical **C**, which initiates a regioselective β C–H amination *via* 1,5-HAT to afford C-centered radical **D** (Δ*G* = −6.2 kcal mol^−1^; Δ*G*^‡^ = +14.3 kcal mol^−1^).^16^ The combination of this alkyl radical with an iodine atom (I˙; or AcOI, I_2_, **B**) provides β iodide **E**. Spontaneous cyclization under these conditions affords oxazoline **F**. At this point, a second HAT likely occurs *via* α-imino C–H abstraction (C–H BDFE; 64.8 kcal mol^−1^) by an electrophilic O-centered acyloxy radical (AcO˙ derived from AcOI) to afford AcOH (O–H BDFE; 98.7 kcal mol^−1^) and α-imino radical **G**.^15^ This previously undetected second HAT is both thermodynamically favored and kinetically feasible (Δ*G* = −32.8 kcal mol^−1^; Δ*G*^‡^ = +4.9 kcal mol^−1^), according to DFT calculations (details below).^16^ Combination of α-imino radical **G** with an iodine donor (*e.g.* I˙, AcOI, I_2_, **B**) then provides α-iodide **H**. Finally, aromatization by loss of HI affords oxazole **I** as a final step in this cascade mechanism.^17^

As noted above, solvent polarity is crucial in mediating the second HAT. A likely explanation is that decarboxylation of the H˙ abstracting reagent, AcO˙, to a less efficient HAT mediator, Me˙, is rapid in polar solvents (MeCN), whereas bimolecular HAT may outcompete this β scission in non-polar solvents (PhMe).^18^

**Fig. 9:**
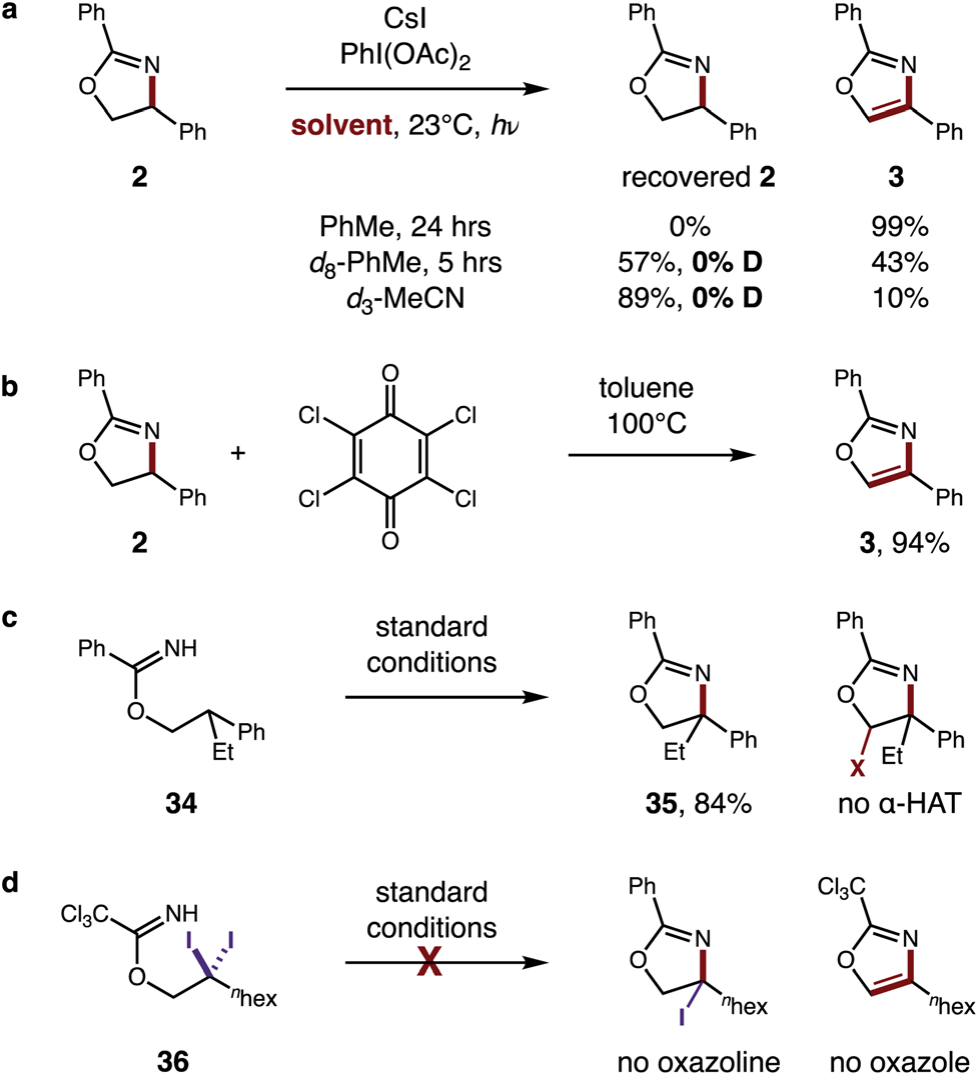
Mechanistic evaluation of second HAT. (a) Intermediate evaluation and deuterium experiments. (b) Benzylic oxidation. (c) HAT regioselectivity. (d) Alternate pathway.

To further evaluate the proposed tandem HAT mechanism, we probed a possibility that solvent does not inhibit the second HAT, but instead prevents conversion of α-imino radical **G** to oxazole by back-HAT from solvent to regenerate the oxazoline (Fig. 9a). To test this hypothesis, oxazoline **2** was prepared and subjected to standard conditions (PhMe, 24 h). The resulting quantitative conversion to oxazole **3** supports the intermediacy of oxazoline **F**. Next, interrupting the reaction at 5 h in *d*_8_-PhMe affords incomplete conversion (43% **3**) with no deuteration of the remaining oxazoline – indicating deleterious back-HAT from solvent is not operative. Similarly, *d*_3_-MeCN affords 10% **3**, but no deuterated oxazoline, suggesting the 89% recovered **2** is more likely a result of inhibited HAT rather than back-HAT. Benzylic oxidation of oxazoline **2** by chloranil, albeit under harsher conditions (100 °C), also affords oxazole **3** (94%), providing further support for this cascade oxidation pathway (Fig. 9b).

To probe the regioselectivity of the proposed second HAT, β di-substituted imidate **34** was prepared and subjected to standard conditions (Fig. 9c). Since the resulting oxazoline **35** contains no α-imino C–H, its efficient formation (84%) and the absence of any further α-oxy oxidation supports the predicted regioselectivity of the second HAT. Finally, an alternate mechanism was also investigated (Fig. 9d), entailing β C–H di-halogenation^6*a*^ followed by subsequent displacement and elimination of the halides to form oxazole. Yet, when subjecting β *gem*-di-iodide **36** to reaction conditions, neither an oxazole nor oxazoline product is observed.

**Fig. 10:**
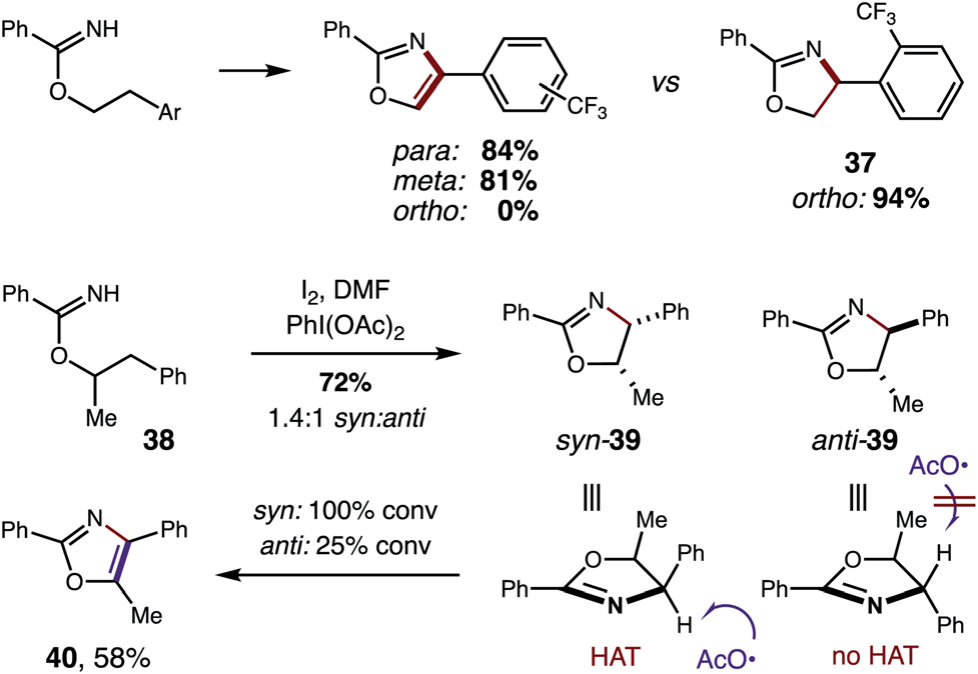
Stereoelectronic effects on second HAT.

In addition to strong solvent effects, we also observed interesting stereoelectronic influences on the second HAT step (Fig. 10). In particular, large steric hindrance by highly withdrawing groups prevent aromatization to the oxazole. For example, a CF_3_ substituent affords oxazoles efficiently when *meta* or *para*, but halts the mechanism at oxazoline **37** when at the *ortho* position. This contrasts with a CH_3_ group, which affords oxazole cleanly – even when substituted at the *ortho* position (**4**). Additionally, acyclic, secondary alcohols exhibit significant steric inhibition for the second HAT. For example, under alternate conditions,^5*b*^ 1-Ph-2-propanol imidate **38** affords a mixture of oxazoline diastereomers (1.4 : 1 *syn* : *anti*). However, upon subjecting oxazolines **39** (mixture of both isomers) to these reaction conditions, the *syn* isomer is readily converted to oxazole **40**, while 75% of the *anti* isomer is recovered. As shown by a model in Fig. 10, the second HAT by AcO˙ is sterically inhibited in the case of the *anti* oxazoline. For a complete list of substrate limitations, see ESI Section X.†

### Computations

**Fig. 11:**
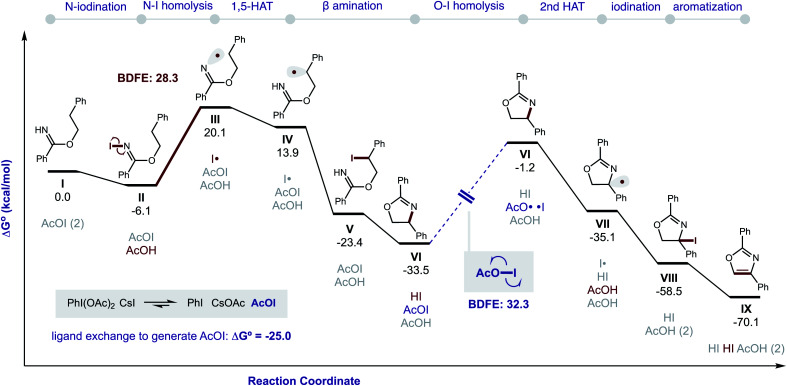
Gibbs free energies of reaction intermediates. ωB97X-D/6-311++G(d,p)/Def2-TZVPP(I and Cs)/PCM(PhMe).

DFT calculations were also performed to provide additional insights into the proposed double HAT cascade mechanism (Fig. 11). First, the free energy of each reaction intermediate was computed – from benzimidate **I** to oxazole **IX**. These results indicate the overall reaction is net exergonic 
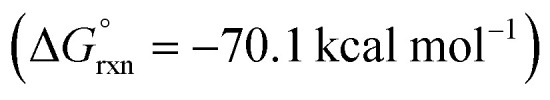
. This is in addition to the energy released upon ligand exchange of CsI and PhI(OAc)_2_ to form AcOI (Δ*G*° = −25.0 kcal mol^−1^). The main contributors to the large thermodynamic driving force for this reaction are aromatization (to form **IX**) and net reduction of AcOI to AcOH (twice; with **II** and **VII**). On the other hand, the most endergonic steps entail radical formation. Specifically, toward generation of the N-centered radical, iodination of imidate **I** to **II** is slightly exergonic (Δ*G* = −6 kcal mol^−1^), but subsequent homolysis of **II** to **III** is much more endergonic (Δ*G* = 26 kcal mol^−1^) – correlating to an N–I BDFE of 28 kcal mol^−1^. Similarly, O–I homolysis (to generate AcO˙ from AcOI) is highly endergonic (BDFE = 32 kcal mol^−1^), which corresponds to the large Δ*G* for **VI** (and intermediates) in the reaction coordinate diagram prior to the second HAT. Notably, applying implicit solvent models (that largely rely on field-based effects such as dielectric constant) to both the decarboxylation and second HAT pathways does not explain the experimentally observed solvent effects.

**Fig. 12:**
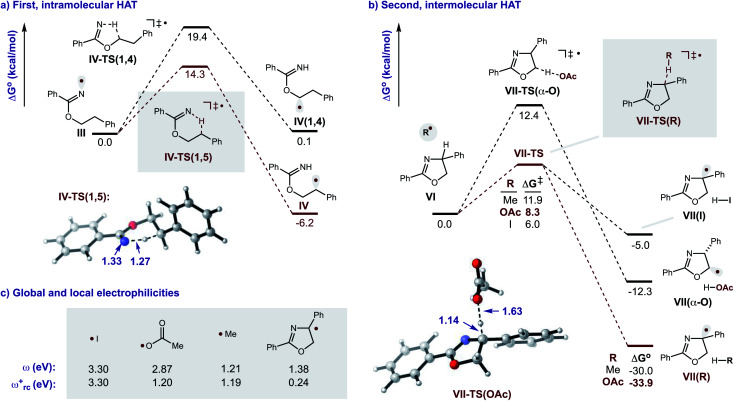
Energetics of HAT steps. (a) 1,5 *vs.* 1,4 HAT. (b) Intermolecular HAT pathways. Selected TS bond lengths in angstroms, Å. (c) Global and local electrophilicities of key radicals. ωB97X-D/6-311++G(d,p)/Def2-TZVPP(I)/PCM(PhMe).

Additional calculations were performed to provide further insight into the key HAT steps of the reaction mechanism (Fig. 12a). First, 1,5-HAT of imidate radical **III** to β-carbon radical **IV***via***IV-TS(1,5)** was found to be kinetically feasible (Δ*G*^‡^ = 14.3 kcal mol^−1^) and thermodynamically downhill 
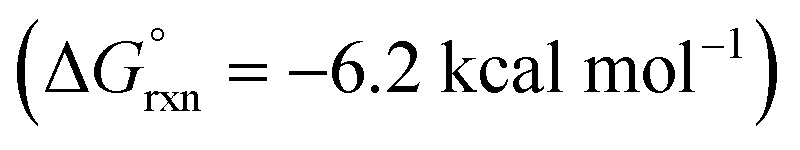
. Next, probing the energetics behind the 1,5-HAT regioselectivity, an alternate 1,4-HAT pathway, **IV-TS(1,4)**, was found to be much less favoured – both kinetically (ΔΔ*G*^‡^ = 5 kcal mol^−1^) and thermodynamically 
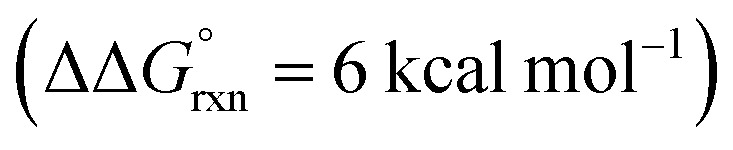
. Additionally, the C–H bond length in transition state **IV-TS(1,5)** is closer to that of starting material **INT-III** (C–H: 1.27 *vs.* 1.09 Å, respectively) than the N–H bond of **IV-TS** is to product **INT-IV** (N–H: 1.32 *vs.* 1.02 Å, respectively), suggesting an early TS, which is consistent with 1,5-HAT being exergonic.

Importantly, we also investigated the second, intermolecular HAT in order to determine the nature and selectivity of this second C–H functionalization (Fig. 12b). Given the presence of several species in the reaction medium that could promote intermolecular HAT of oxazoline **VI** (*e.g.* I˙, AcO˙, Me˙), we individually computed each possible pathway. Among these, HAT by I˙ was found to have the lowest kinetic barrier (**VII-TS(I)**, Δ*G*^‡^ = 6.0 kcal mol^−1^), but results in the least exergonic pathway (**VII(I)**, 
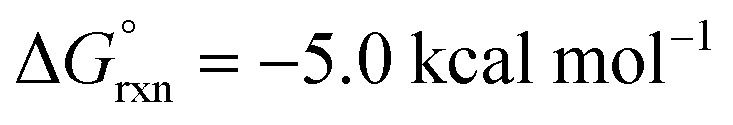
). Thus, whereas HAT by I˙ is feasible, the reverse reaction would be competitive. On the other hand, AcO˙ mediated HAT was determined to be next most accessible, kinetically (**VII-TS(OAc)**, Δ*G*^‡^ = 8.3 kcal mol^−1^), as well as the most exergonic (**VII(OAc)**, Δ*G*° = −33.9 kcal mol^−1^). Thus, we expect HAT by AcO˙ to be substantially less reversible. We also probed HAT by Me˙ since these species are formed by β-fragmentation of acetoxy radicals. Whereas Me˙ to Me–H HAT is feasible, it is kinetically slower than AcO˙ (**VII-TS(Me)**, Δ*G*^‡^ = 11.9 *vs.* 8.3 kcal mol^−1^) and less exergonic (**VII(Me)**, Δ*G*° = −30.0 *vs.* −33.9 kcal mol^−1^). Lastly, we considered the possibility of intermolecular HAT by an imidate N-centered radical – albeit this scenario would necessitate intermolecular HAT to outcompete intramolecular HAT. Nevertheless, such a pathway was found to have the highest kinetic barrier (Δ*G*^‡^ = 15.9 kcal mol^−1^), while being only moderately exergonic (Δ*G*° = −22.0 kcal mol^−1^). In support of these calculations, the experimental addition of non-1,5-HAT-capable imidates lacking β hydrogens, does not improve reaction efficiency (see ESI, Section IX†). Collectively, these data suggest HAT by acetoxy radical is the most likely pathway, since AcO˙ is the second most reactive and first most exergonic.

Turning our attention to selectivity of C–H abstraction from the oxazoline, we considered a competitive HAT at the α-oxygen C–H bond. Approach of AcO˙ was made from the sterically less hindered face, *trans* to the phenyl group. When compared to the α-imino C–H, this HAT is disfavoured, both kinetically (**VII-TS(α-O)**, Δ*G*^‡^ = 12.4 *vs.* 8.3 kcal mol^−1^) and thermodynamically (**VII(α-O)**, 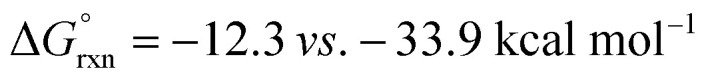).

Since kinetic reactivity of radicals are heavily influenced by their polarity,^19^ we sought to account for these effects by calculating the global and local electrophilicities (*ω* and *ω*_rc_^+^, respectively) of the candidate H-abstractors (Fig. 12c). As radical **VII** (resulting from second HAT) is nucleophilic (*ω*_rc_^+^ = 0.24 eV), its generation is best matched with an electrophilic H-atom abstractor. Indeed, the relative order of kinetic reactivity was found to correlate with increasing local electrophilicity, from most to least reactive: iodine (3.30), acetoxy (1.20), methyl (1.19), and imidate (0.96). These data corroborate the free energy results that suggest AcO˙ is the likely mediator of the second HAT.

## Conclusions

In summary, a radical cascade strategy has enabled a streamlined, modular synthesis of azoles. This rapid approach for constructing molecular complexity from feedstock reagents is enabled by a tandem HAT mechanism. We expect the methods and mechanistic insights for this regio- and chemo-selective β C–H bis-functionalization presented herein will serve as a foundation for developing additional multi-component couplings based on radical-mediated C–H difunctionalization.

## Conflicts of interest

There are no conflicts to declare.

## Supplementary Material

SC-011-C9SC06239D-s001

SC-011-C9SC06239D-s002
