# Supplementary figures and images for: Radical cascade synthesis of azoles via tandem hydrogen atom transfer
Source: Chem Sci. 2020 Jan 31;11(9):2479–86. doi: 10.1039/c9sc06239d (PMC8157396; doi:10.1039/c9sc06239d)

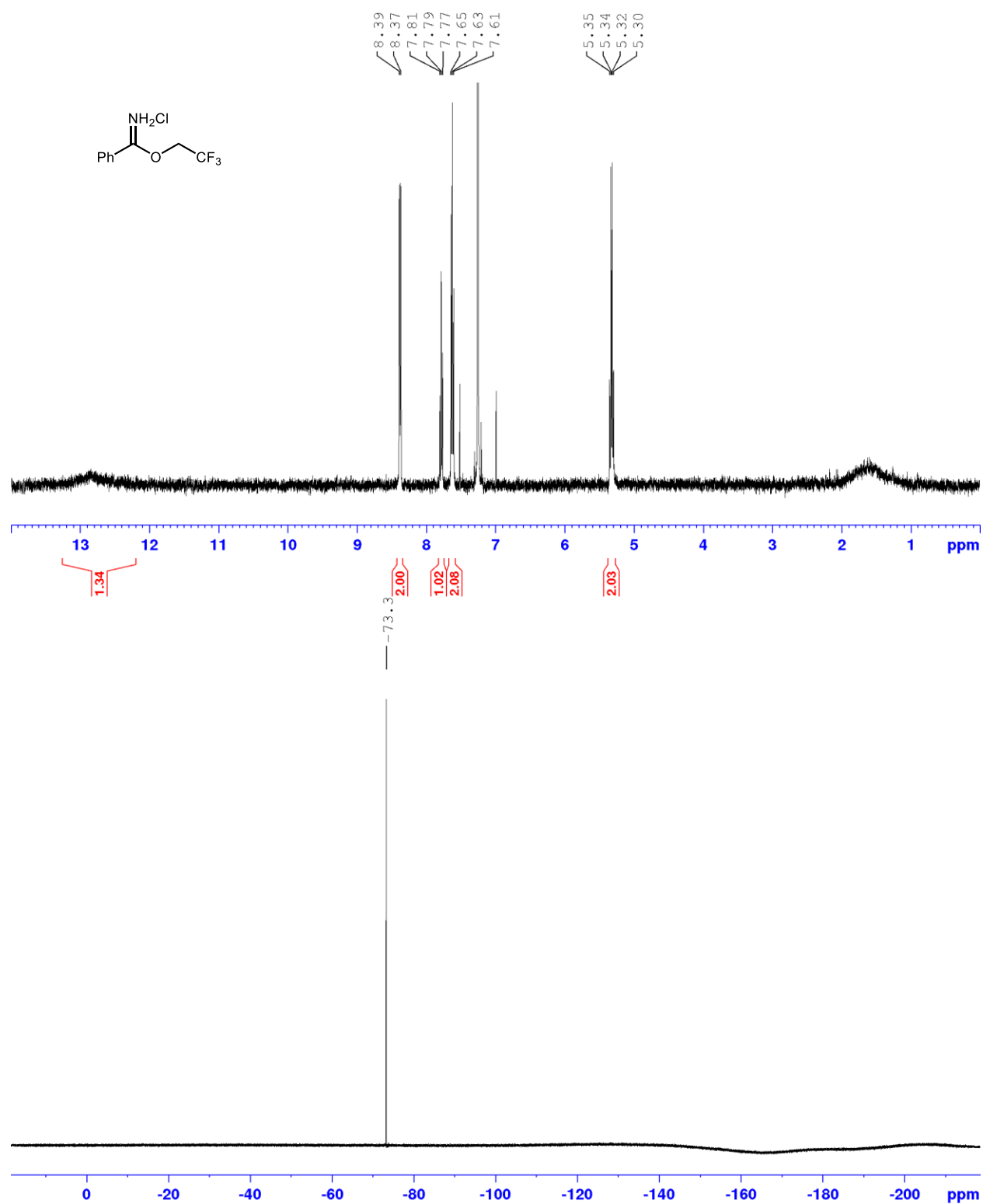

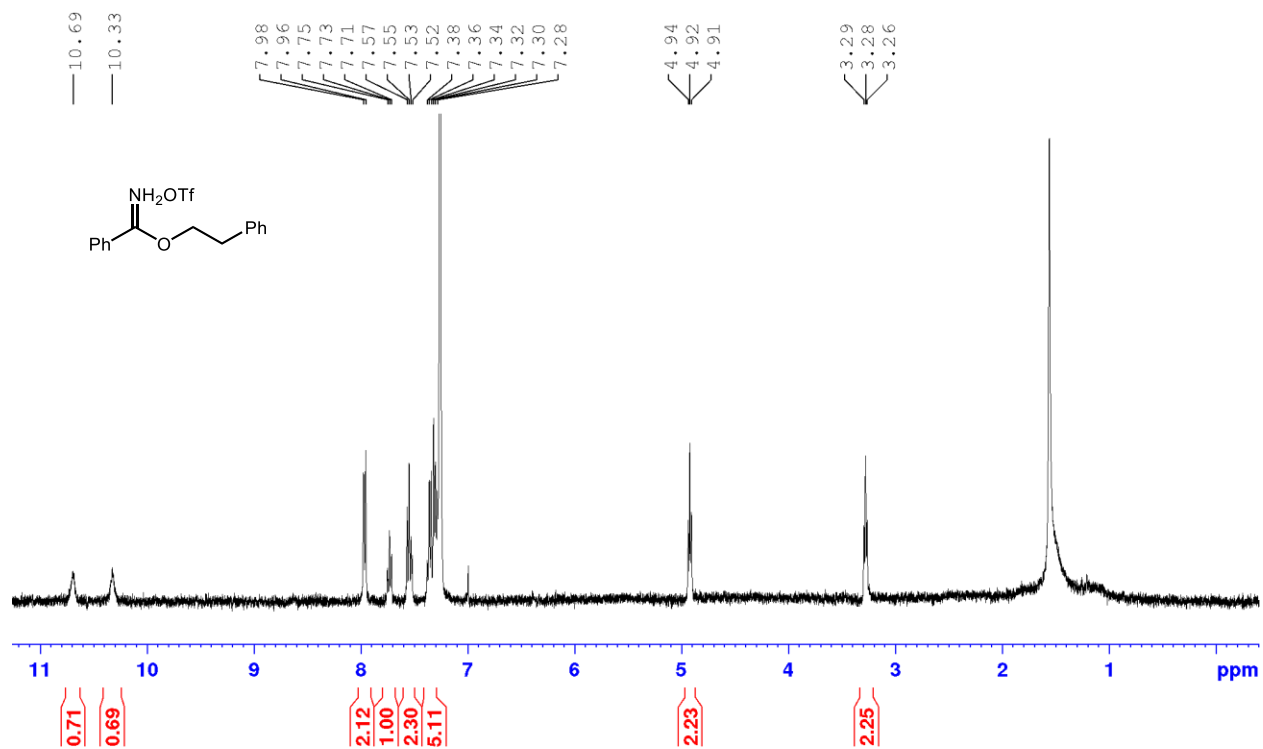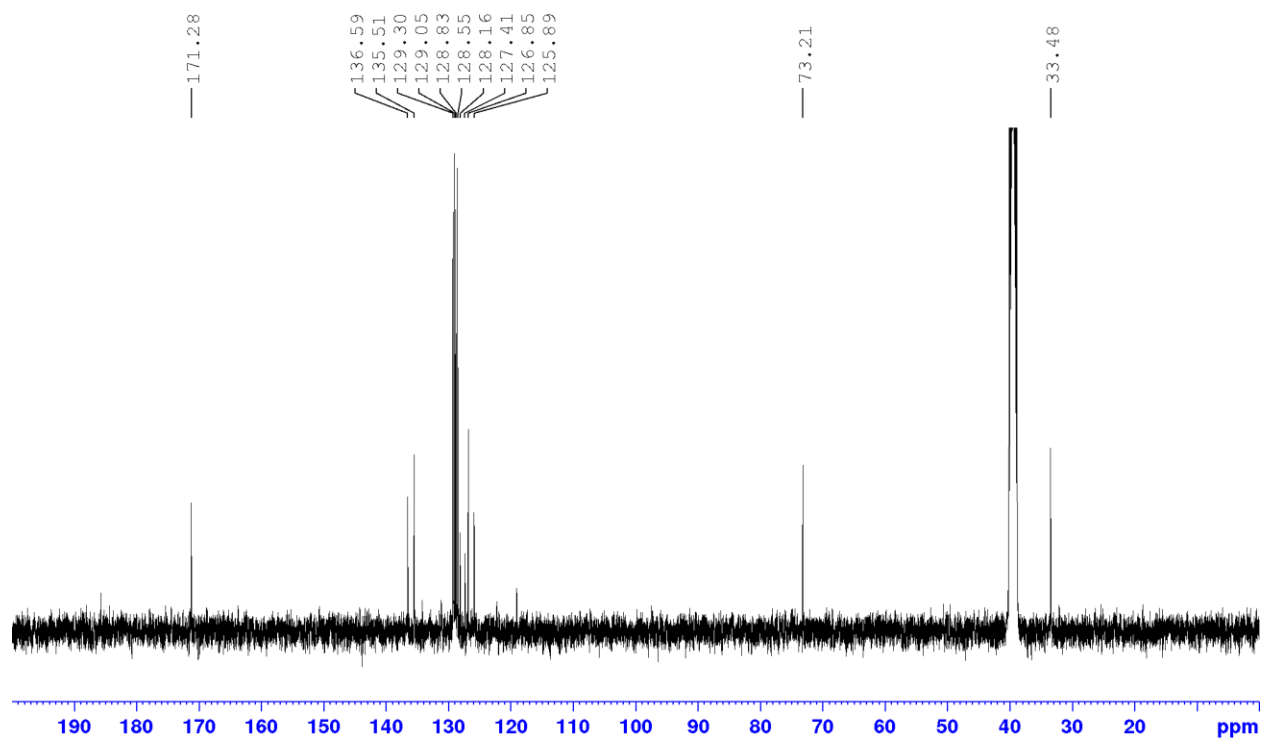

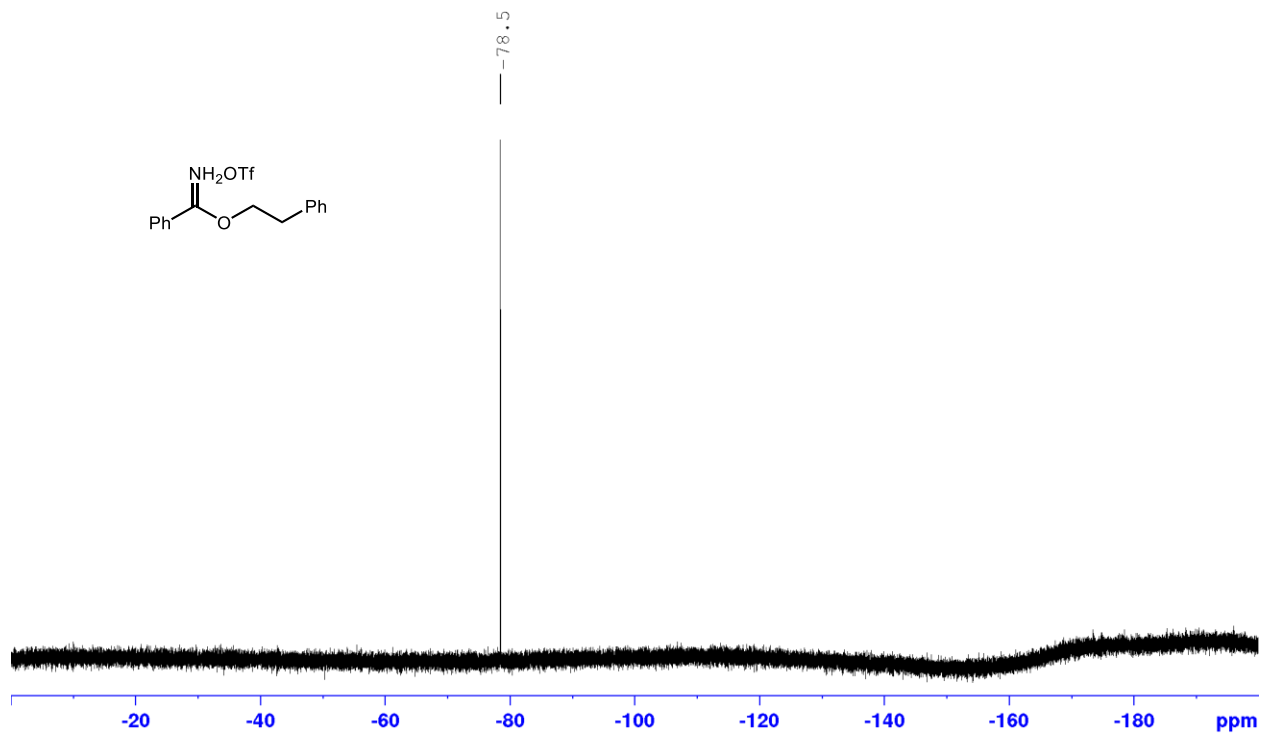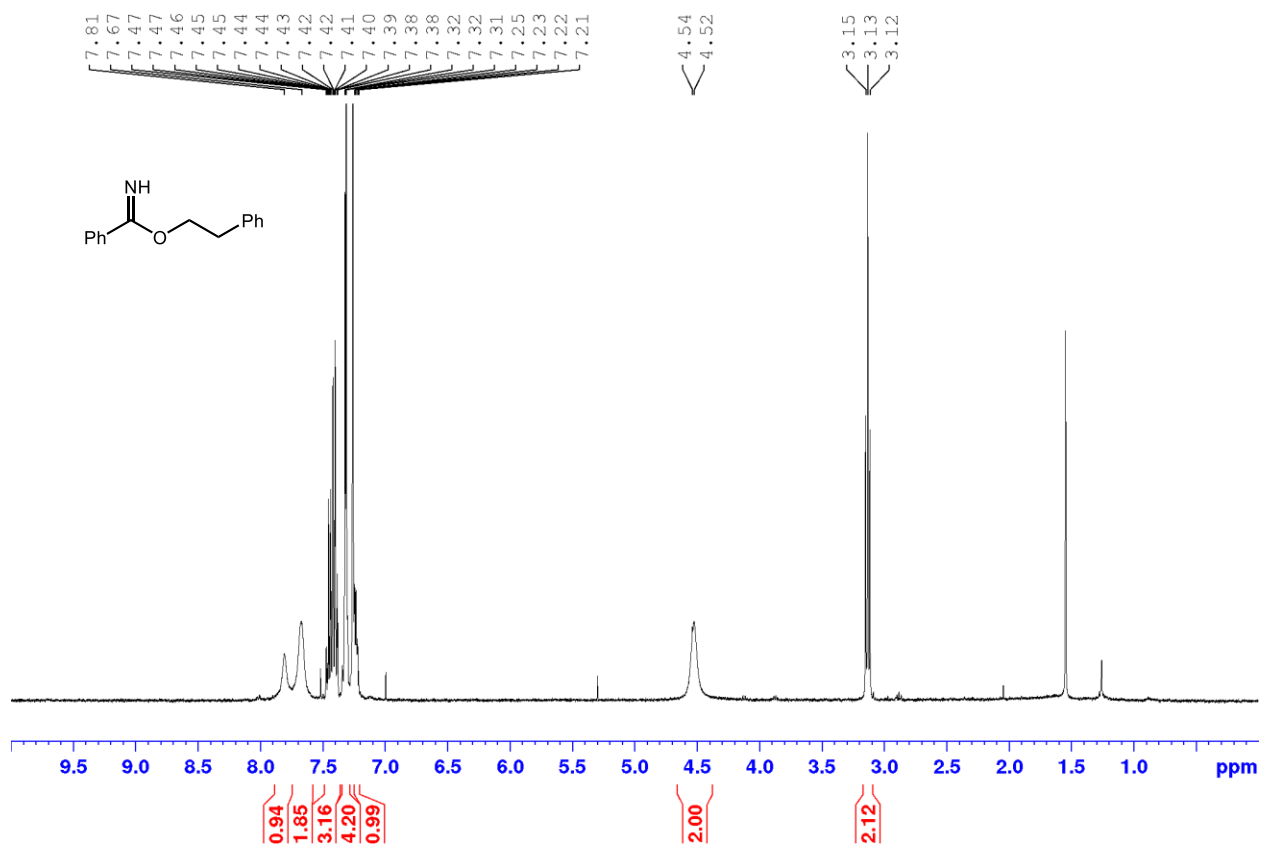

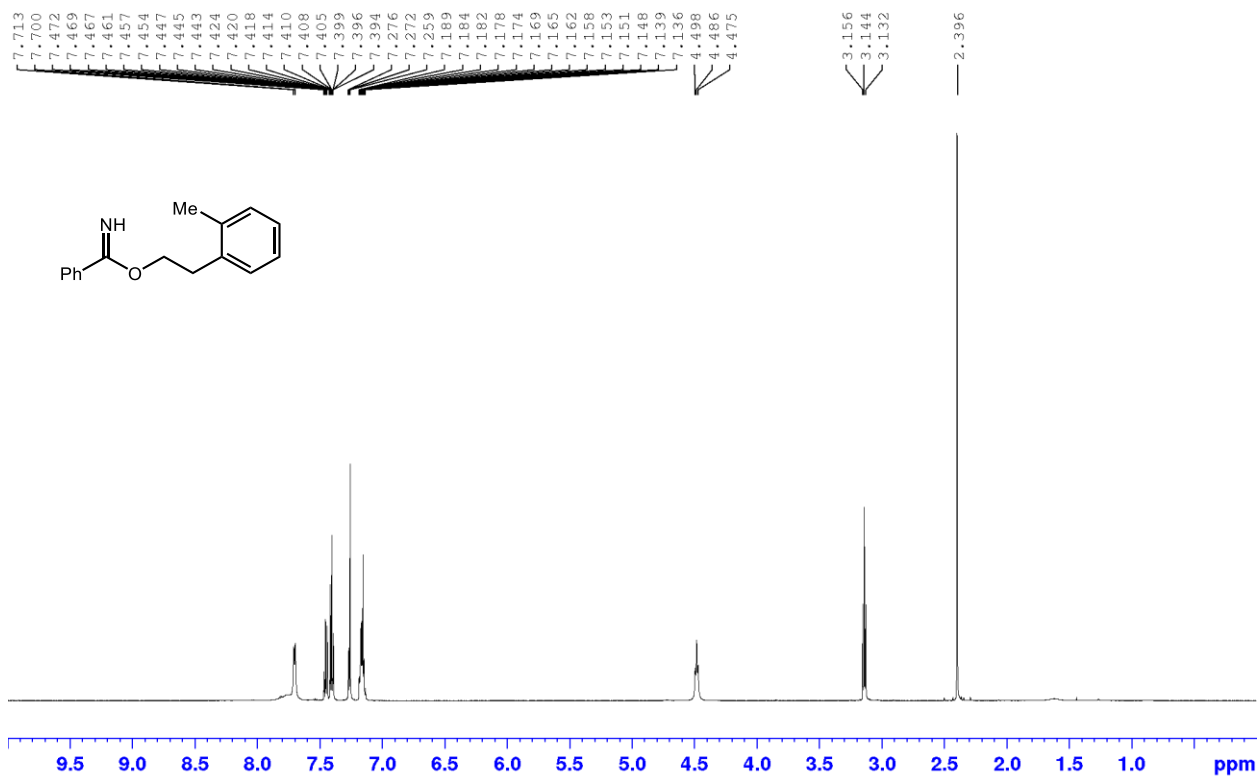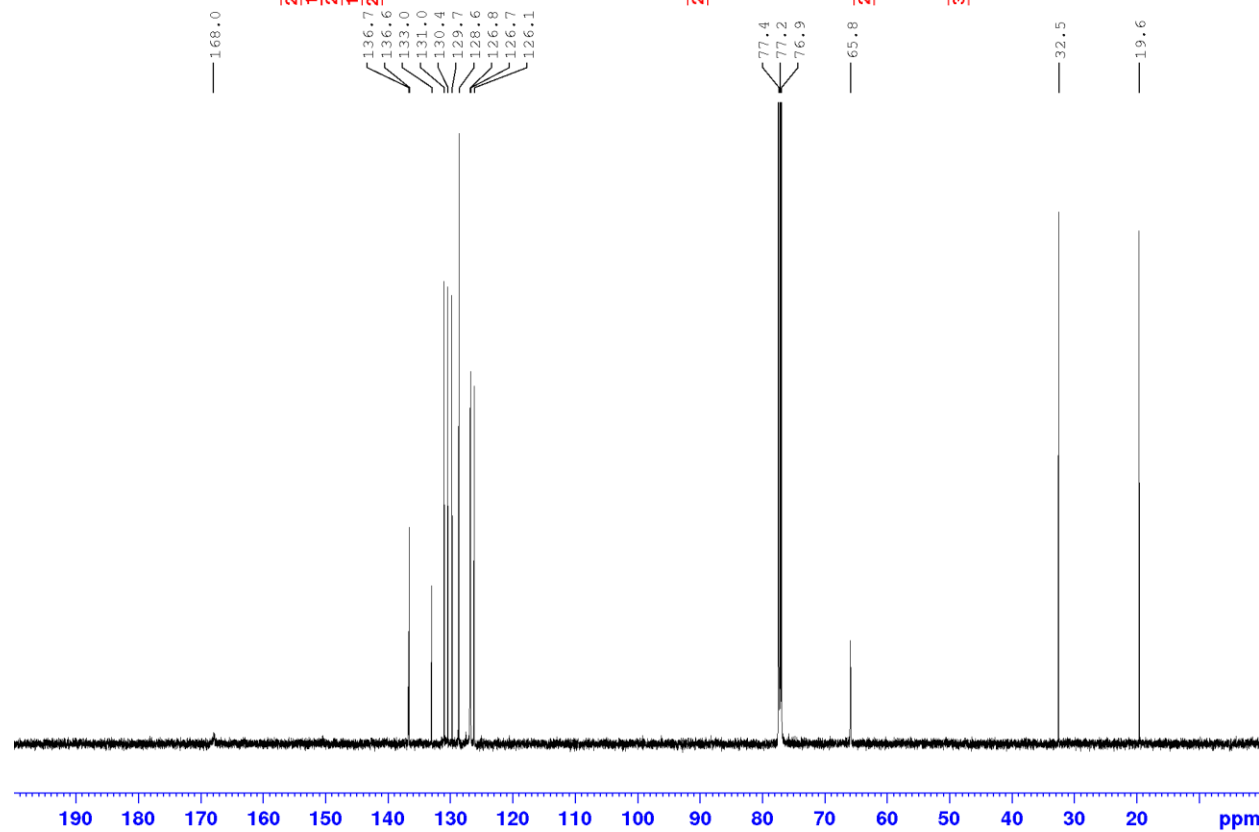

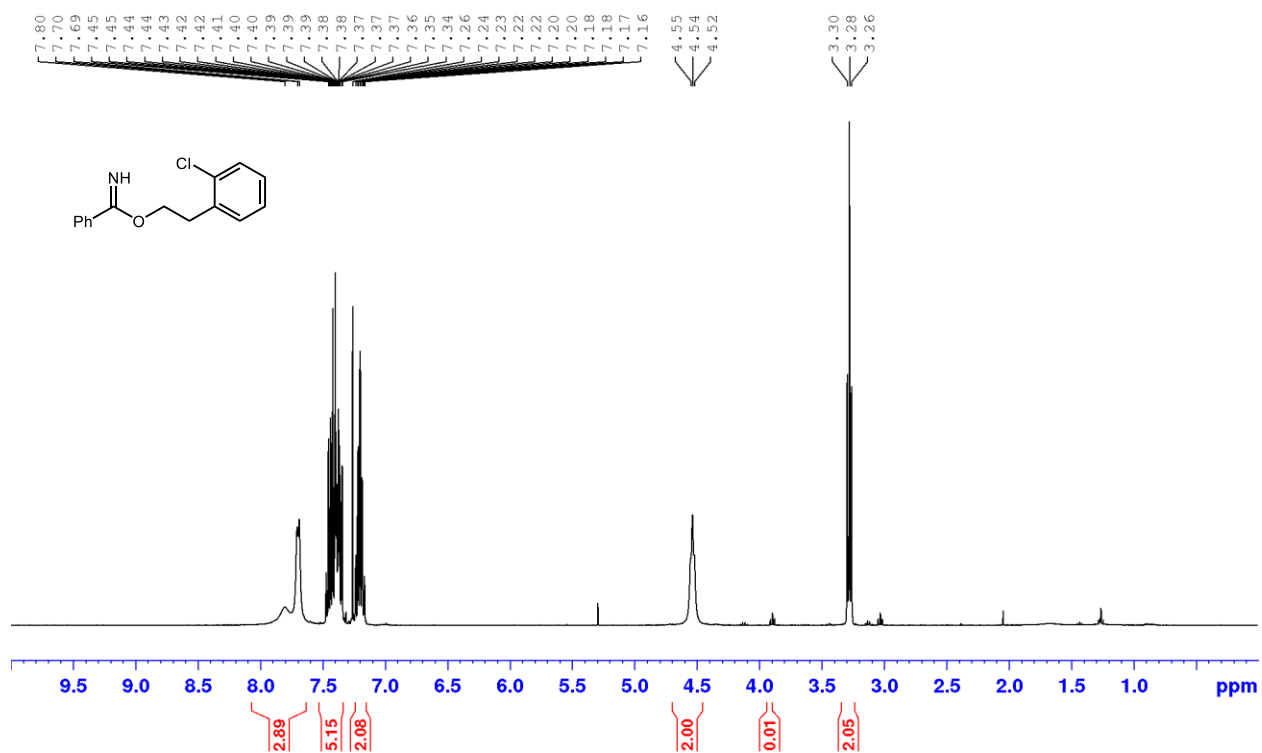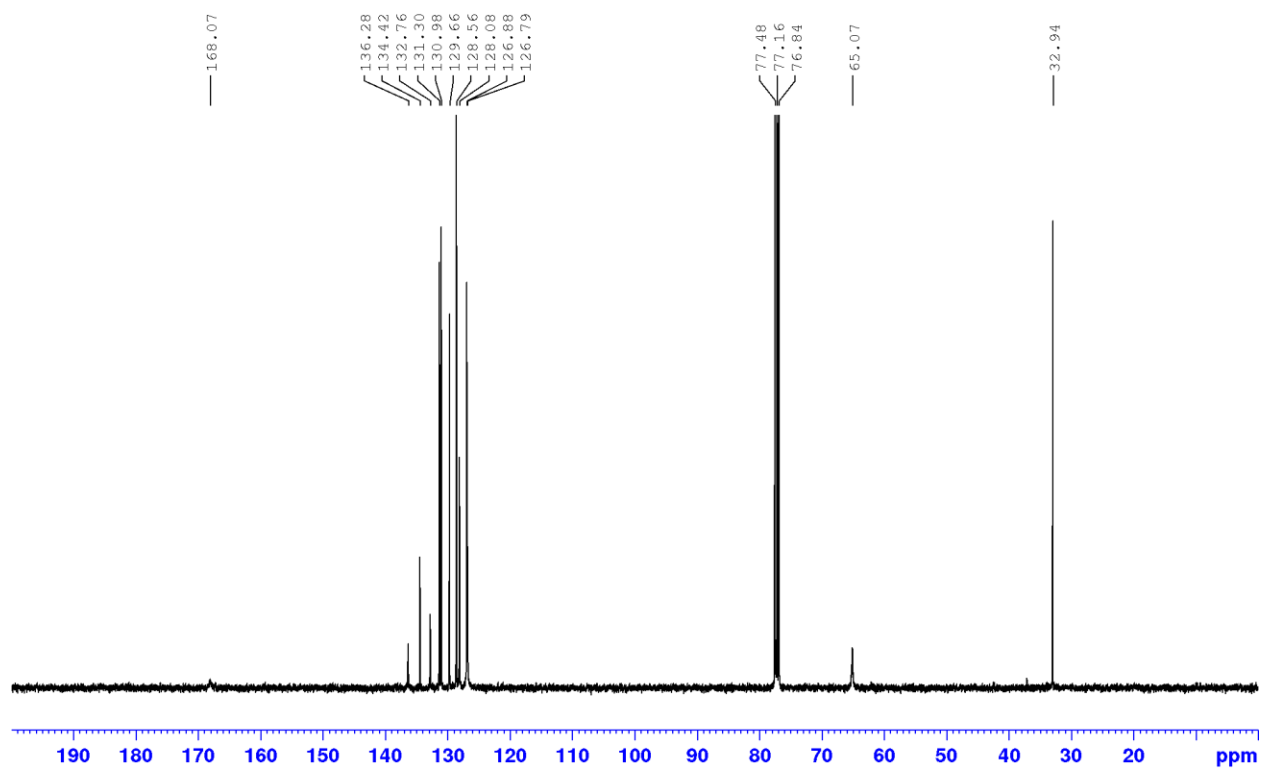

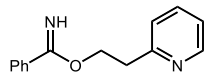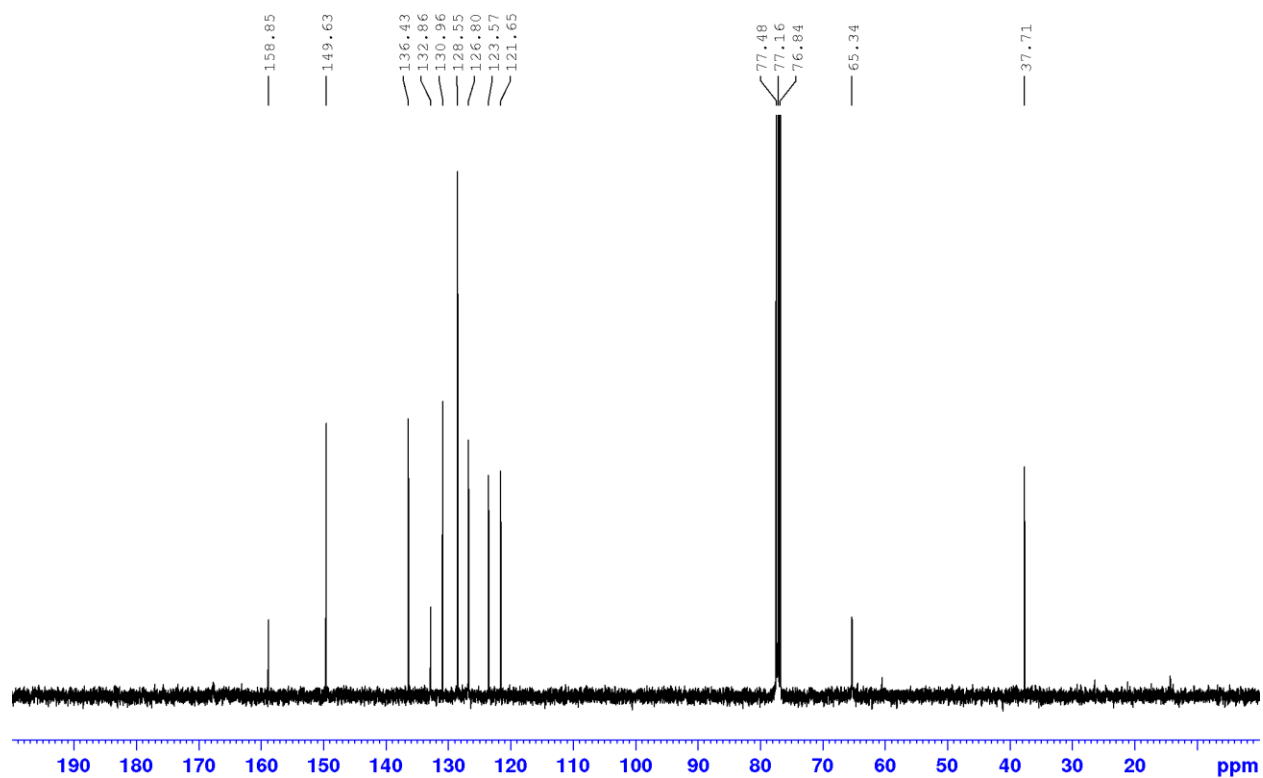

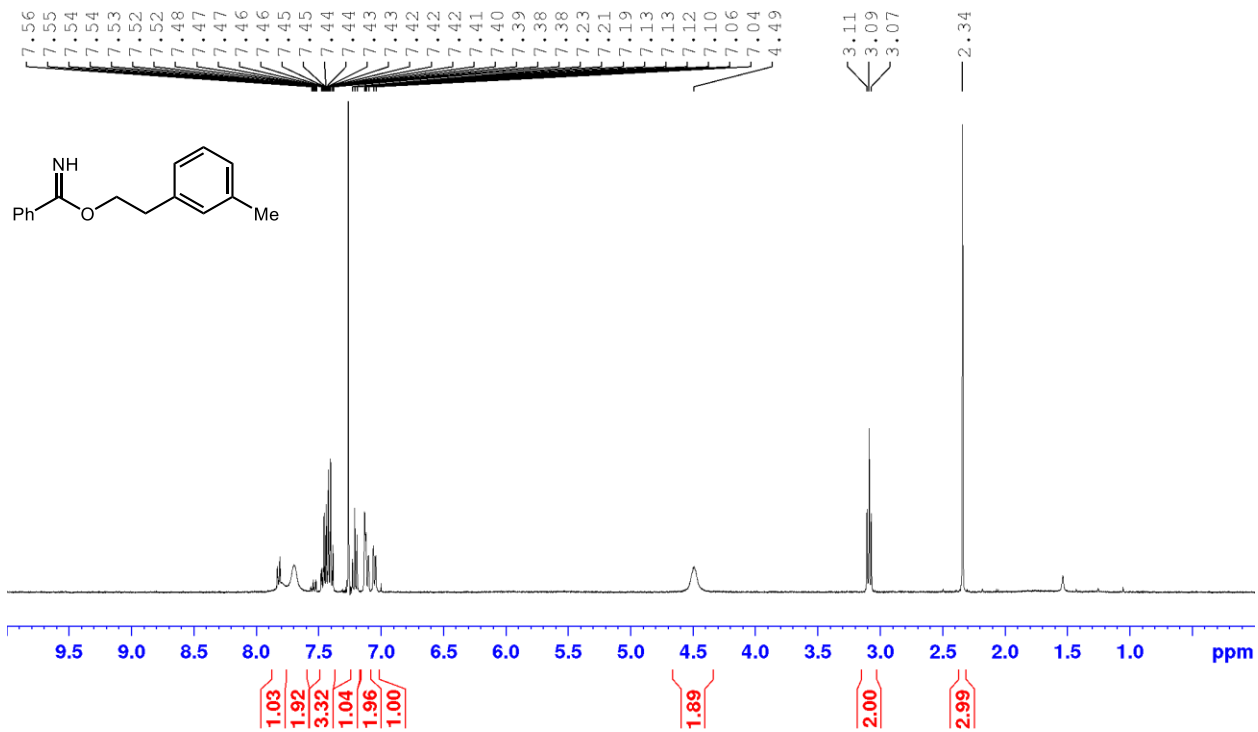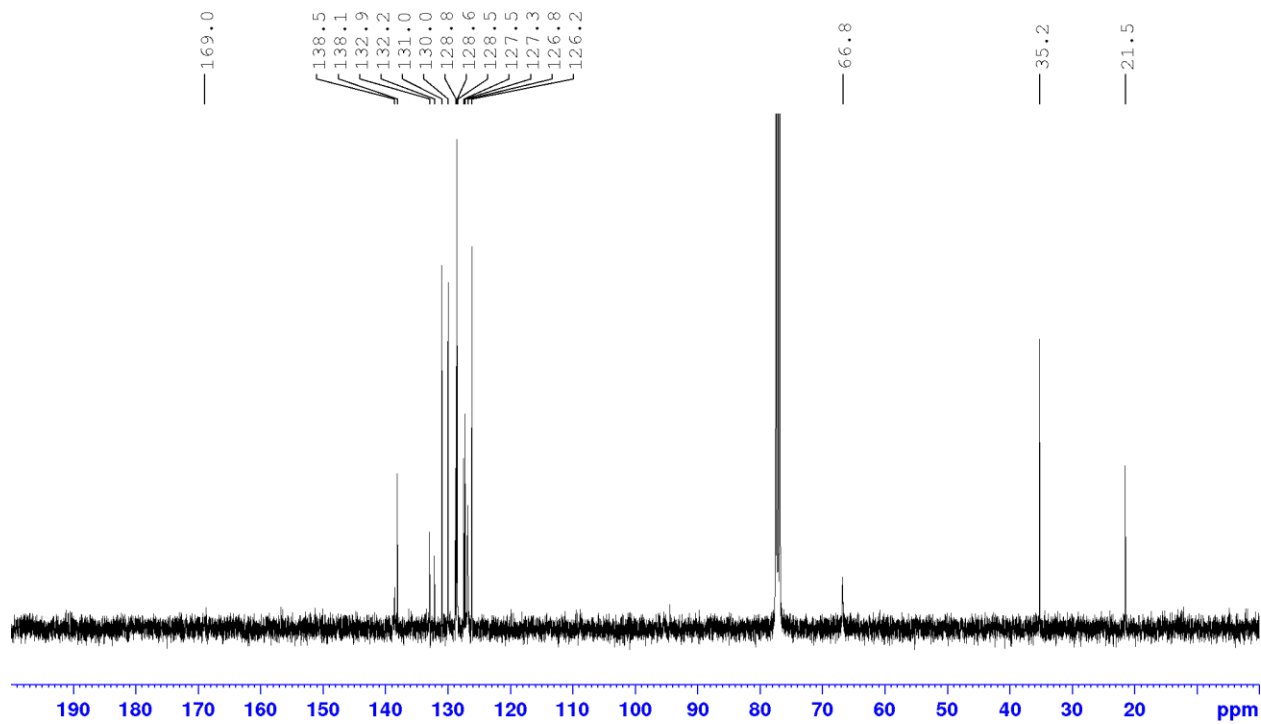

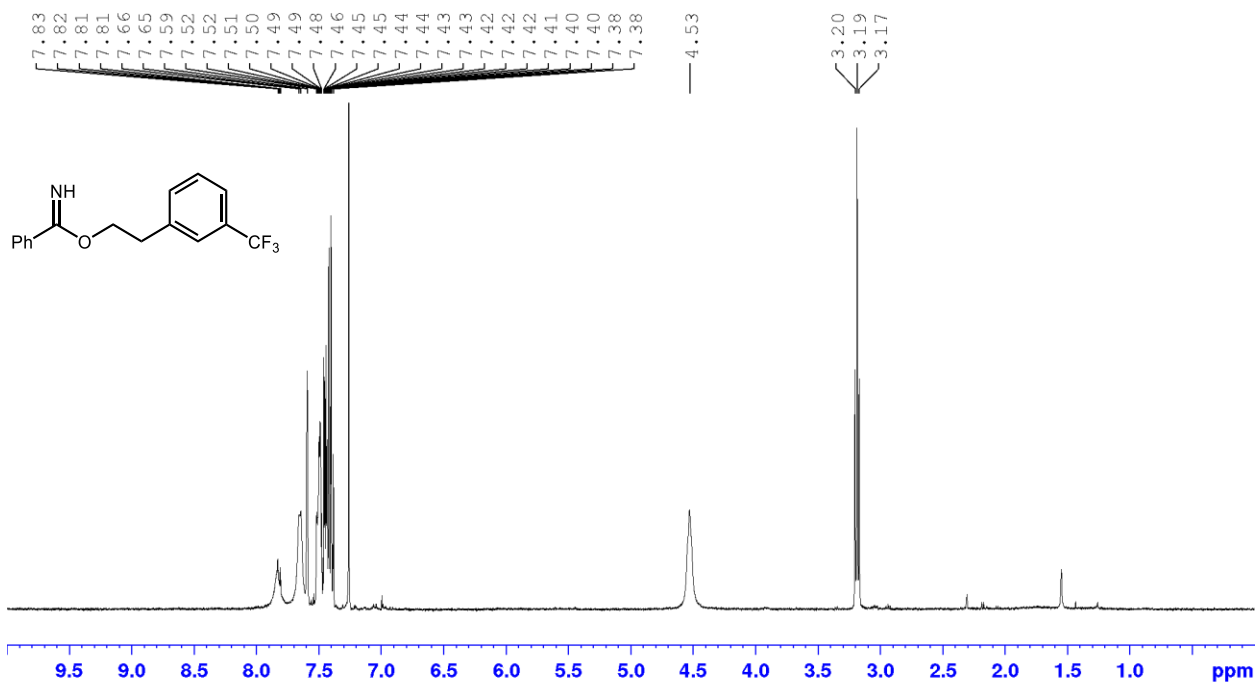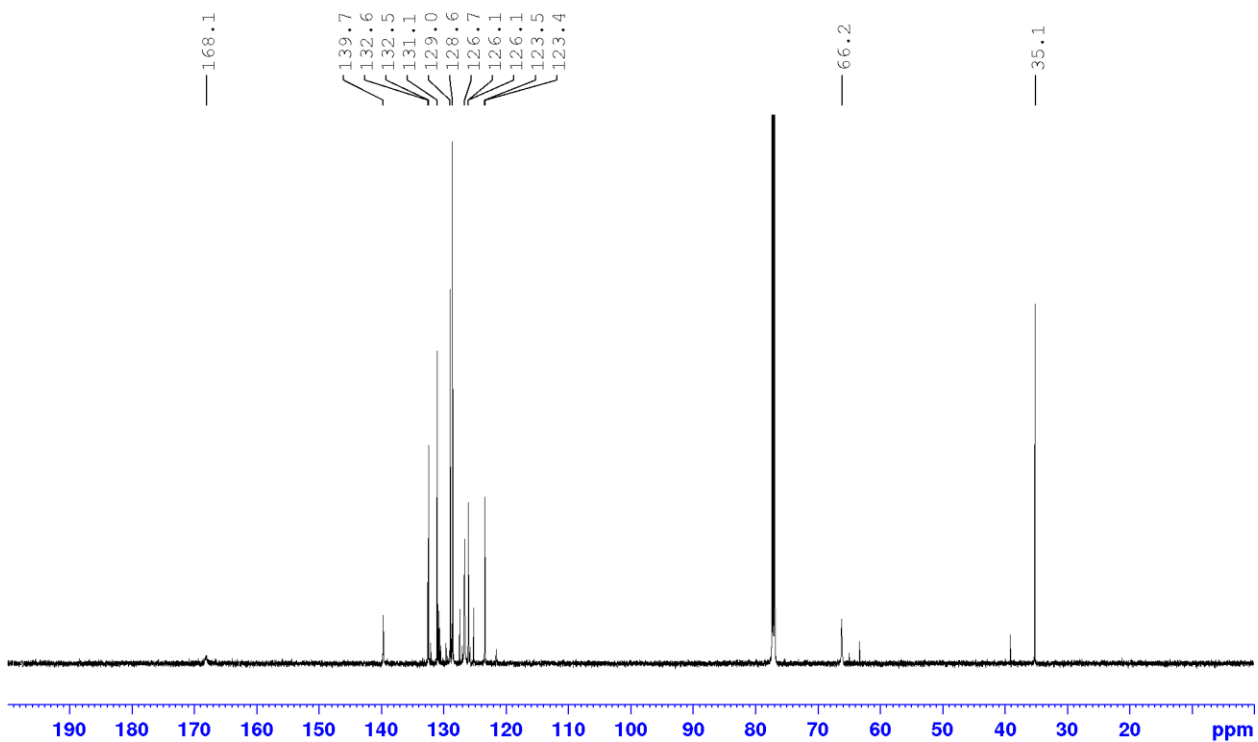

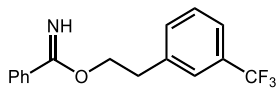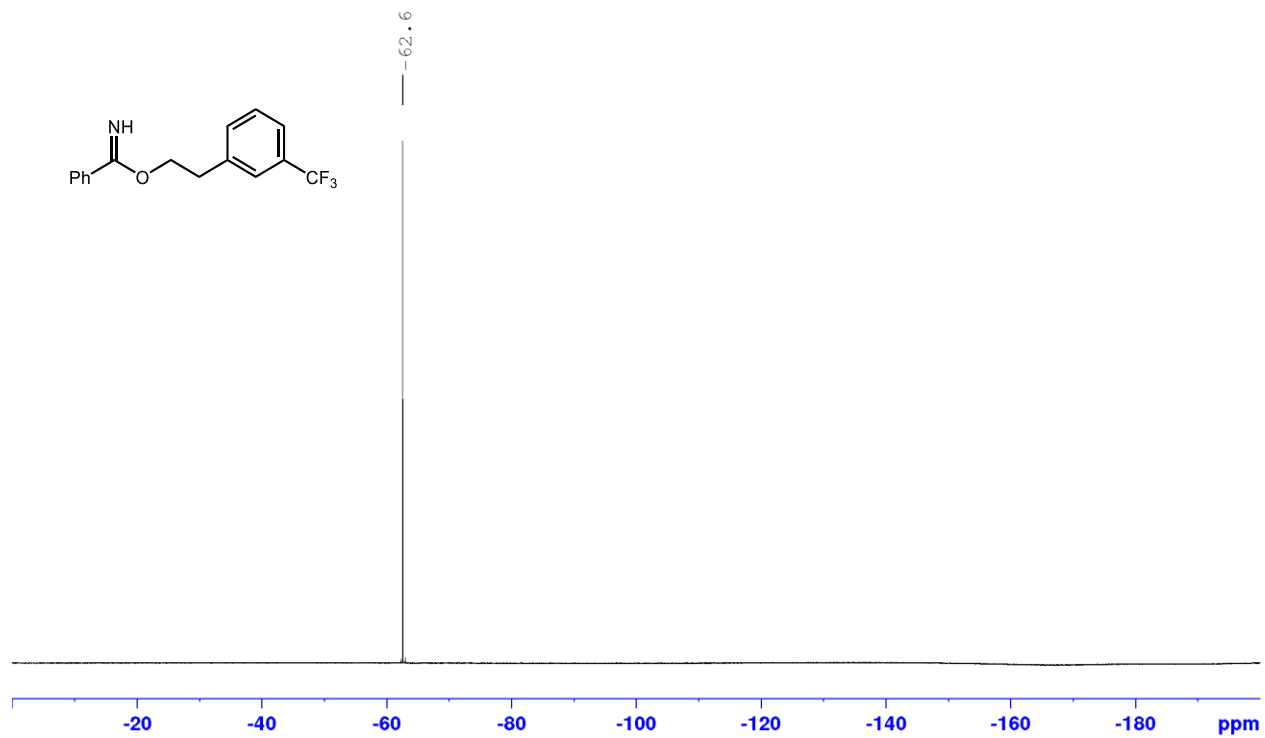

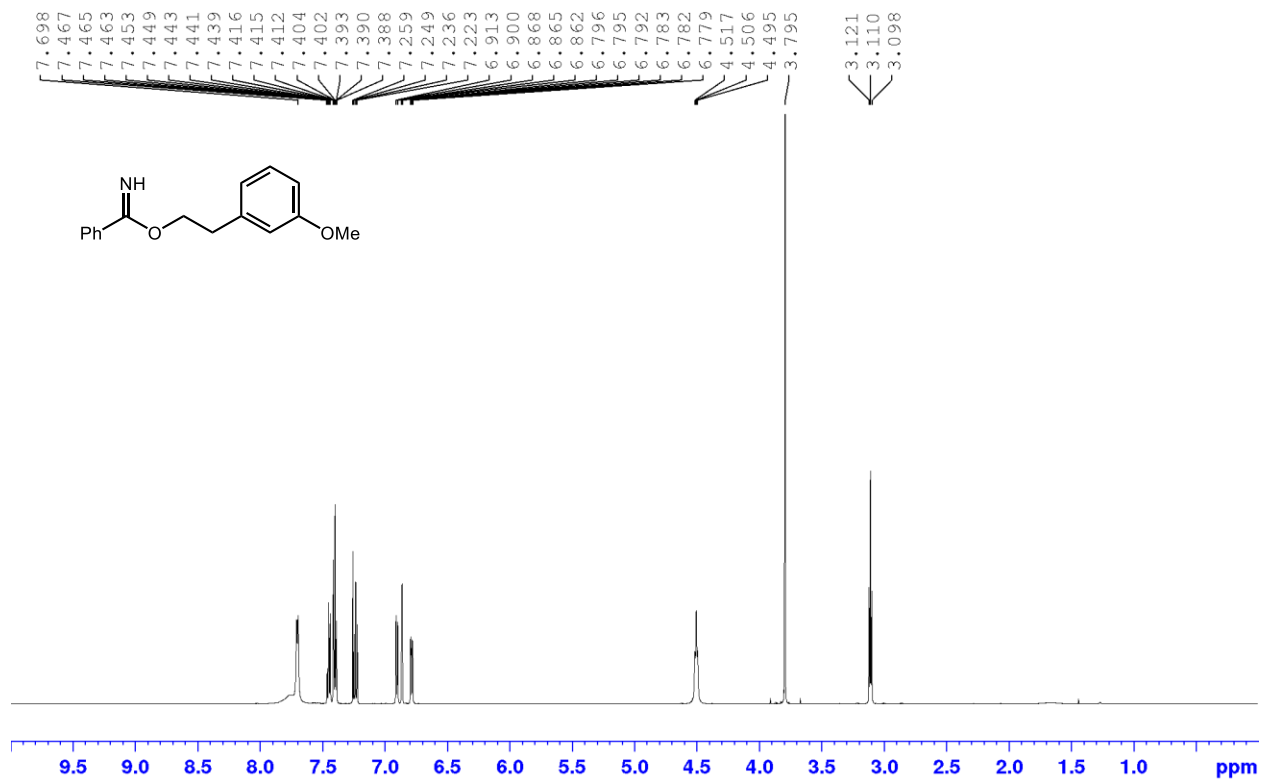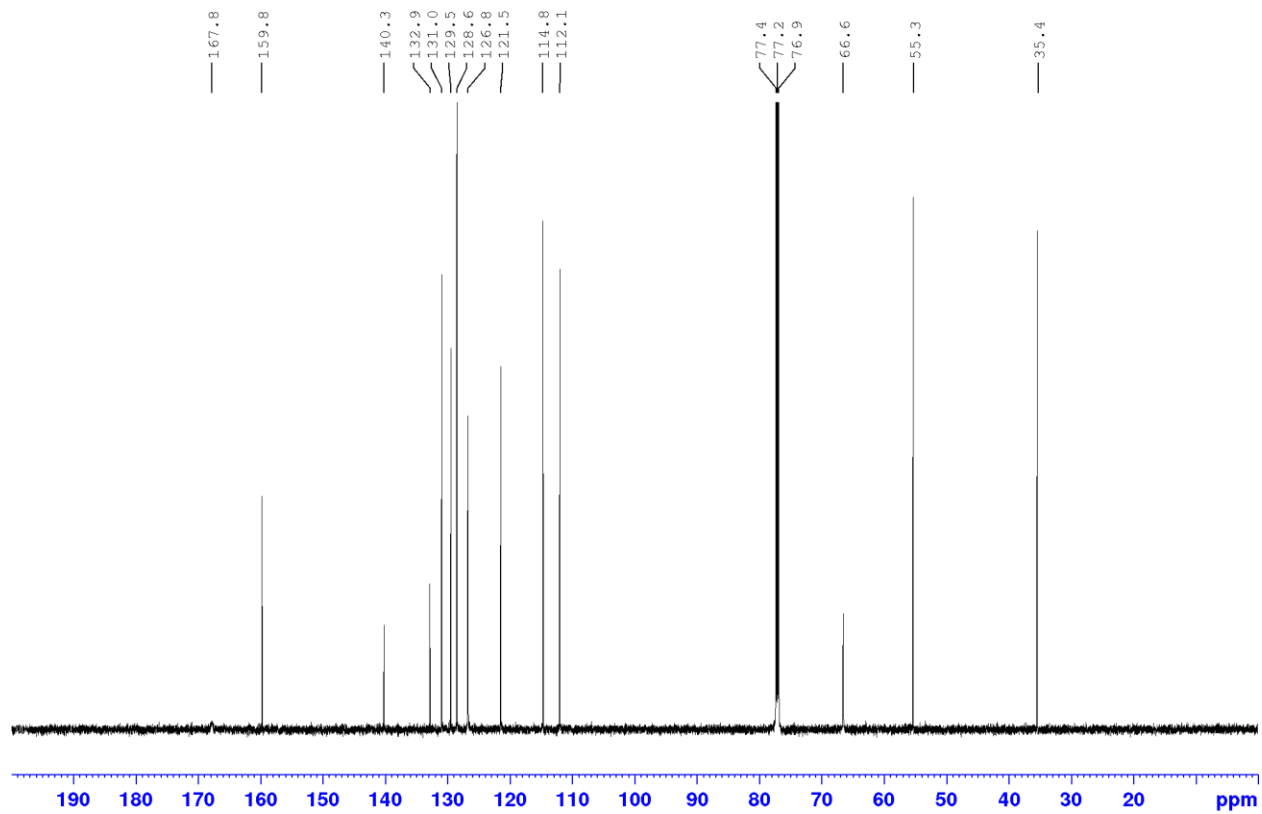

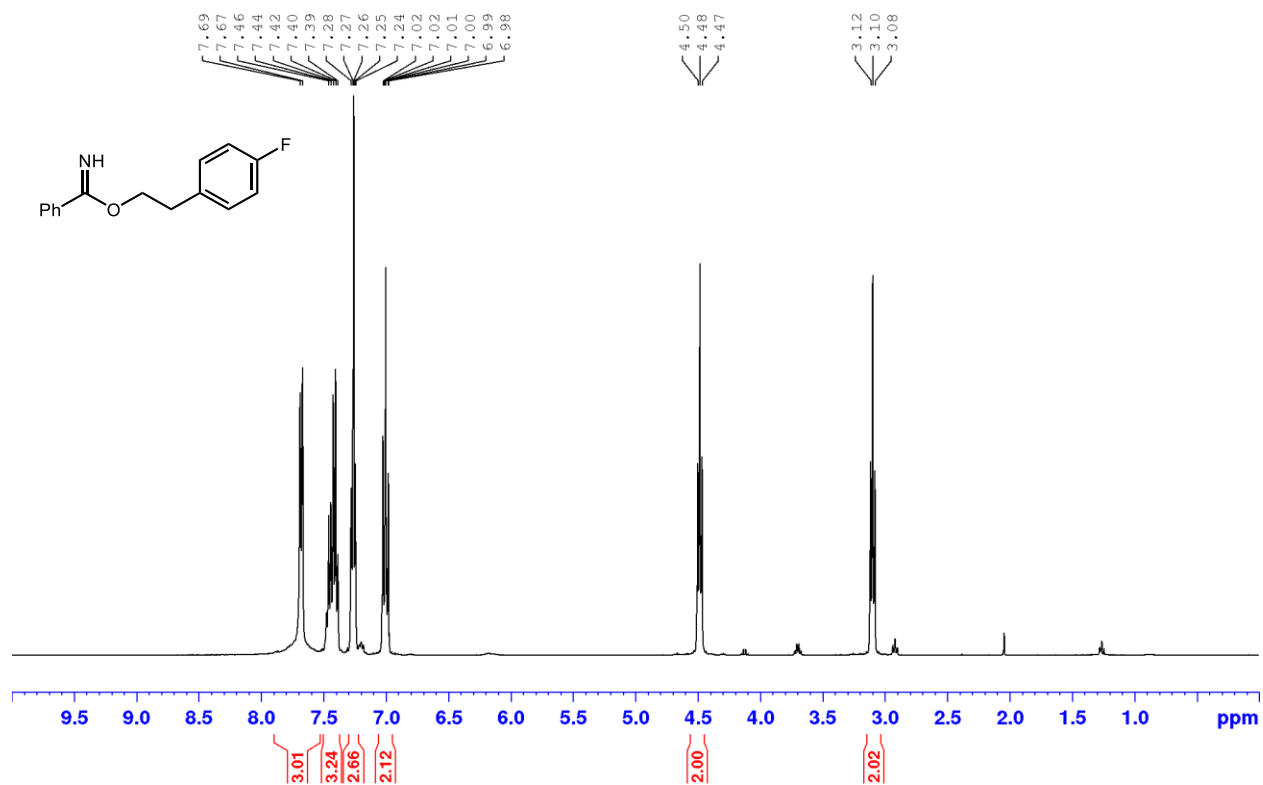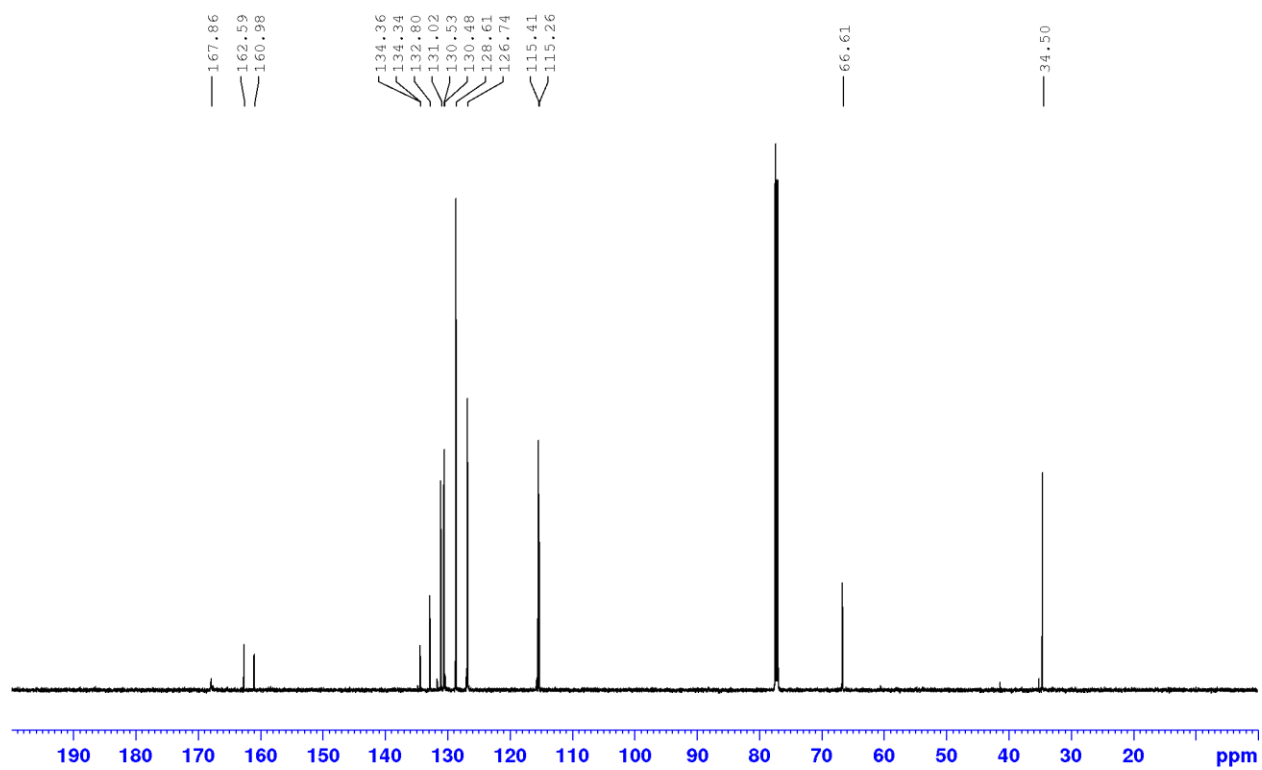

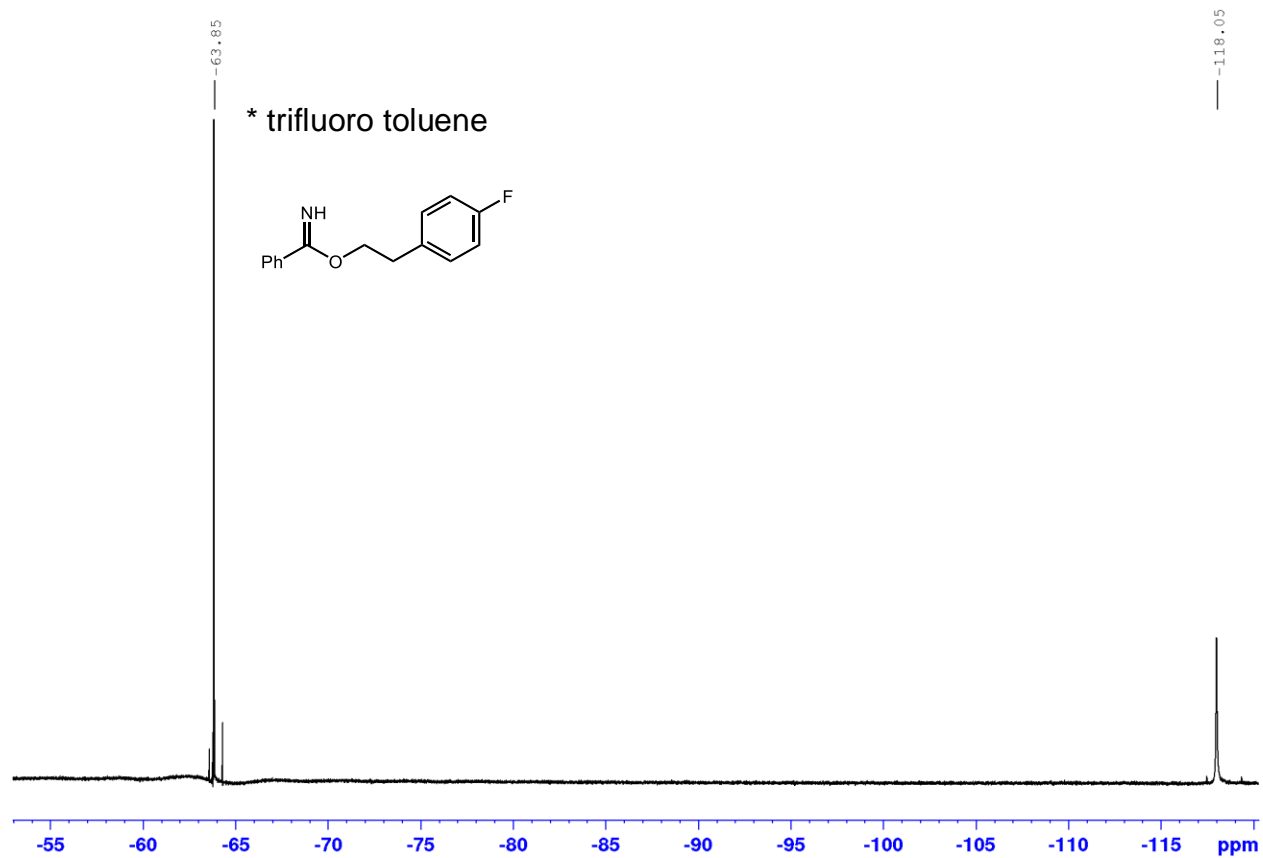

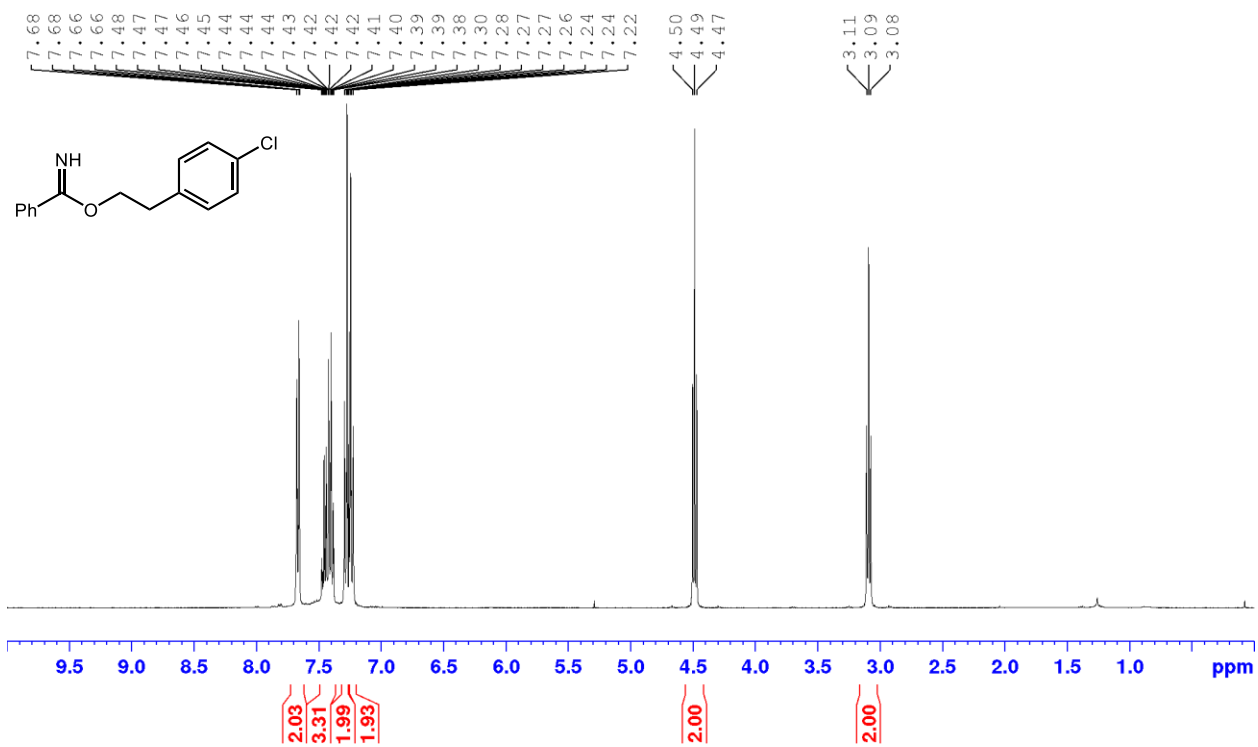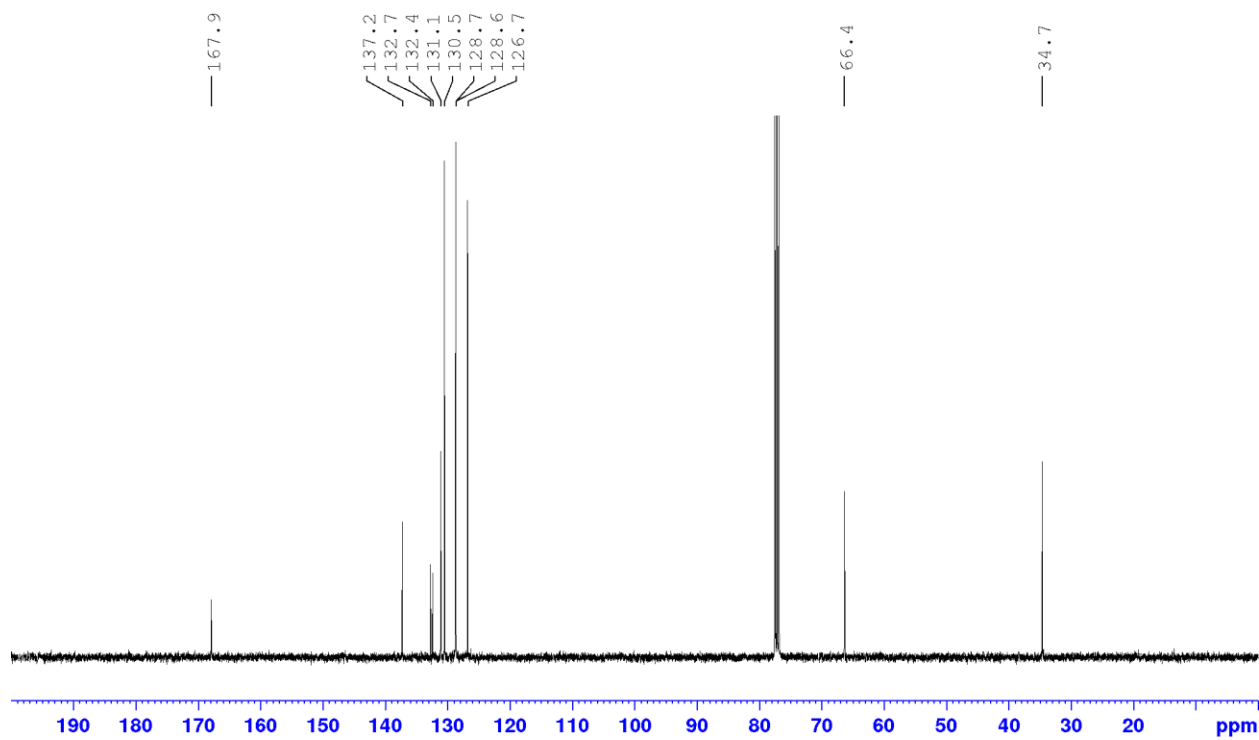

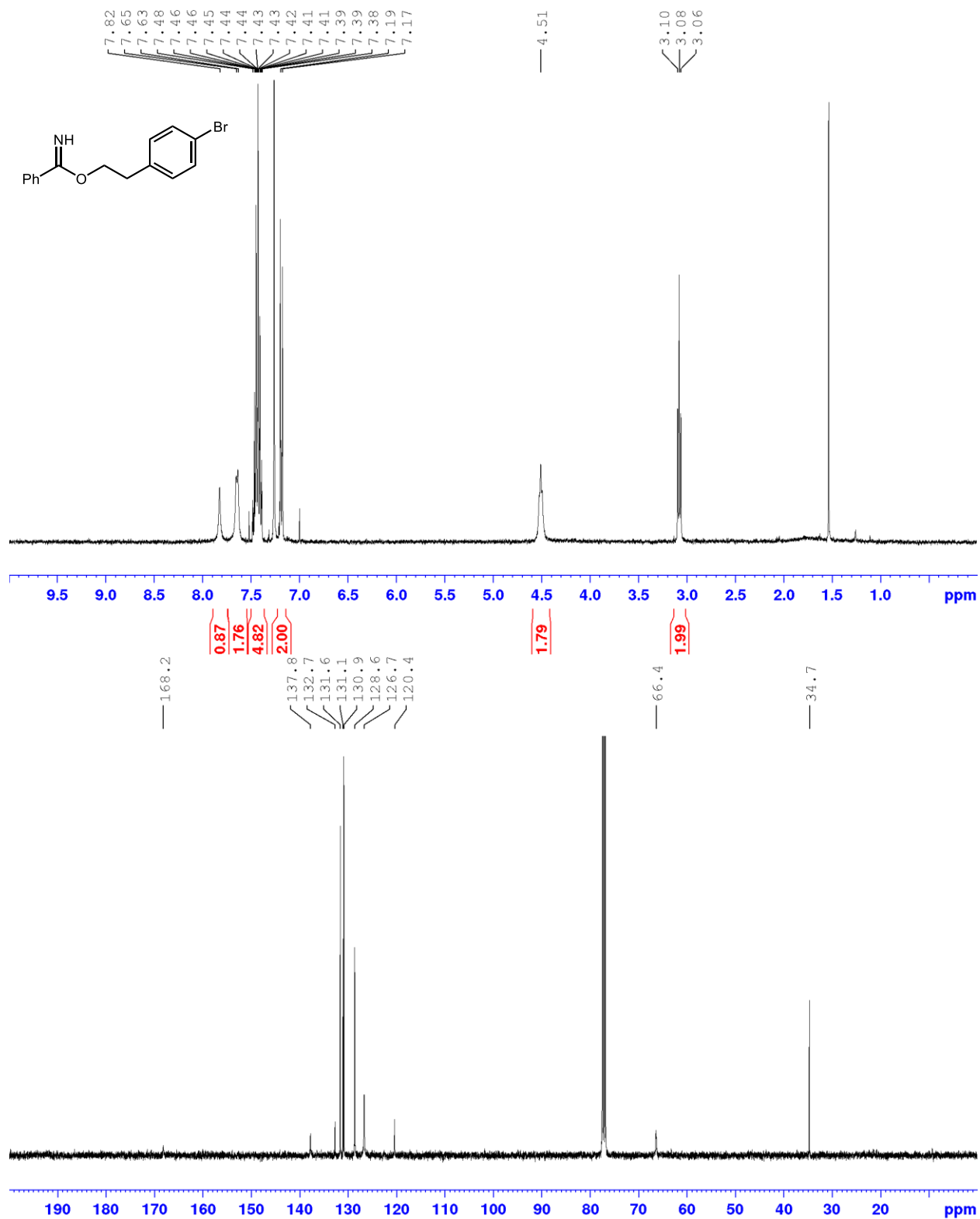

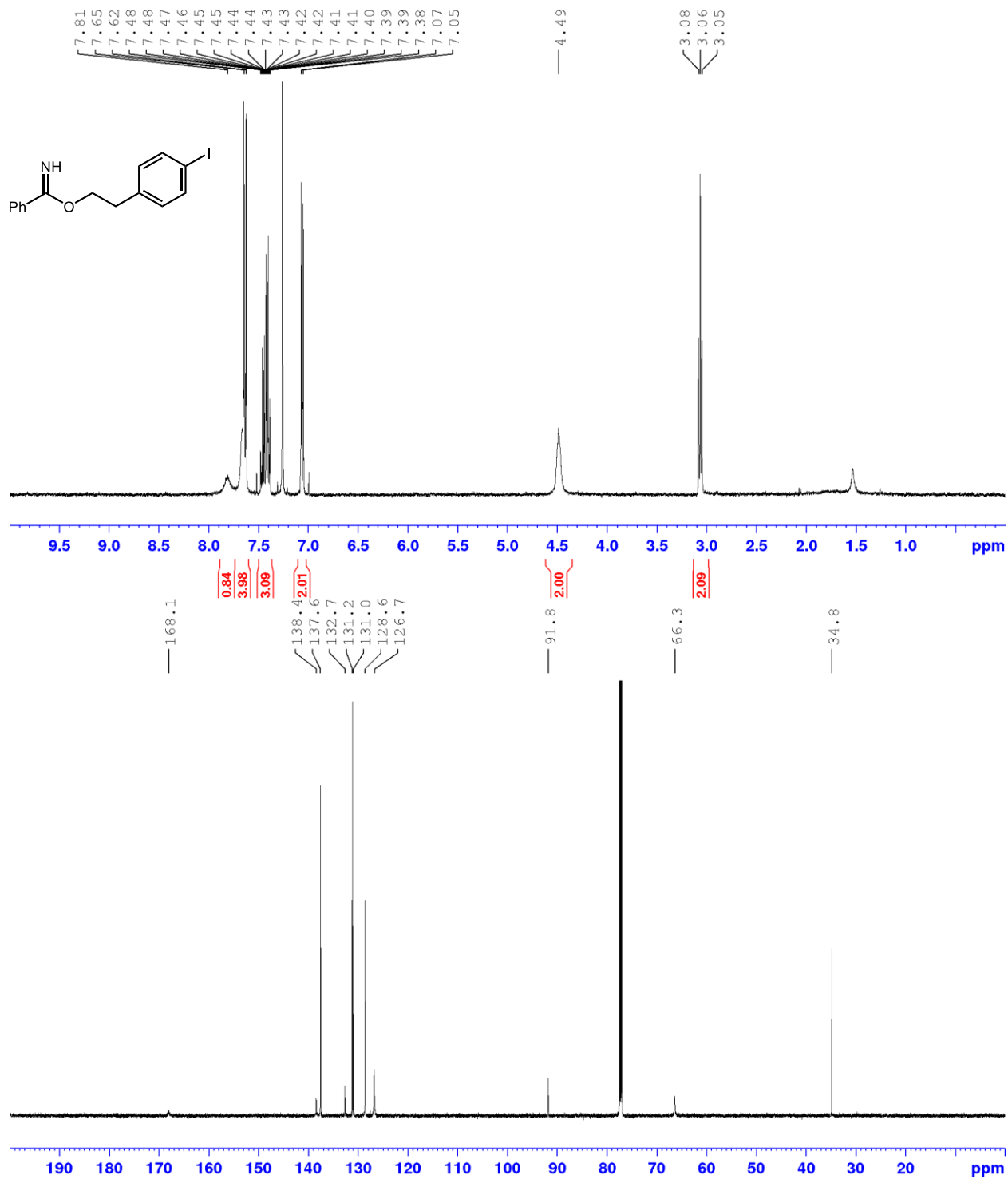

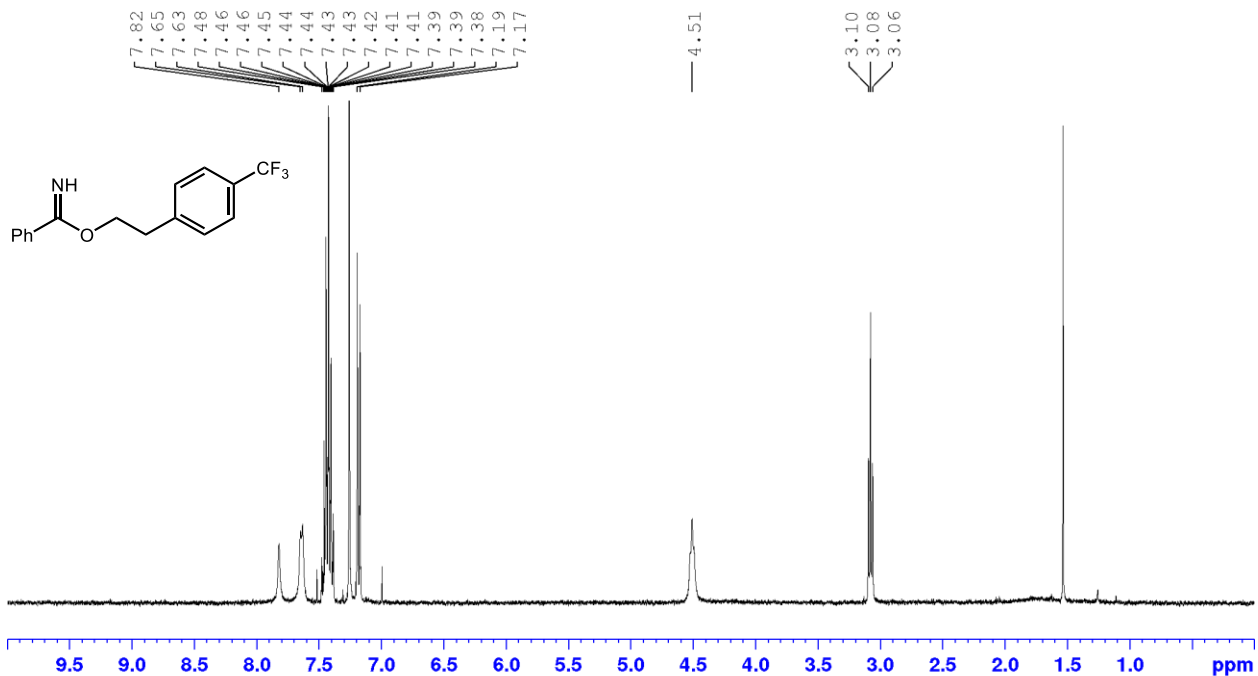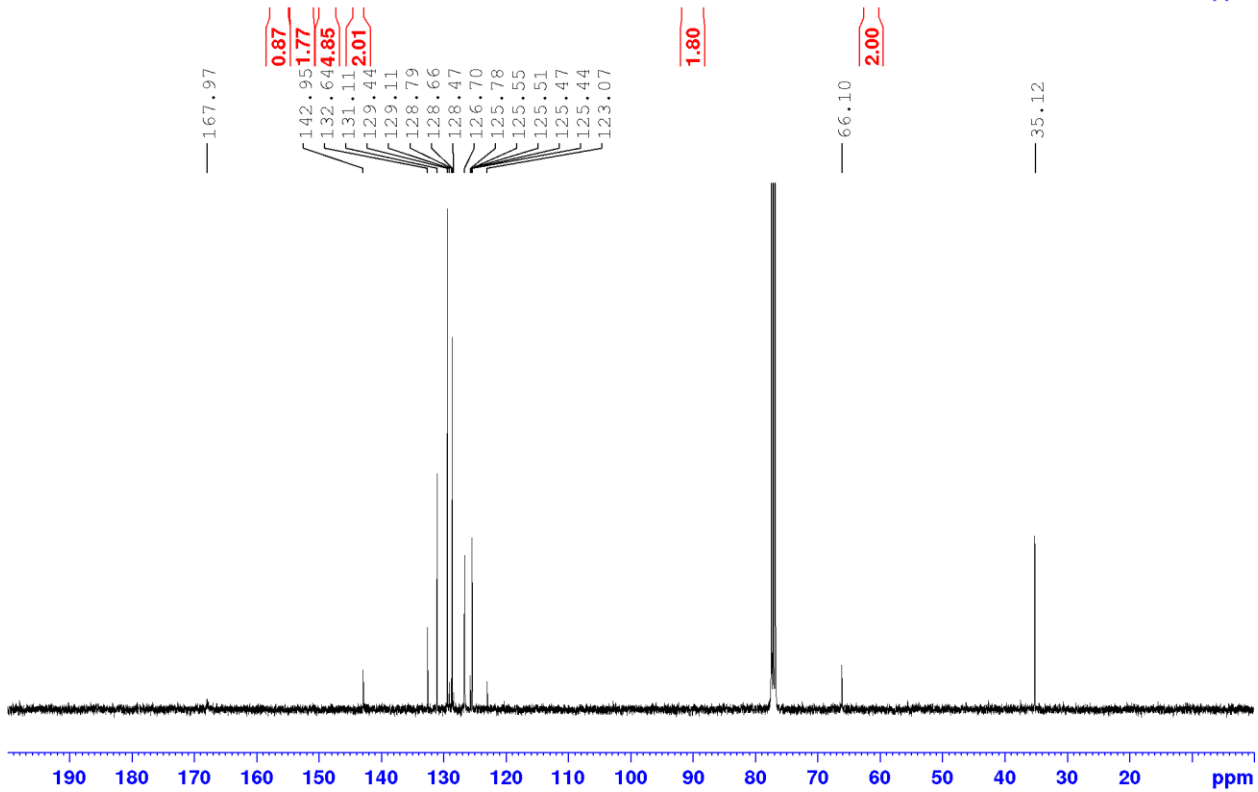

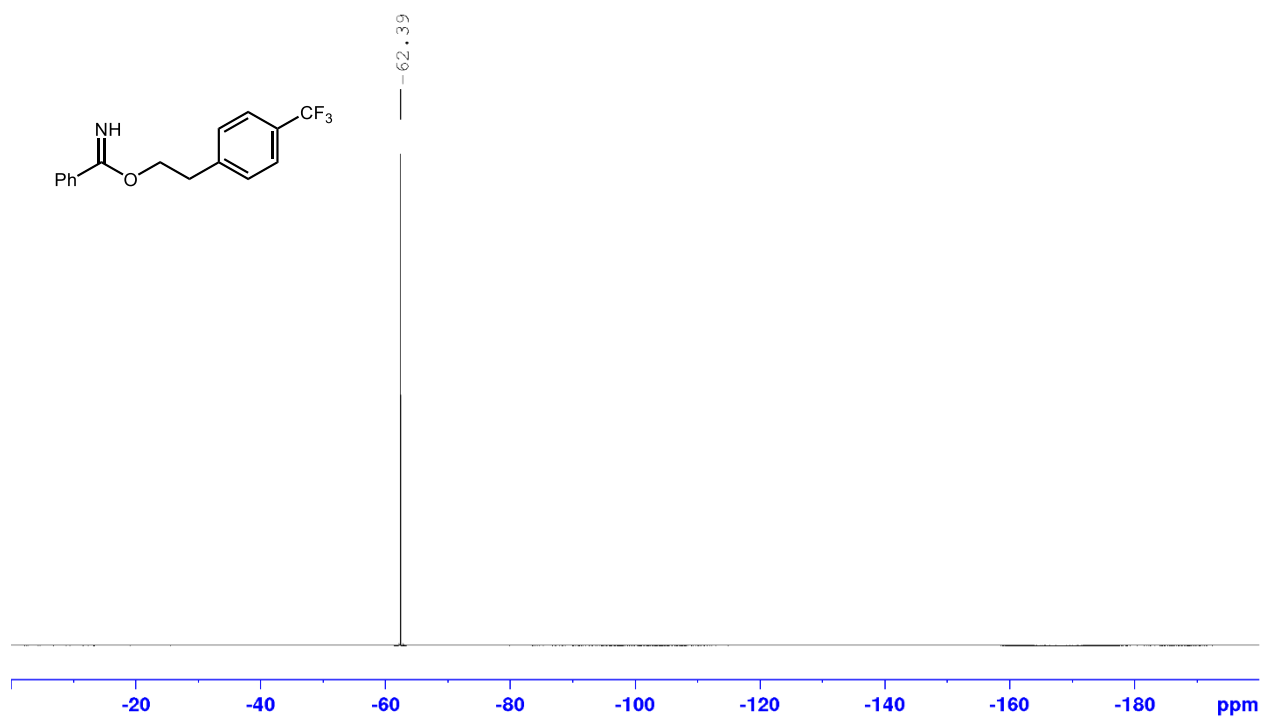

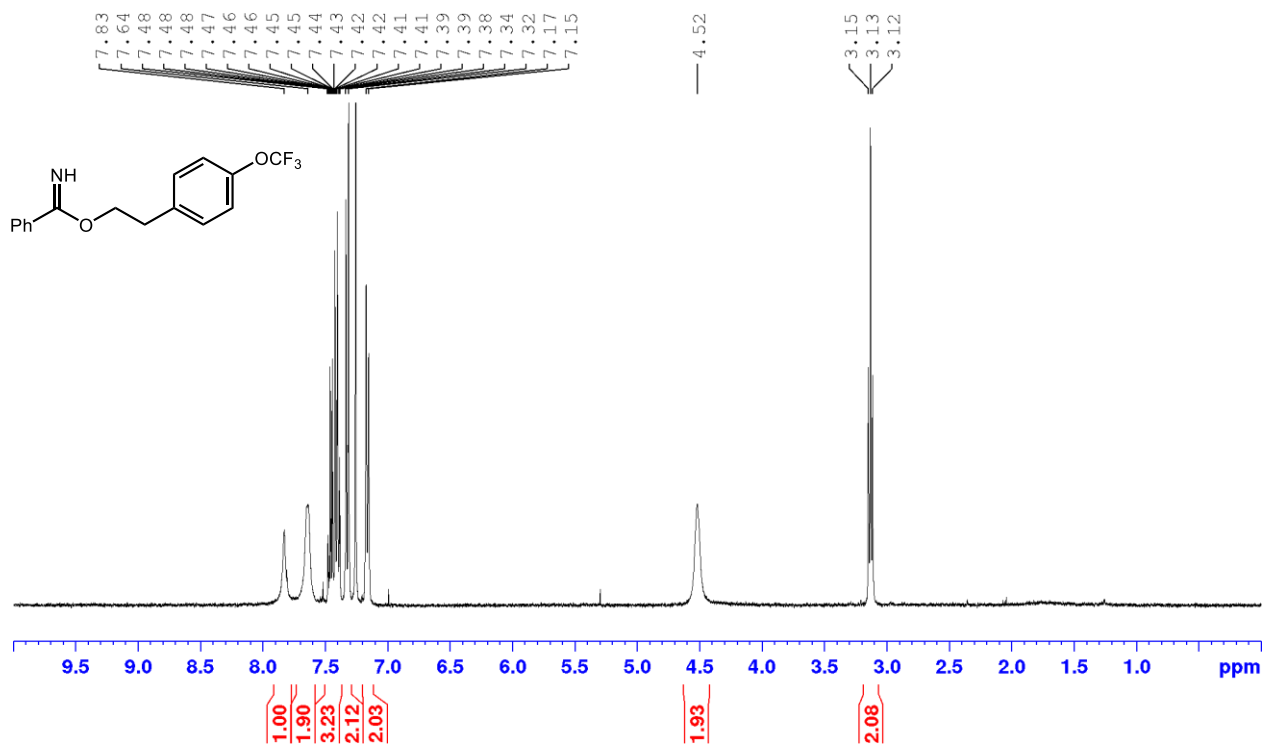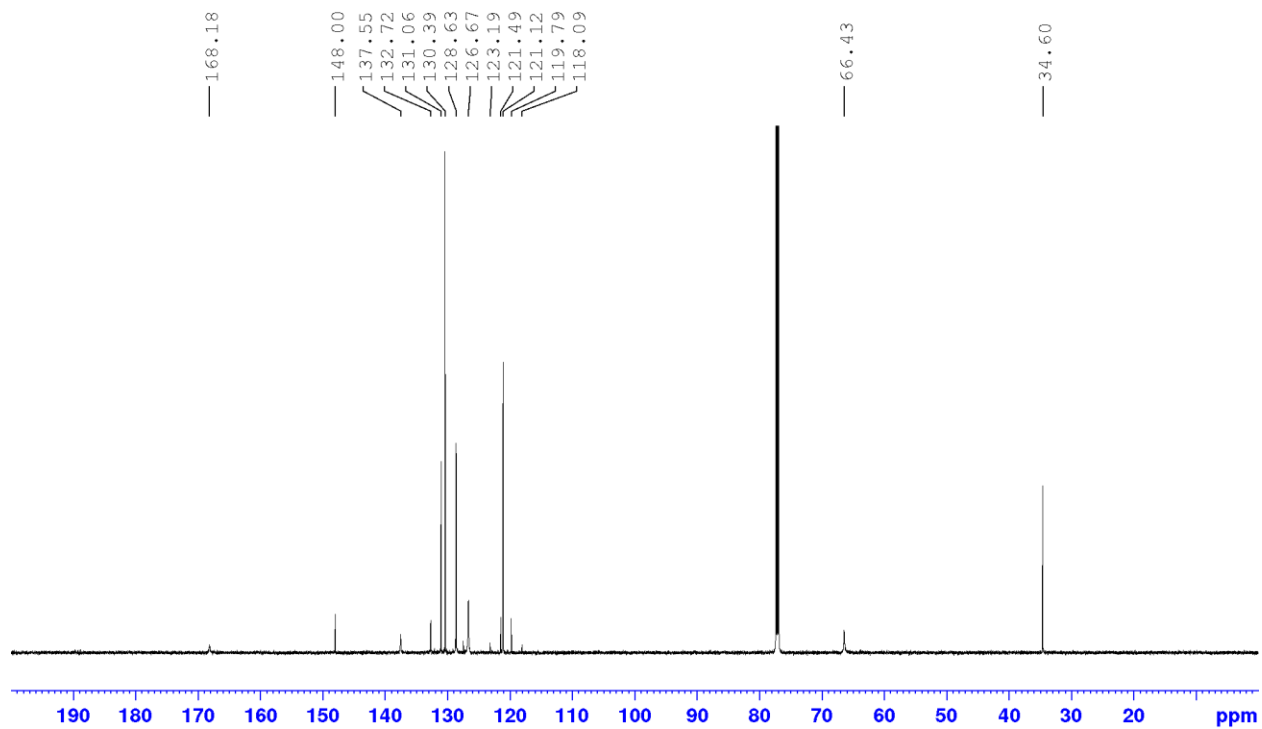

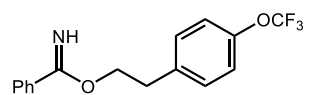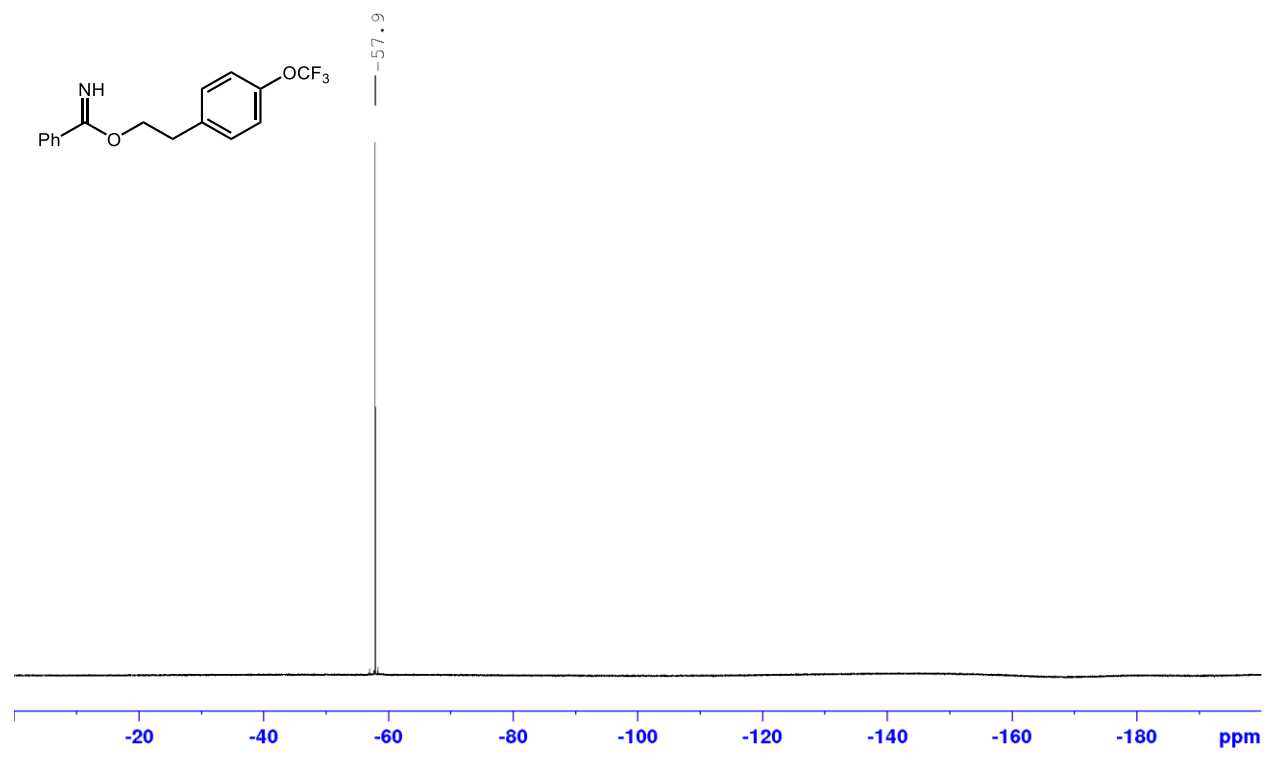

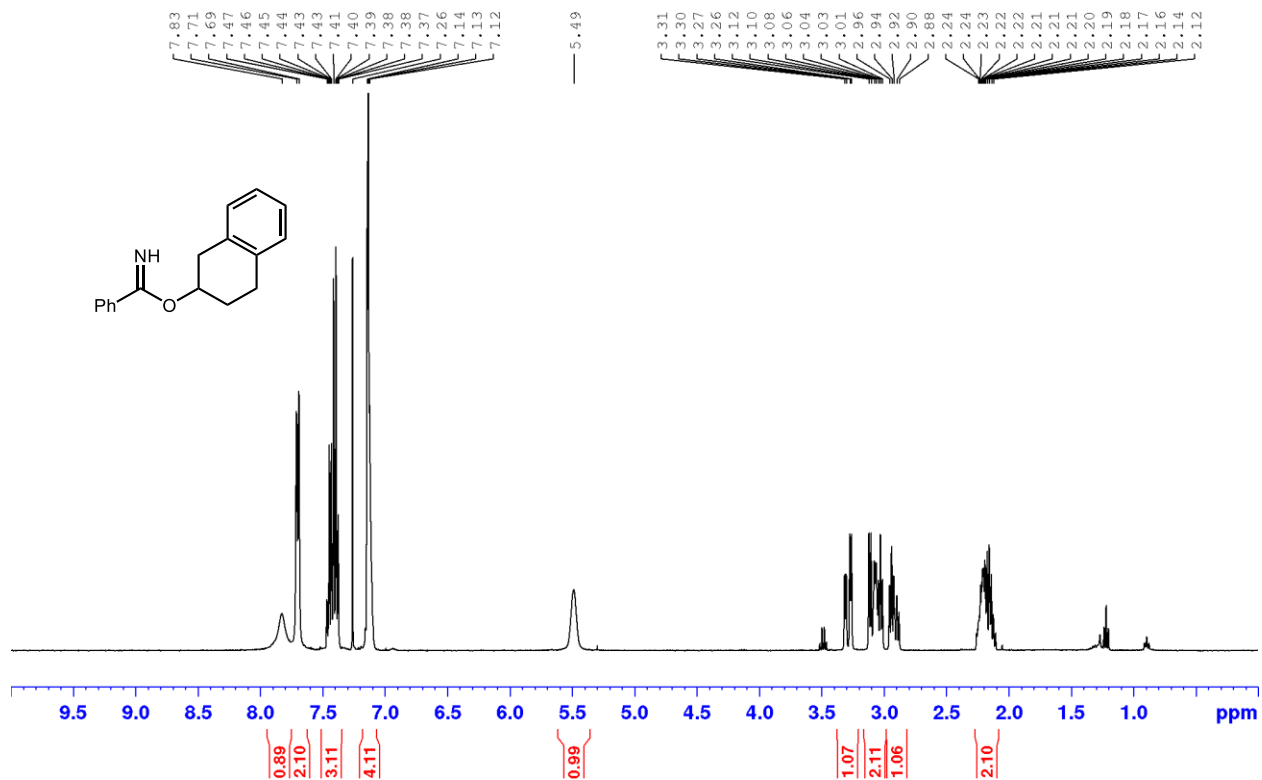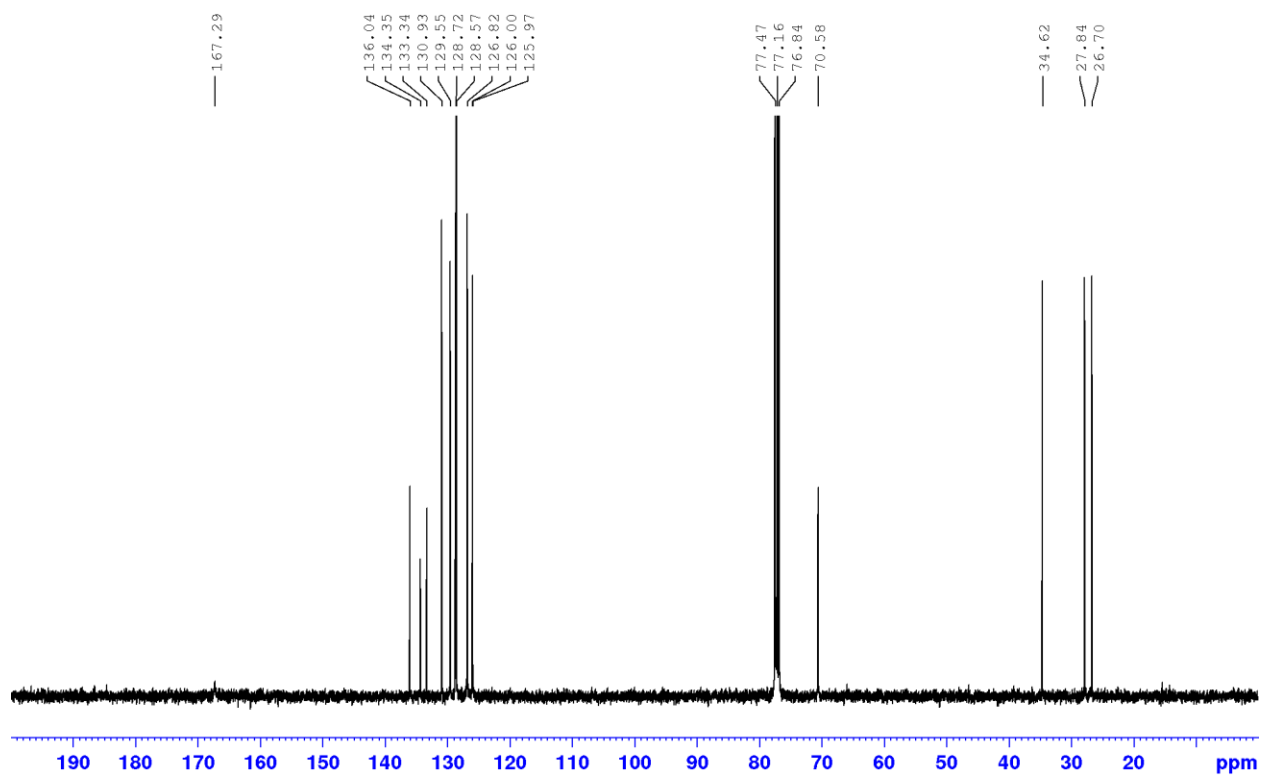

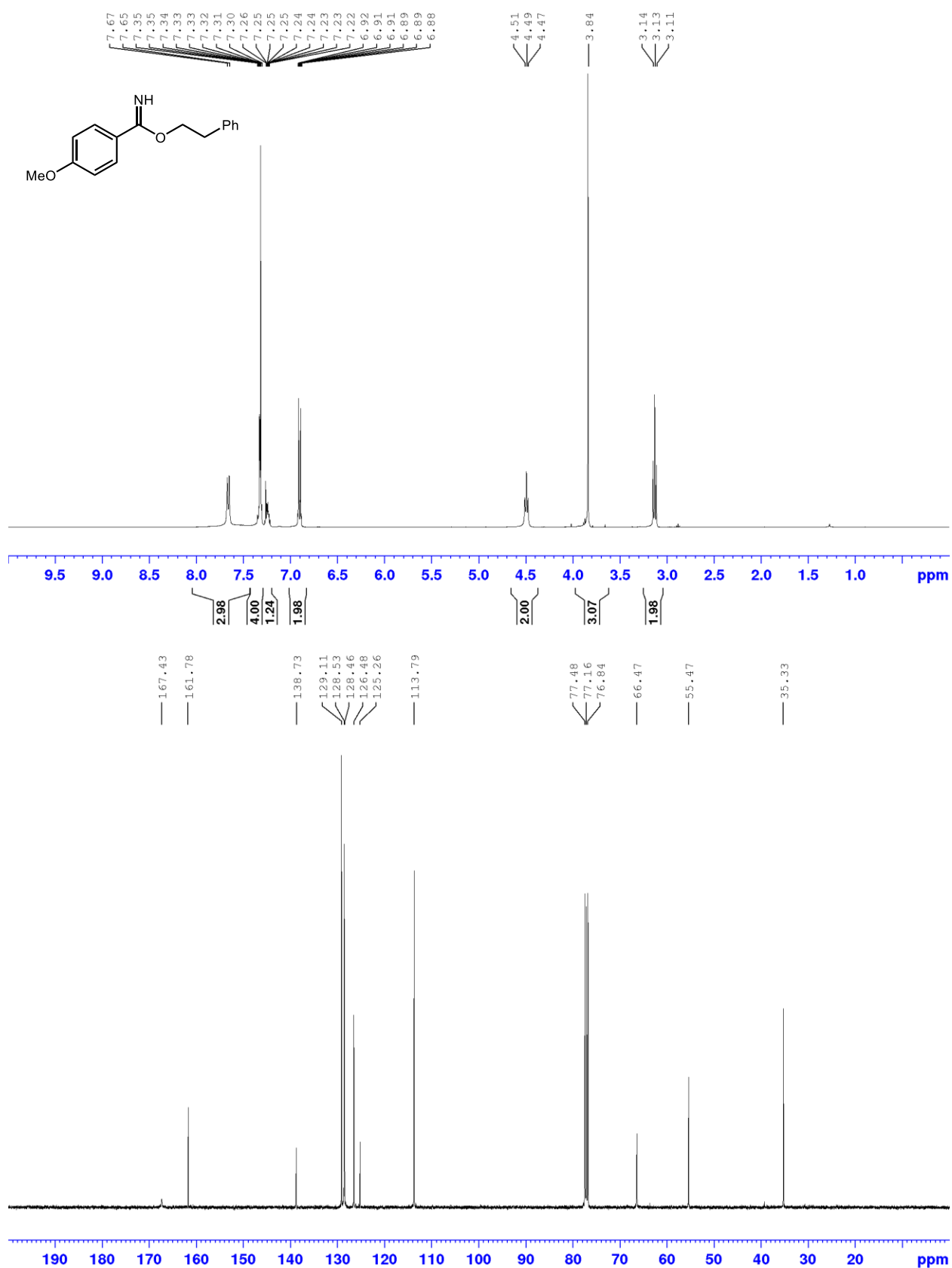

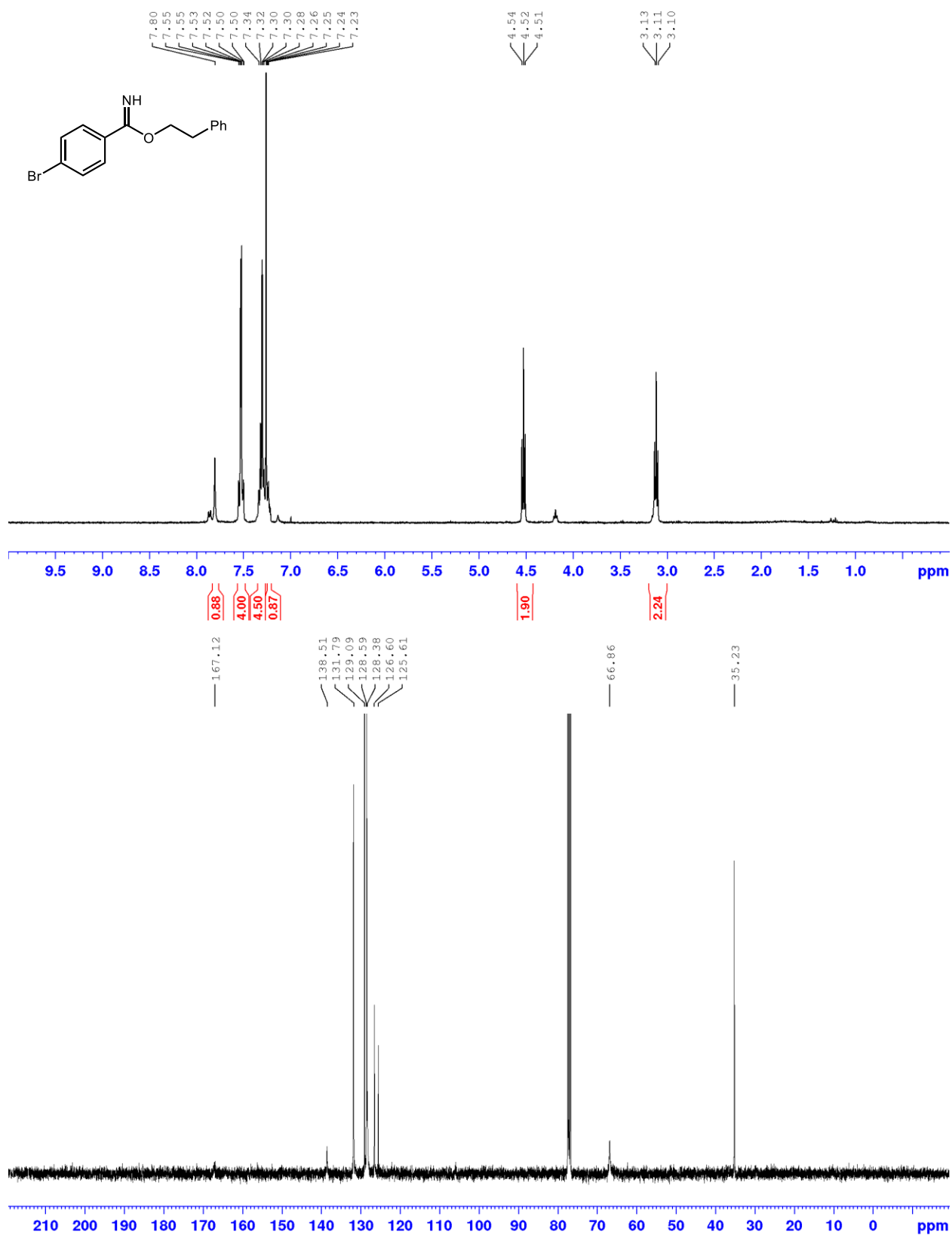

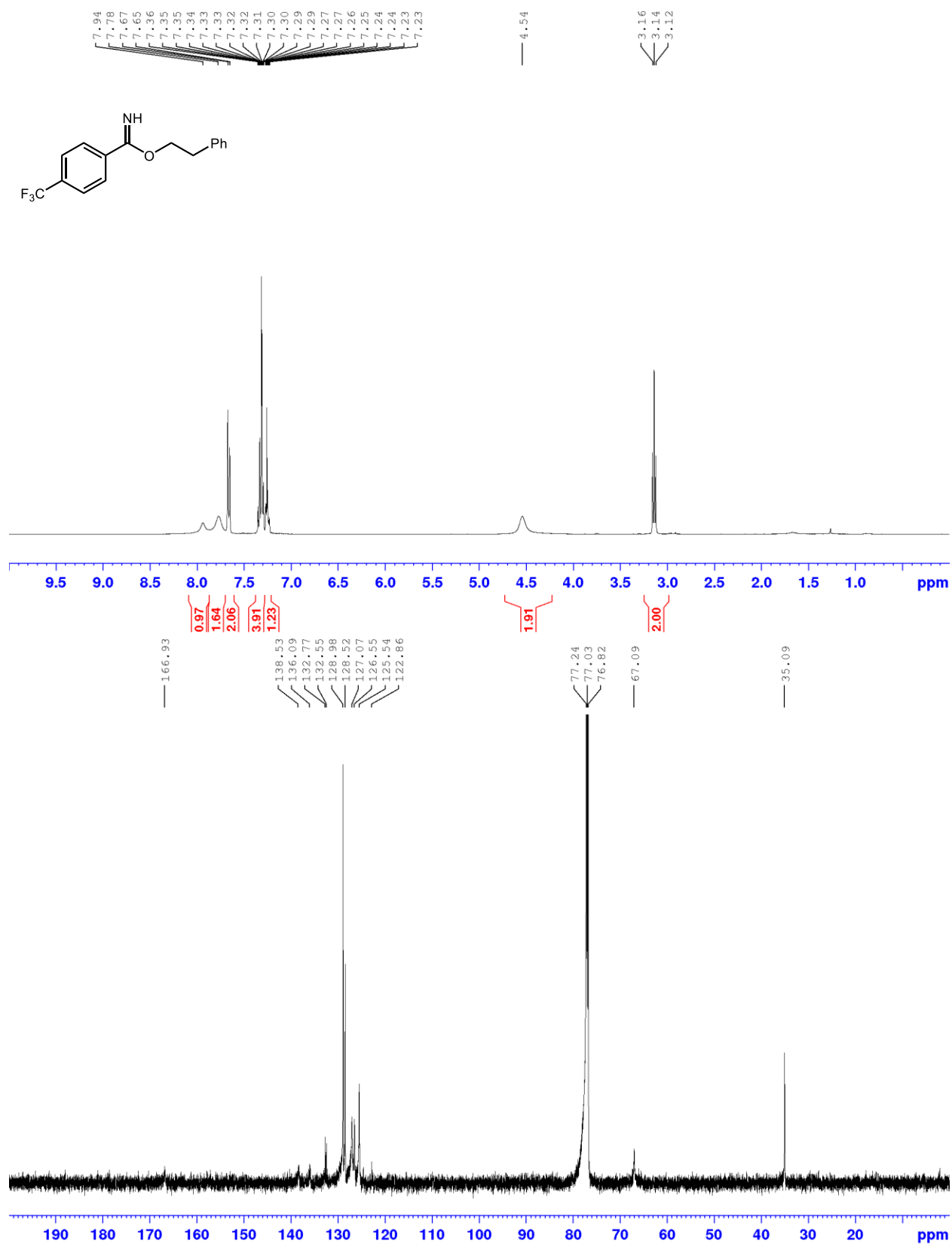

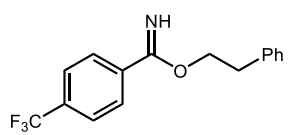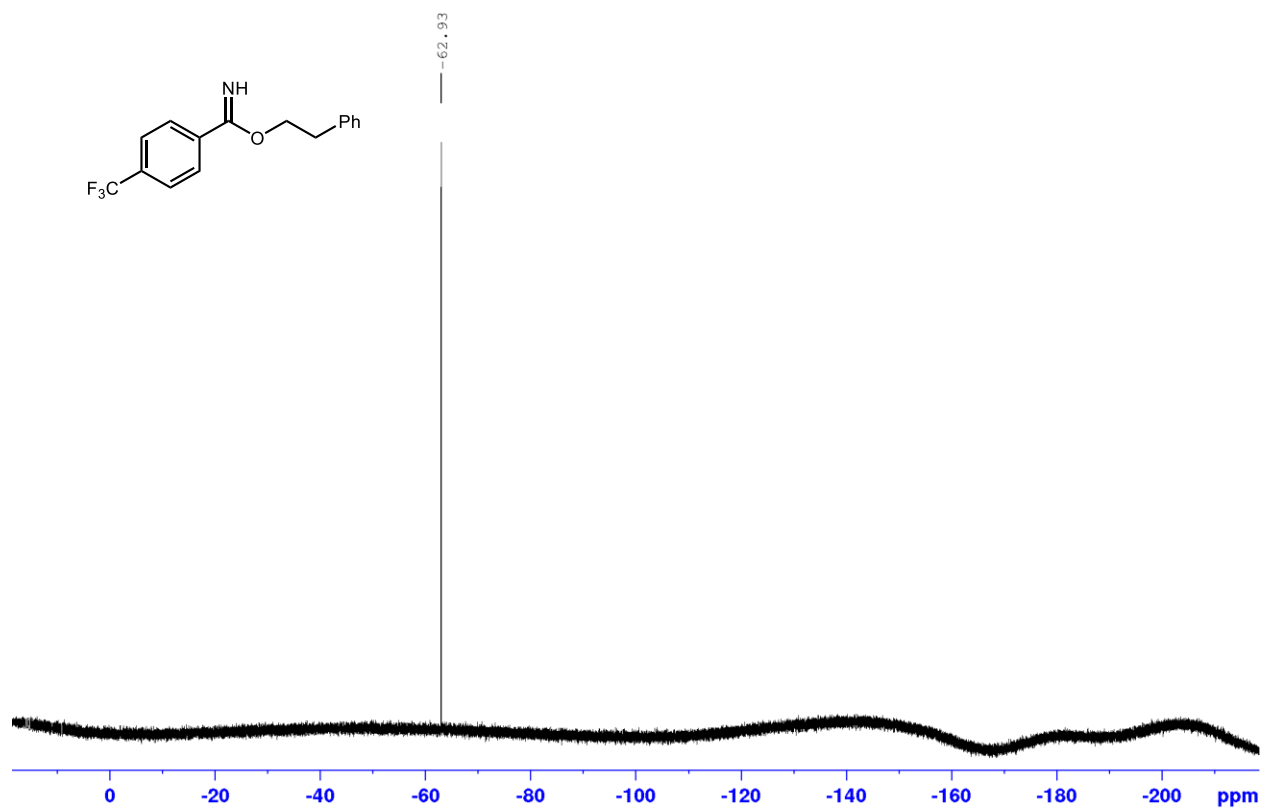

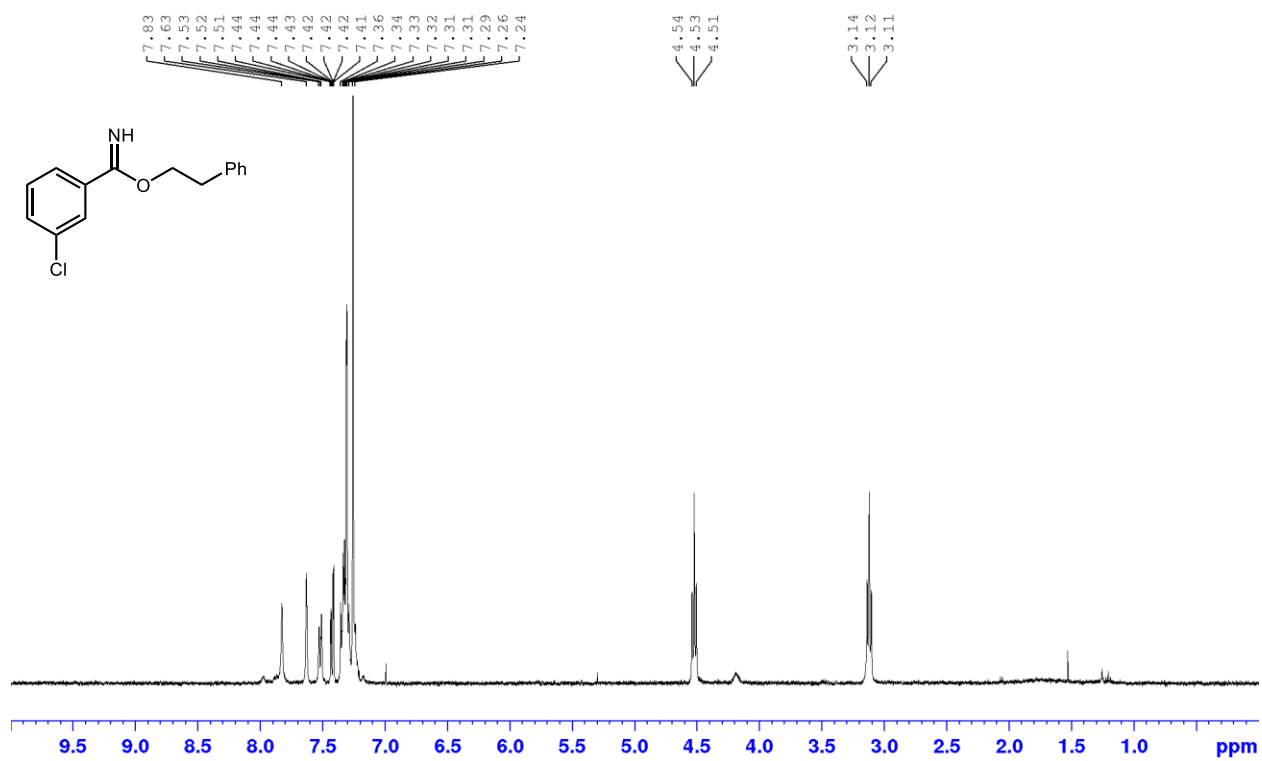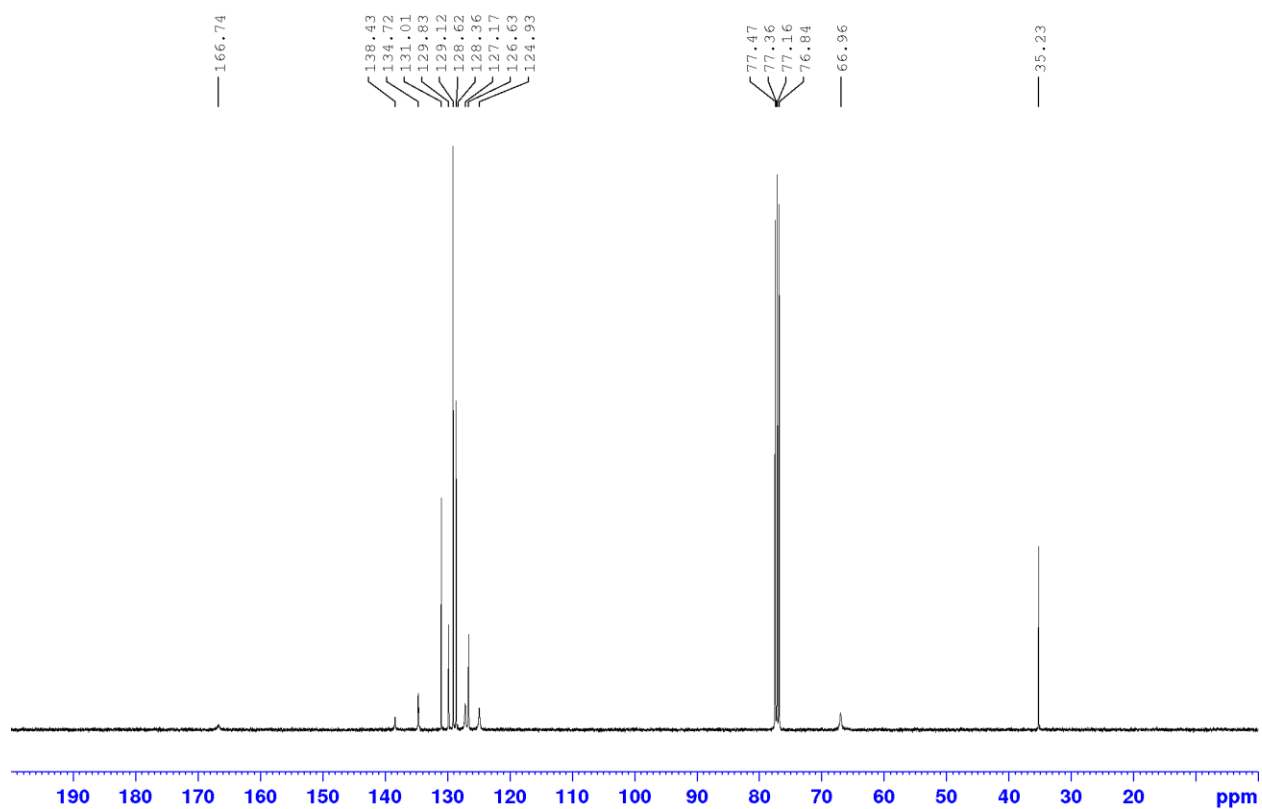

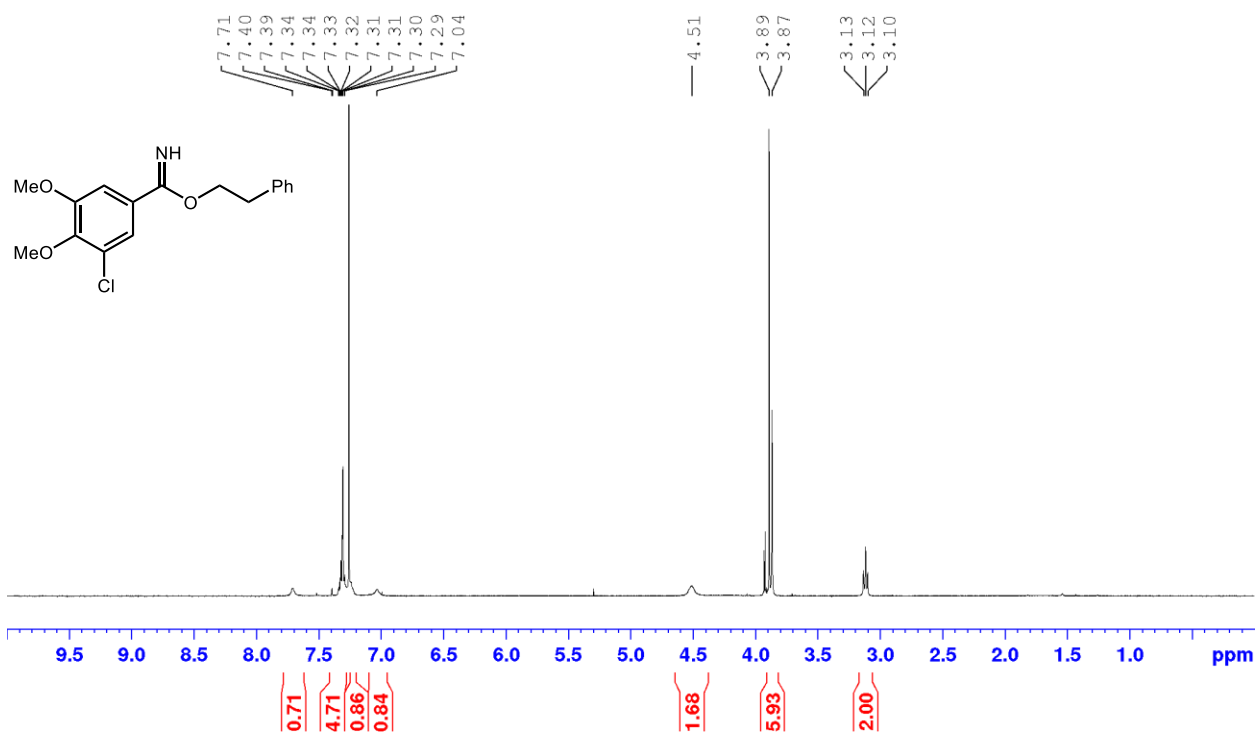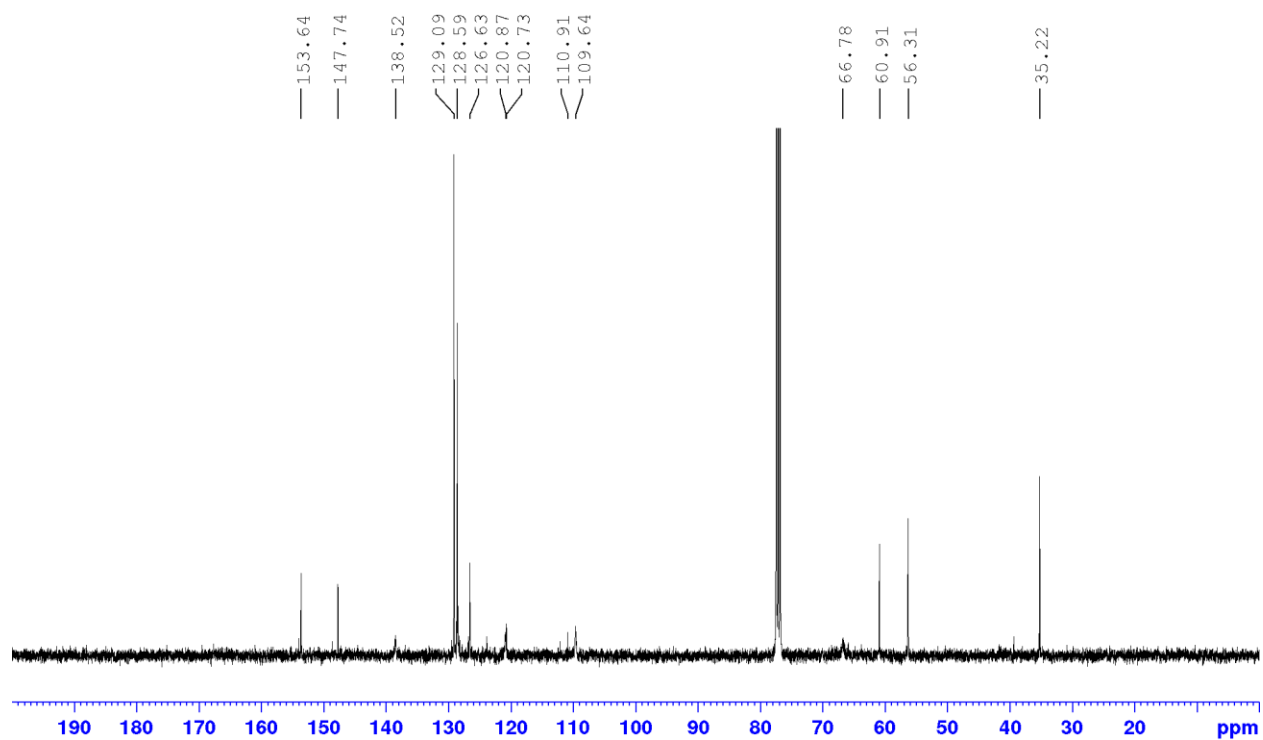

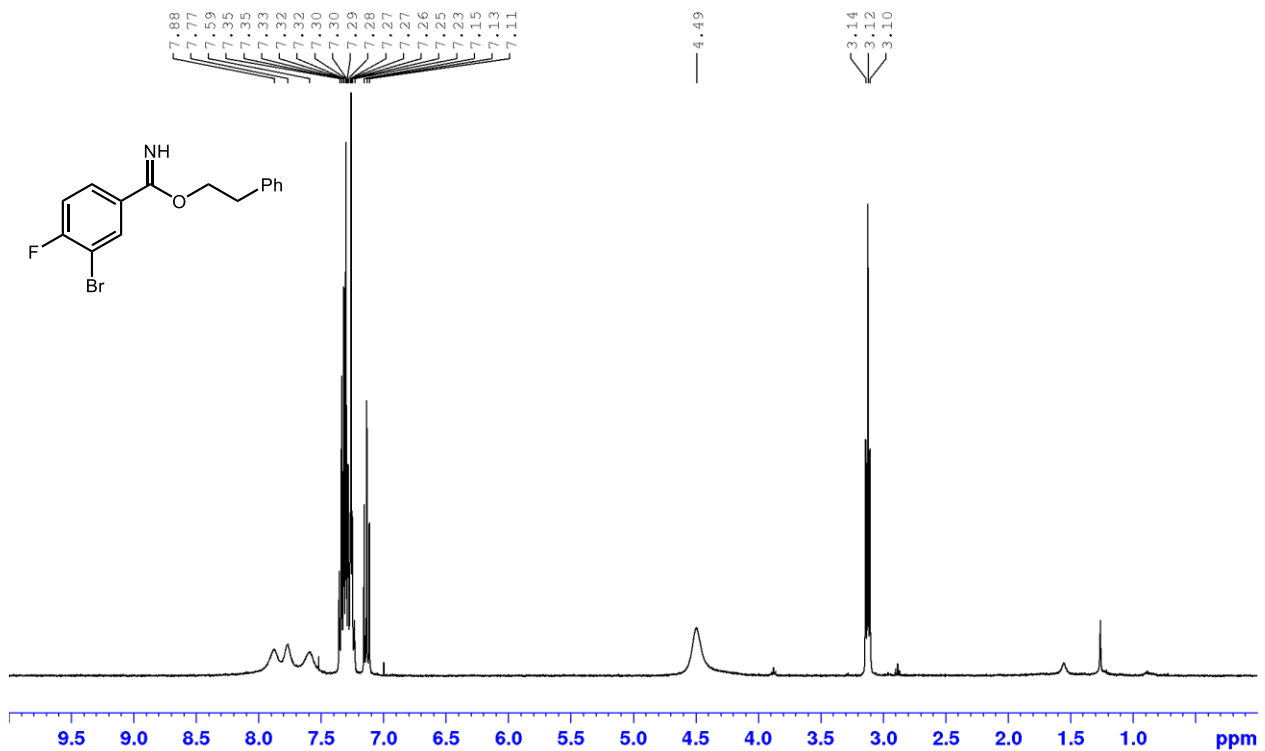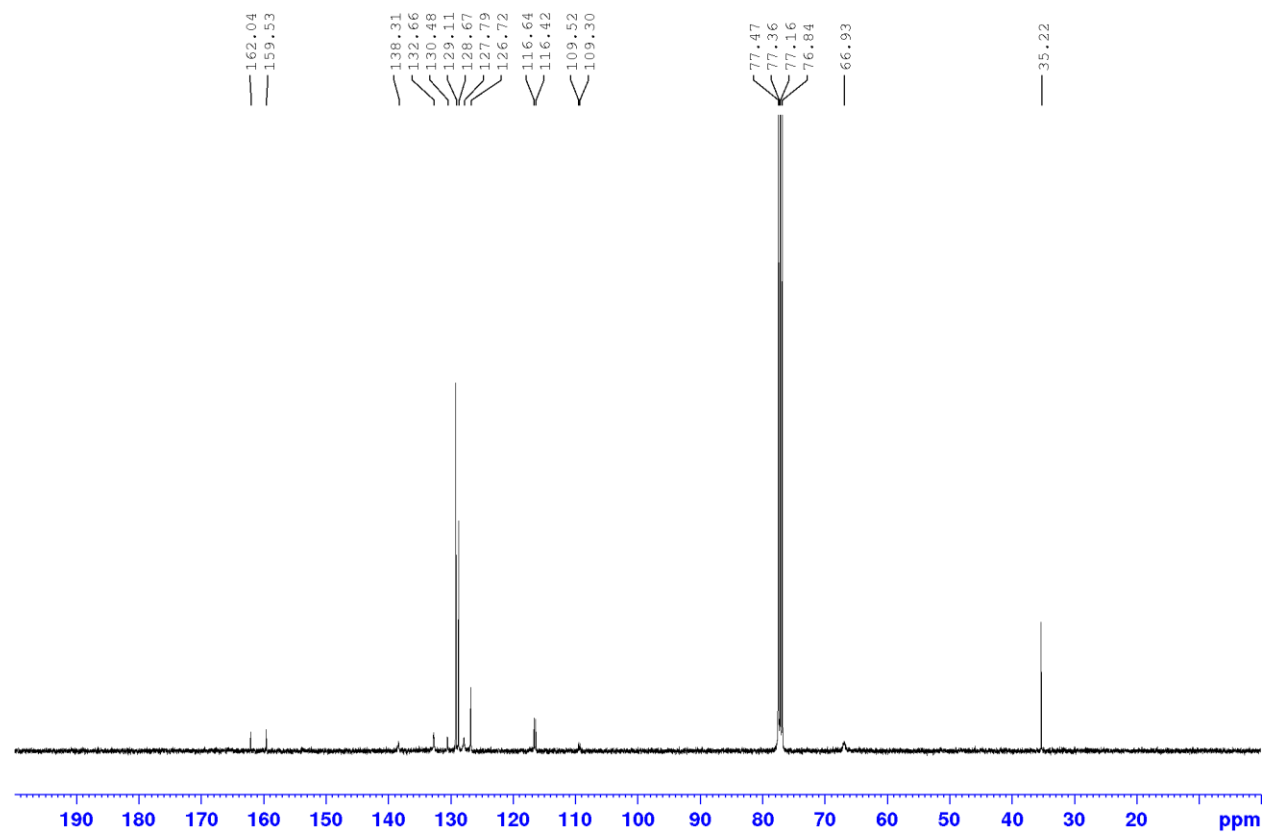

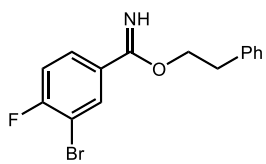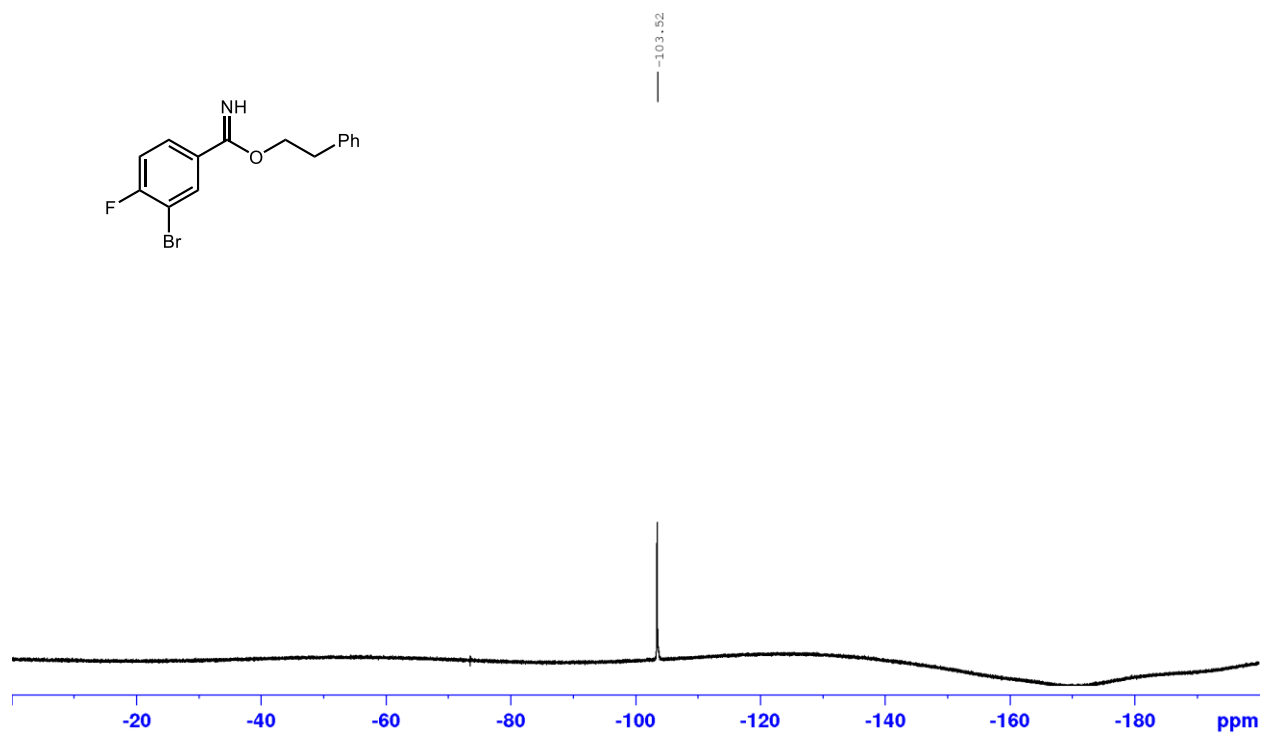

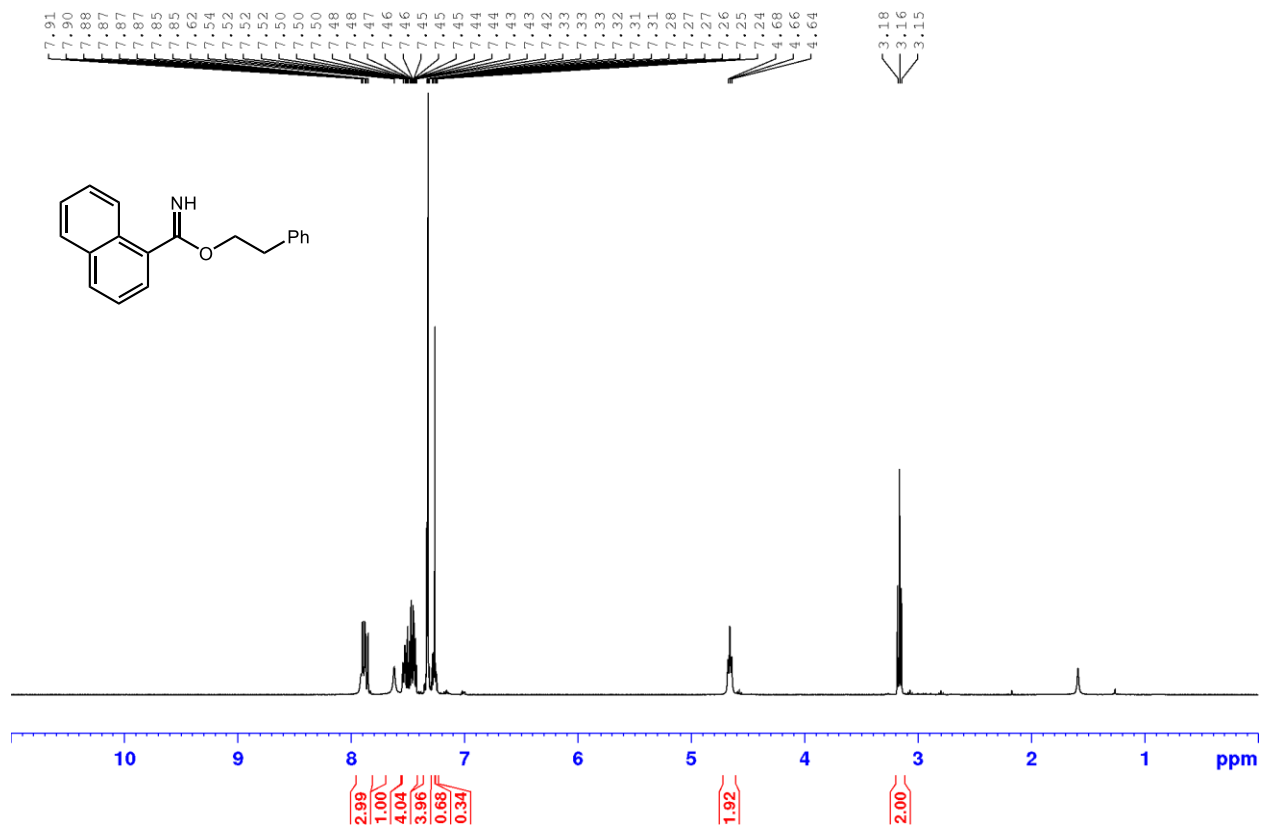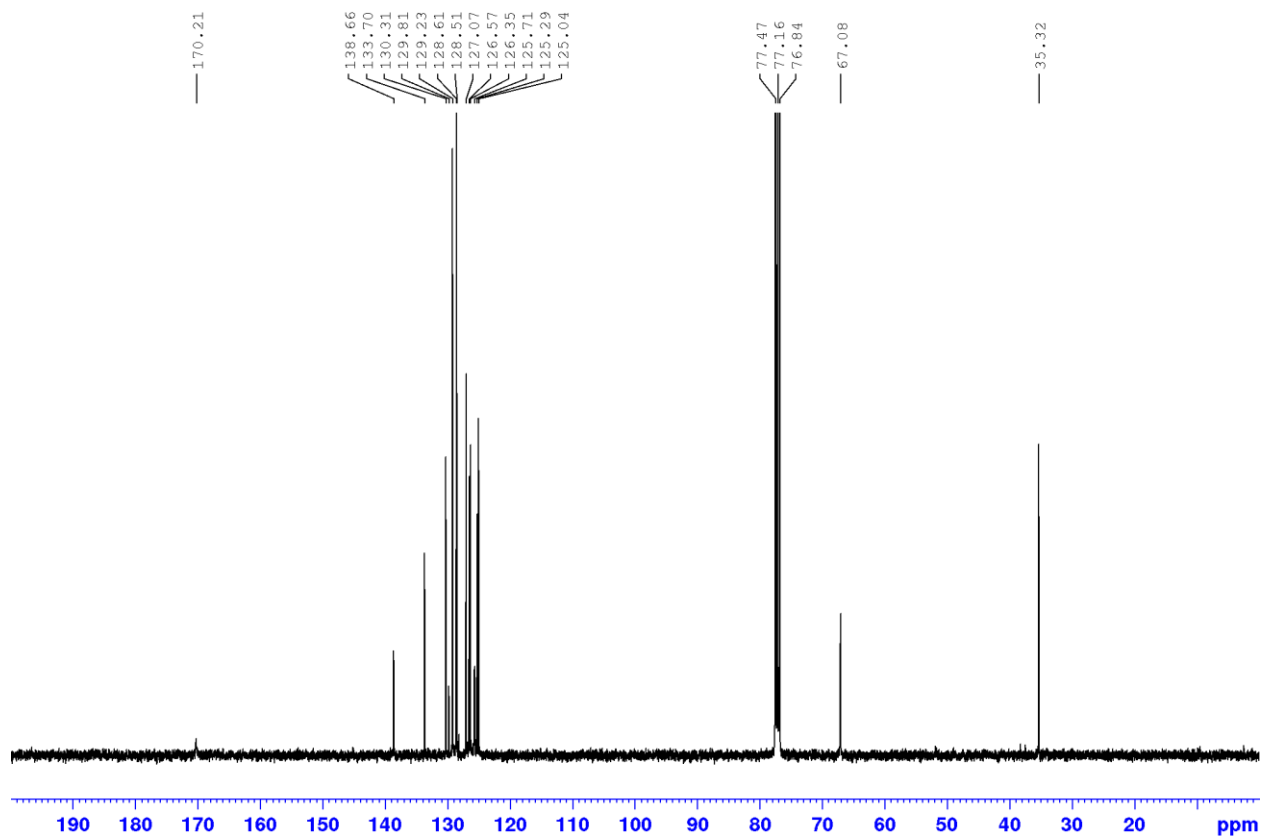

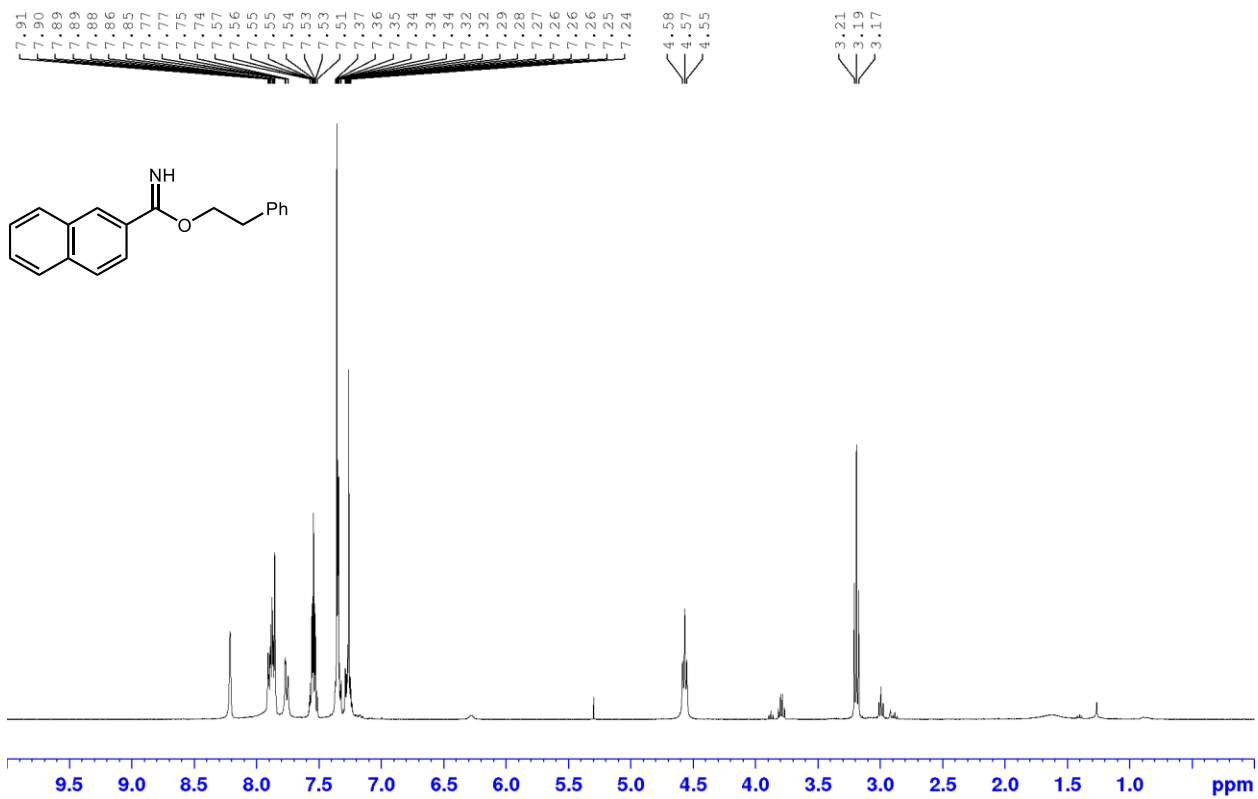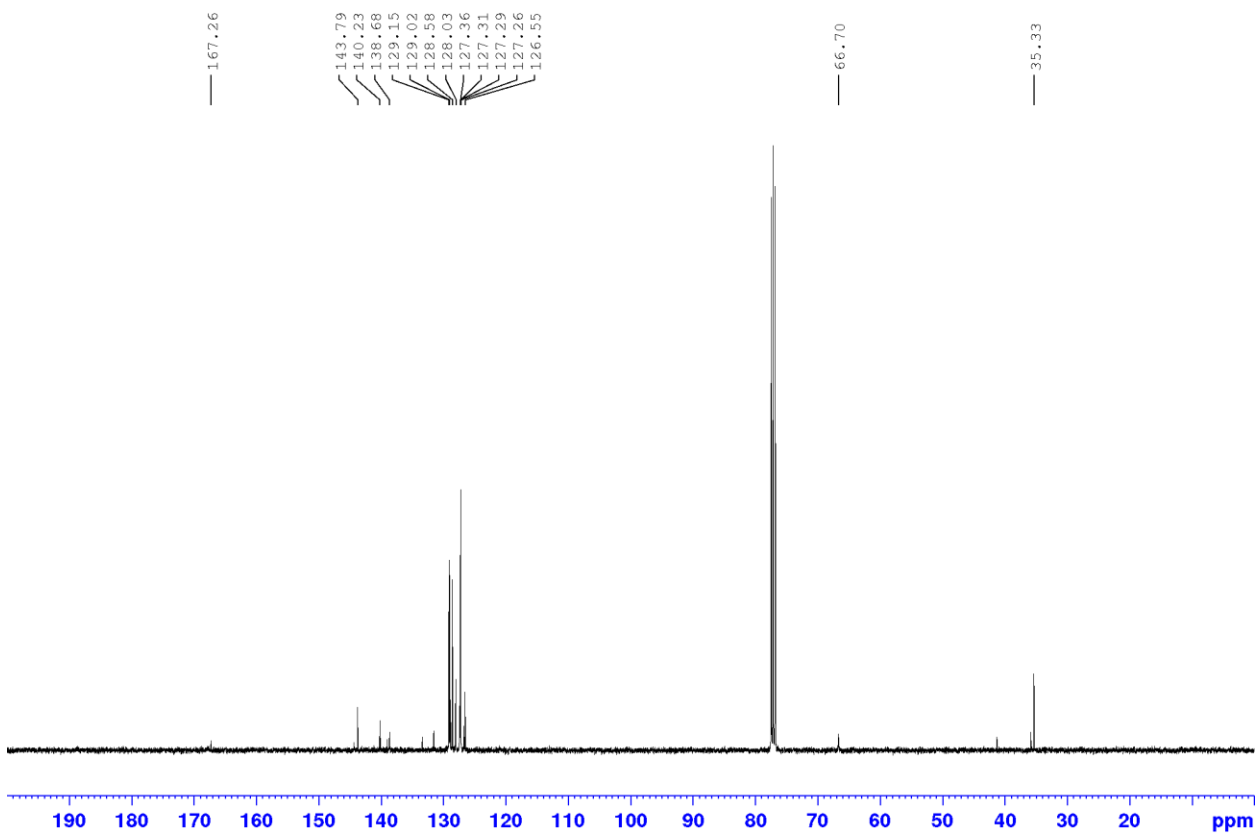

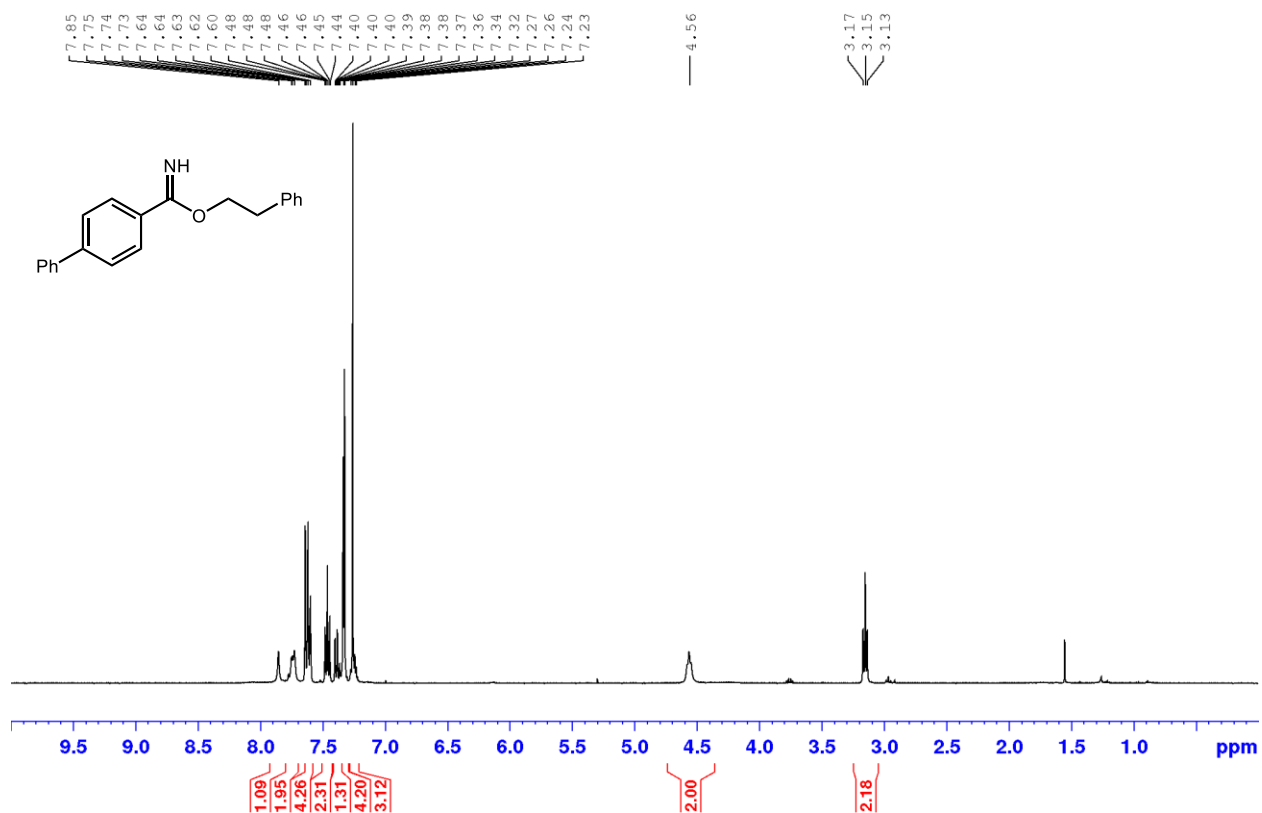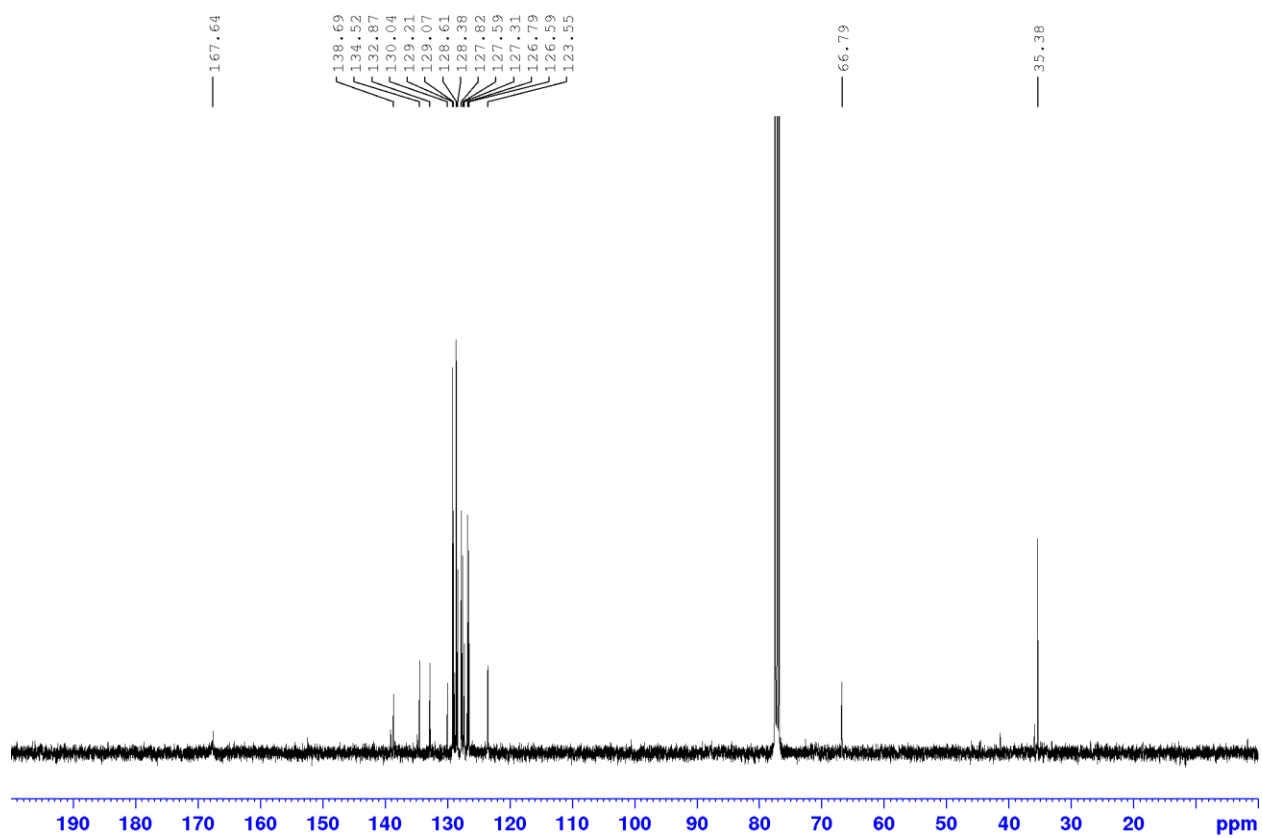

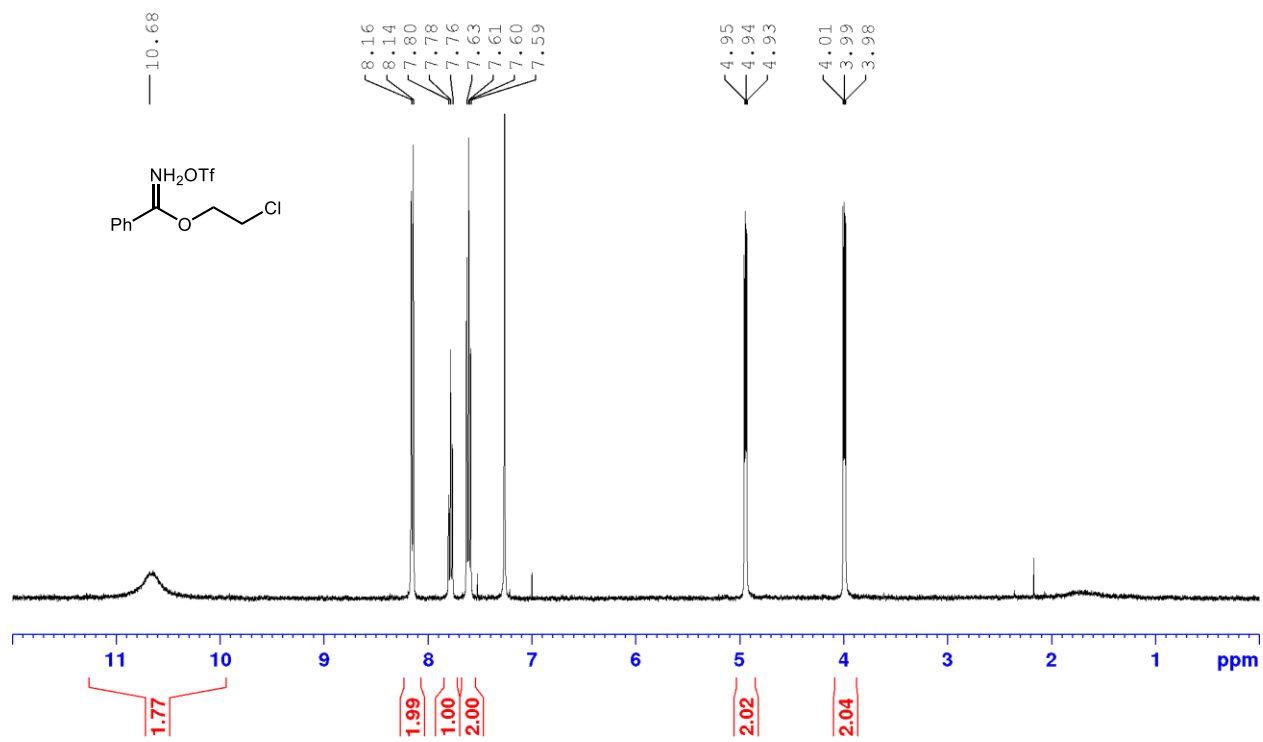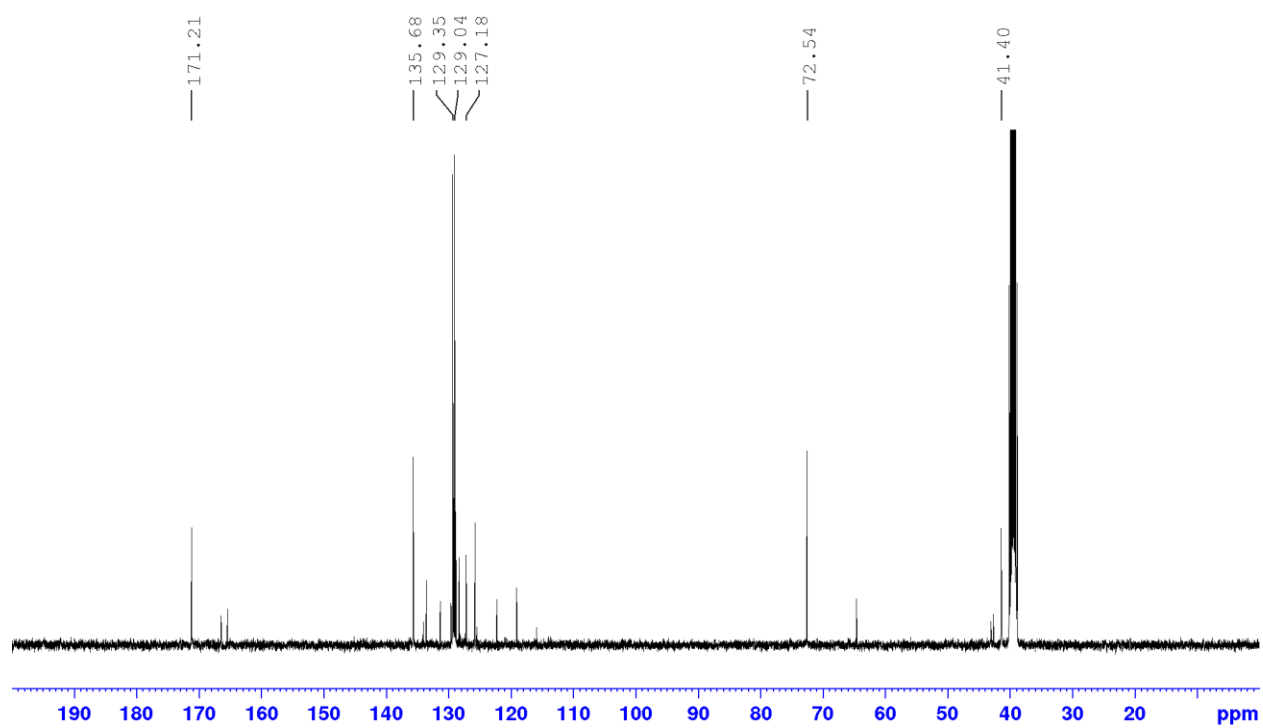

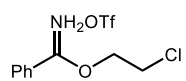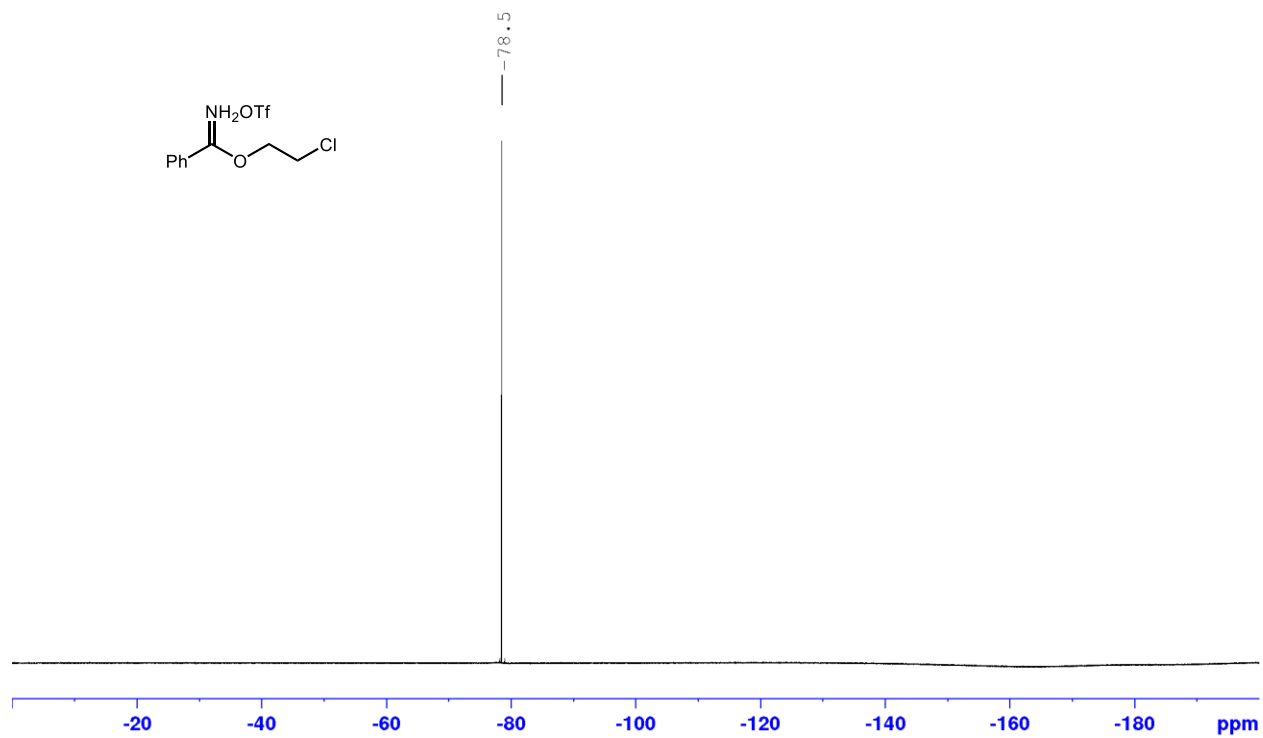

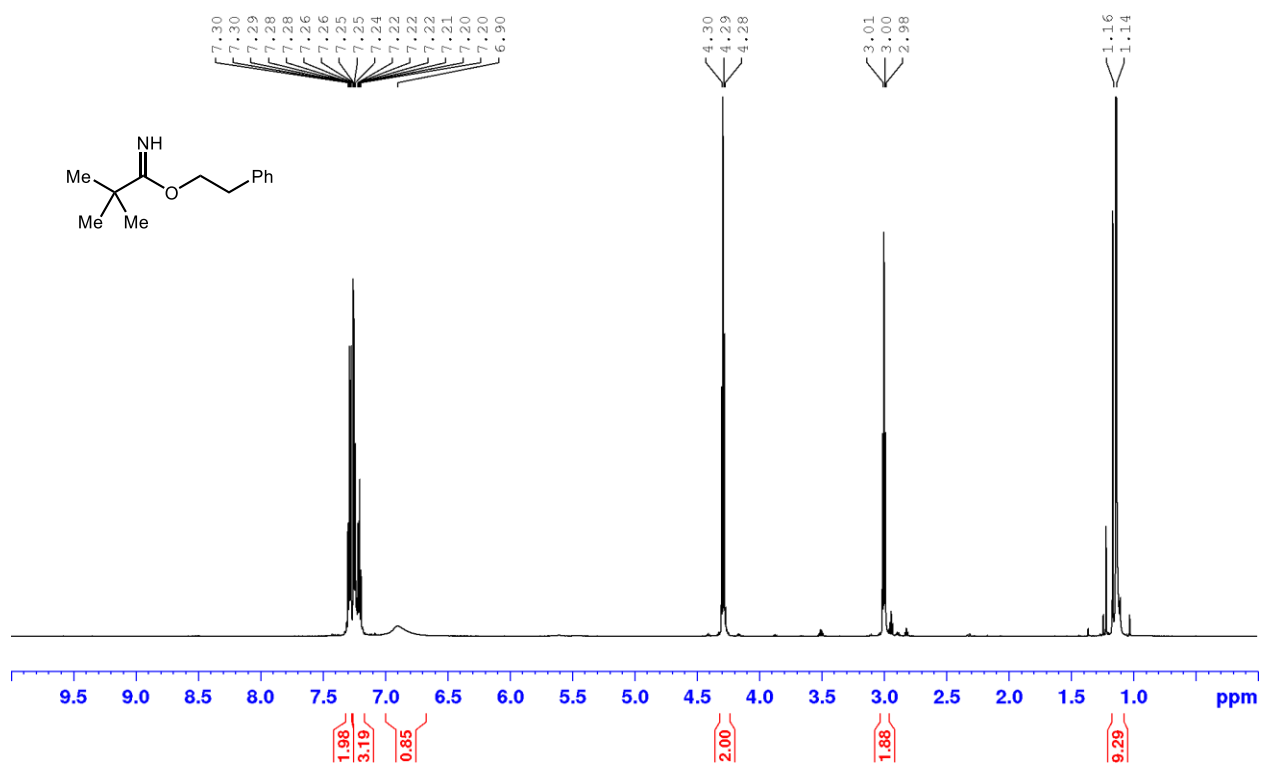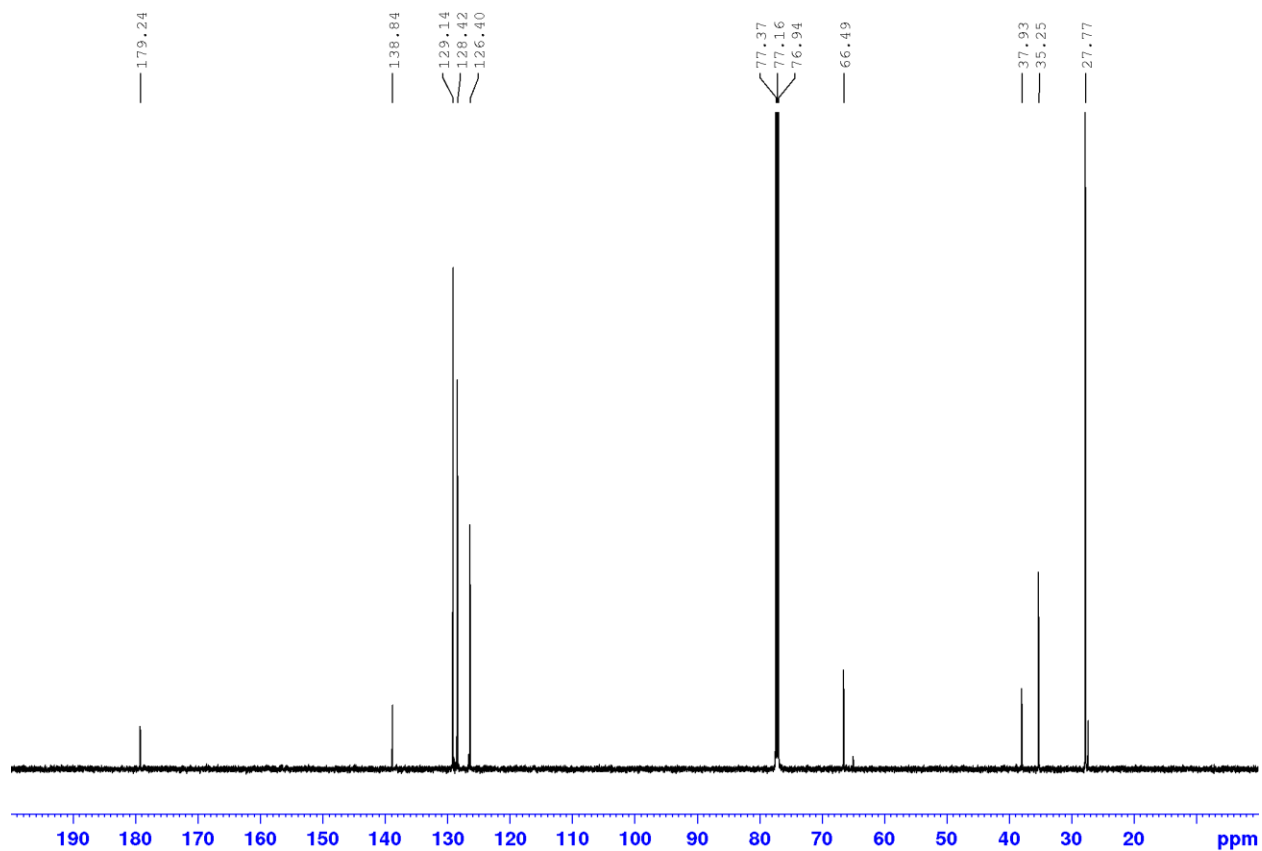

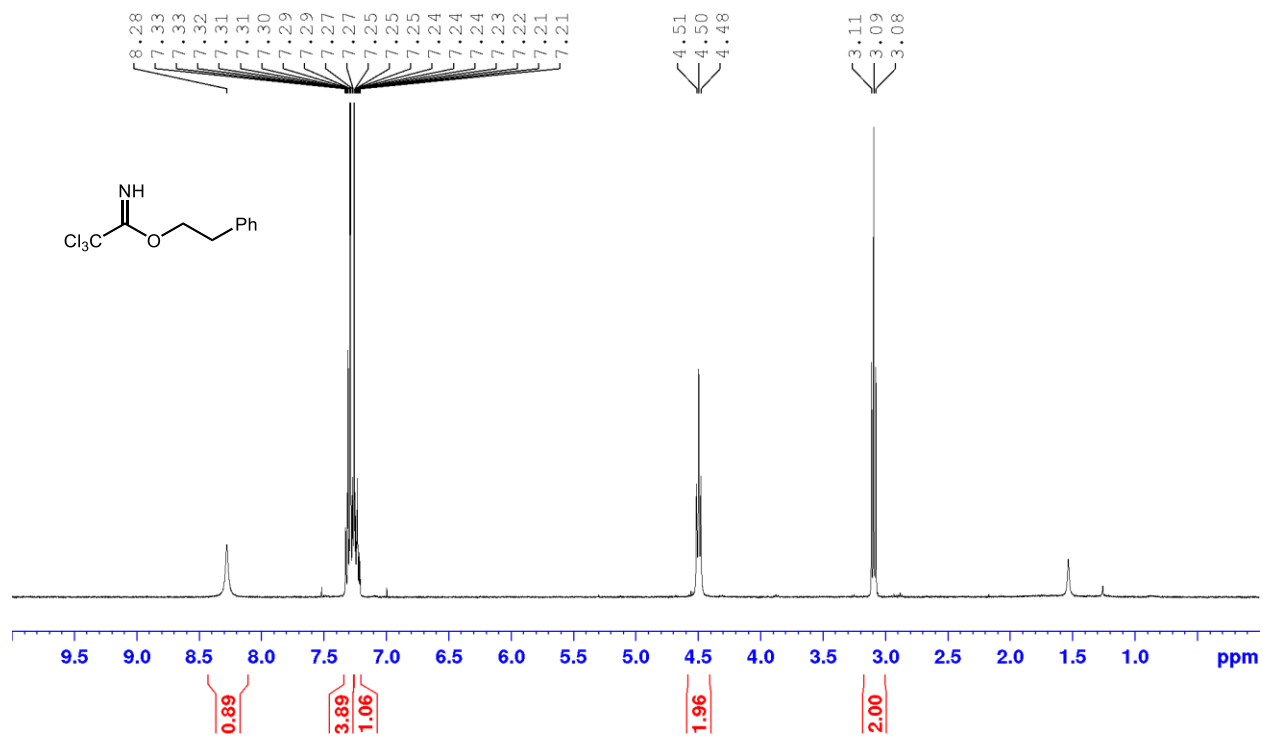

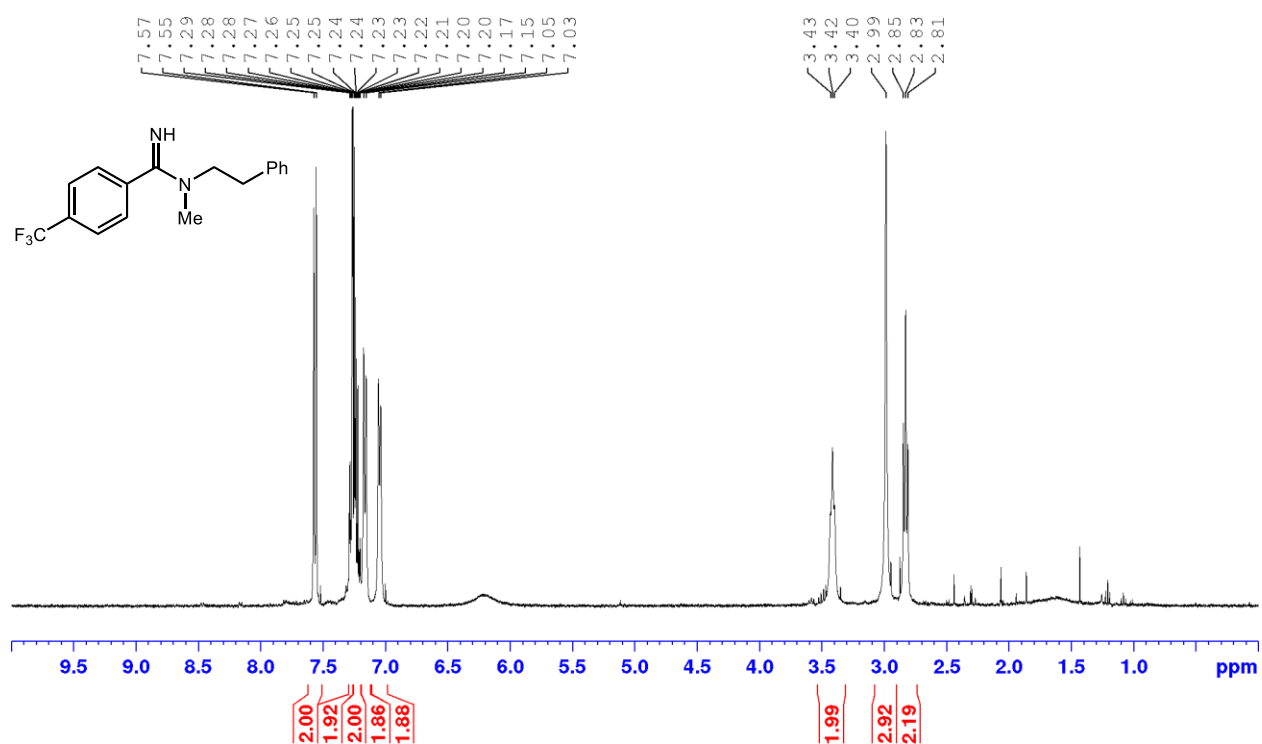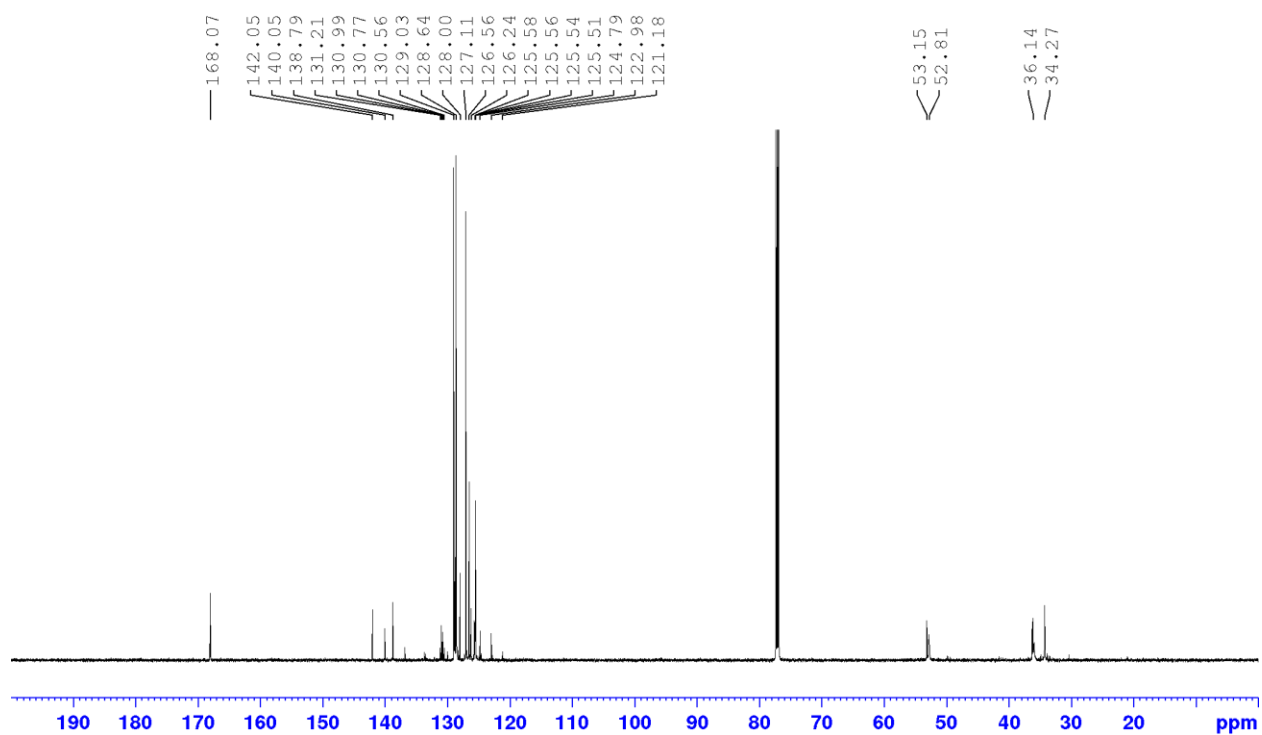

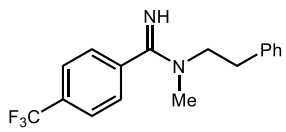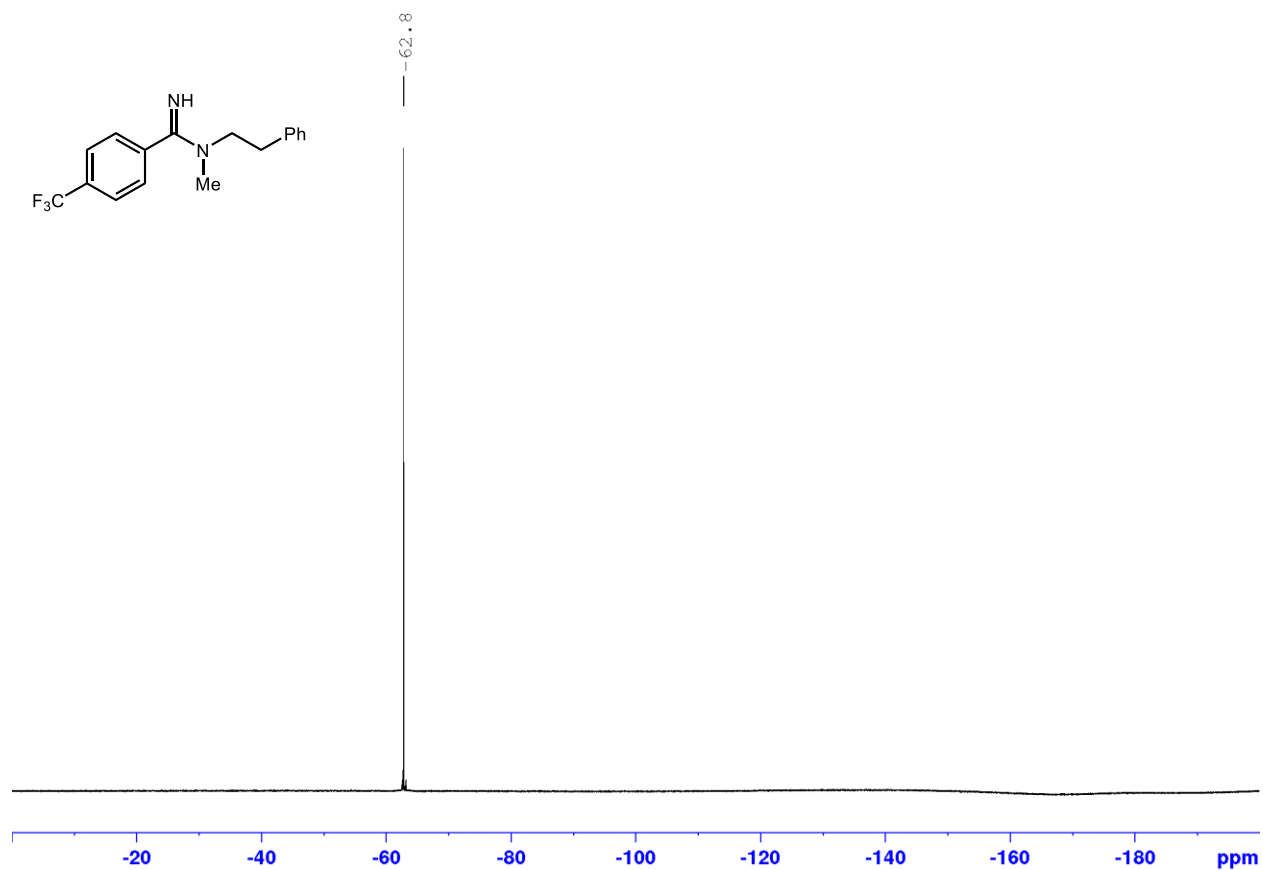

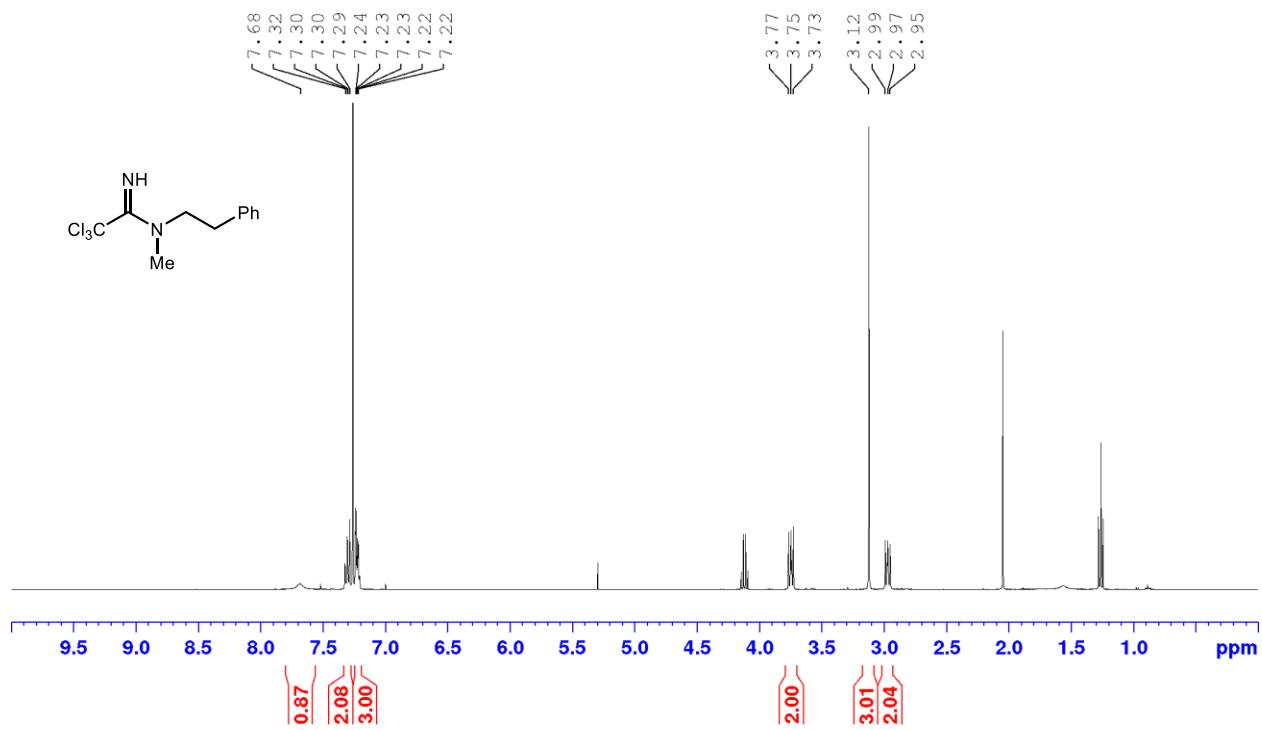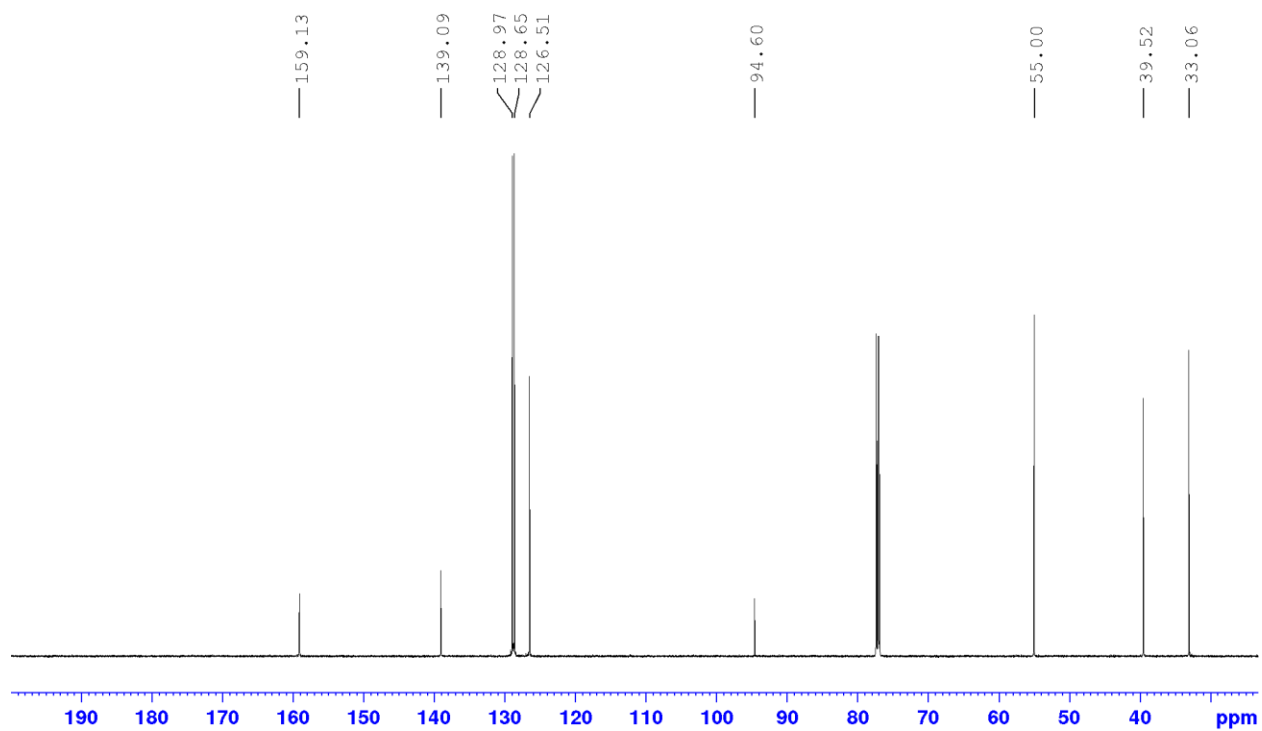

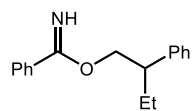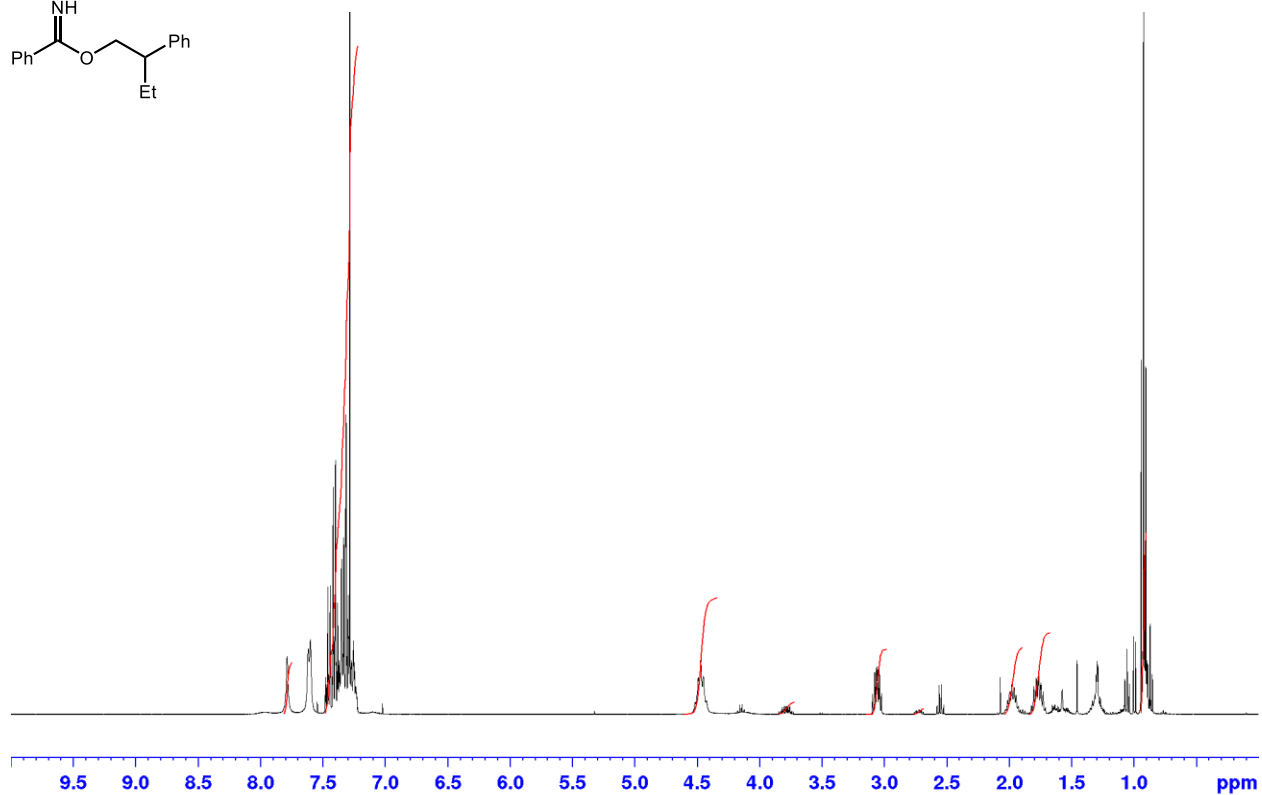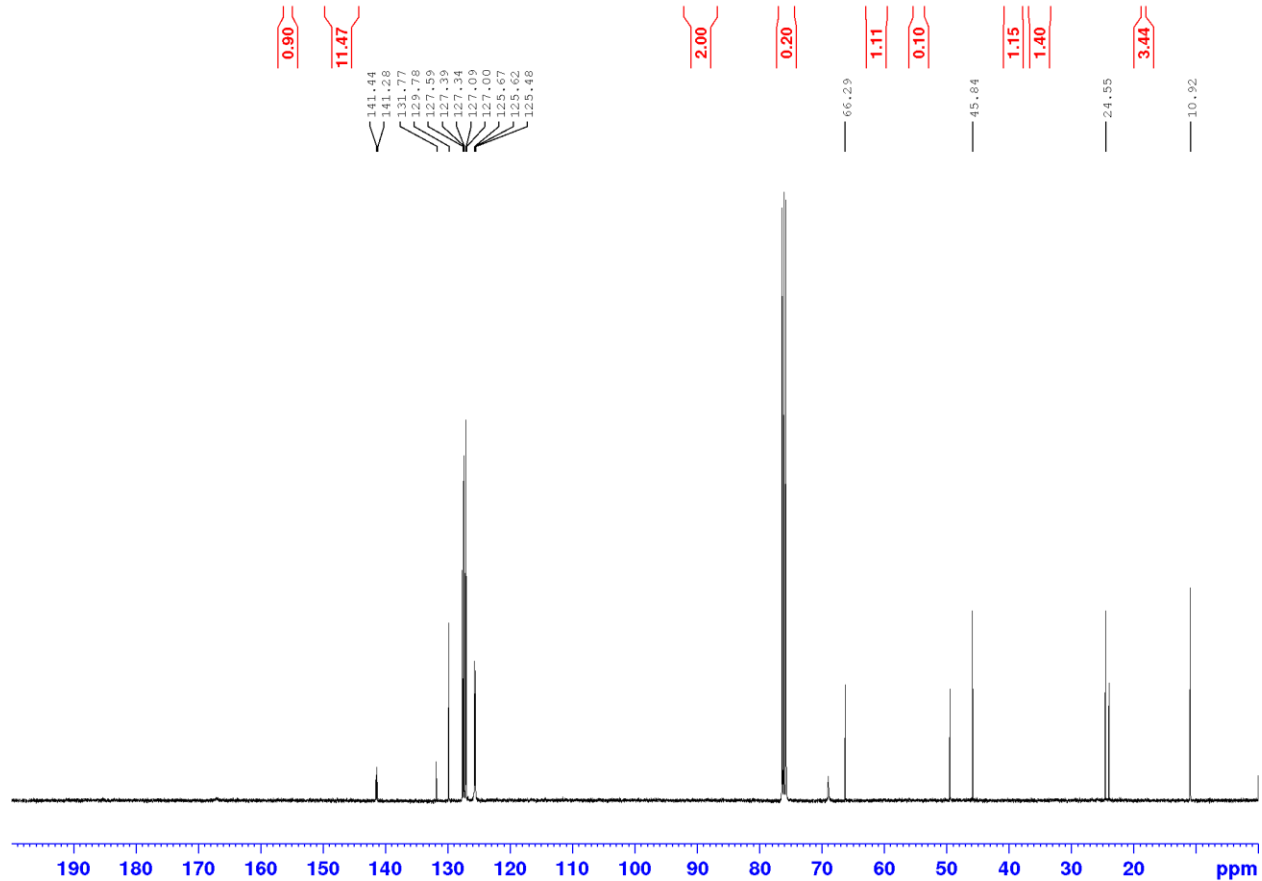

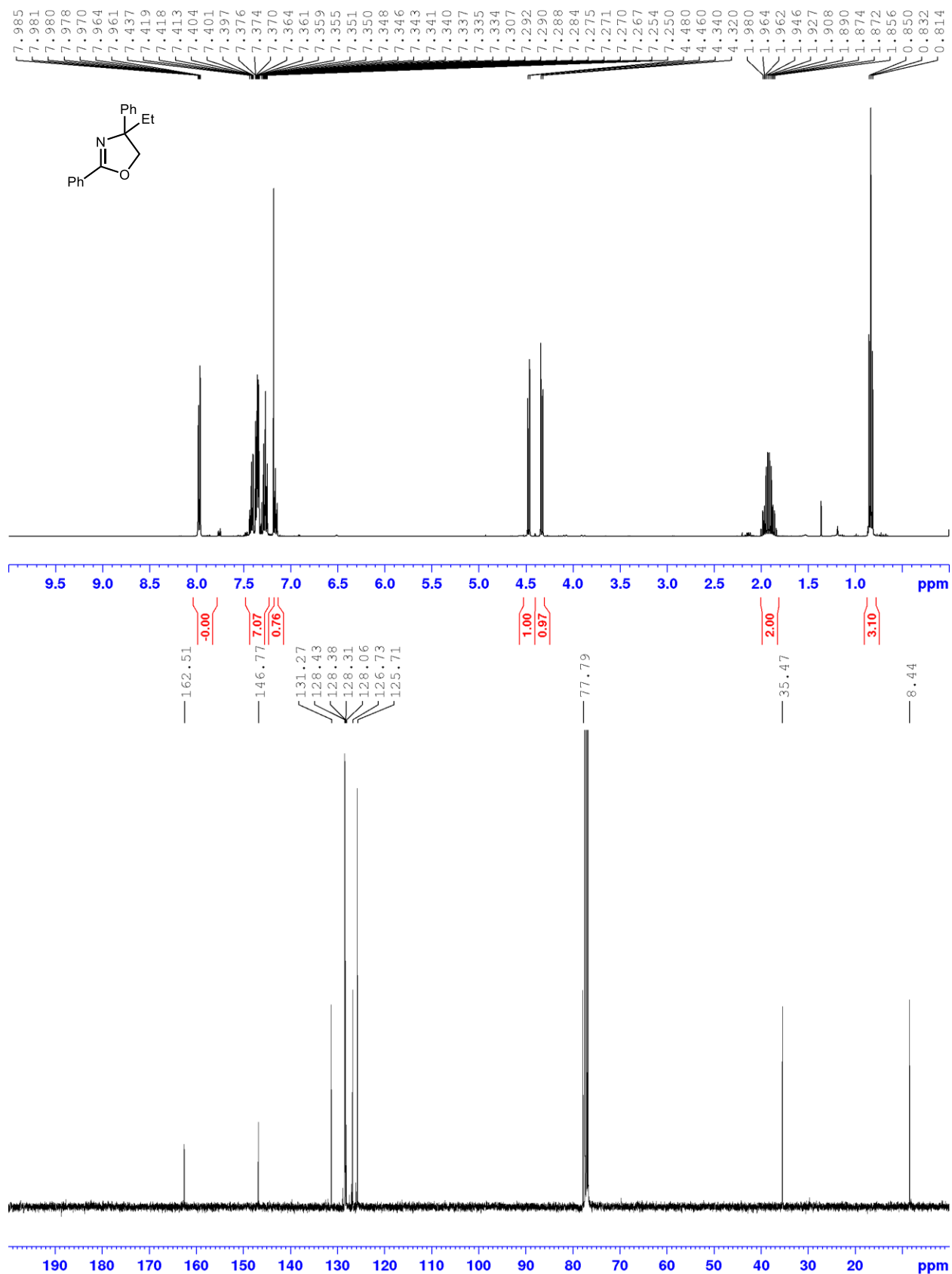

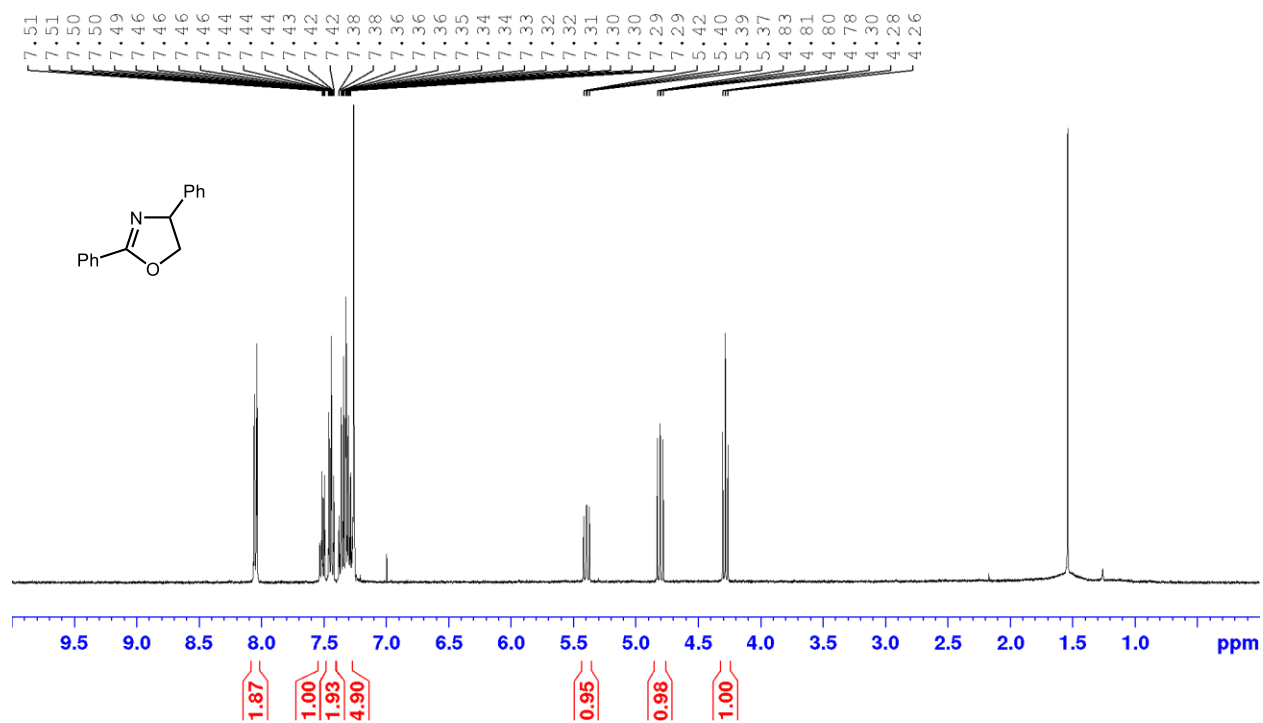

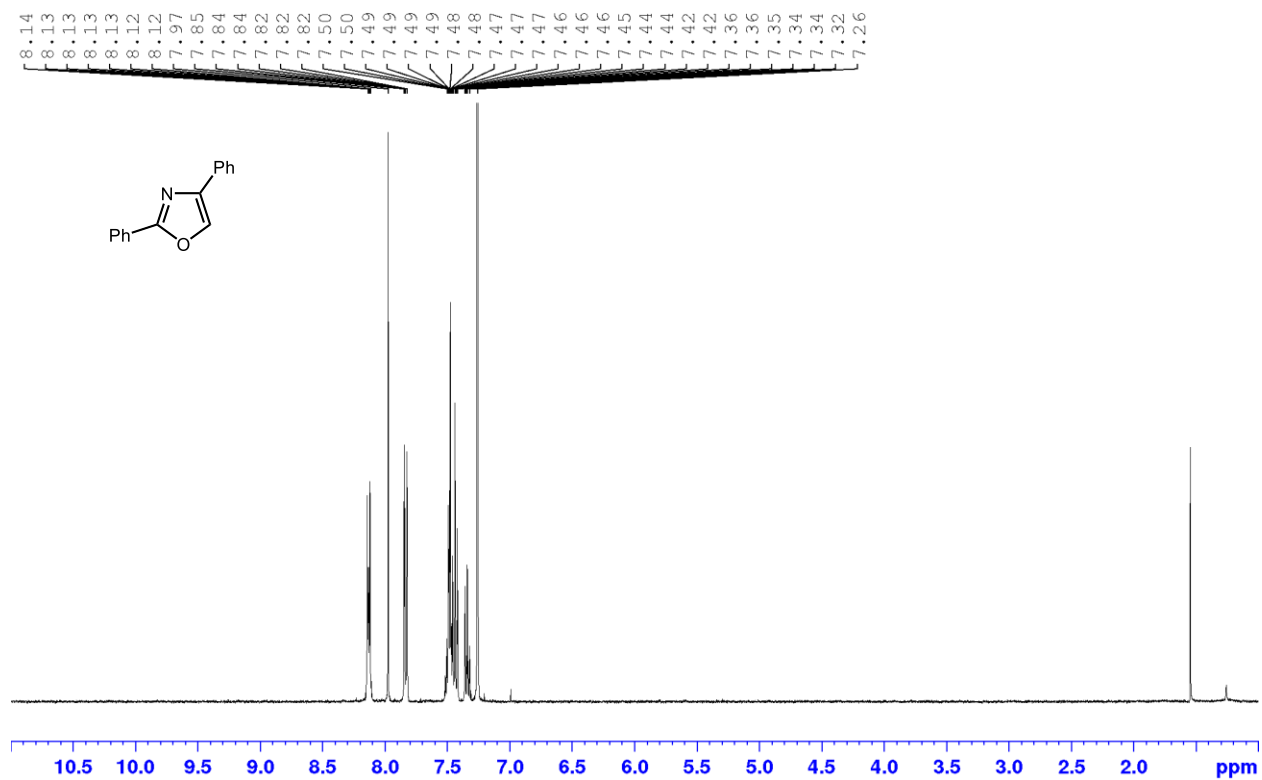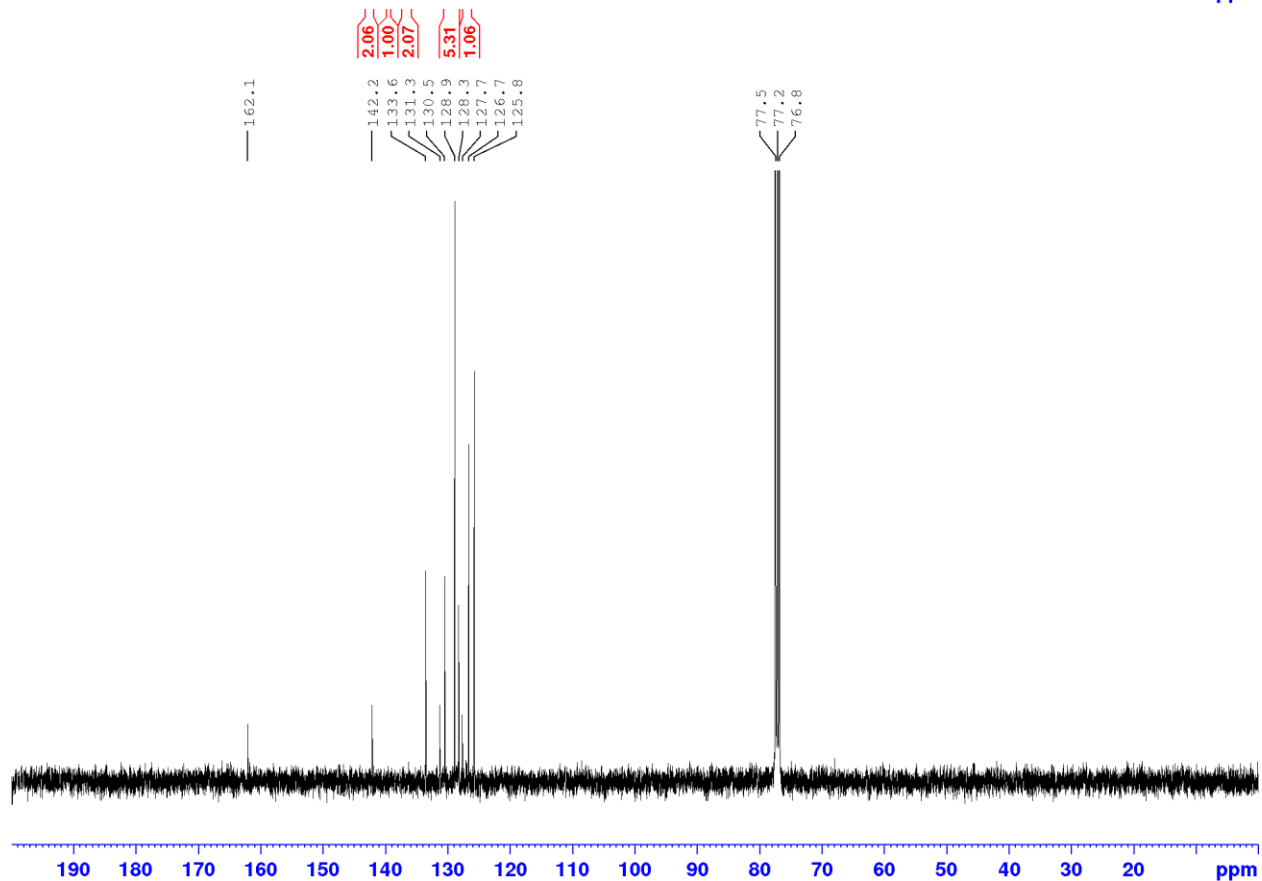

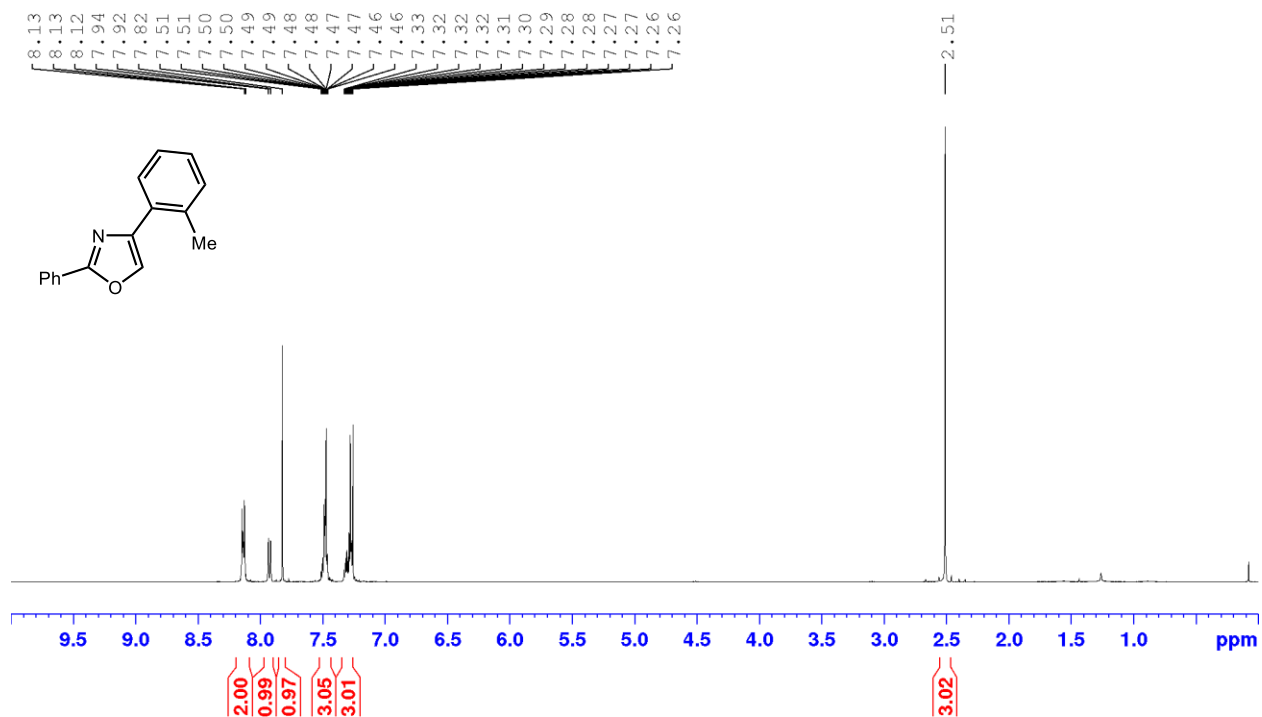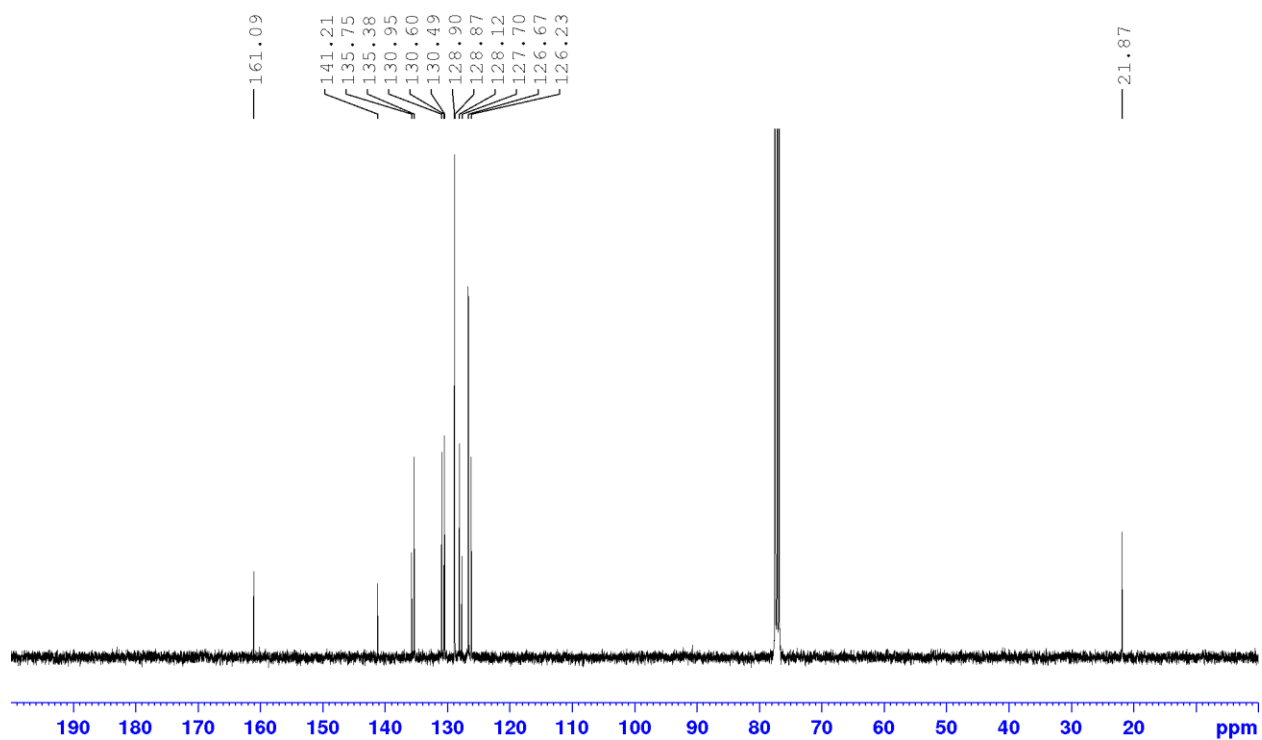

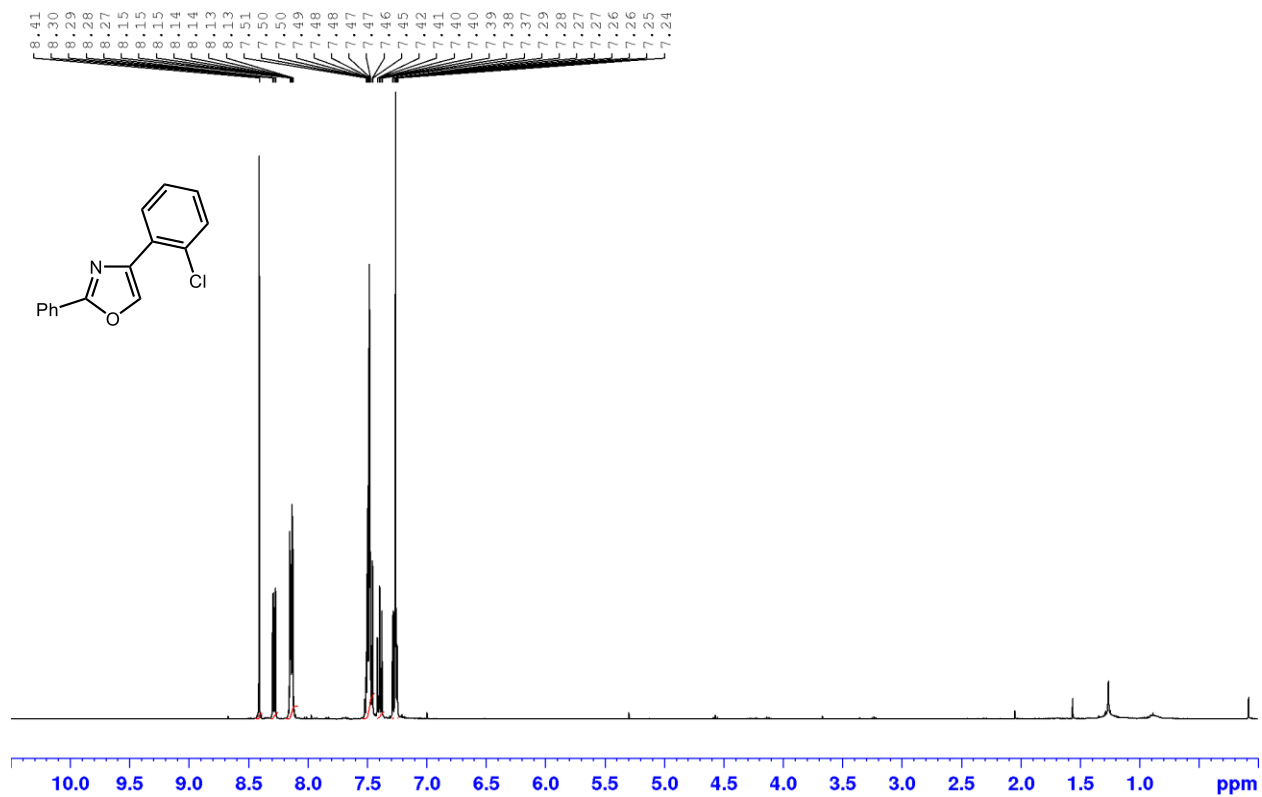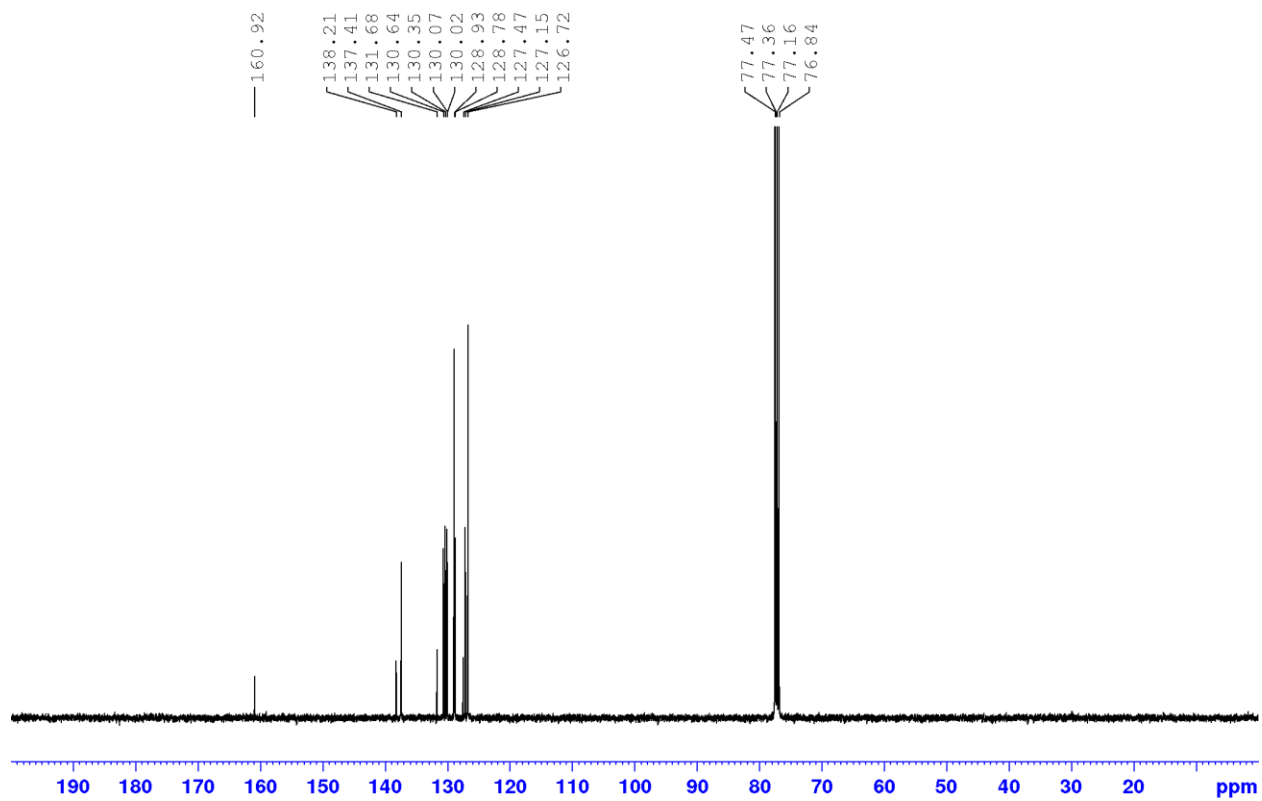

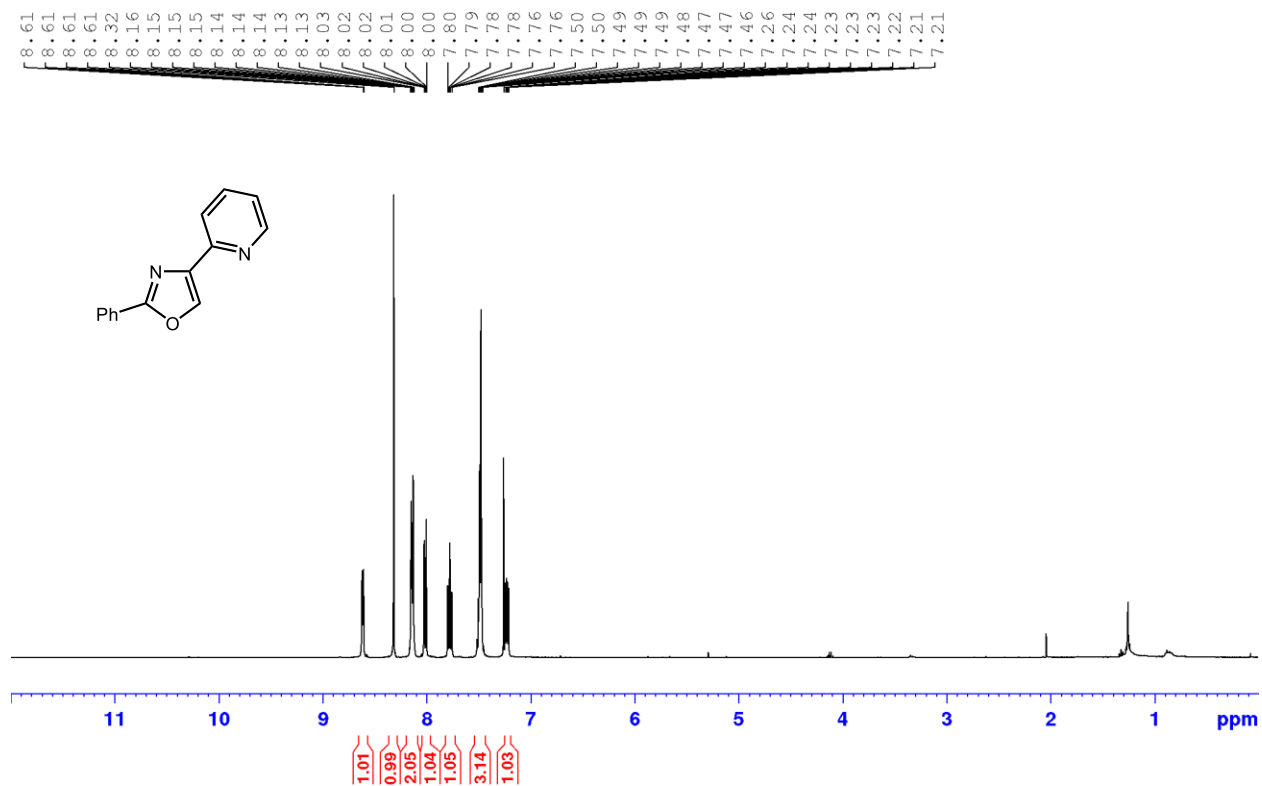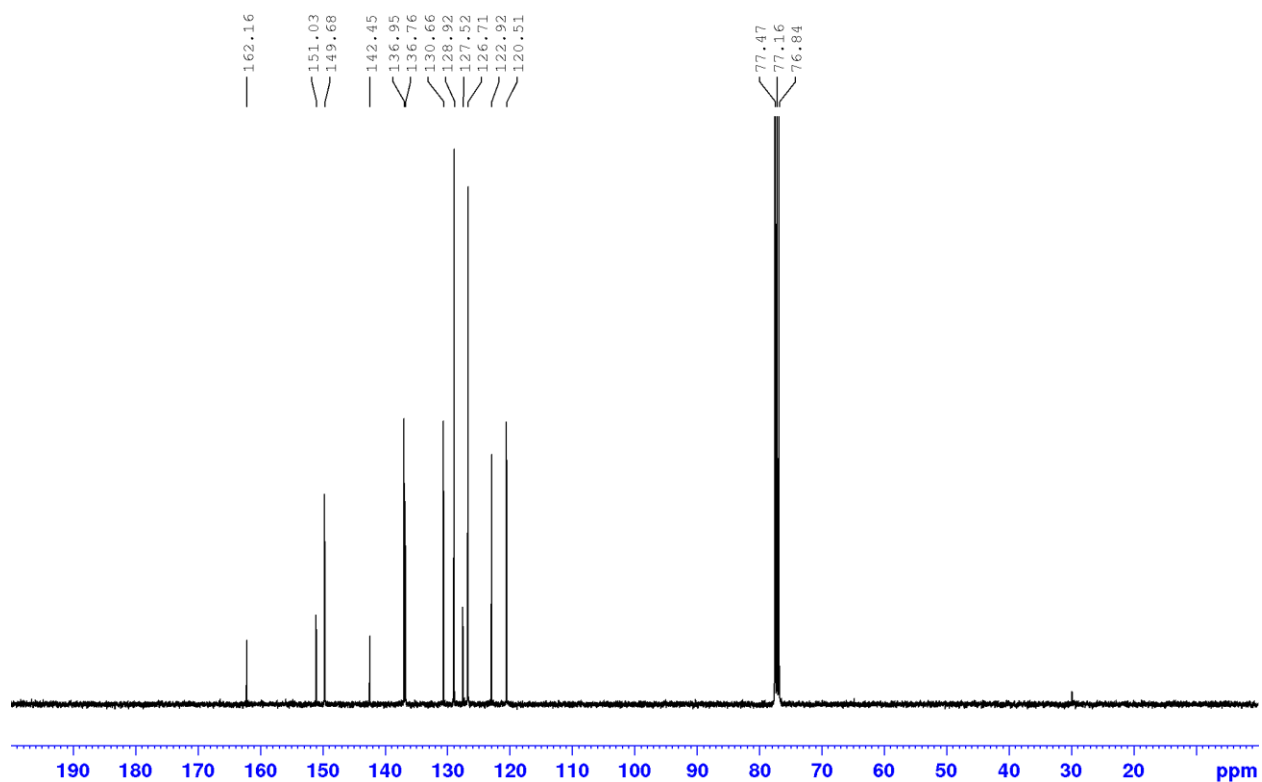

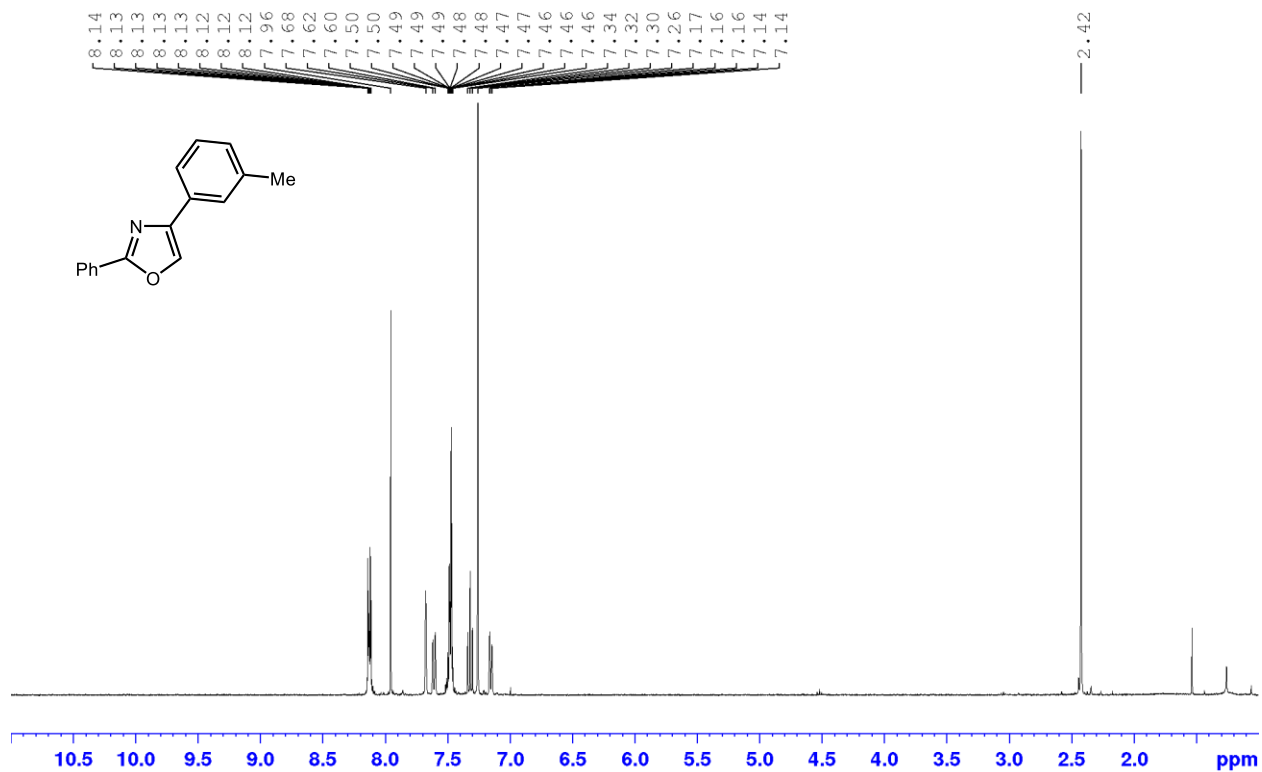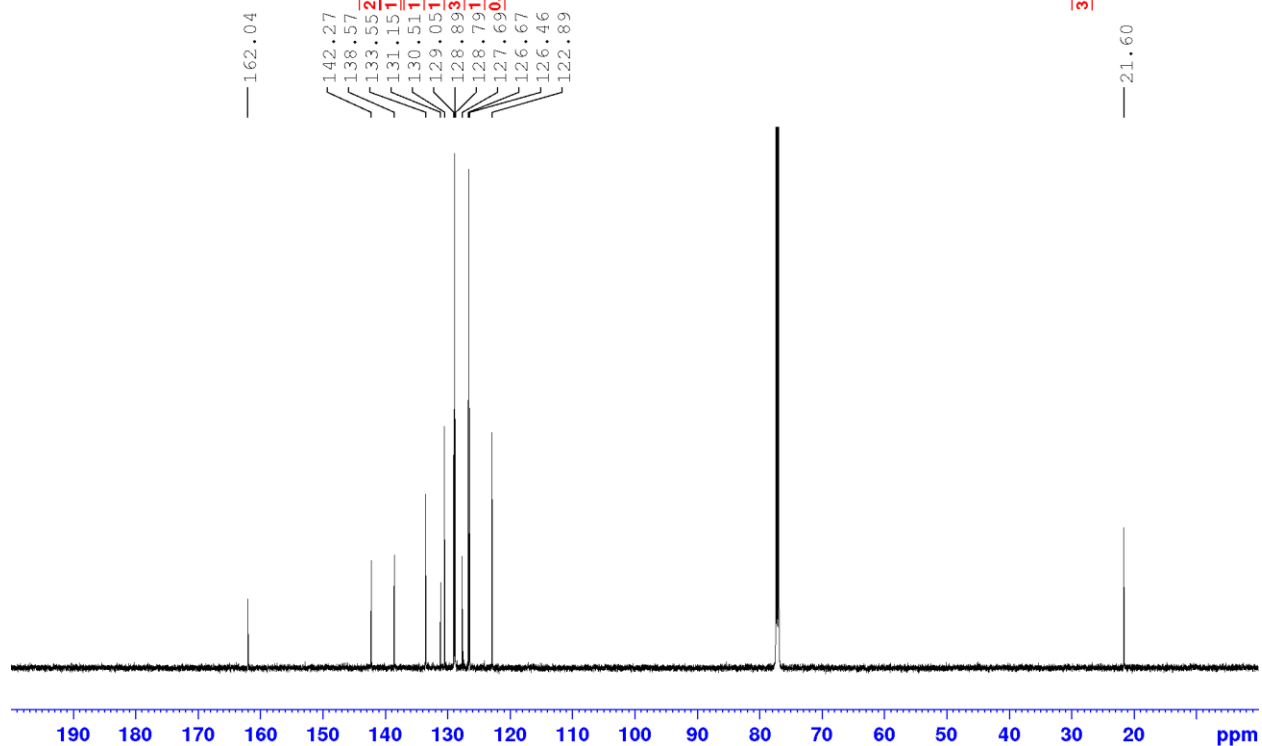

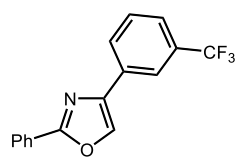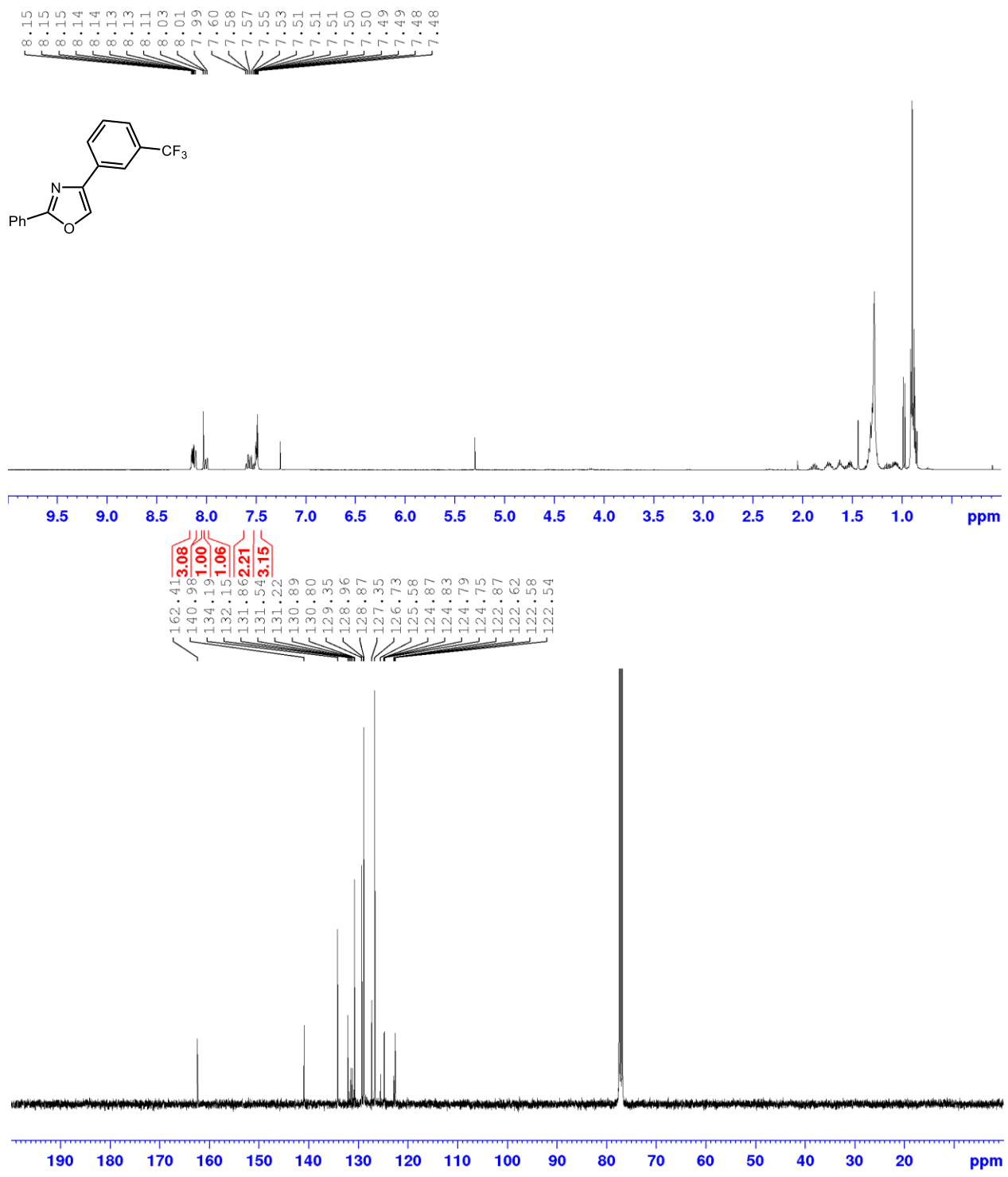

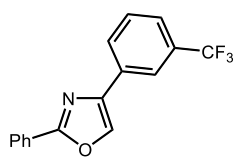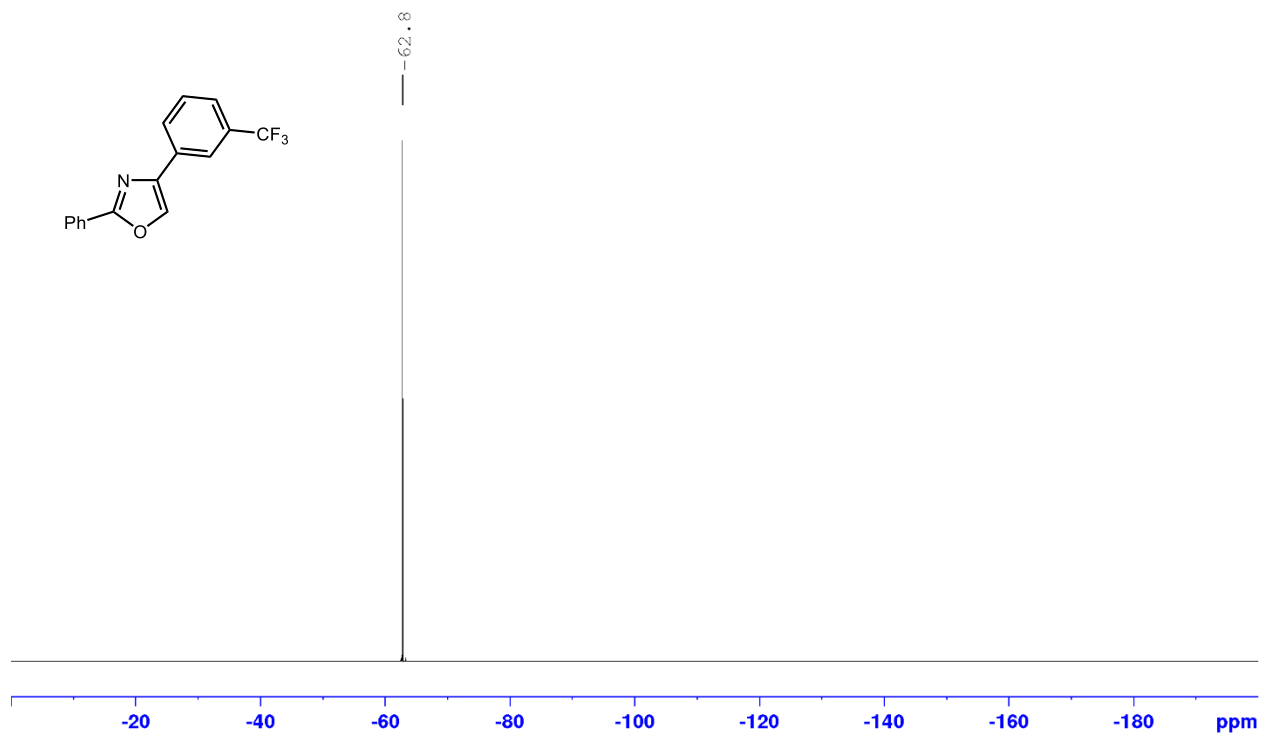

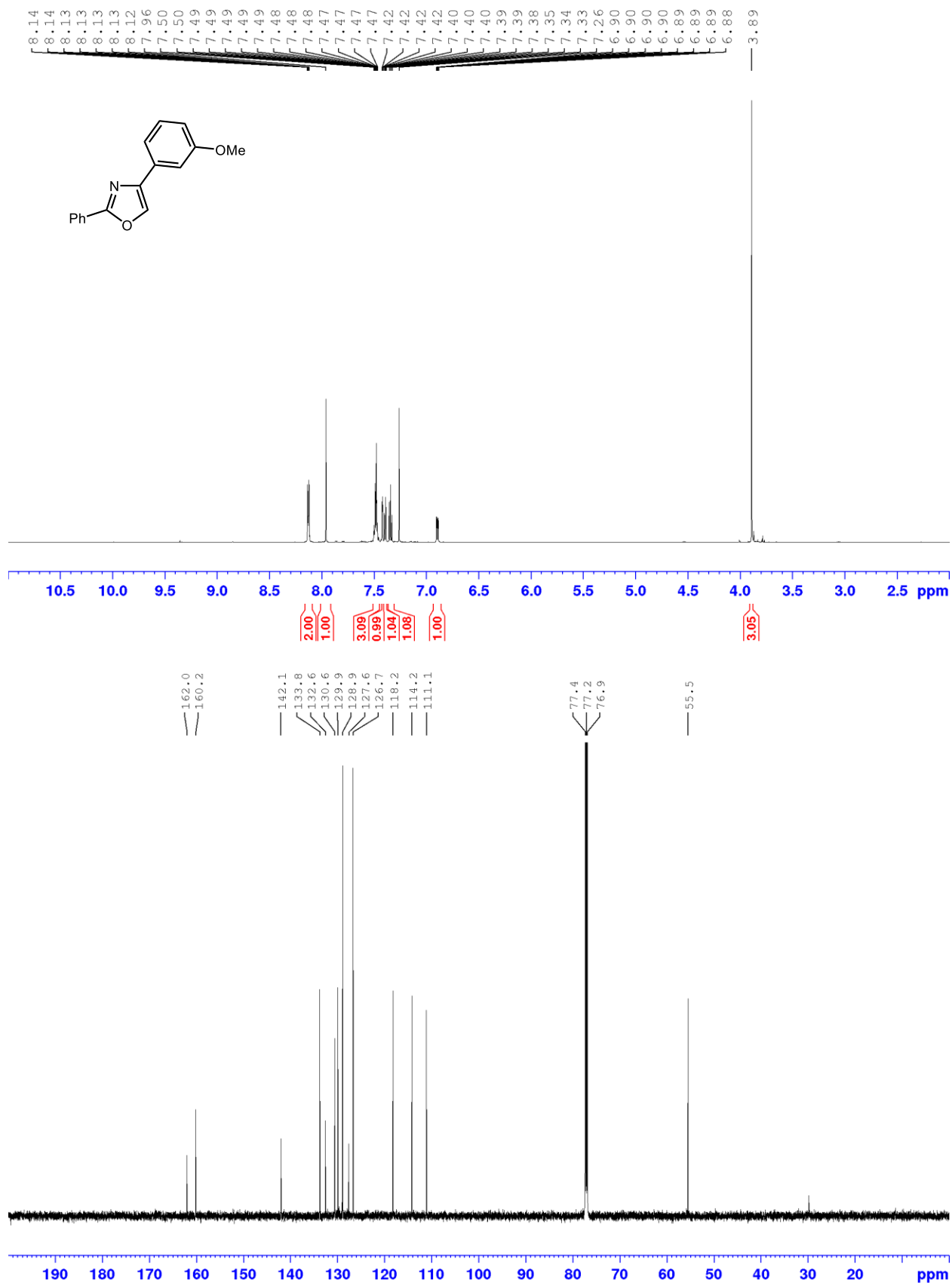

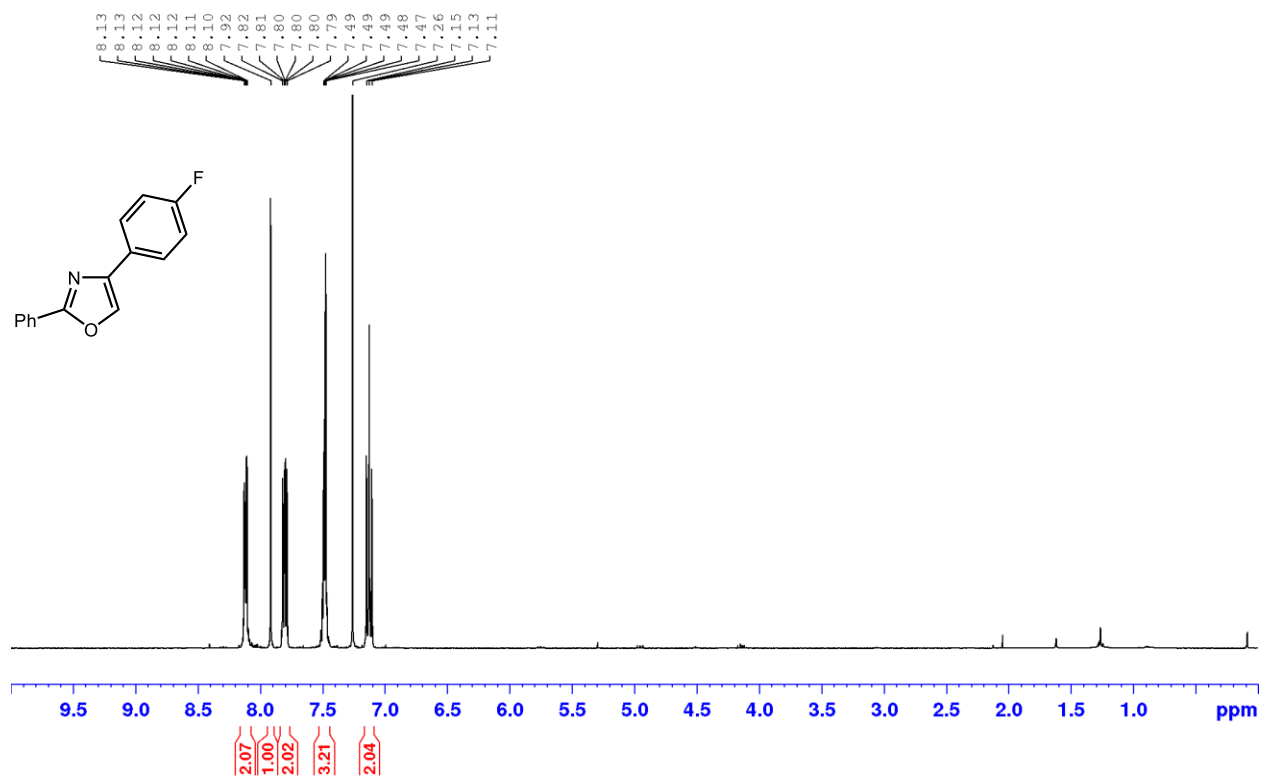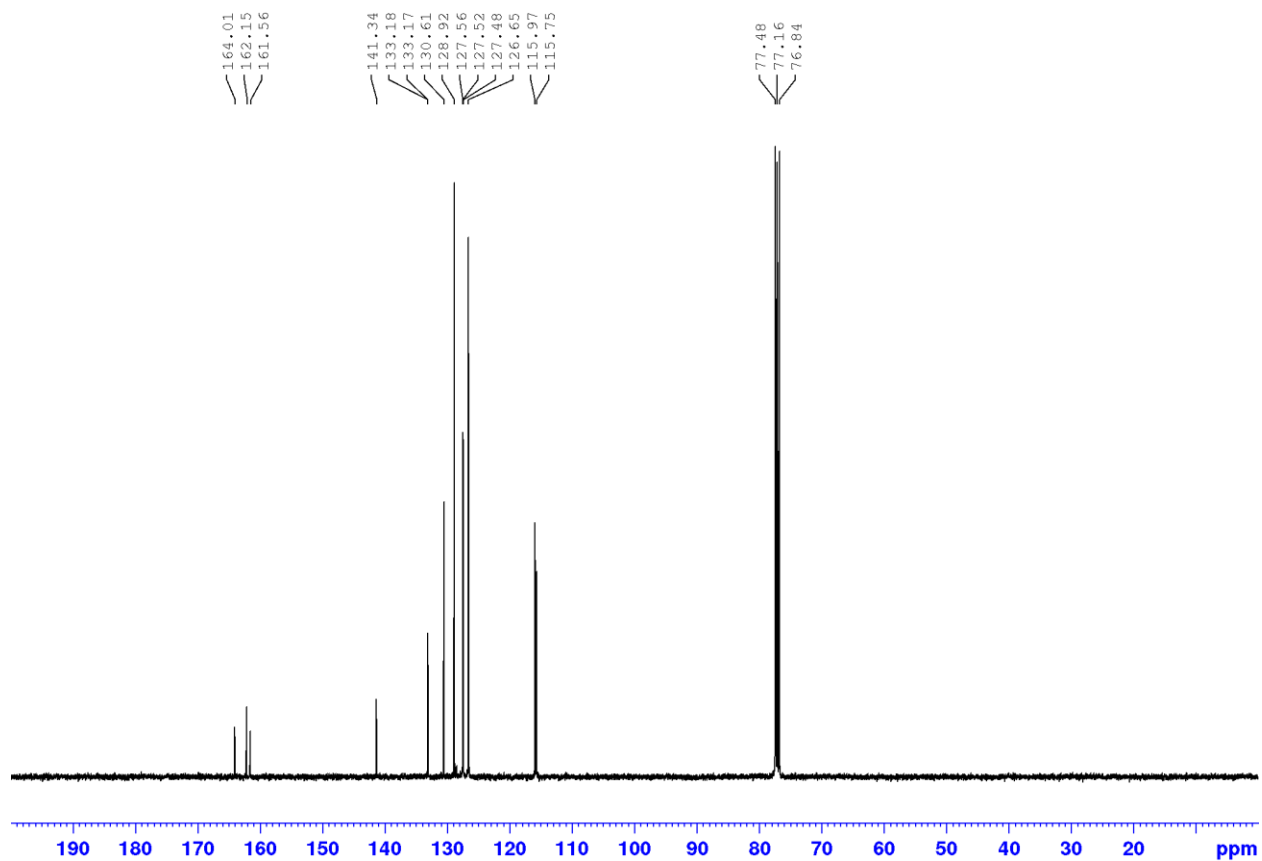

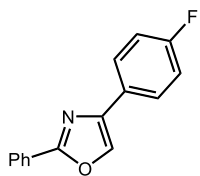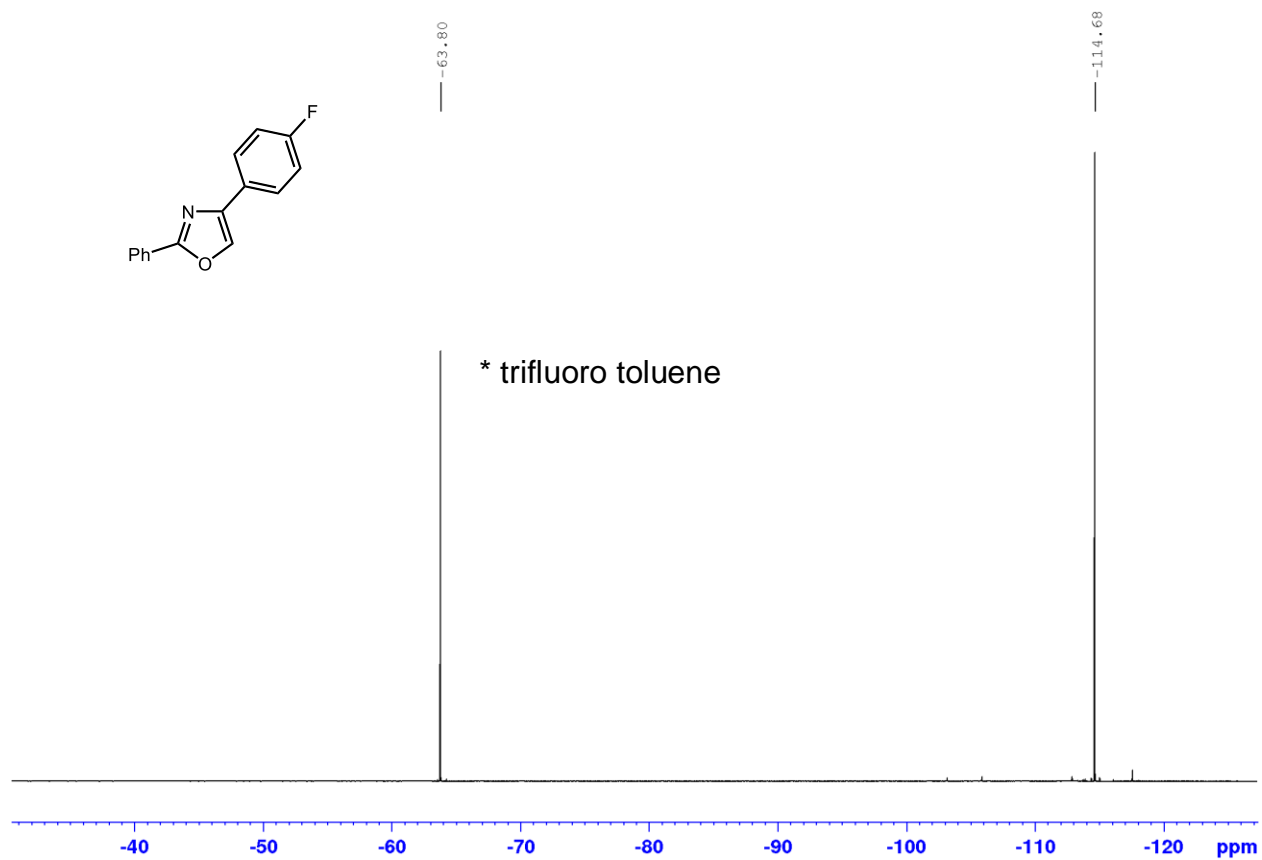

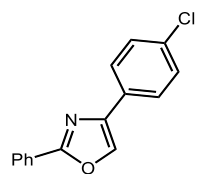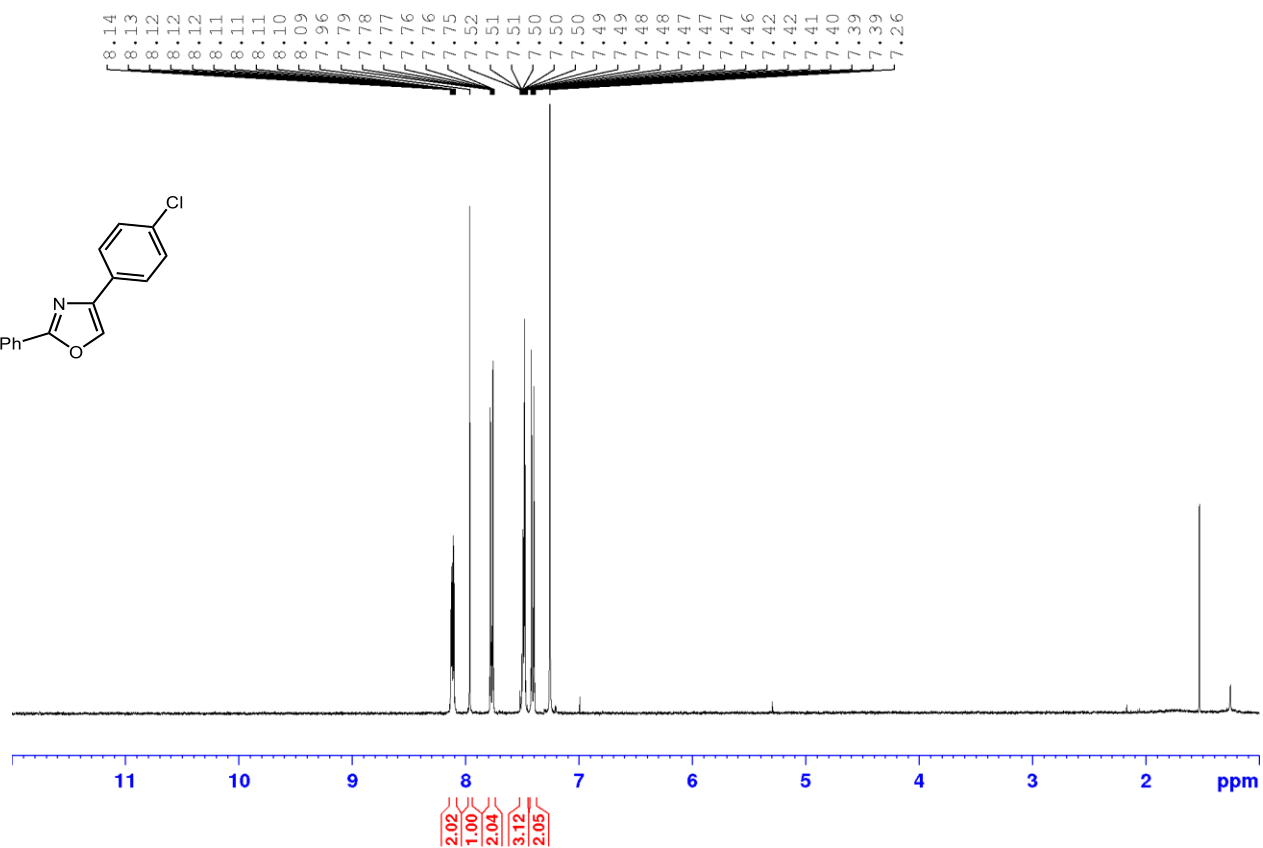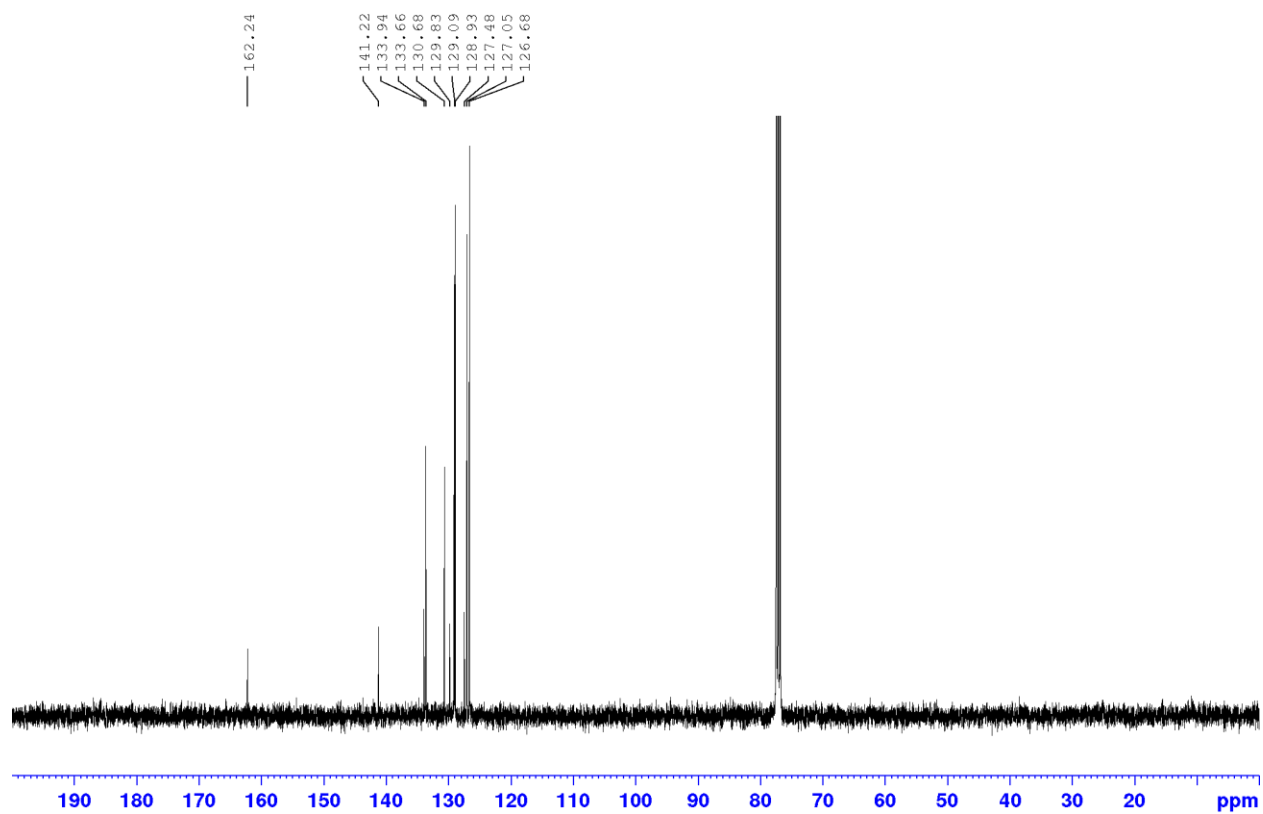

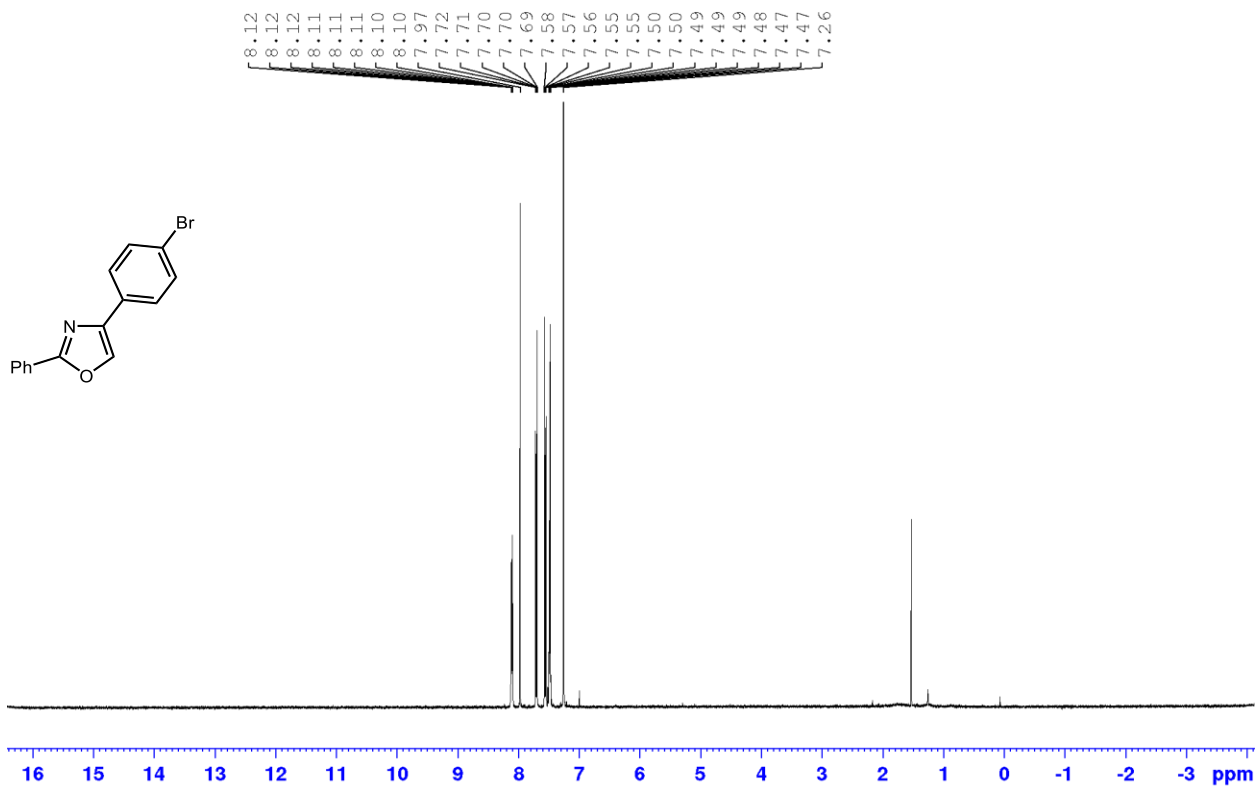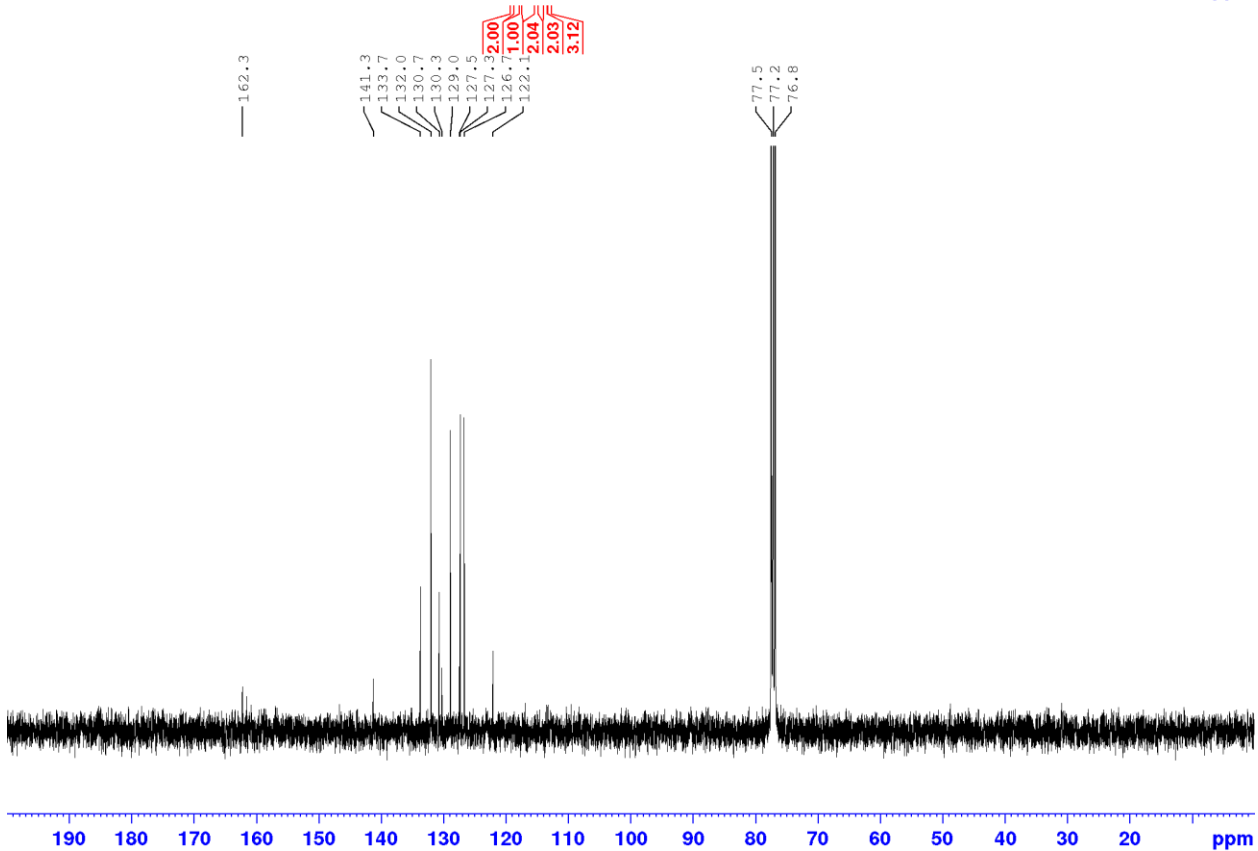

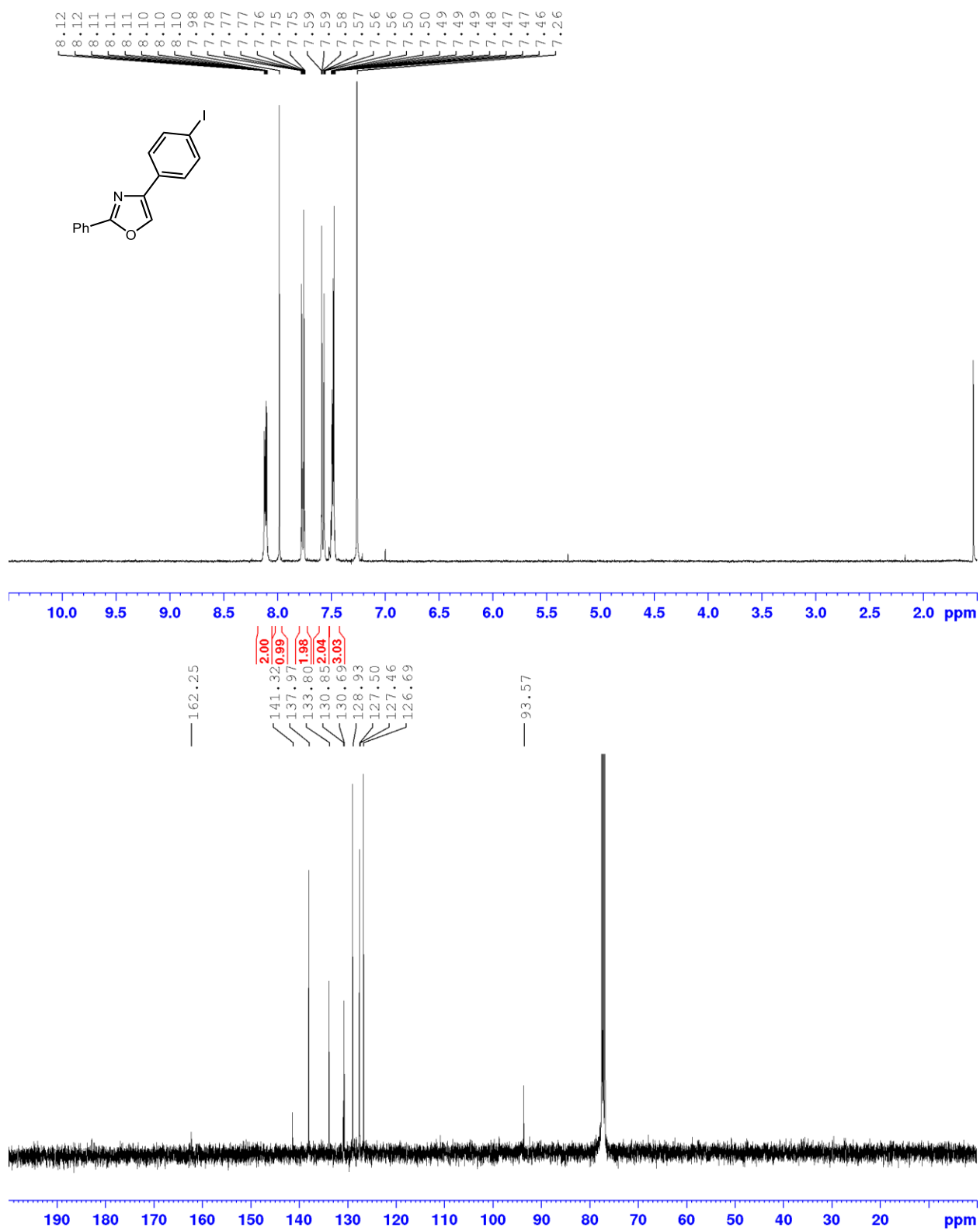

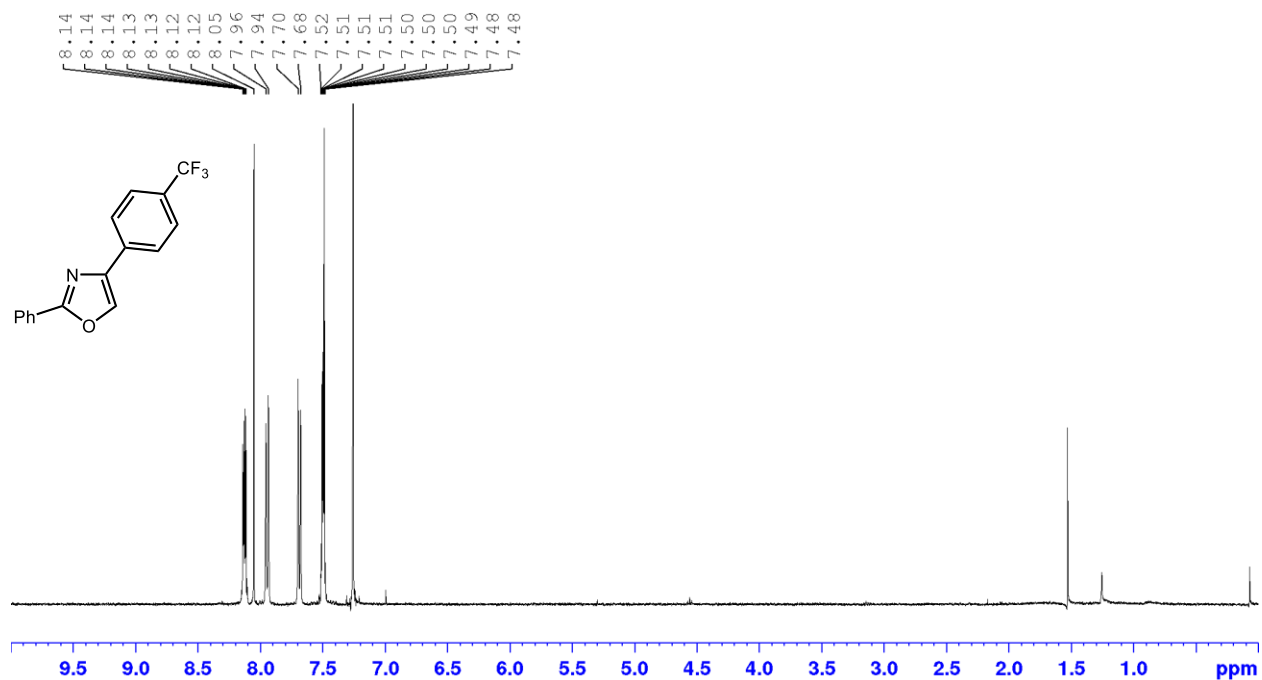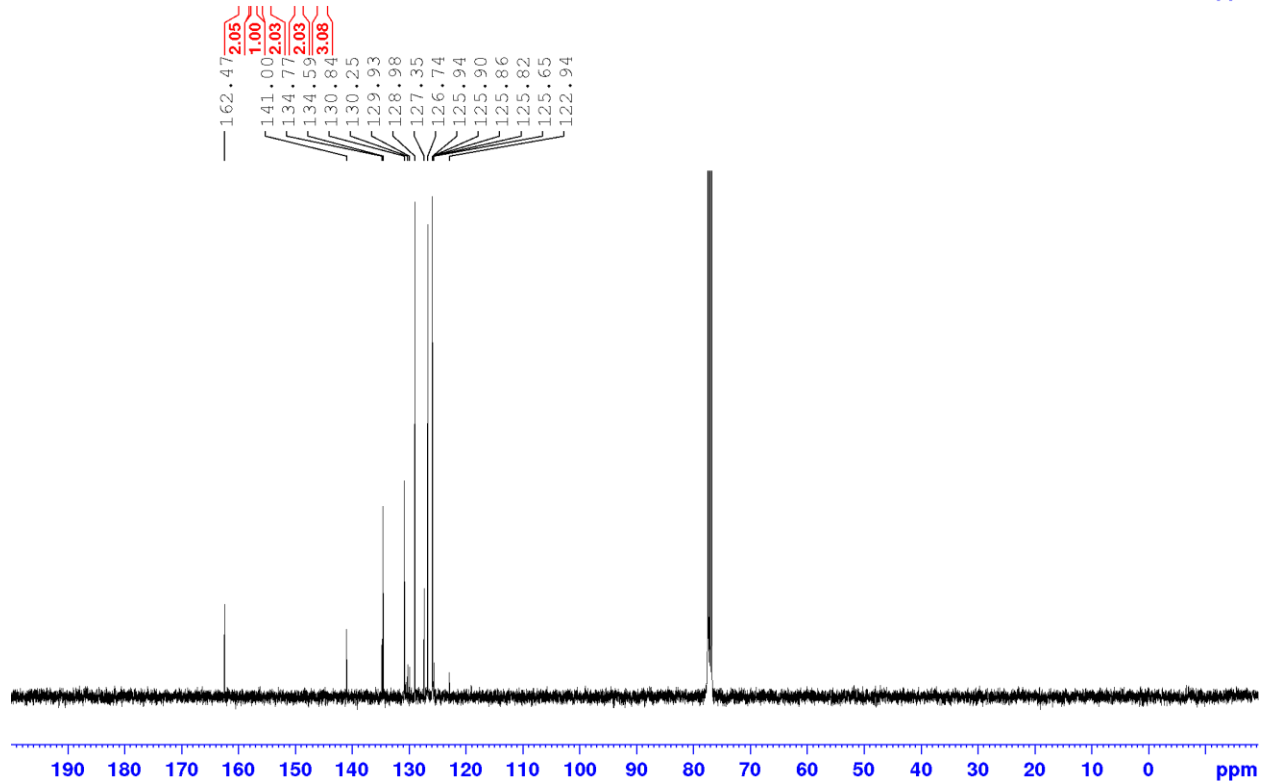

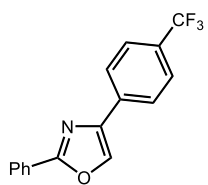

— -62.56

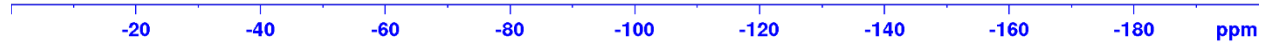

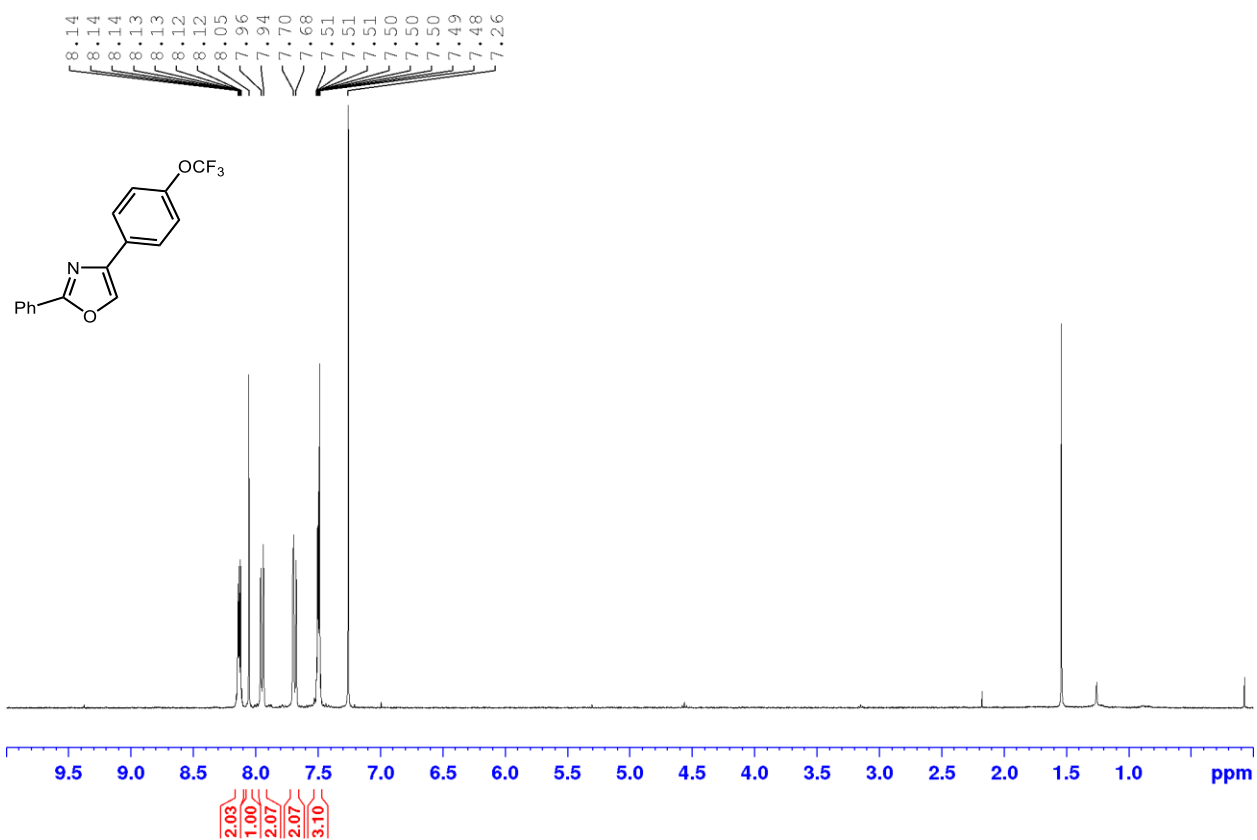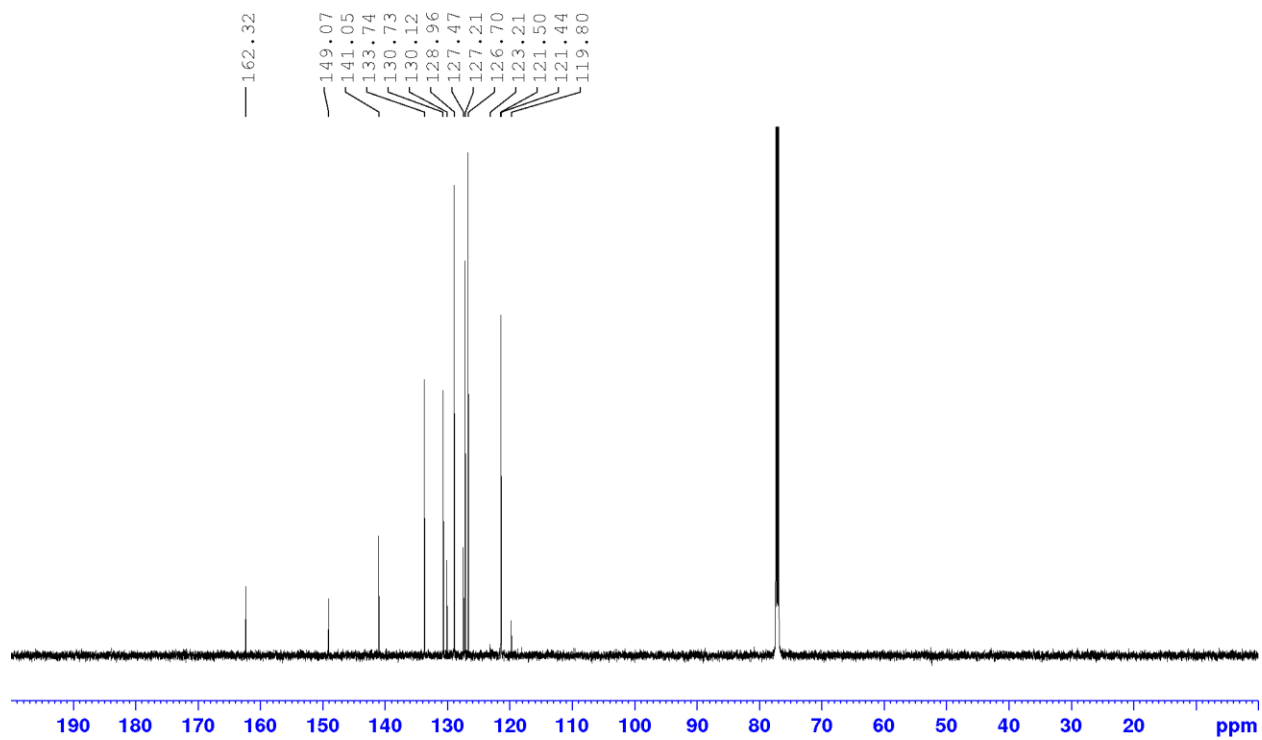

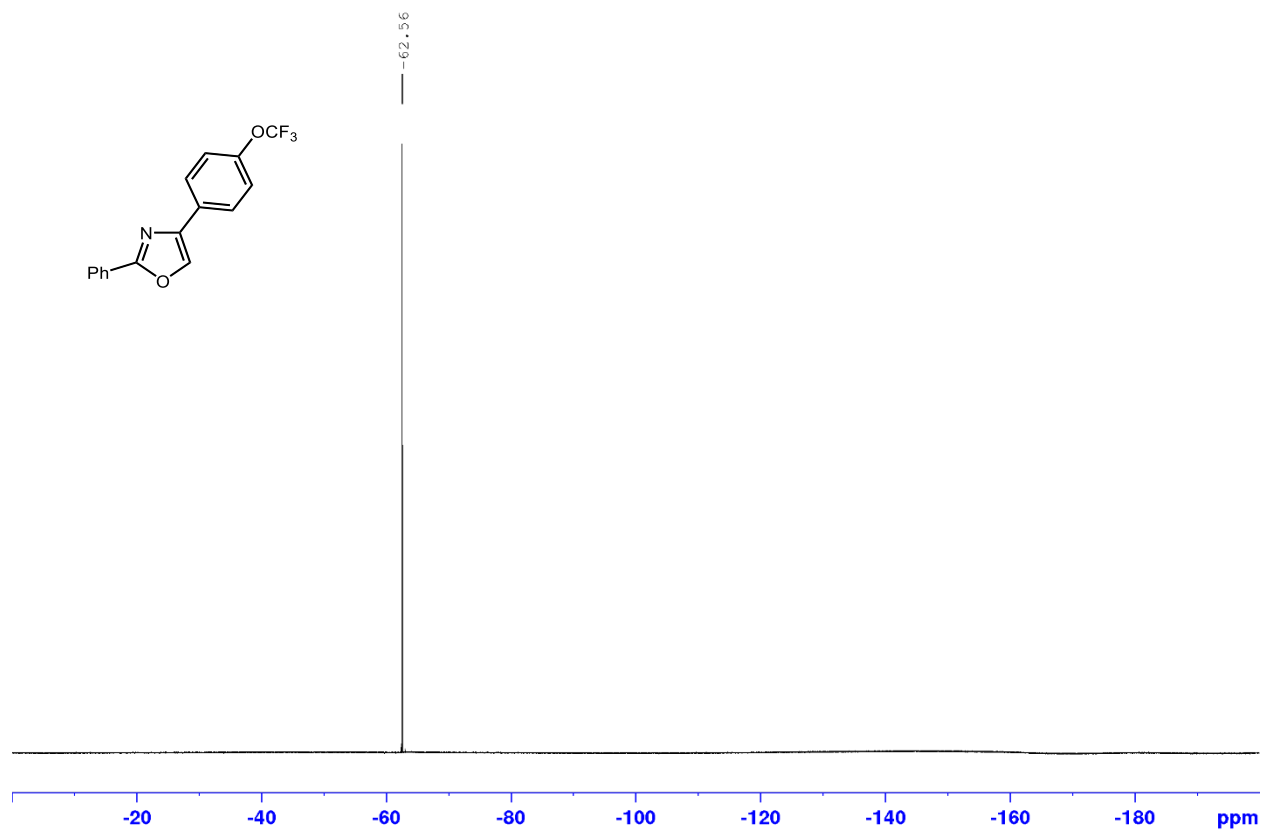

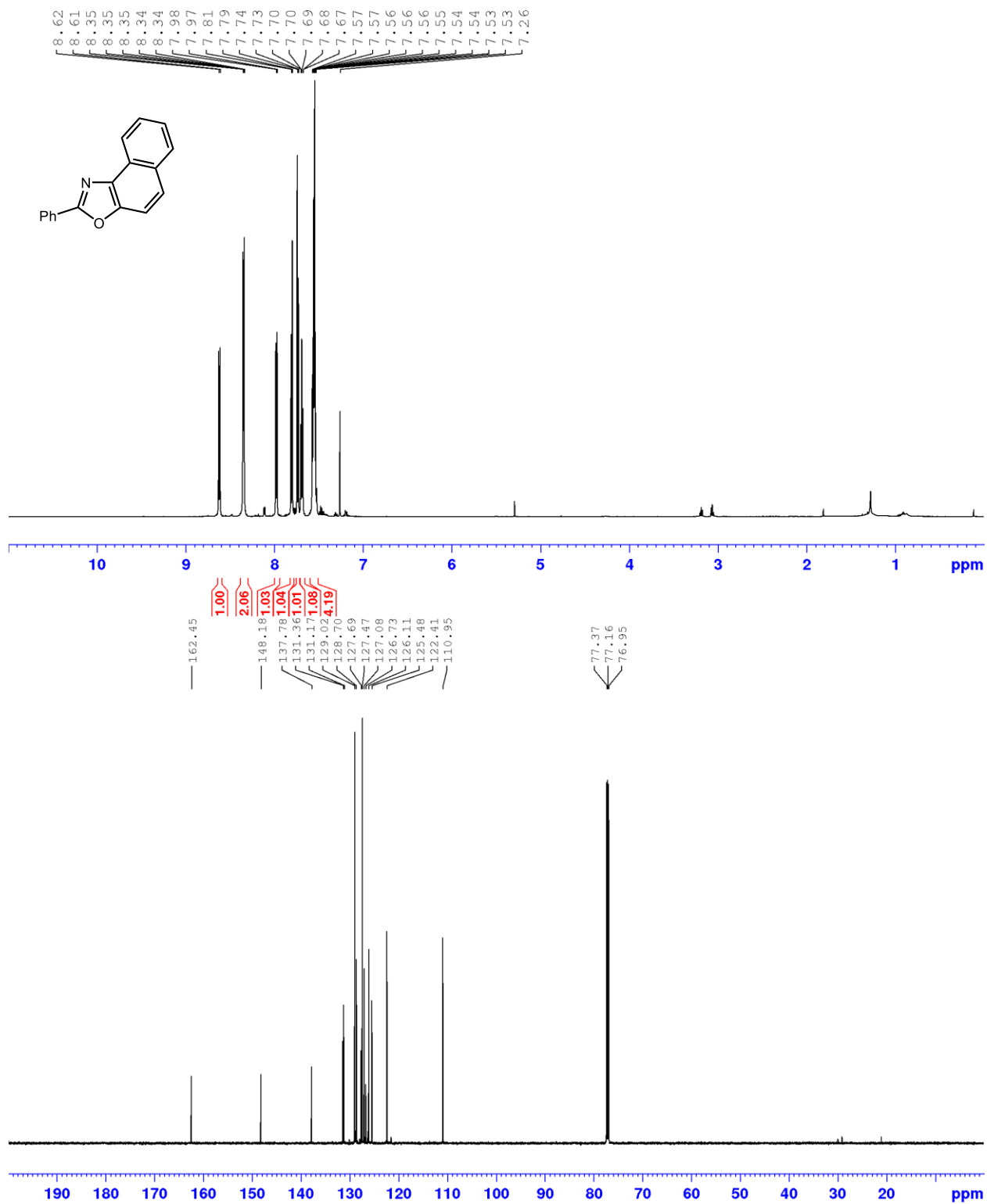

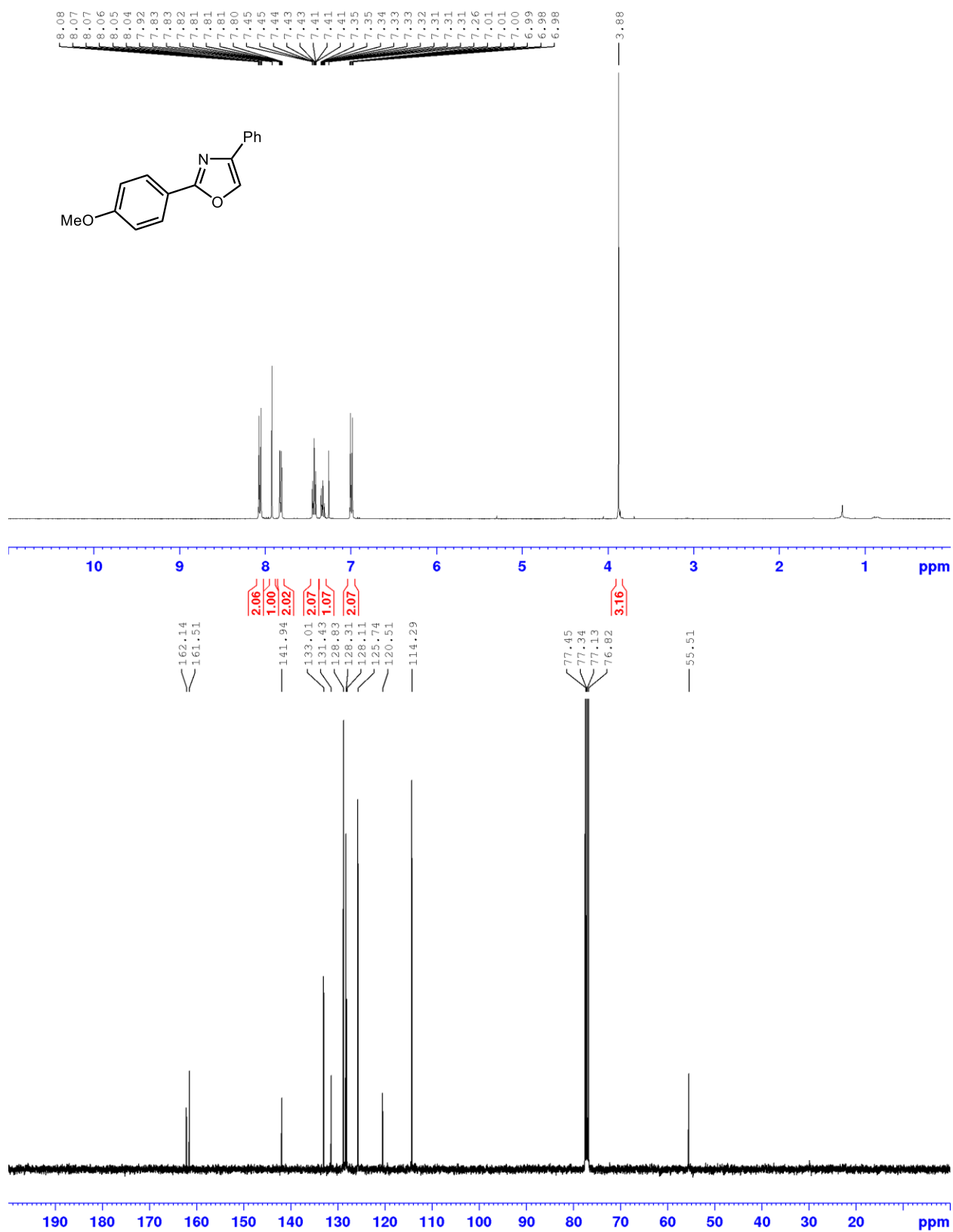

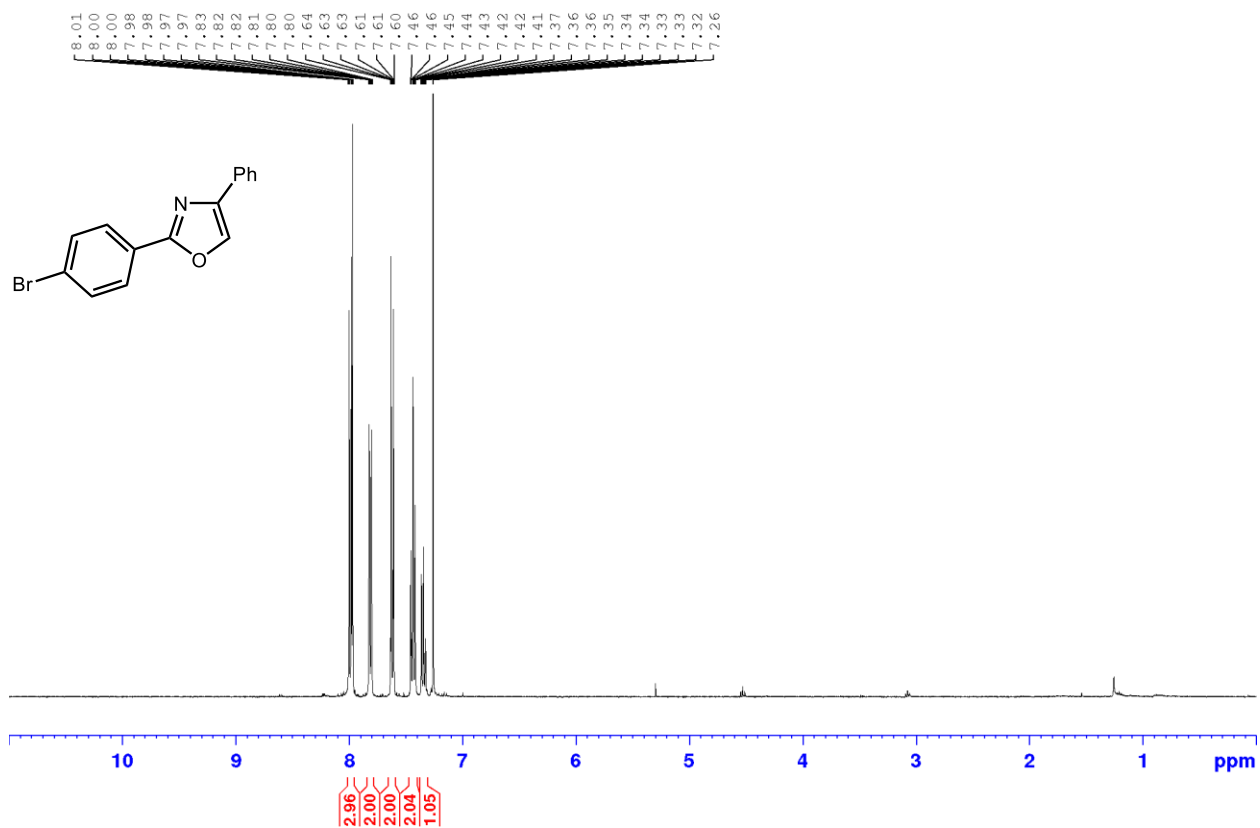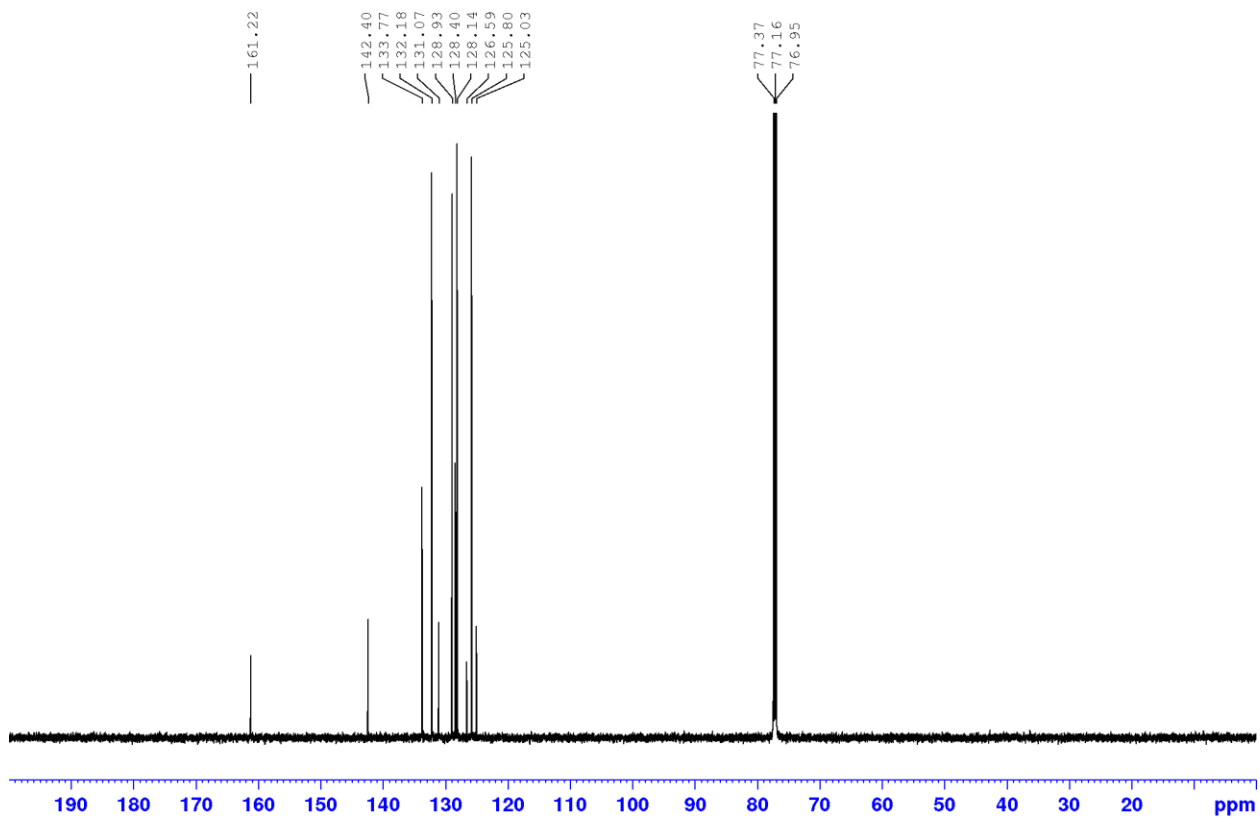

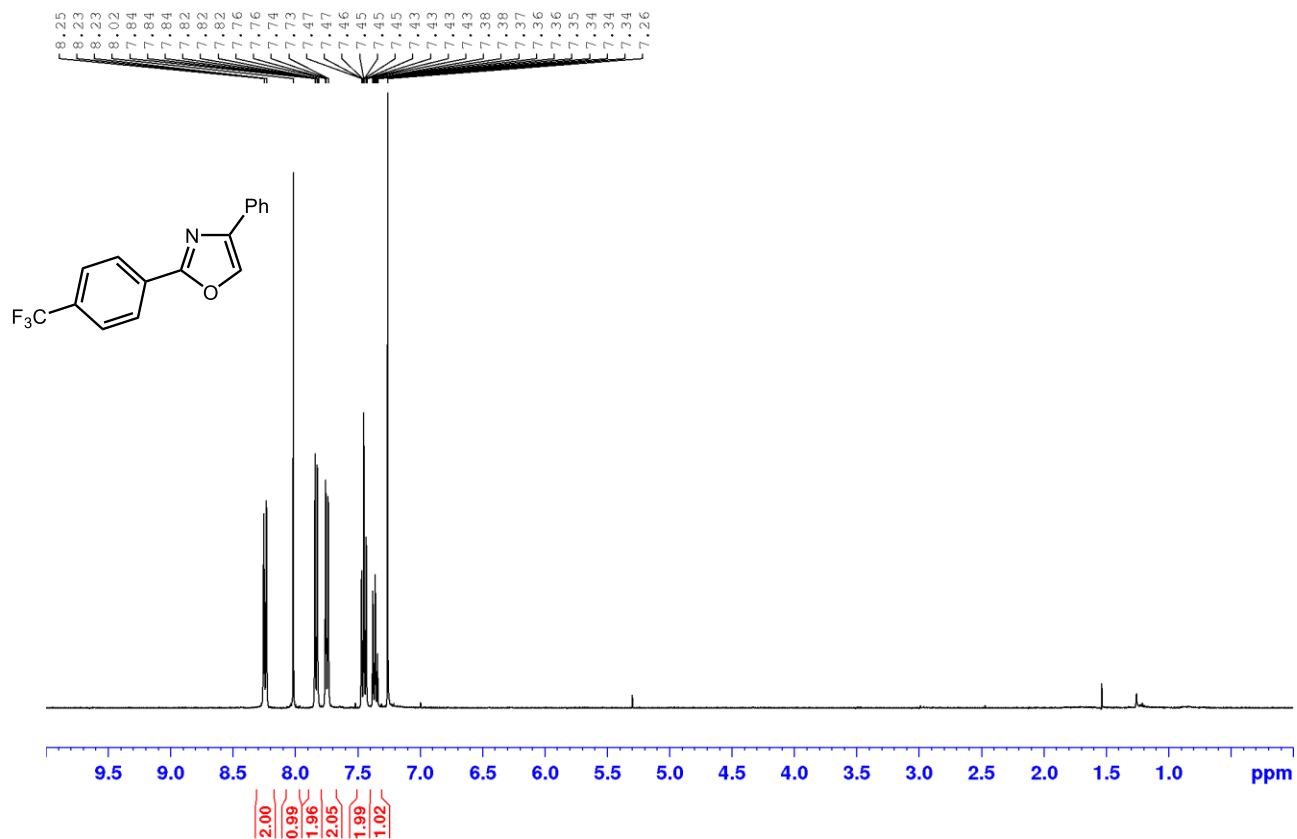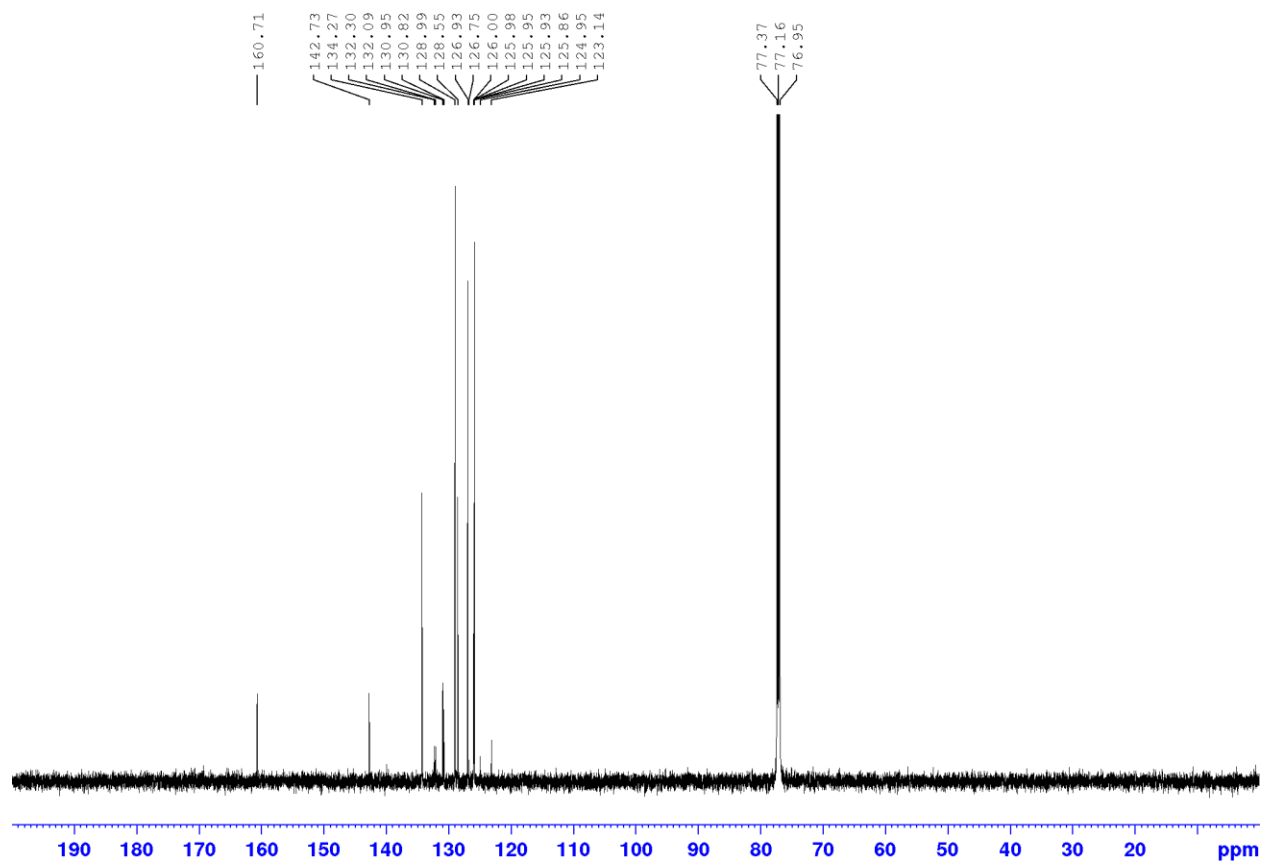

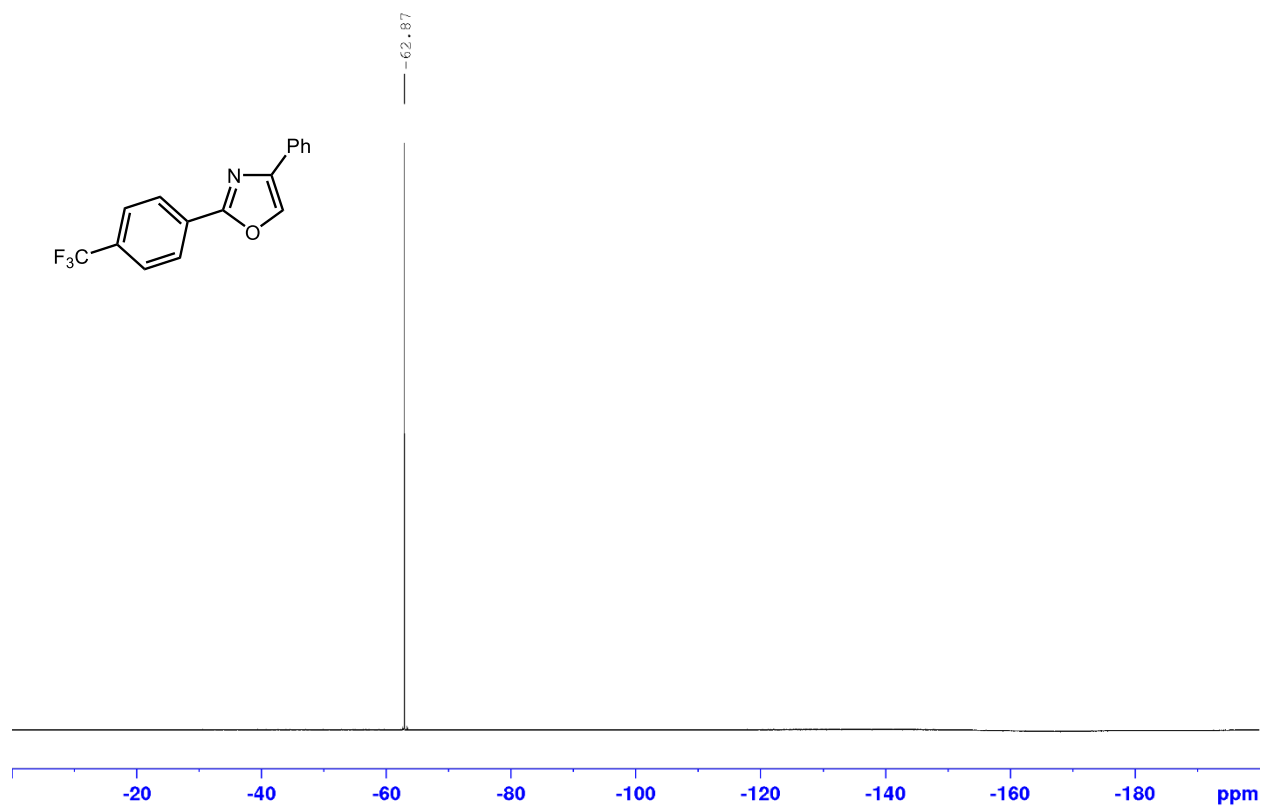

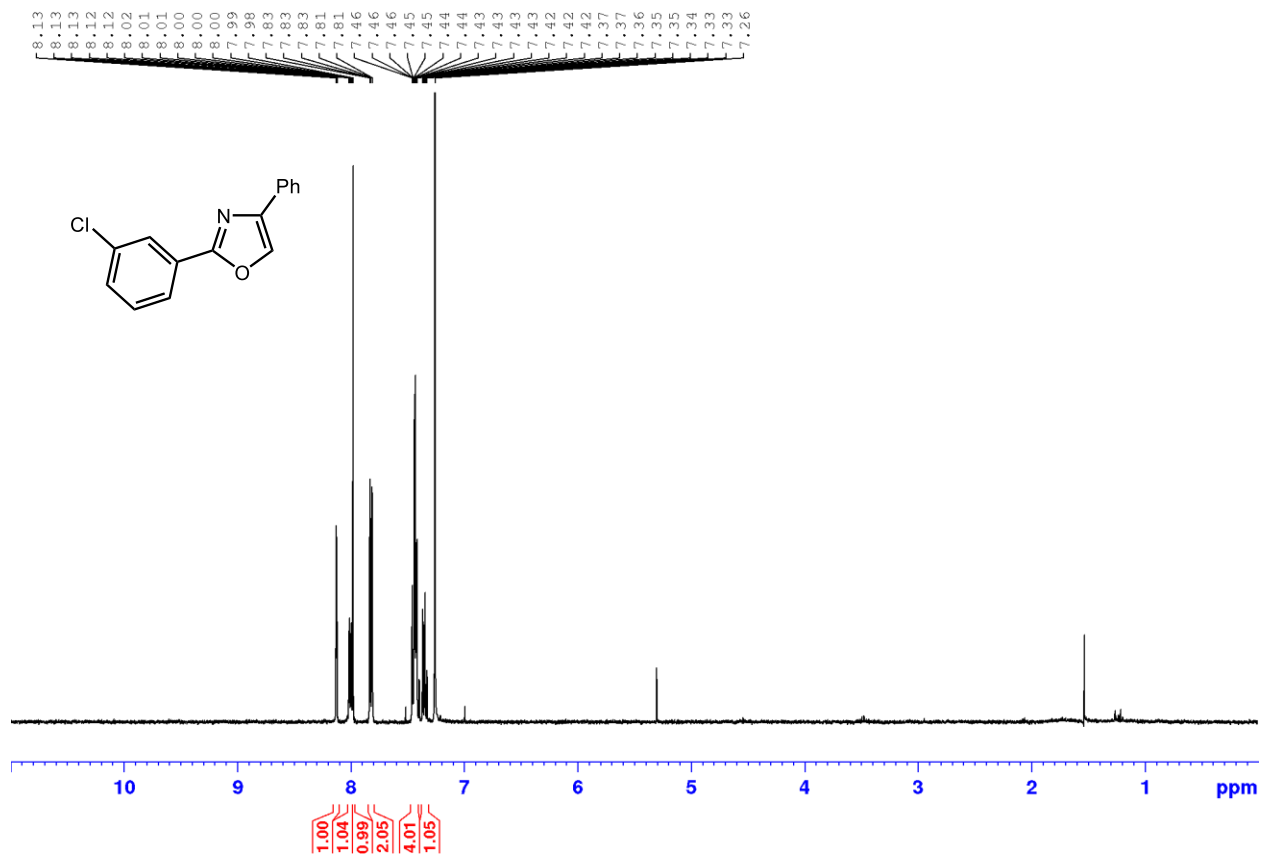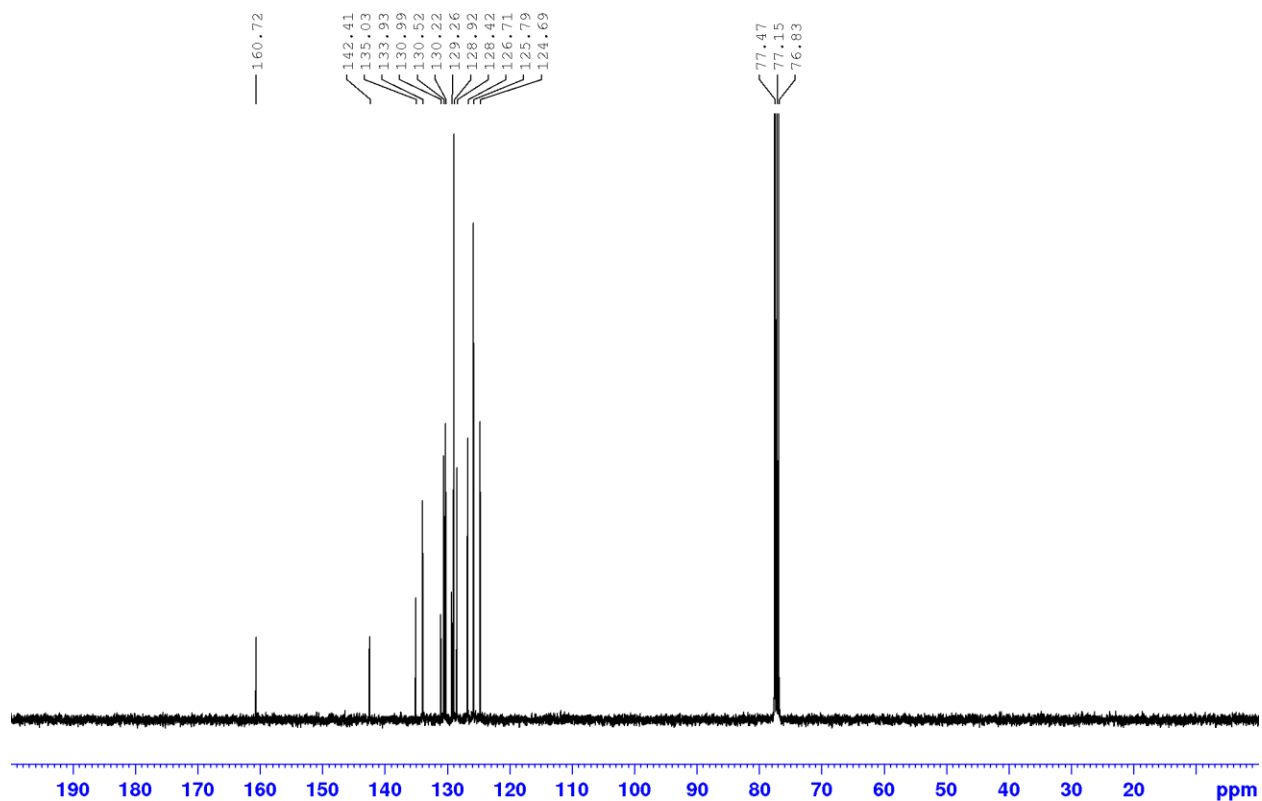

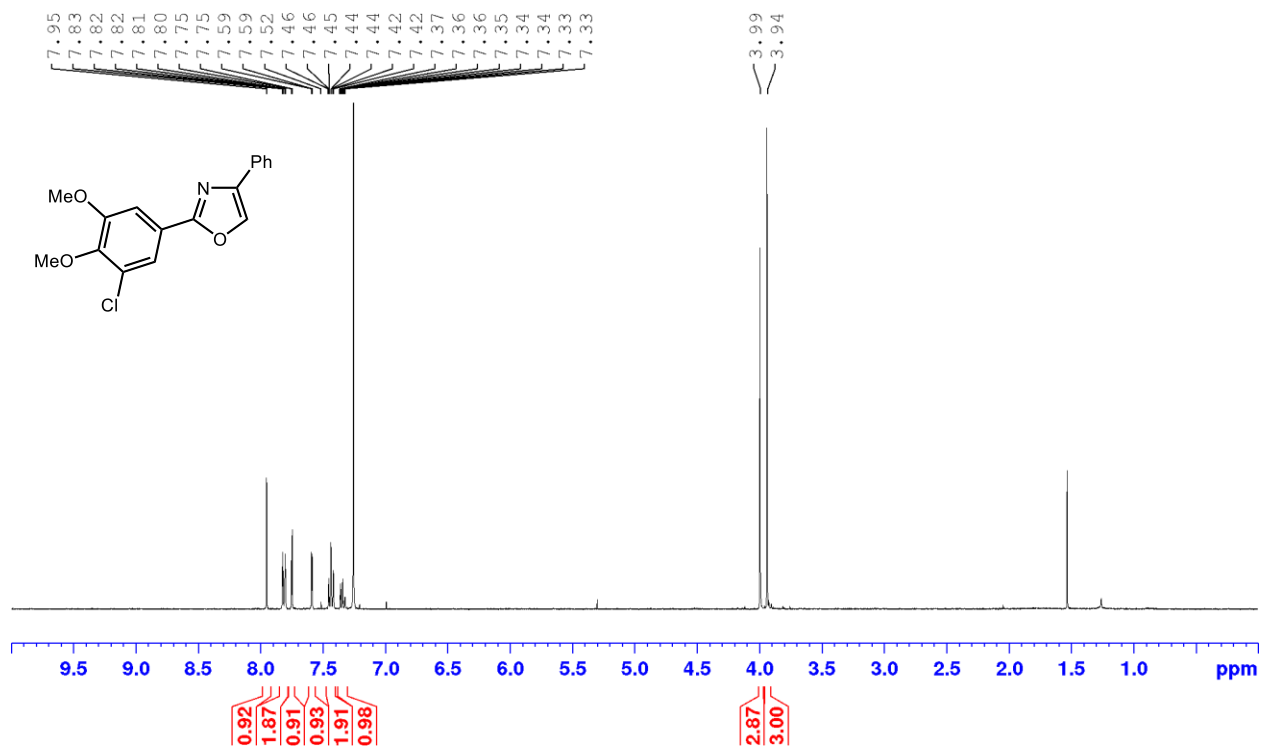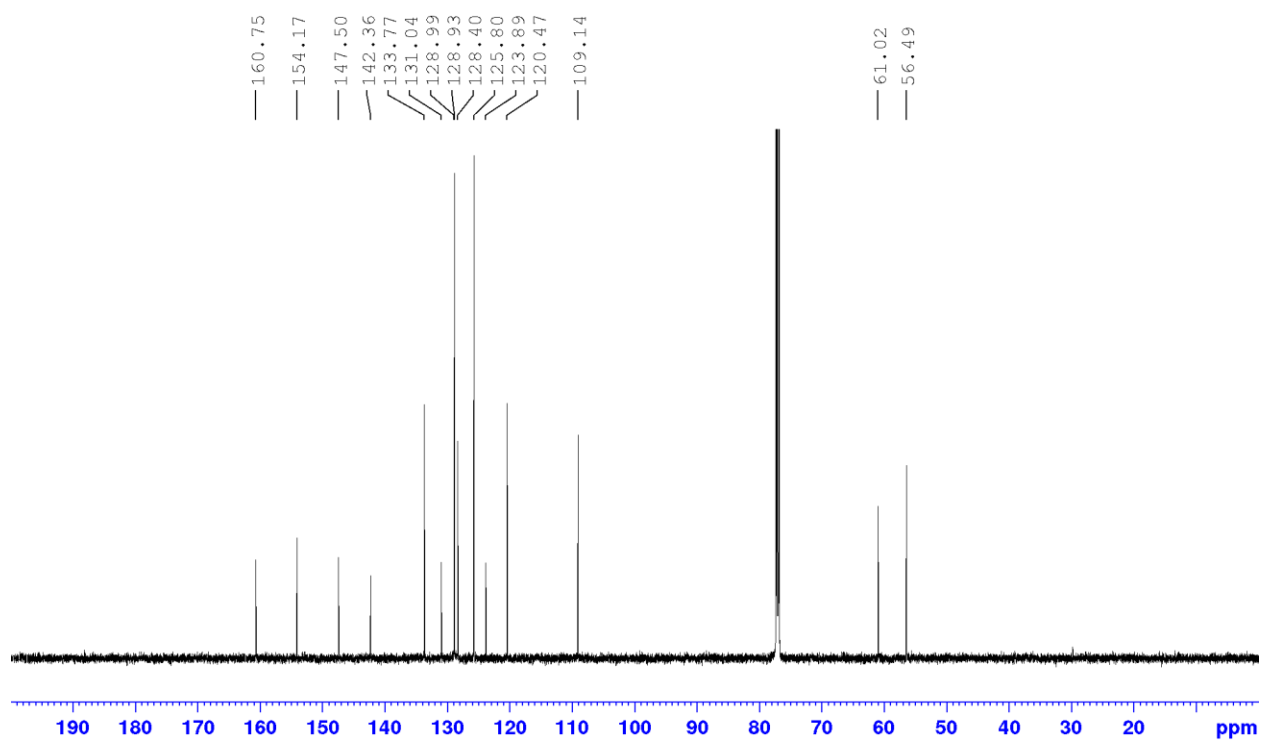

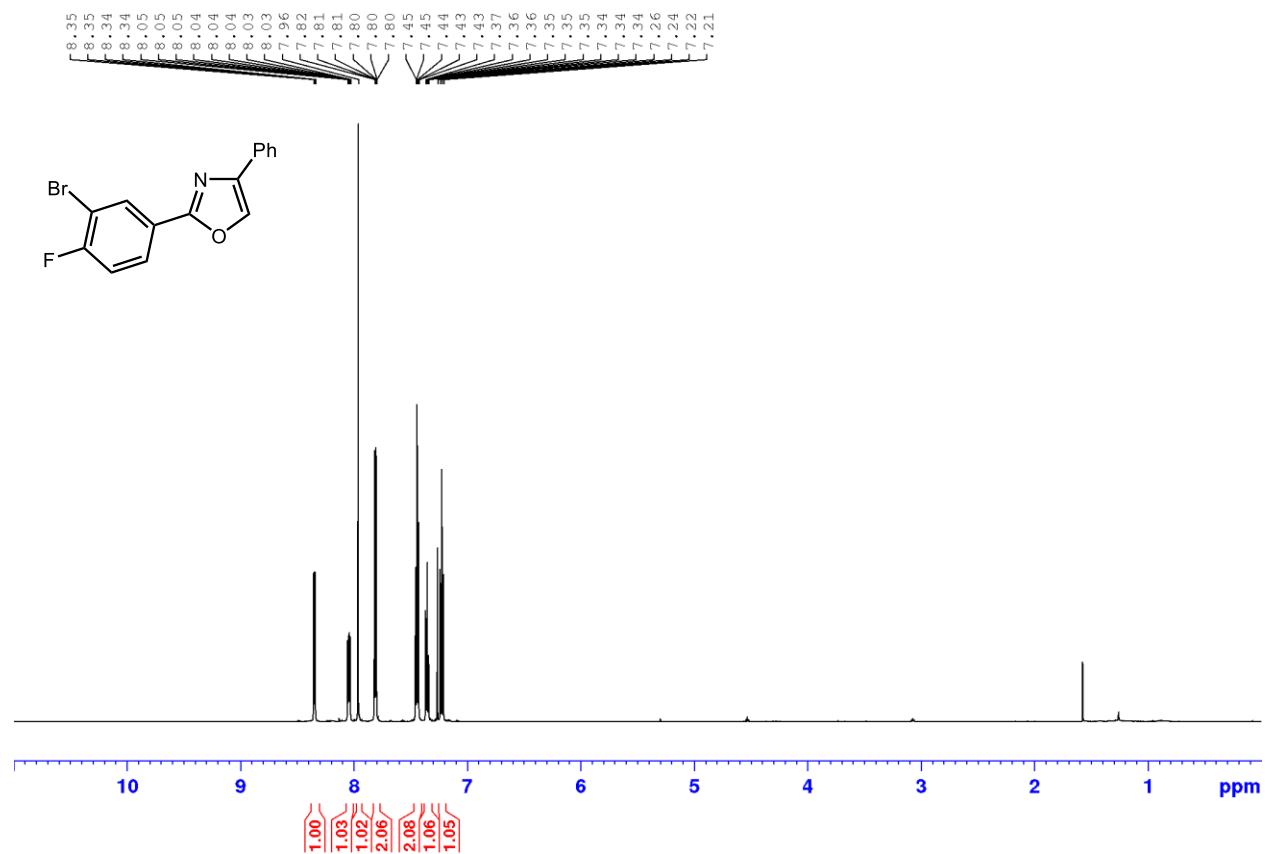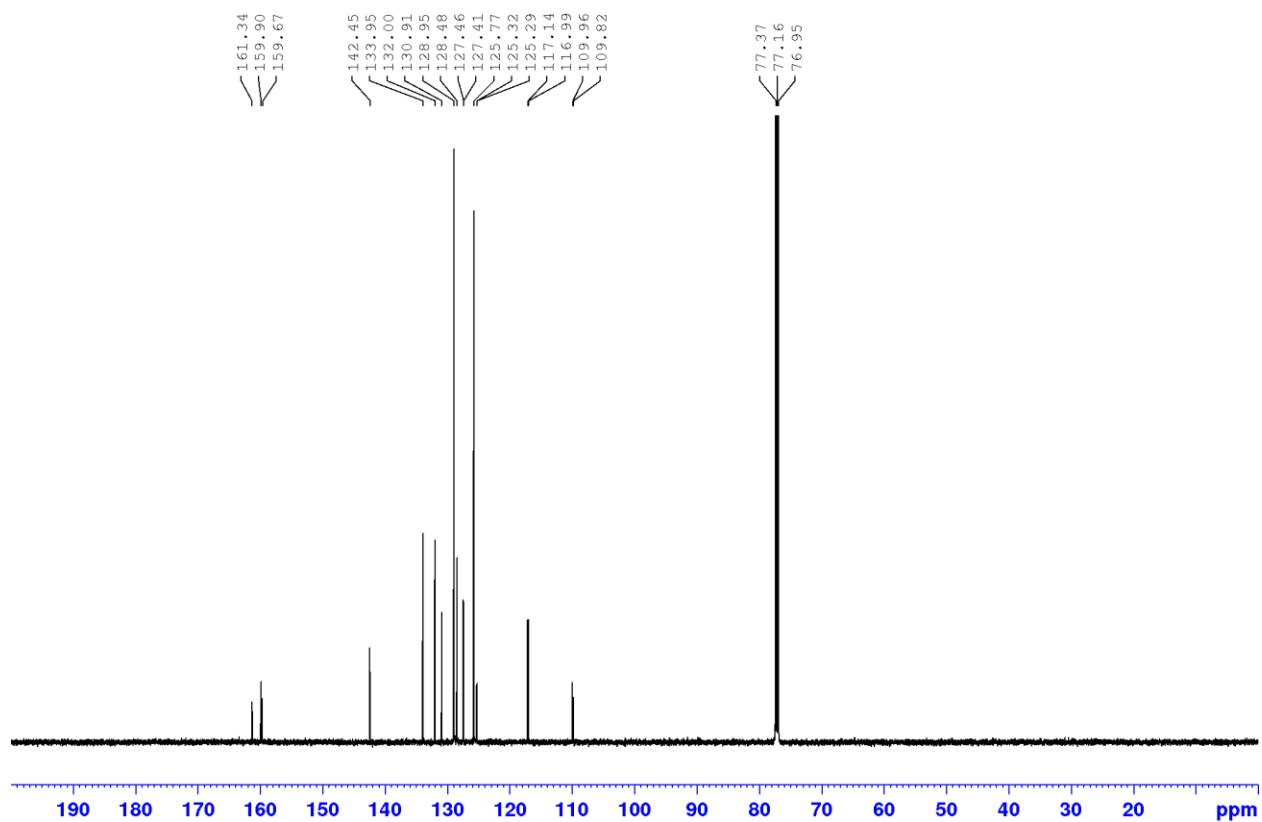

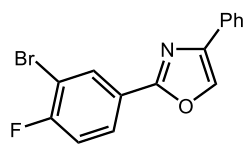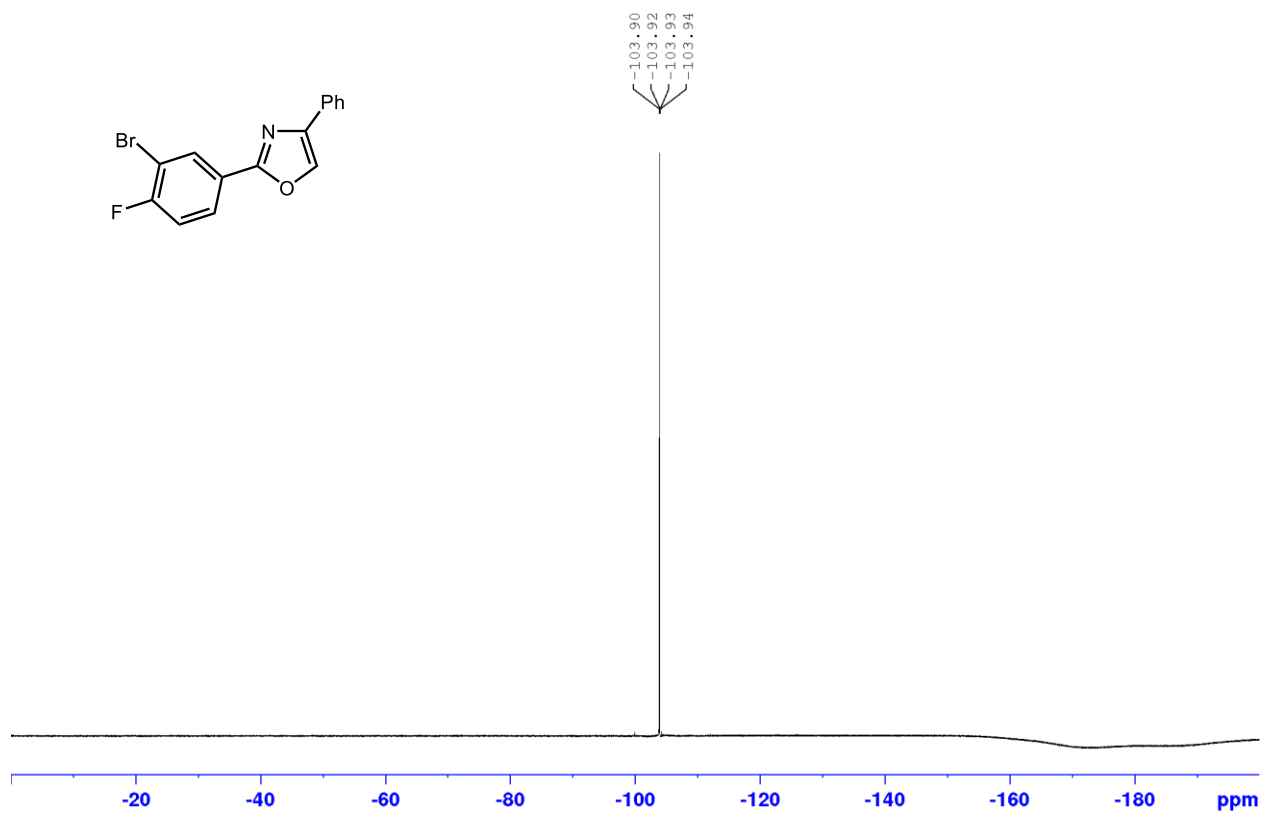

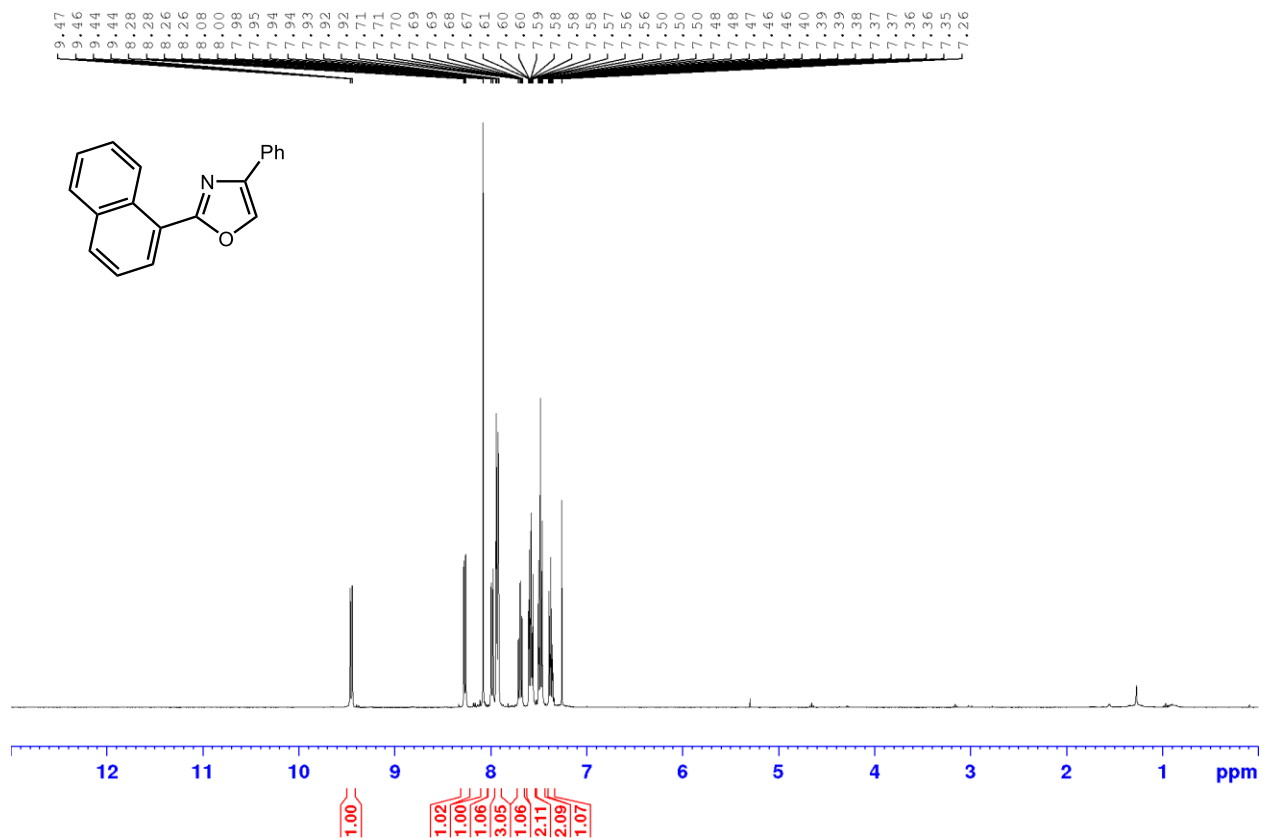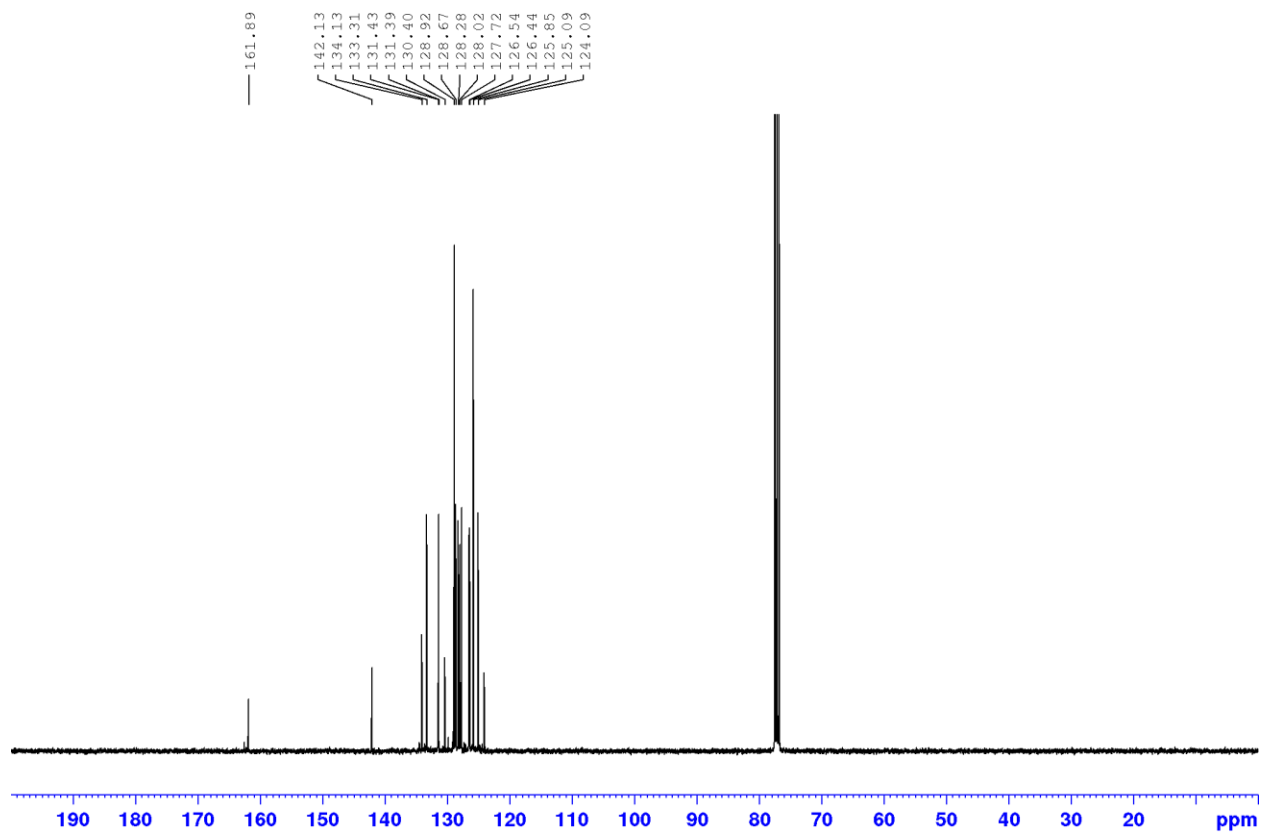

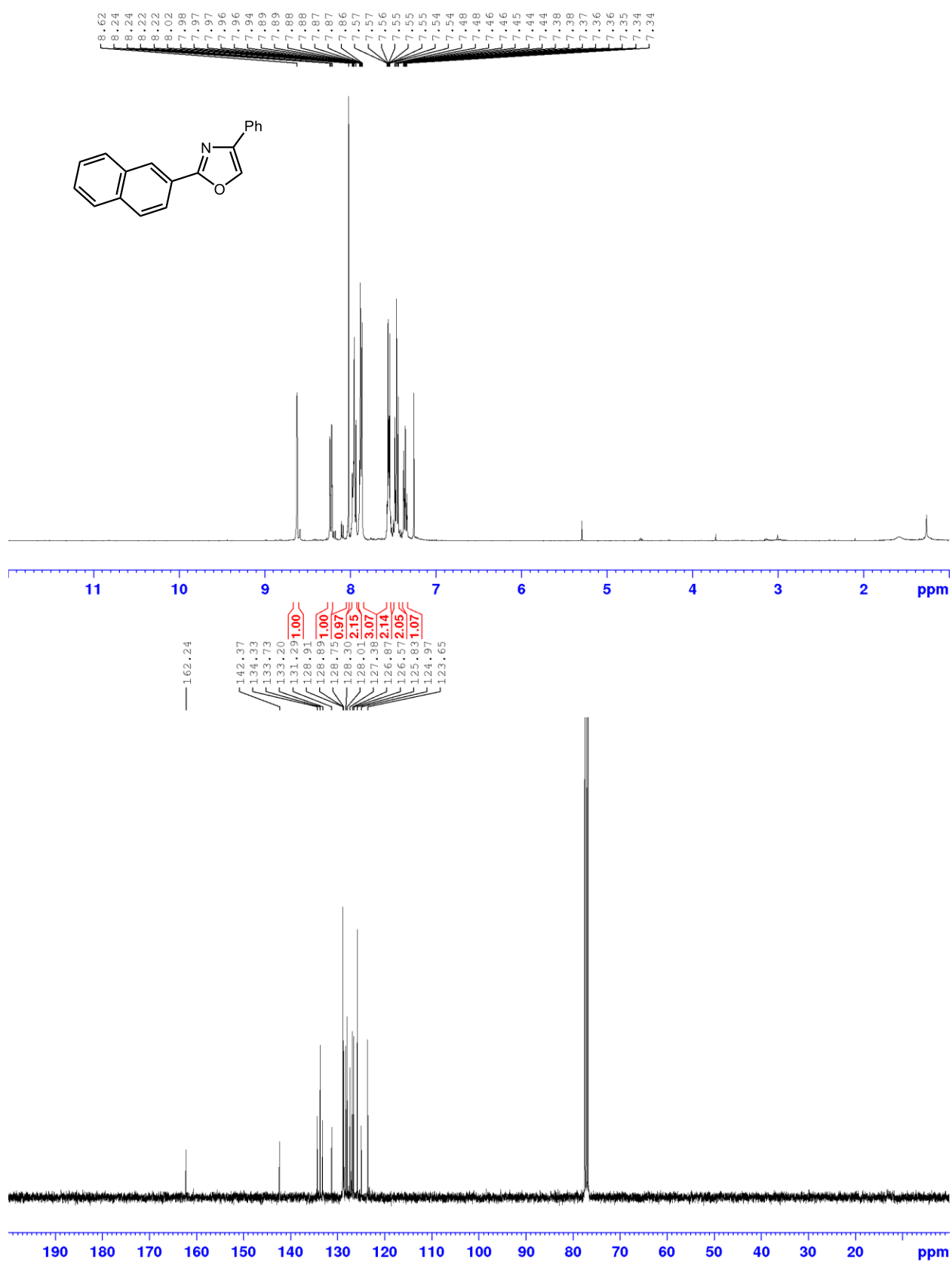

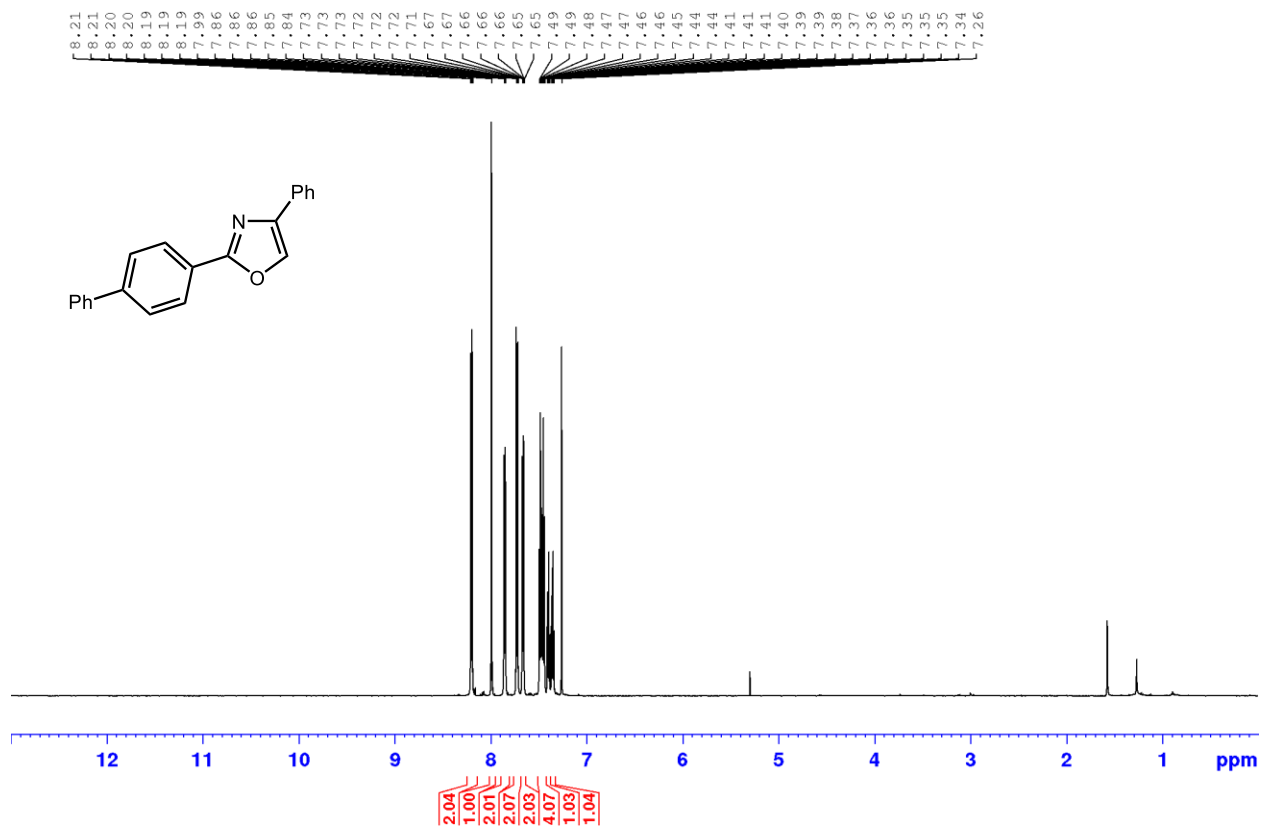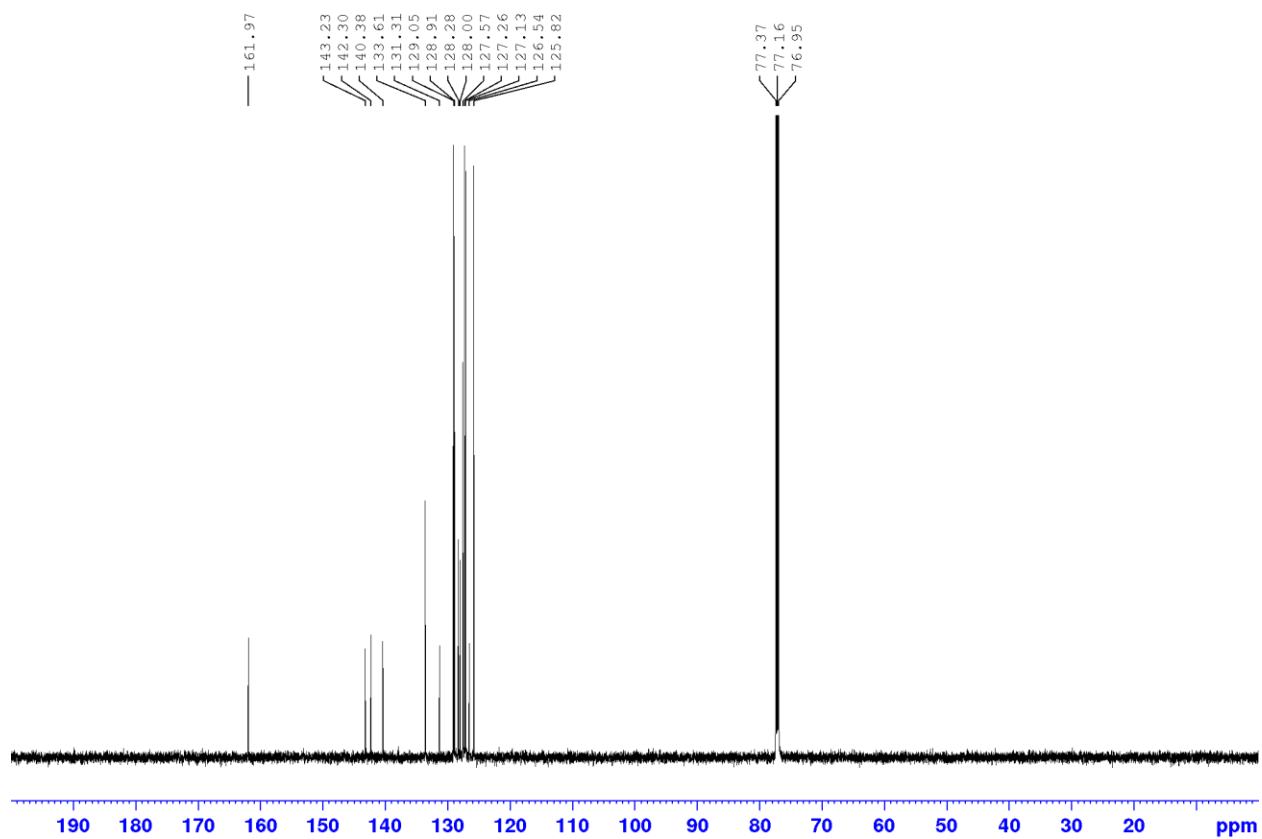

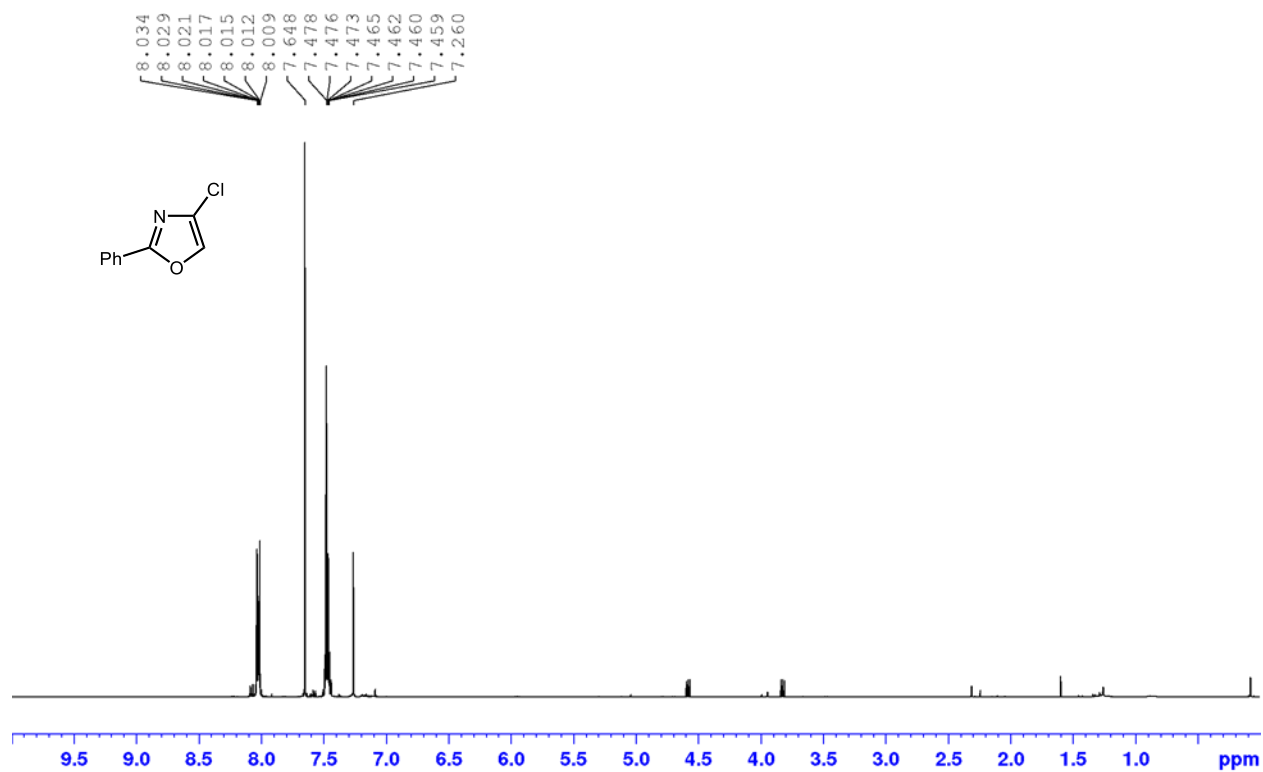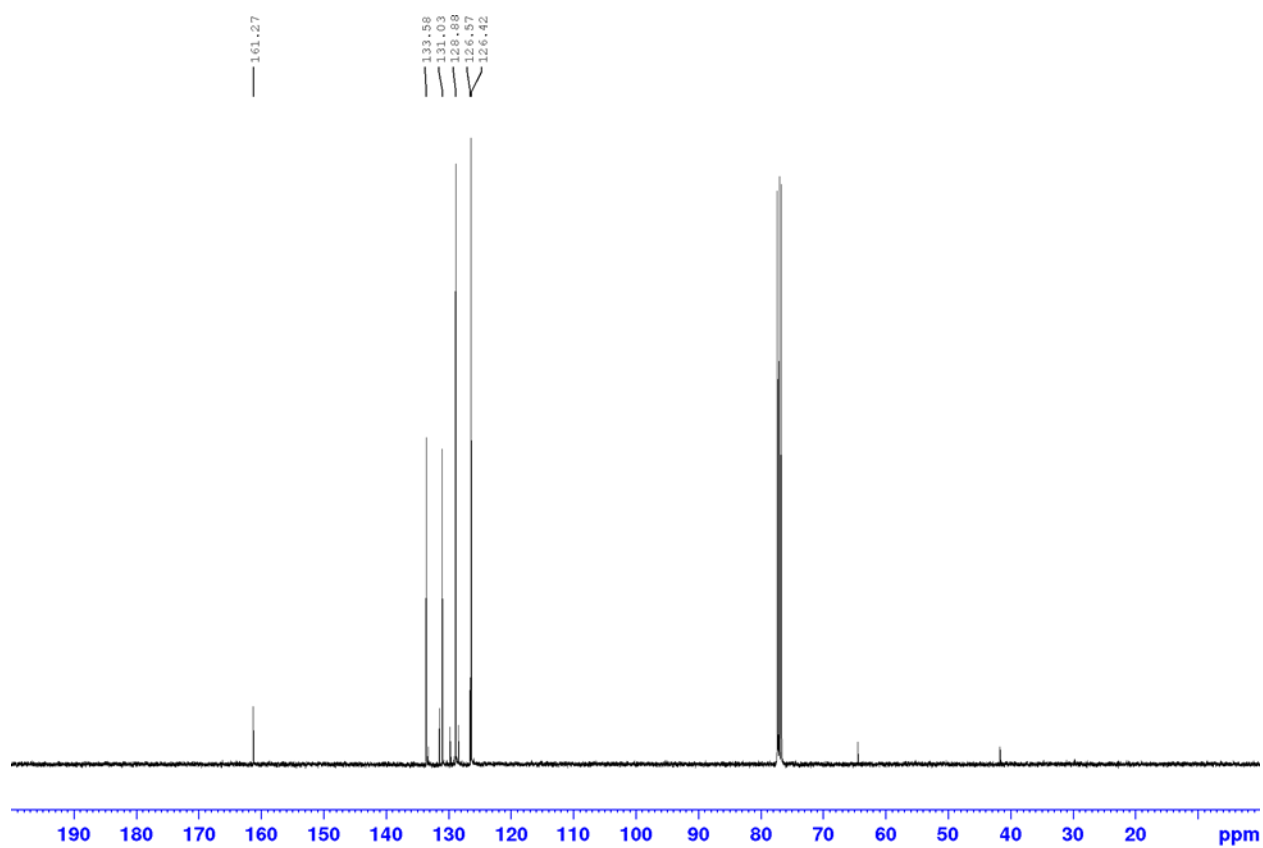

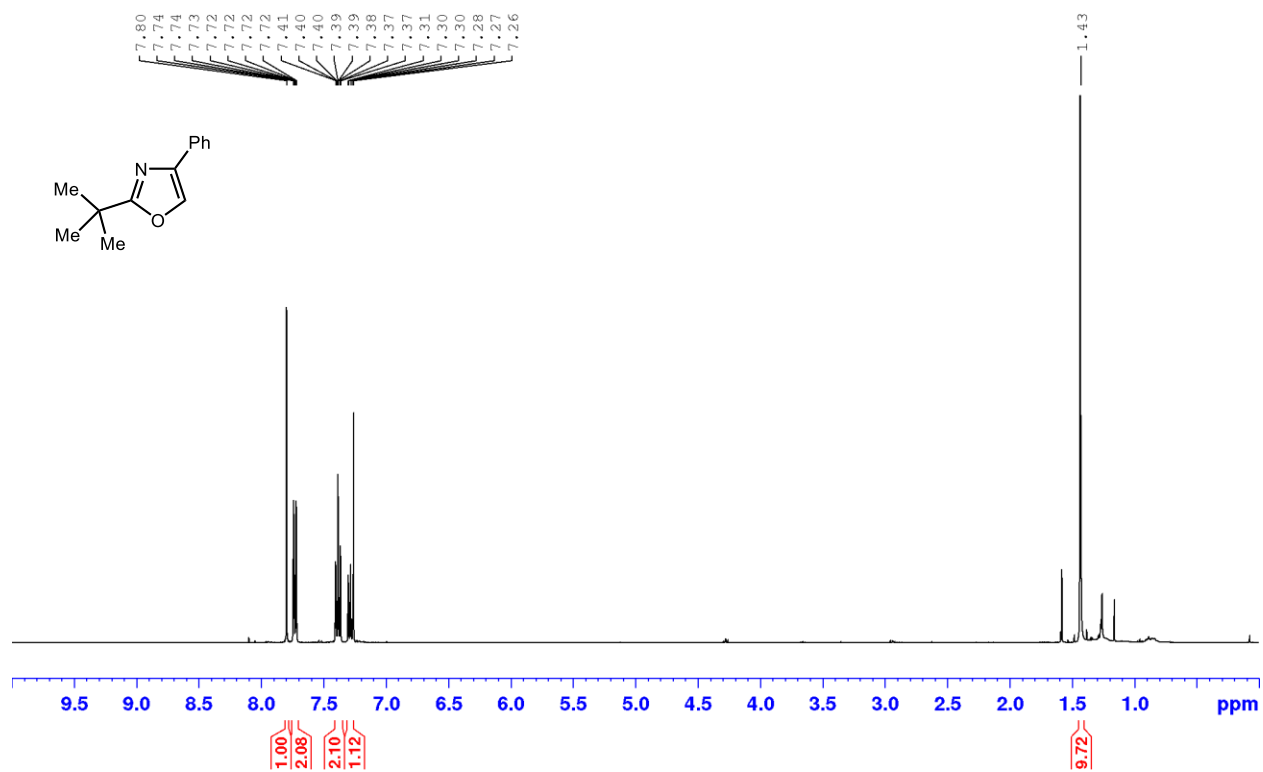

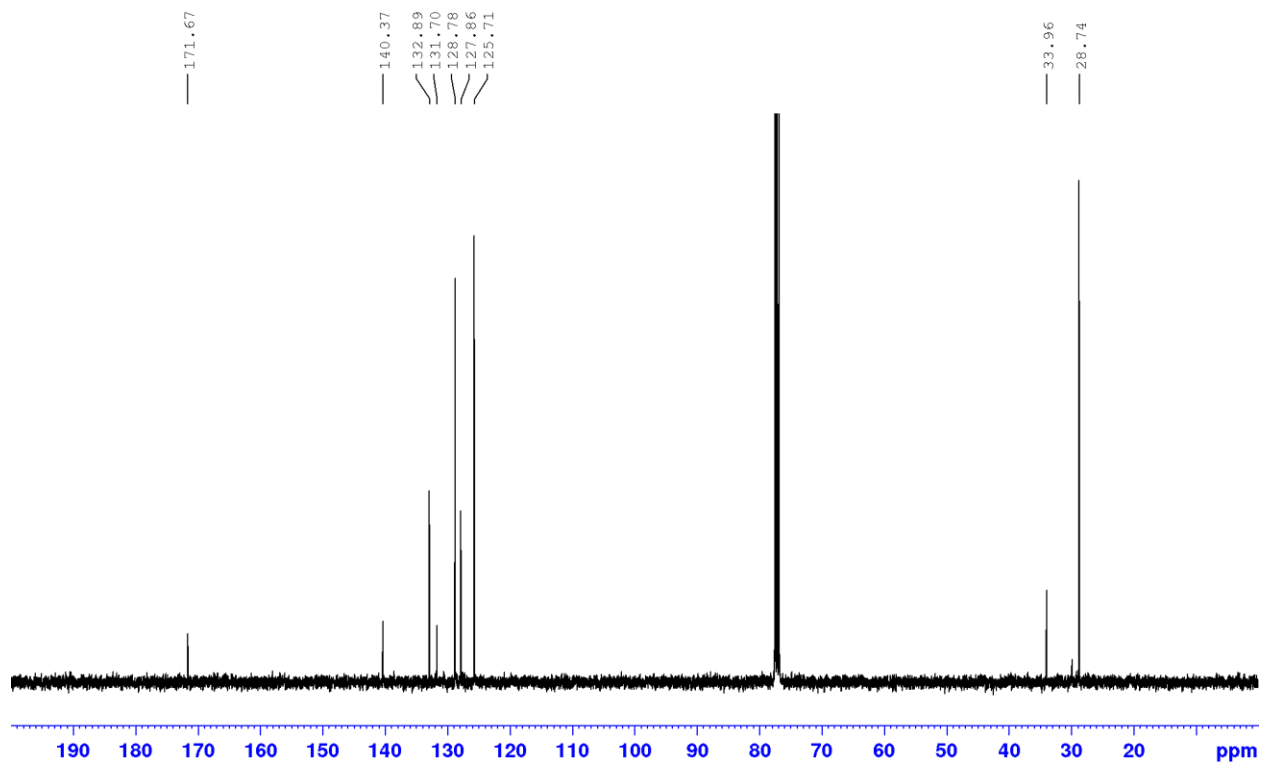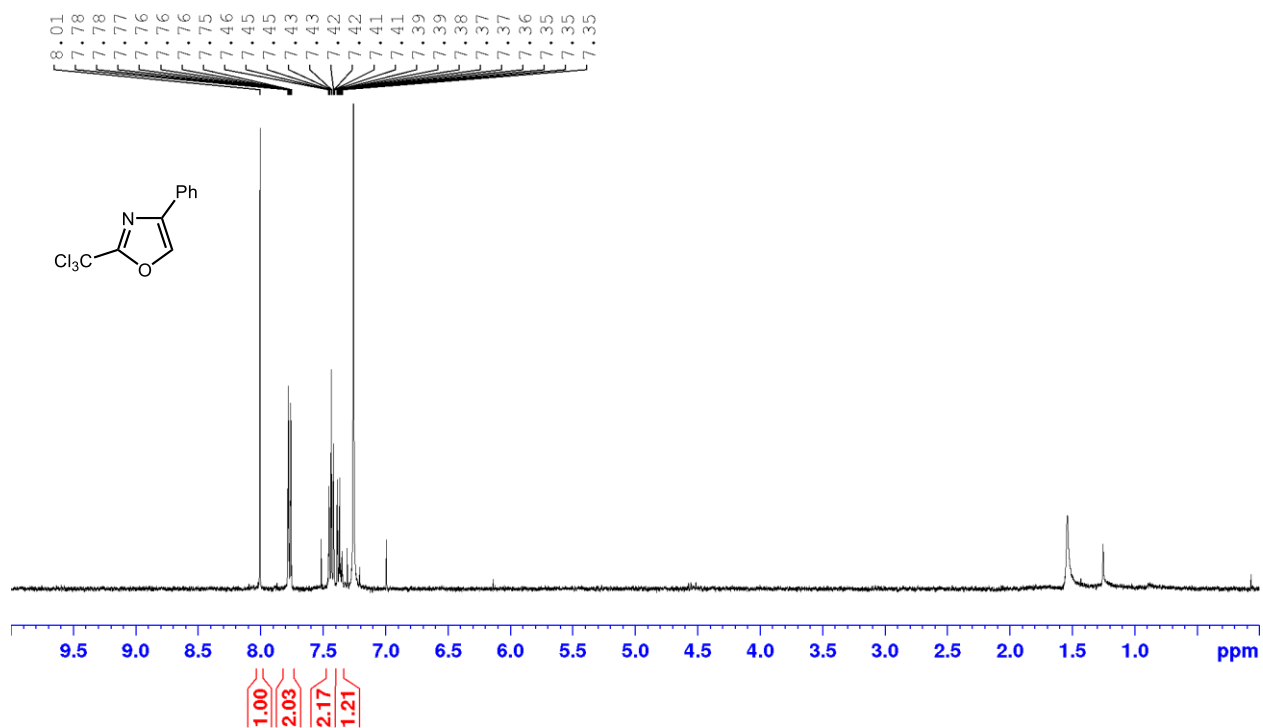

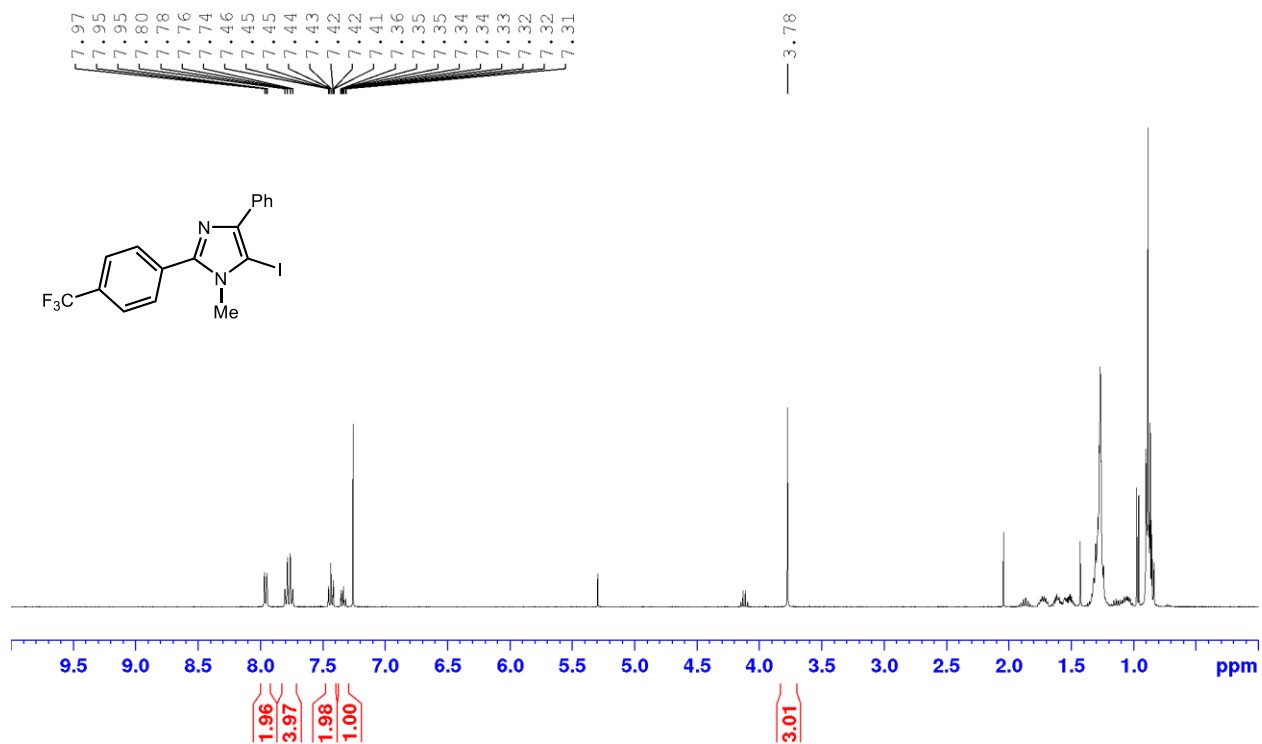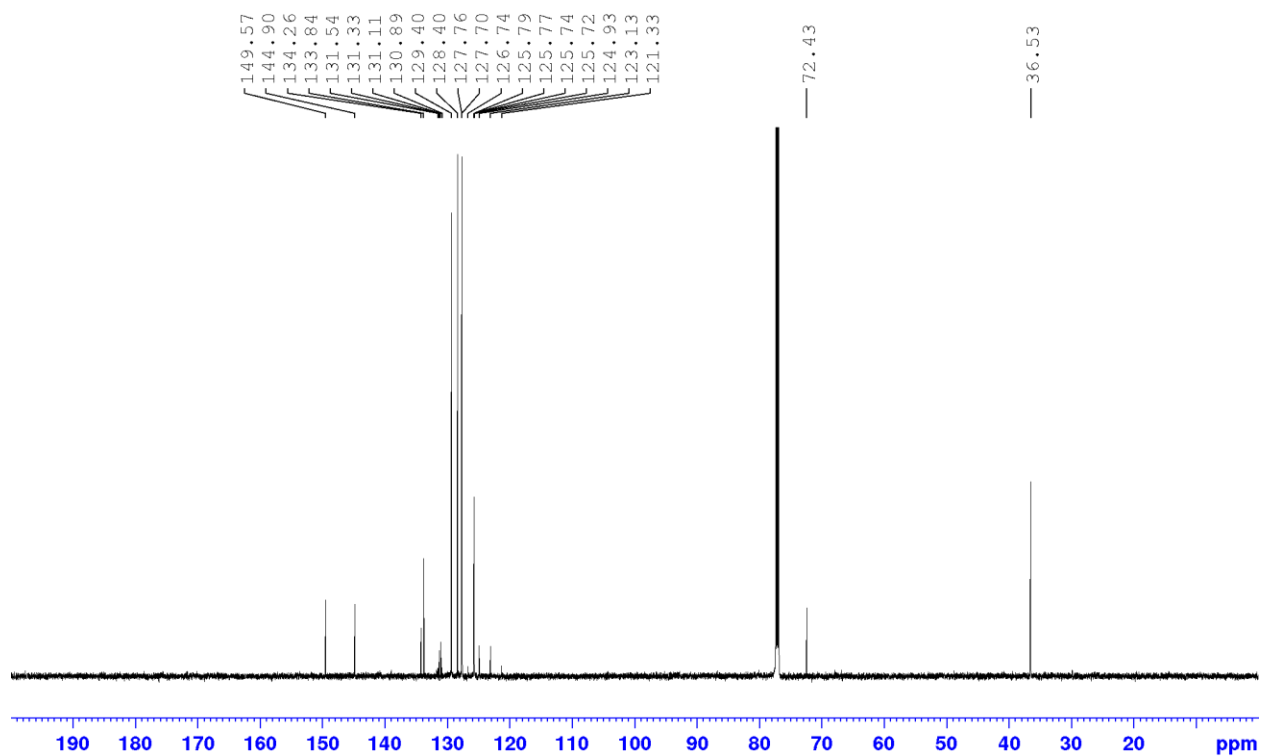

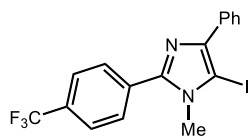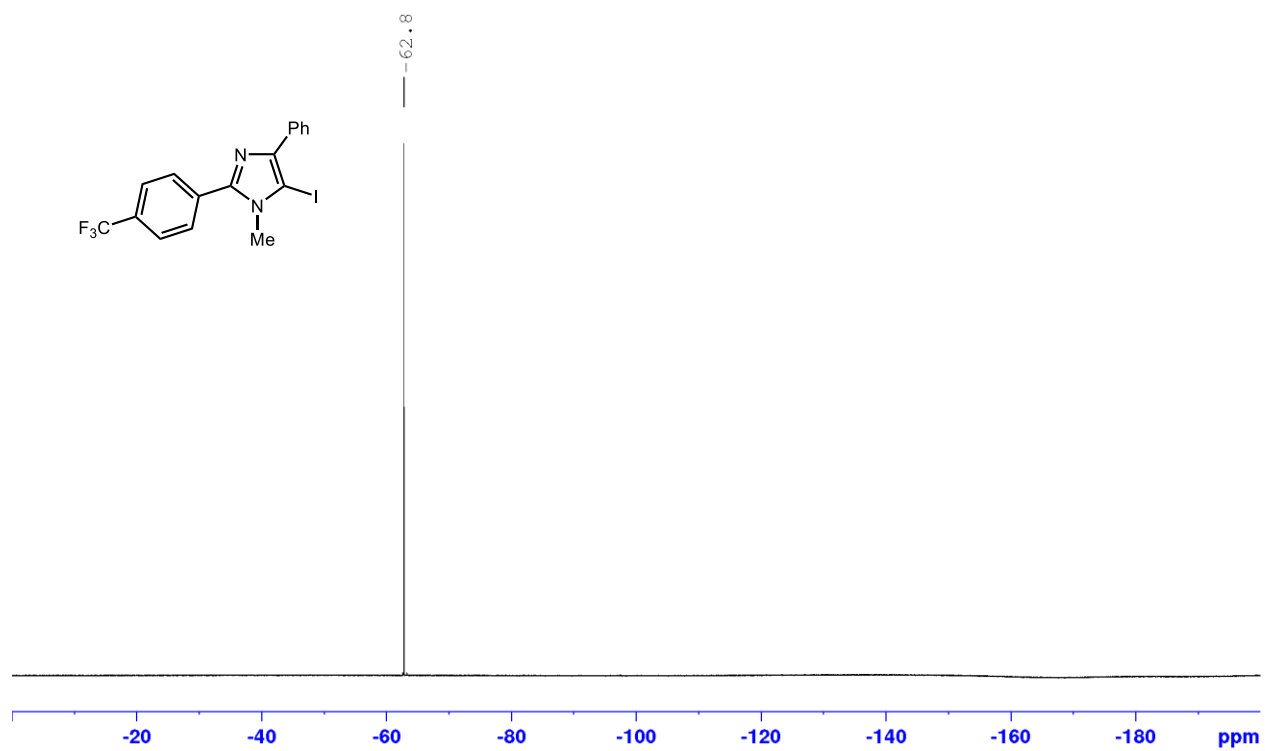

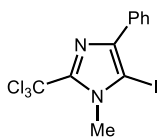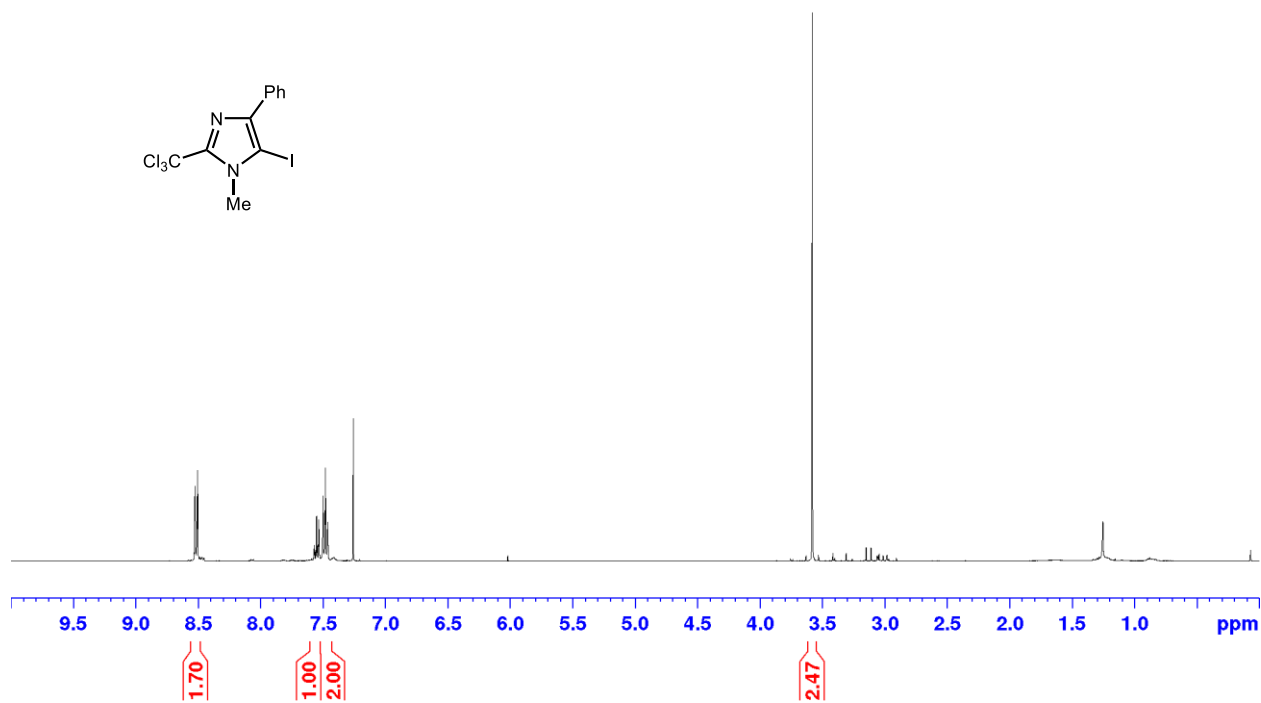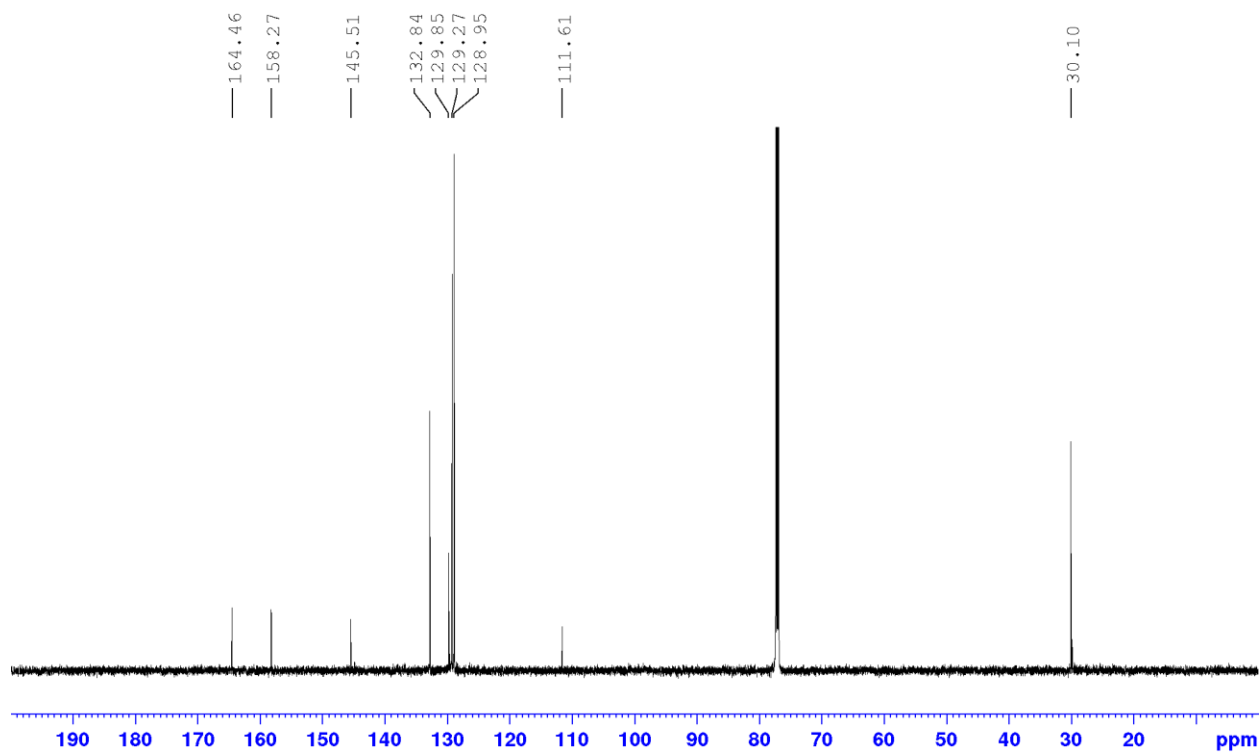

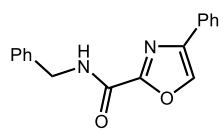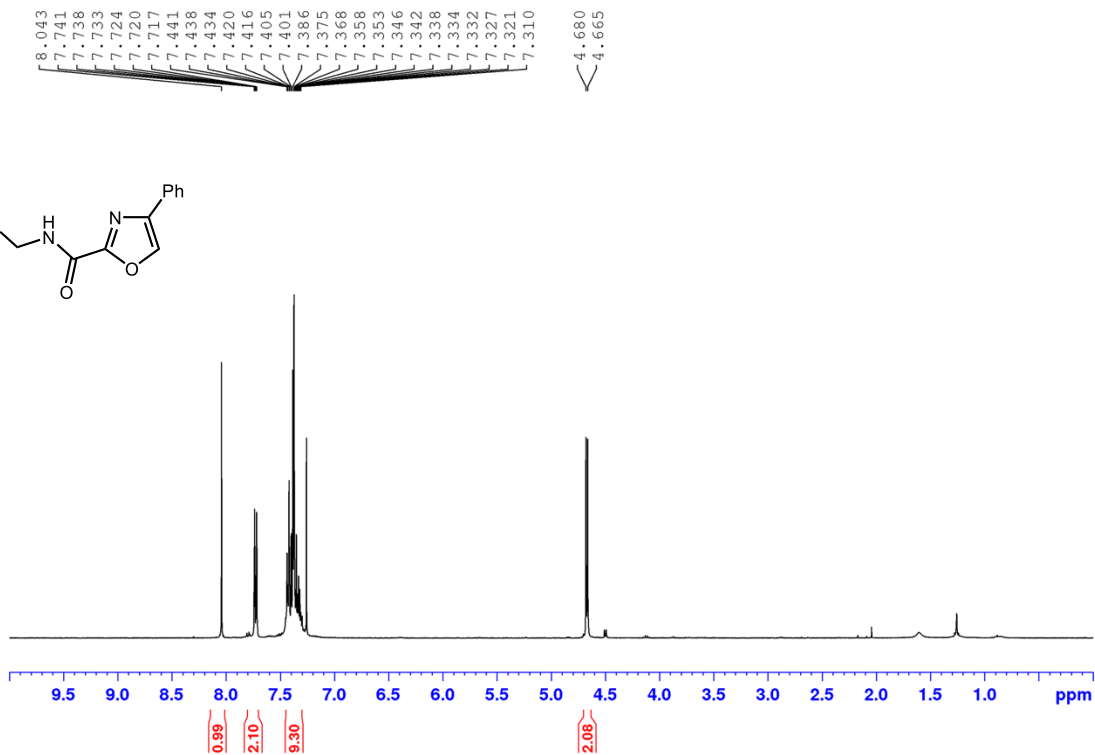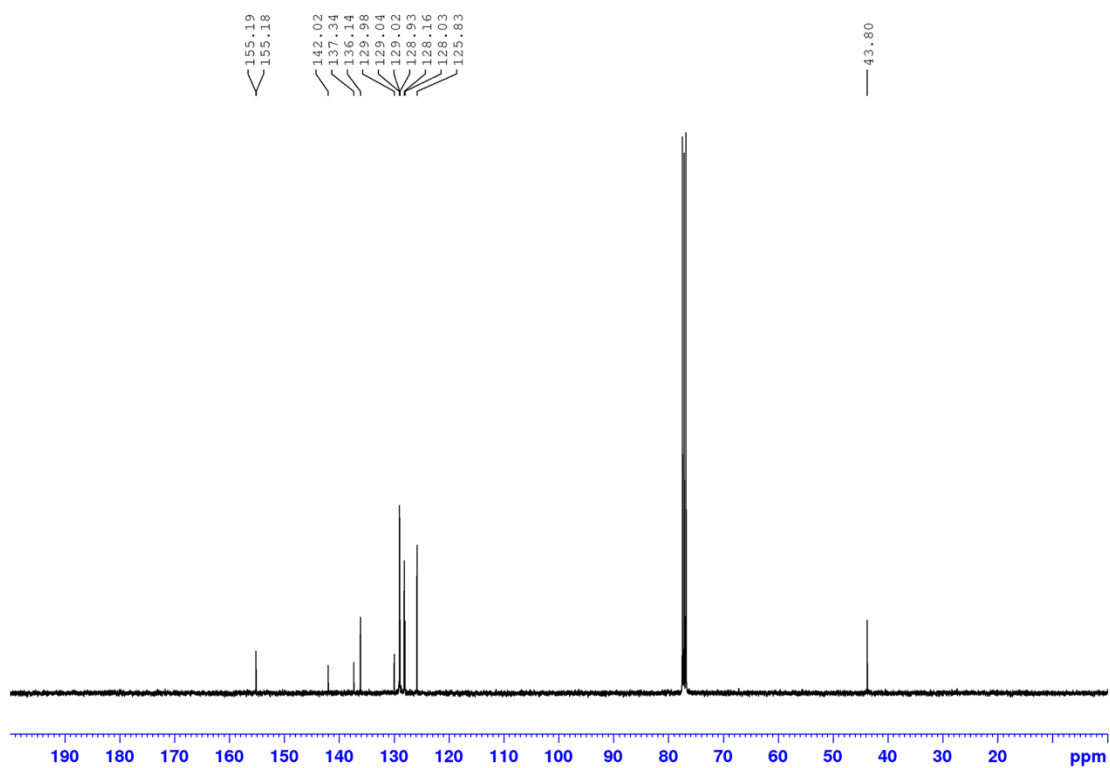

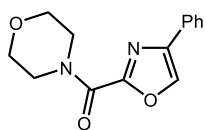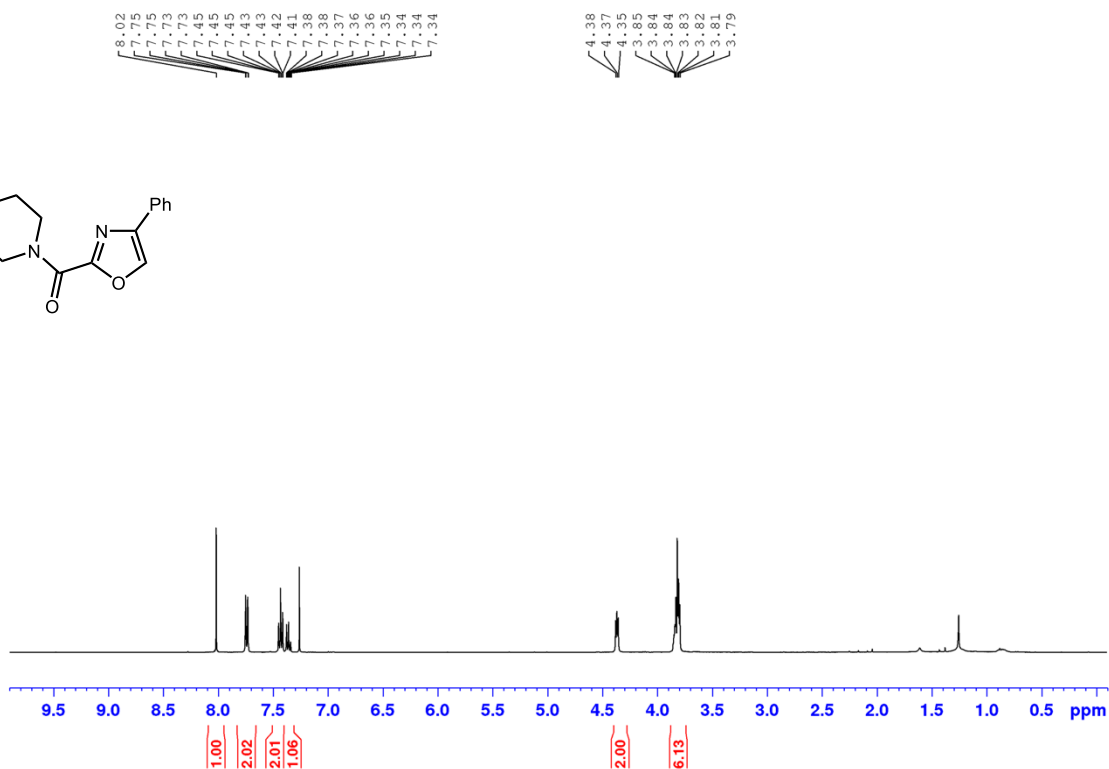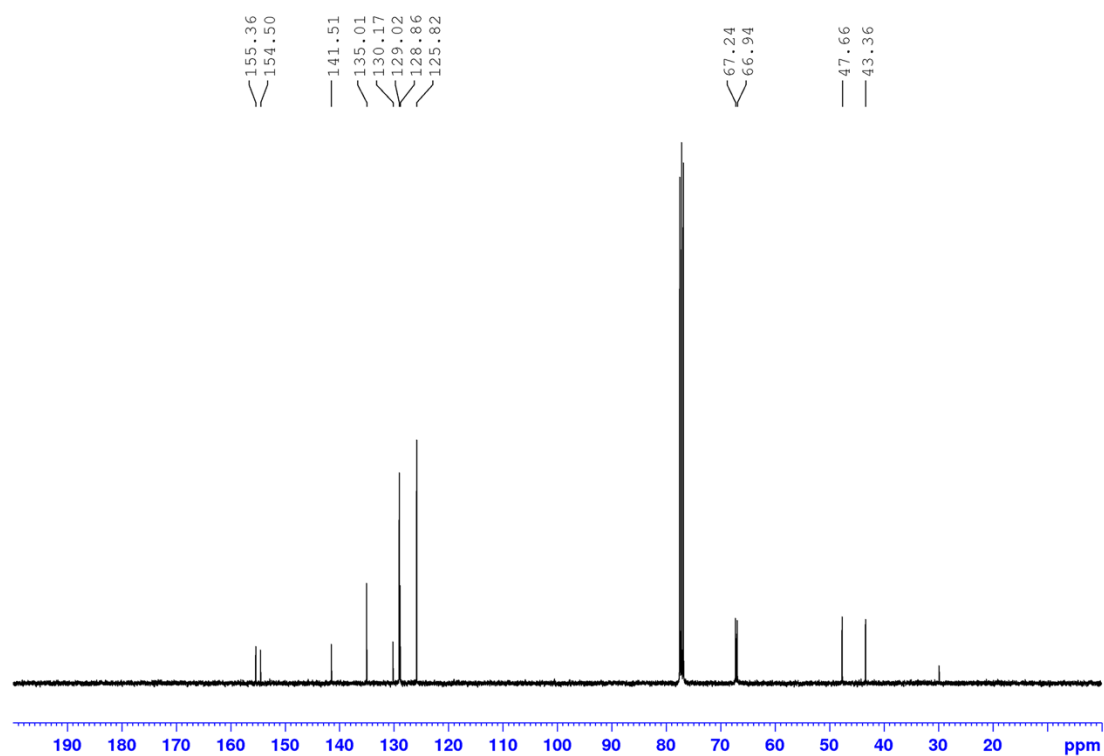

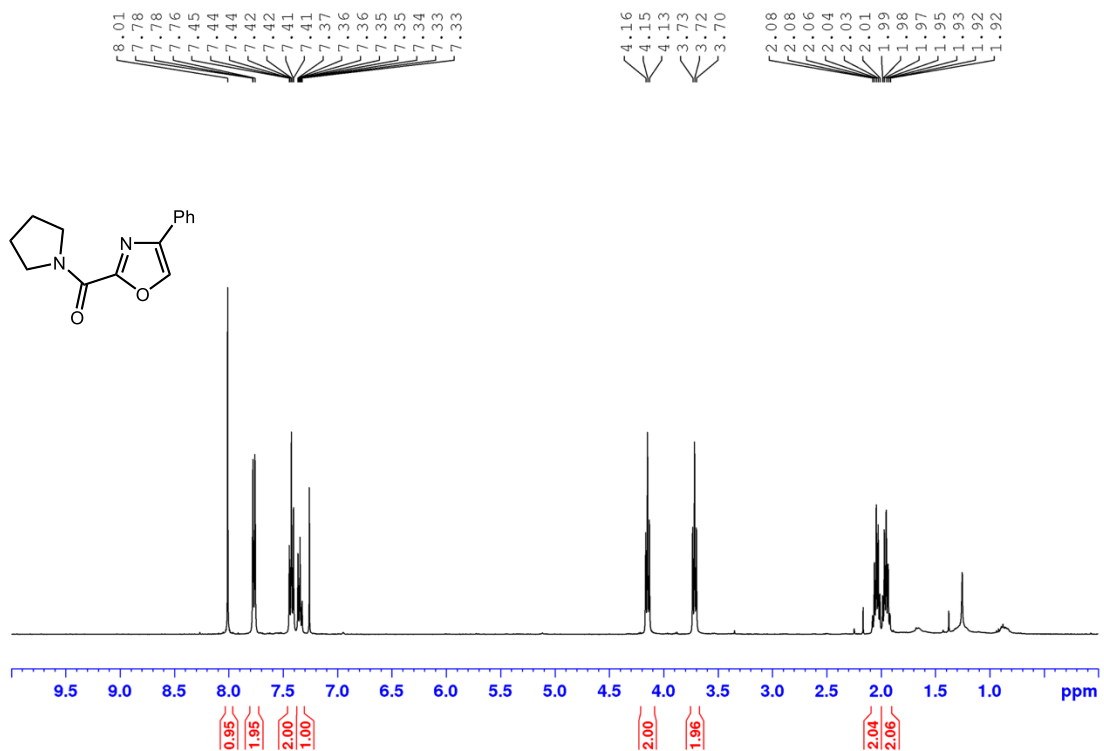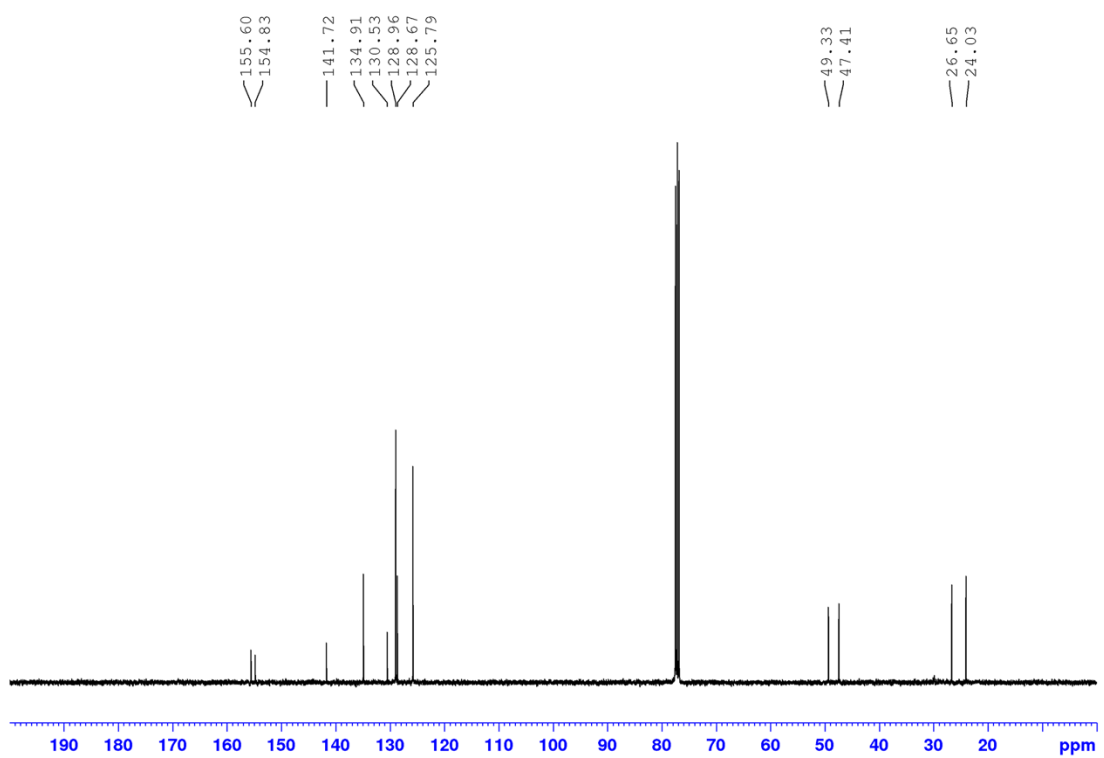

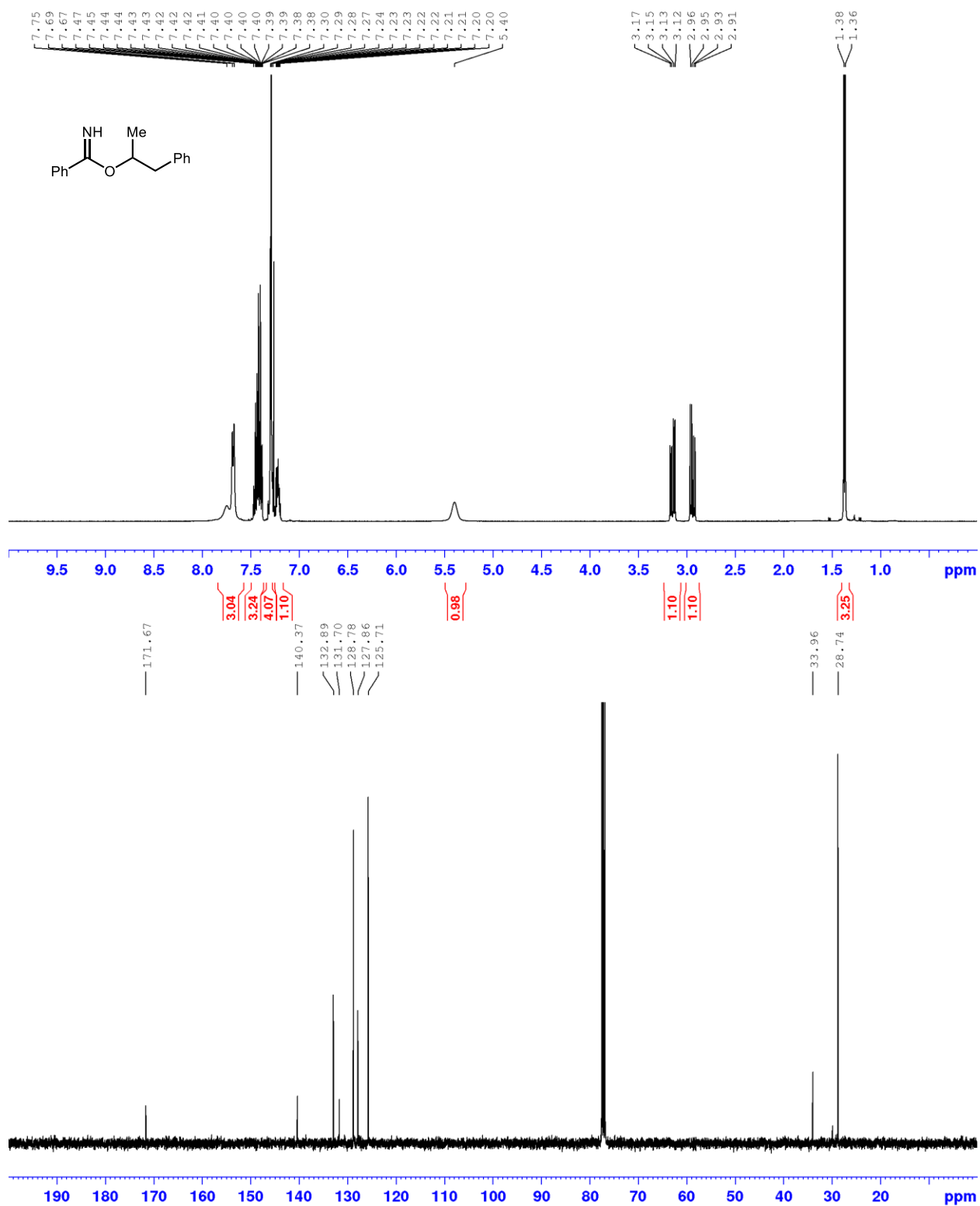

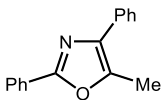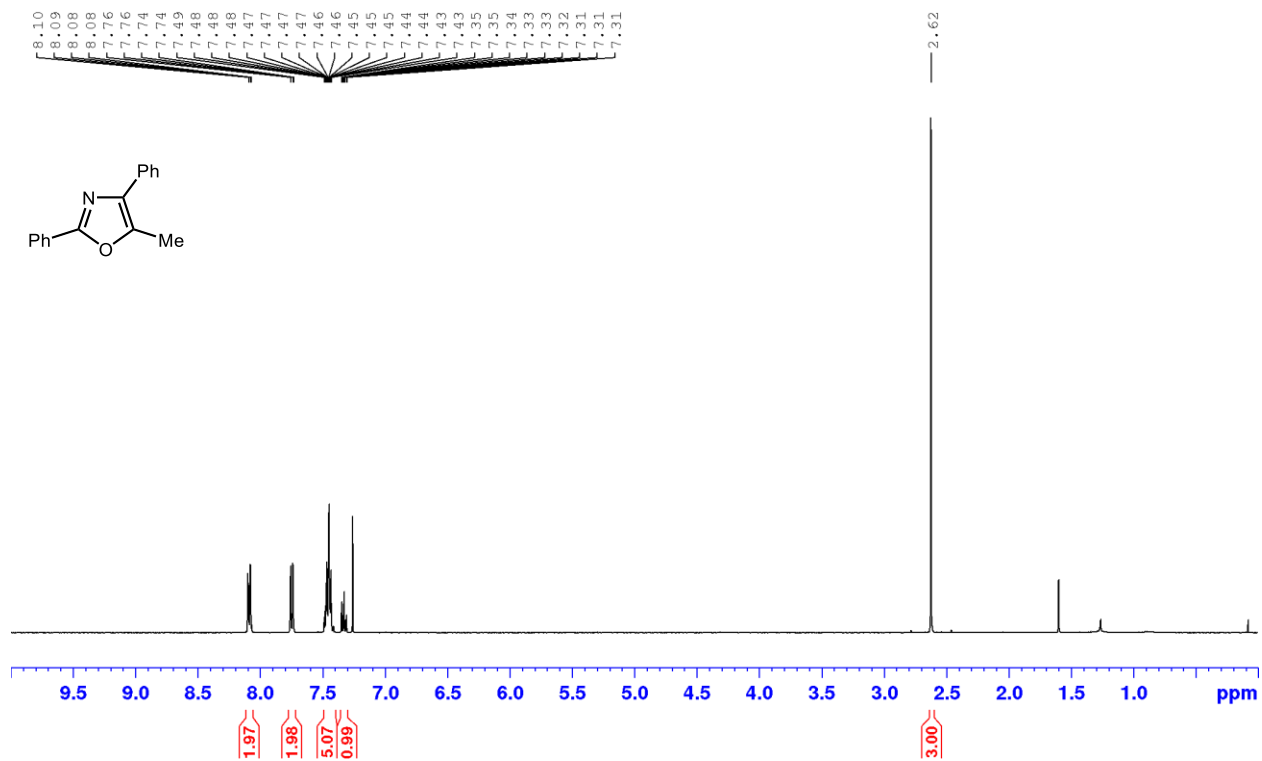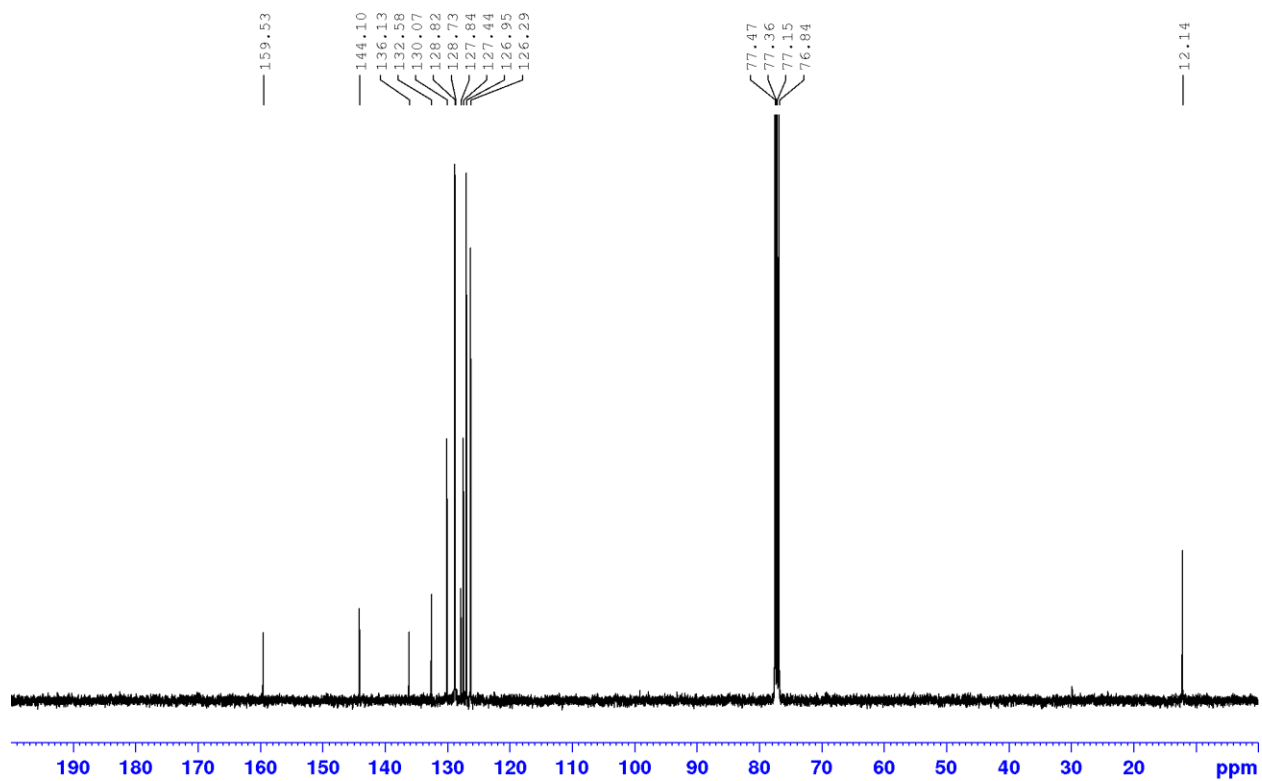

Supplement: SC-011-C9SC06239D-s001 [file SC-011-C9SC06239D-s001.pdf]
