# Supplementary material for: Radical cascade synthesis of azoles via tandem hydrogen atom transfer
Source: Chem Sci. 2020 Jan 31;11(9):2479–86. doi: 10.1039/c9sc06239d (PMC8157396; doi:10.1039/c9sc06239d)
Supplement: SC-011-C9SC06239D-s002 [file SC-011-C9SC06239D-s002.pdf]

Electronic Supplementary Material (ESI) for Chemical Science.
This journal is © The Royal Society of Chemistry 2020

Supplementary Information

Radical Cascade Synthesis of Azoles via Tandem Hydrogen Atom Transfer

Andrew D. Chen,† James H. Herbort,† Ethan A. Wappes,
Kohki M. Nakafuku, Darsheed N. Mustafa, David A. Nagib*

Department of Chemistry & Biochemistry
The Ohio State University
151 W Woodruff Ave., Columbus, OH 43210

Corresponding Author
* E-mail: nagib.1@osu.edu

Table of Contents
(with links)

		Page
I.	General Information	S3
II.	General Procedures	S4
III.	Imidate Synthesis Optimization	S6
IV.	Substrate Synthesis	S8
V.	Tandem Oxidation Optimization	S22
VI.	Tandem Oxidation	S26
VII.	One-Pot Optimization	S39
VIII.	Post-Synthetic Functionalization	S41
IX.	Mechanistic Studies	S43
X.	Substrate Limitations	S47
XI.	Commercial Availability of Feedstock Chemicals	S48
XII.	Computational Studies	S49

I. General Information

All chemicals and reagents were purchased from Sigma-Aldrich, Alfa Aesar, Acros, TCI, or ChemImpex. DCE, MeCN and Et_3N were distilled over CaH_2 before use. PhMe, CH_2Cl_2, and Et_2O were dried using an Innovative Technology solvent system. Silicycle F60 (230-400 mesh) silica gel was used for flash column chromatography. Thin layer chromatography (TLC) analyses were performed using Merck silica gel 60 F254 plates and visualized under UV (254 nm) or $KMnO_4$ stain. ^1H, ^{19}F, ^{13}C NMR spectra were recorded using a Bruker AVIII 400 or AVIII 600 MHz NMR spectrometer. ^1H NMR and ^{13}C NMR chemical shifts are reported in parts per million and referenced with respect to $CDCl_3$ (^1H: residual $CHCl_3$ at δ 7.26, ^{13}C: $CDCl_3$ triplet at δ 77.16) or DMSO-d6 (^1H: residual DMSO quintet at 2.50, ^{13}C: DMSO-d6 sept at 39.51). ^1H NMR data are reported as chemical shifts (δ ppm), multiplicity (s = singlet, bs = broad singlet, d = doublet, t = triplet, q = quartet, quint = quintet, sept = septet, m = multiplet, app t = apparent triplet, app q = apparent quartet, app qd = apparent quartet of doublets), coupling constant (Hz), relative integral. ^{19}F NMR data are reported as chemical shifts (δ ppm). High resolution mass spectra were obtained using Bruker MicrOTOF (ESI). IR spectra were recorded using a Thermo Fisher Nicolet iS10 FT-IR and are reported in terms of frequency of absorption (cm^{-1}). Melting points were determined using a Laboratory Devices MEL-TEMP II.

Photochemical reactions were performed by placing reaction vessels approximately 5 cm away from two 23W TCP Model EDXO-23 compact fluorescent lightbulbs (CFL, 0.380 A, 1450 lumens). Reaction temperature was maintained at approximately 23 °C by with two fans (**Figure S1**).

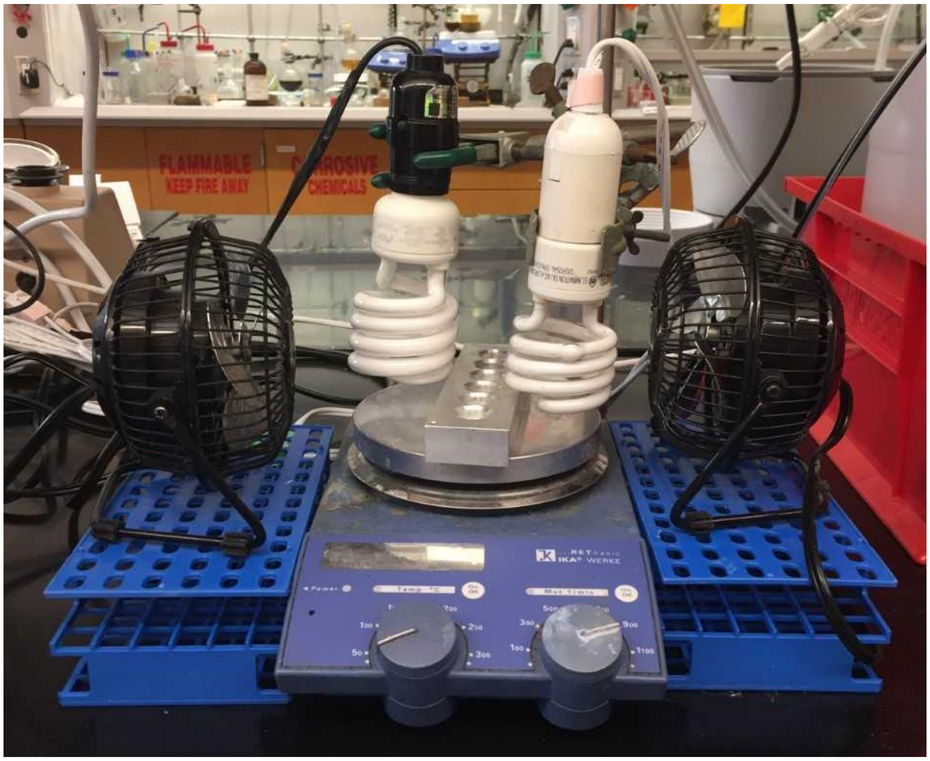

Figure S1. Photochemistry reaction set-up with two 23W CFLs and fans

II. General Procedures

General Procedure to Prepare Benzimidates (GP1)

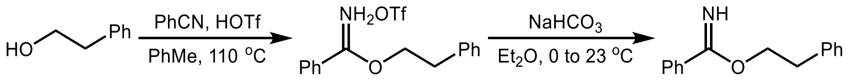

To address challenges with benzimidate synthesis, a new method was developed (see **Section III** for further discussion)

To a 4-dram vial containing a stir bar was added alcohol (1 equiv.), nitrile (1.1 equiv.), PhMe (0.5 M), and triflic acid (1.2 equiv.). The solution was heated to 110 °C and stirred. After 24 h the solution was cooled to room temperature, and then stored at -15 °C until crystallization of the hydrotriflate salt was observed. The salt was then isolated via filtration and washed with cold hexanes and Et_2O; residual solvent was removed under vacuum. The salt was suspended in Et_2O (0.1 M), and $NaHCO_3$ (sat. aqueous) was added dropwise until the dissolution of the salt observed (typically ~5 minutes). The aqueous phase was extracted with CH_2Cl_2, and the combined organic phases were dried over Na_2SO_4, concentrated under vacuum, and then used as is, or purified via column chromatography (silica gel treated with 1% Et_3N/hexanes to avoid hydrolysis).

General Procedure for Transimidation (GP2)

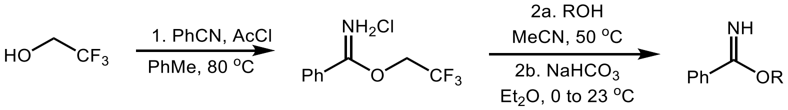

Step 1: To a pressure tube equipped with a stir bar was added nitrile (1 equiv.), trifluoroethanol (12 equiv.), and acetyl chloride (8 equiv.). The solution was heated to 80 °C and stirred. After 48 h the reaction was cooled to room temperature and carefully vented (Note: HCl gas is formed as a by-product, see below for additional instructions on safe handling), which immediately induced precipitation of the benzimidate hydrochloride salt. The benzimidate salt was collected via filtration with cold hexanes.

Caution: The pressure tube reactions were vented by bubbling through a solution of saturated $NaHCO_3$ to neutralize the by-product HCl gas. Even at room temperature, venting may be violent.

Step 2: To a 2-dram vial equipped with a stir bar was added trifluoroethyl benzimidate hydrochloride salt (1 equiv.), alcohol (1 equiv.), and MeCN (0.16 M). The reaction was heated to 50 °C and stirred. Reaction progress was monitored by consumption of starting trifluoroethyl benzimidate via crude ^{1}H NMR. Upon completion, the solution was concentrated and the resulting crude solid was suspended in dry Et_2O and subjected to the free-base protocol from **GP1**. The crude reaction mixture was then purified via column chromatography (silica gel treated with 1% Et_3N in hexanes to avoid hydrolysis).

Note: Transimidation is time-sensitive. Prolonged reaction times lead to decomposition.

General Procedure for Tandem Oxidation (GP3)

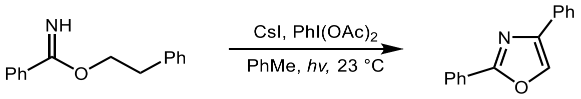

To a 2-dram vial containing a stir bar were added: imidate (0.2 mmol, 1 equiv.), CsI (0.6 mmol, 3 equiv.), and $PhI(OAc)_2$ (0.6 mmol, 3 equiv.). This was followed by evacuation and backfilling of the headspace with N_2, three times. Degassed PhMe (0.1 M, degassing method described below) was then added, and the solution was stirred under visible irradiation (2 x 23W CFL bulbs) with two fans maintaining ambient temperature (23 °C vs. 35 ºC without). Reaction progress was monitored by TLC. Upon completion, the crude reaction was quenched with 10% aq. $Na_2S_2O_3$ and extracted with CH_2Cl_2. The combined organic layers were dried over Na_2SO_4, concentrated, and then purified via column chromatography (silica gel with ethyl acetate and hexanes).

Note: Reaction solvents were degassed using three freeze-pump-thaw cycles prior to use. CsI and $PhI(OAc)_2$ were ground with a mortar and pestle and dried under high vacuum prior to usage. This both increased yields and improved reproducibility.

III. Imidate Synthesis Optimization

To improve the efficiency, handling, and safety of our benzimidate synthesis, we developed a triflic acid variant of the Pinner reaction.[1] To activate benzonitrile (pK_a of conjugate acid = -10),[2] we used triflic acid (pK_a = -14) as opposed to HCl (pK_a = -8). Triflic acid also has the significant advantage of being a liquid, preventing the need to use a sealed pressure tube or venting of gaseous species upon work-up.

2-phenylethan-1-ol was subjected to **GP1** under modified conditions listed below. Upon completion, the crude mixture was concentrated and quantified via ^{1}H NMR using 1 equiv of 1,2-dichloroethane or 1,2-dibromomethane as an internal standard.

Table S1. Optimization of Triflic Acid Pinner Reaction

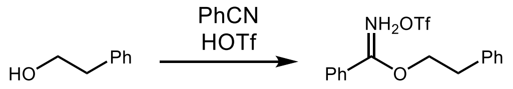

Solvent	T (°C)	Alcohol (eq)	PhCN (eq)	HOTf (eq)	Time (h)	Yield
CH_2Cl_2	23	2	1	1	17	6%
CH_2Cl_2	23	1	1	2	17	12%
CH_2Cl_2	50	1	1	1	17	58%
DCE	80	1	1	1	21	75%
DCE	80	1	2	1.2	24	69%*
DCE	80	2	1	1.2	24	73%*
**PhMe**	**110**	**1**	**1**	**1.2**	**24**	**70%***

*indicates isolated yield of the hydrotriflate salt **S2**

The reaction efficiency was found to scale well, from 1.0 mmol (0.12 g) to 24.0 mmol (2.93 g, last entry of **Table S1**). A variety of Lewis acids were tried unsuccessfully in the place of HOTf: $BF_3{\cdot}Et_2O$, $CuBr_2$, $CuCl_2$, $Cu(OAc)_2$, $Cu(acac)_2$, $Cu(tfac)_2$, $AlMe_3$, $AlCl_3$, $AlBr_3$, $AuCl_3$, $Hf(OTf)_4$; all resulting in no formation of imidate.

Notes: The hydrotriflate salts typically precipitate out upon cooling to room temperature, which can be facilitated with the addition of diethyl ether and hexanes, and refrigeration. Isolation is carried out via filtration and, with sufficient solvent washes, the resulting hydrotriflate salt is more stable for storage compared to its free-based form.

The salt was suspended in Et_2O (0.1 M), and $NaHCO_3$ (sat. aqueous) was added dropwise until the dissolution of the salt observed (typically ~5 minutes). If the initial salt was washed carefully during the prior filtration step, the resultant free-based imidate was often pure enough to use without additional purification (*i.e.* no column chromatography required).

When additional purification was needed, the silica gel was deactivated with 1% NEt_3 in hexanes, as hydrolysis of the benzimidates to their corresponding esters was often observed. For particularly sensitive compounds, shorter lengths of silica and quicker elution times were necessary to prevent hydrolysis. The benzimidates were typically stored neat in a refrigerator.

---

[1] Roger, R.; Neilson, D. G. *Chem. Rev.* **1961**, *61*, 179–211.
[2] Deno, C. N.; Gaugler, R. W.; Wisotsky, M. J. *J. Org. Chem.* **1966**, 31, 1967–1968.

Decomposition of the free-based imidate to esters and amides was observed after a few months, while the salt was stable indefinitely.

In the course of substrate exploration, we observed that secondary or benzylic alcohols afforded amides via cationic rearrangement, but primary alcohols (including phenol) and thiols formed imidate salts with varying efficiency (**Figure S2**).

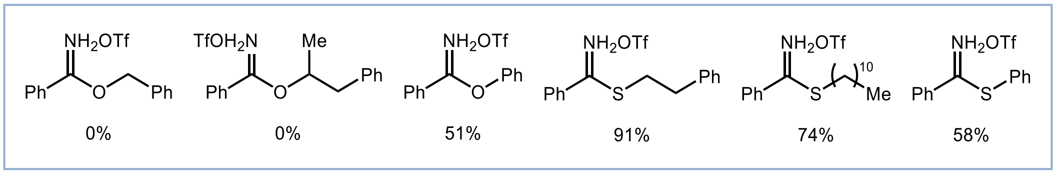

Figure S2. Summary of alcohol and thiol compatibilities under Triflic Acid Pinner conditions

IV. Substrate Synthesis

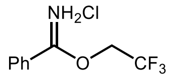

2,2,2-trifluoroethyl benzimidate hydrochloride salt (S1)

2,2,2-Trifluoroethanol (16.8 mL, 222 mmol) and benzonitrile (2 mL, 19.4 mmol) were subjected to **GP2 Step 1.** After filtration, the benzimidate hydrochloride salt **S1** was isolated (3.4 g, 74%) as a white solid. Characterization data is consistent with reported literature data.[3]

^{1}H NMR (400 MHz, $CDCl_3$): δ = 13.48 – 11.96 (bs, 2H), 8.42 – 8.35 (d, J = 7.5 Hz, 2H), 7.83 – 7.75 (t, J = 7.5 Hz, 1H), 7.67 – 7.58 (t, *J* = 7.9 Hz, 2H), 5.38 – 5.28 (q, $^{3}J_{HF}$ = 7.5 Hz, 2H).
^{19}F NMR (376 MHz, $CDCl_3$): δ = –73.3.

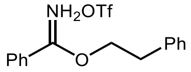

Phenethyl benzimidate hydrotriflate salt (S2)

2-phenylethan-1-ol (1.2 g, 9.7 mmol) was subjected to **GP1**, with the following modifications: dry DCE (20 mL) instead of PhMe, and a reaction temperature of 80 °C instead of 110 °C. After filtration, the benzimidate hydrotriflate salt **S2** was isolated (2.0 g, 56%) as a white solid.

^{1}H NMR (400 MHz, $CDCl_3$): δ = 10.69 (bs, 1H), 10.33 (bs, 1H), 7.97 (d, *J* = 8.4 Hz, 2H), 7.73 (t, *J* = 7.5 Hz, 1H), 7.55 (t, *J* = 7.3 Hz, 2H), 7.40 – 7.28 (m, 5H), 4.92 (t, *J* = 6.2 Hz, 2H), 3.27 (t, *J* = 6.2 Hz, 2H).
^{13}C NMR (100 MHz, DMSO-d6): δ = 171.3, 136.6, 135.5, 129.3, 129.1, 128.8, 128.6, 128.2, 127.4, 126.9, 125.9, 73.2, 33.5.
^{19}F NMR (376 MHz, $CDCl_3$): δ = –78.5.
HRMS (ESI-TOF) *m/z*: calc'd for $C_{15}H_{16}NO^+$ $[M+H]^+$ 226.1226, found 226.1232.
IR (film) (cm^{-1}): 3065, 2360, 2341, 1653, 1602, 1574, 1506, 1455, 1437, 1369, 1241, 1224, 1163, 1106, 1027, 966, 936.
MP: 118 – 119 °C.

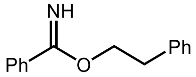

Phenethyl benzimidate (1)

Benzimidate hydrotriflate salt **S2** (1.0 g, 2.7 mmol) was free-based according to **GP1**. Purification via column chromatography (silica gel, 1% ethyl acetate/hexanes with 1% Et_3N to 7.5% ethyl acetate/hexanes) yielded benzimidate **1** (0.59 g, 98%) as a clear oil. Characterization data is consistent with reported literature data.[3]

R_f: 0.46 (30% Ethyl acetate/hexanes)
^{1}H NMR (400 MHz, $CDCl_3$): δ = 7.81 (bs, 1H), 7.67 (bs, 2H), 7.49 – 7.37 (m, 3H), 7.35 – 7.29 (m, 4H), 7.25 – 7.20 (m, 1H), 4.53 (bs, 2H), 3.13 (t, *J* = 6.9 Hz, 2H).

---

[3] Wappes, E. A.; Nakafuku, K. M.; Nagib, D. A. *J. Am. Chem. Soc.*, **2017**, *139*, 10204 – 10207.

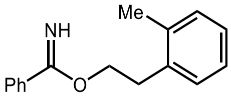

2-methylphenethyl benzimidate (S4)

2-(*o*-tolyl)ethan-1-ol (0.14 g, 1.0 mmol) was subjected to **GP2,** with acidification after transimidation to facilitate removal of unconsumed alcohol (see below). Upon completion, the free-based crude was concentrated, dissolved in dry Et_2O, and acidified with 2M HCl in Et_2O. Upon precipitation, the hydrochloride salt was isolated via filtration, and washed with cold hexanes and Et_2O, yielding the benzimidate salt (0.17 g, 63%) as a white solid. The benzimidate salt was free-based according to **GP1**; no purification was needed, yielding benzimidate **S4** (0.14 g, 98%) as a clear oil.

R_f: 0.38 (30% Ethyl acetate/hexanes)
^{1}H NMR (600 MHz, $CDCl_3$): δ = 7.76 (bs, 1H), 7.71 (d, *J* = 7.4 Hz, 2H), 7.47 – 7.44 (m, 1H), 7.42 – 7.39 (m, 2H), 7.28 – 7.26 (m, 1H), 7.19 – 7.14 (m, 3H), 4.49 (t, *J* = 7.0 Hz, 2H), 3.14 (t, *J* = 7.0 Hz, 2H), 2.40 (s, 3H).
^{13}C NMR (150 MHz, $CDCl_3$): δ = 168.0, 136.7, 136.6, 133.0, 131.0, 130.4, 129.7, 128.6, 126.8, 126.7, 126.1, 65.8, 32.5, 19.6.
HRMS (ESI-TOF) *m/z*: calc'd for $C_{16}H_{18}NO^+$ $[M+H]^+$ 240.1383, found 240.1388.
IR (film) (cm^{-1}): 3330, 3061, 3022, 2950, 2359, 2341, 1717, 1632, 1577, 1492, 1447, 1392, 1329, 1295, 1272, 1164, 1075, 1027, 1000, 973.

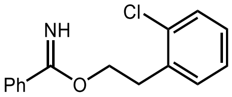

2-chlorophenethyl benzimidate (S5)

2-(2-chlorophenyl)ethan-1-ol (0.47 g, 3.0 mmol) was subjected to **GP1** without isolation of the hydrotriflate salt, as no precipitation was observed. The crude reaction was free-based according to **GP1**. Purification via column chromatography (silica gel, 1% ethyl acetate/hexanes with 1% Et_3N to 20% ethyl acetate/hexanes), yielded benzimidate **S5** (0.32 g, 41%) as a yellow oil.

R_f: 0.31 (30% Ethyl acetate/hexanes)
^{1}H NMR (600 MHz, $CDCl_3$): δ = 7.80 (bs, 1H), 7.70 (d, *J* = 6.6 Hz, 2H), 7.45 – 7.34 (m, 5H), 7.24 – 7.16 (m, 2H), 4.44 (m, 2H), 3.28, (t, *J* = 6.8 Hz, 2H).
^{13}C NMR (100 MHz, $CDCl_3$): δ = 168.1, 136.3, 134.4, 132.8, 131.3, 131.0, 129.7, 128.6, 128.1, 126.9, 126.8, 65.1, 33.0.
HRMS (ESI-TOF) *m/z*: calc'd for $C_{15}H_{15}ClNO$ $[M+H]^+$ 260.0837, found 260.0842.
IR (film) cm^{-1}: 3330, 3059, 2948, 1633.

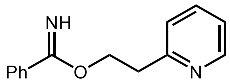

2-(pyridin-2-yl)ethyl benzimidate (S6)

2-(pyridin-2-yl)ethan-1-ol (0.79 mL, 7.0 mmol) was subjected to **GP1**, with the following modifications: additional triflic acid (1.4 mL, 15 mmol) was used, reaction temperature was 100 °C instead of 110 °C, and sufficient precipitation was achieved at 23 °C. After filtration, the

benzimidate bis hydrotriflate salt was isolated (3.7 g, 100%) as a brown solid. A portion of the benzimidate salt (1.5 g, 2.8 mmol) was free-based according to **GP1**; final purification was done via column chromatography (silica gel, 10% ethyl acetate/hexanes with 1% Et_3N to 50% ethyl acetate/hexanes with 1% Et_3N), yielding benzimidate **S6** (0.30 g, 46%) as a yellow oil.

R_f: 0.1 (50% Ethyl acetate/hexanes)
^{1}H NMR (600 MHz, $CDCl_3$): δ = 8.57 (d, *J* = 4.7 Hz, 1H), 7.81 (m, 1H), 7.65 (bs, 1H), 7.61 (td, *J* = 7.6, 1.7 Hz, 2H), 7.46 – 7.42 (m, 1H), 7.39 – 7.37 (m, 2H), 7.27 (d, *J* = 2.7 Hz, 1H) 7.14 (ddd, *J* = 7.5, 4.9, 1.0 Hz, 1H), 4.67 (bs, 2H), 3.31 (t, *J* = 6.6 Hz, 2H).
^{13}C NMR (100 MHz, $CDCl_3$): δ = 158.9, 149.6, 136.4, 132.9, 131.0, 128.6, 126.8, 123.6, 121.6, 65.3, 37.7.
HRMS (ESI-TOF) *m/z*: calc'd for $C_{14}H_{15}N_2O$ $[M+H]^+$ 227.1179, found 227.1178.
IR (film) cm^{-1}: 3283, 2922, 1633.

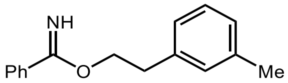

3-methylphenethyl benzimidate (S7)

2-(*m*-tolyl)ethan-1-ol (0.55 g, 4.0 mmol) was subjected to **GP1**. After filtration, the benzimidate salt was isolated (0.40 g, 25%) as a white solid. A portion of the benzimidate salt (0.29 g, 0.8 mmol) was free-based according to **GP1**; no purification was needed, yielding benzimidate **S7** (0.17 g, 100%) as an off-white solid.

R_f: 0.46 (30% Ethyl acetate/hexanes)
^{1}H NMR (400 MHz, $CDCl_3$): δ = 7.85 – 7.78 (m, 1H), 7.69 (bs, 2H), 7.49 – 7.37 (m, 3H), 7.21 (t, *J* = 7.5 Hz, 1H), 7.15 – 7.09 (m, 2H), 7.08 – 7.02 (m, 1H), 4.49 (bs, 2H), 3.09 (t, *J* = 7.1 Hz, 2H), 3.34 (s, 3H).
^{13}C NMR (100 MHz, $CDCl_3$): δ = 169.0, 138.5, 138.1, 132.9, 132.2, 131.0, 130.0, 128.8, 128.6, 128.5, 127.5, 127.3, 126.8, 126.2, 66.8, 35.2, 21.5.
HRMS (ESI-TOF) *m/z*: calc'd for $C_{16}H_{18}NO^+$ $[M+H]^+$ 240.1383, found 240.1388.
IR (film) cm^{-1}: 3325, 3025, 2950, 2359, 2341, 1670, 1632, 1577, 1489, 1447, 1392, 1329, 1295, 1164, 1078, 1027, 1000, 973.

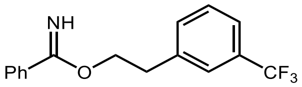

3-(trifluoromethyl)phenethyl benzimidate (S8)

2-(3-(trifluoromethyl)phenyl)ethan-1-ol (0.76 g, 4.0 mmol) and benzonitrile (0.45 mL, 4.4 mmol) were subjected to **GP1**. After filtration, the benzimidate salt was isolated (0.97 g, 55%) as a white solid. A portion of the benzimidate salt (0.50 g, 1.1 mmol) was free-based according to **GP1**; no purification was needed, yielding benzimidate **S8** (0.31 g, 96%) as an off-white oil.

R_f: 0.46 (30% Ethyl acetate/hexanes)
^{1}H NMR (400 MHz, $CDCl_3$): δ = 7.83 (bs, 1H), 7.74 – 7.61 (m, 2H), 7.59 (s, 1H), 7.54 – 7.47 (m, 2H), 7.47 – 7.37 (m, 4H), 4.63 – 4.39 (m, 2H), 3.19 (t, J = 6.6 Hz, 2H).
^{13}C NMR (150 MHz, $CDCl_3$): δ = 168.1, 139.7, 132.6, 132.5, 131.1, 129.0, 128.6, 126.7, 126.1, 123.5, 123.4, 66.2, 35.1.
^{19}F NMR (376 MHz, $CDCl_3$): δ = –62.6.

HRMS (ESI-TOF) *m/z*: calc'd for $C_{16}H_{15}F_3NO^+$ $[M+H]^+$ 294.1100, found 294.1093.
IR (film) (cm^{-1}): 3334, 3061, 2953, 2359, 1633, 1578, 1492, 1470, 1448, 1392, 1330, 1321, 1197, 1160, 1118, 1071, 1028, 1001, 781, 693.

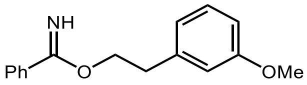

3-methoxyphenethyl benzimidate (S9)

2-(3-methoxyphenyl)ethan-1-ol (0.15 g, 1.0 mmol) was subjected to **GP2,** with acidification after transimidation to facilitate removal of unconsumed alcohol (see below). Upon completion, the free-based crude was concentrated, dissolved in dry Et_2O, and acidified with 2M HCl in Et_2O. Upon precipitation, the hydrochloride salt was isolated via filtration, and washed with cold hexanes and Et_2O, yielding the benzimidate salt (0.16 g, 66%) as a white solid. The benzimidate salt was free-based according to **GP1**; no purification was needed, yielding benzimidate **S9** (0.14 g, 98%) as a clear oil.

R$_f$: 0.46 (30% Ethyl acetate/hexanes)
^{1}H NMR (600 MHz, CDCl$_3$): δ = 7.76 (bs, 1H), 7.70 (d, *J* = 7.3 Hz, 2H), 7.47 – 7.44 (m, 1H), 7.42 – 7.39 (m, 2H), 7.24 (t, *J* = 7.9 Hz, 1H), 6.91 (d, *J* = 7.5 Hz, 1H), 6.87 – 6.86 (m, 1H), 6.79 (dd, *J* = 8.2, 2.5 Hz, 1H), 4.51 (t, *J* = 6.5 Hz, 2H), 3.80 (s, 3H), 3.11 (t, *J* = 6.9 Hz, 2H).
^{13}C NMR (150 MHz, CDCl$_3$): δ = 167.8, 159.8, 140.3, 132.9, 131.0, 129.5, 128.6, 126.8, 121.5, 114.8, 112.1, 66.6, 55.3, 35.4.
HRMS (ESI-TOF) *m/z*: calc'd for $C_{16}H_{18}NO_2^+$ $[M+H]^+$ 256.1332, found 256.1329.
IR (film) (cm^{-1}): 3330, 2952, 2834, 2359, 2341, 1717, 1633, 1601, 1578, 1487, 1448, 1392, 1328, 1294, 1257, 1164, 1151, 1077, 1057, 1041, 1028, 995, 975, 925.

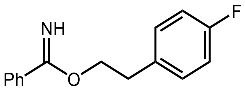

4-fluorophenethyl benzimidate (S10)

2-(4-fluorophenyl)ethan-1-ol (0.50 mL, 4.0 mmol) was subjected to **GP1**. After filtration, the benzimidate salt was isolated (1.4 g, 91%) as a white solid. A portion of the benzimidate salt (0.89 g, 2.3 mmol) was free-based according to **GP1**. Purification via column chromatography (silica gel, 10% ethyl acetate/hexanes with 1% Et_3N to 30% ethyl acetate/hexanes) yielded benzimidate **S10** (0.23 g, 41%) as a white solid.

R$_f$: 0.23 (30% Ethyl acetate/hexanes)
^{1}H NMR (400 MHz, CDCl$_3$): δ = 7.81 (bs, 1H), 7.68 (d, *J* = 7.5 Hz, 2H), 7.48 – 7.39 (m, 3H), 7.29 – 7.24 (m, 2H), 7.03 – 6.97 (m, 2H), 4.19 (bs, 2H), 3.16 (t, *J* = 6.8 Hz, 1H).
^{13}C NMR (100 MHz, CDCl$_3$): δ = 167.9, 161.8 (d, $^1J_{C\text{-}F}$ = 243.9 Hz), 134.4 (d, $^4J_{C\text{-}F}$ = 2.7 Hz), 132.8, 131.0, 130.5 (d, $^3J_{C\text{-}F}$ = 8.0 Hz), 128.6, 126.7, 115.3 (d, $^2J_{C\text{-}F}$ = 21.5 Hz), 66.6, 34.5.
^{19}F NMR (376 MHz, CDCl$_3$): δ = –118.1.
HRMS (ESI-TOF) *m/z*: calc'd for $C_{15}H_{15}FNO^+$ $[M+H]^+$ 244.1132, found 244.1137.
IR (film) cm^{-1}: 3335, 3066, 1629
MP: 53 – 54 °C.

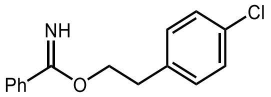

4-chlorophenethyl benzimidate (S11)

2-(4-chlorophenyl)ethan-1-ol (0.62 g, 4.0 mmol) was subjected to **GP1**. After filtration, the benzimidate salt was isolated (1.4 g, 84%) as a white solid. A portion of the benzimidate salt (0.26 g, 0.6 mmol) was free-based according to **GP1**; no purification was needed, yielding benzimidate **S11** (0.16 g, 100%) as an off-white solid.

^{1}H NMR (400 MHz, $CDCl_3$): δ = 7.71 – 7.62 (m, 2H), 7.49 – 7.37 (m, 2H), 7.32 – 7.26 (m, 2H), 7.25 – 7.20 (m, 2H), 4.49 (t, *J* = 6.7 Hz, 2H), 3.09 (t, *J* = 5.0 Hz, 2H).
^{13}C NMR (100 MHz, $CDCl_3$): δ = 167.9, 137.2, 132.7, 133.4, 131.1, 130.5, 128.7, 128.6, 126.7, 63.4, 34.7.
HRMS (ESI-TOF) *m/z*: calc'd for $C_{15}H_{15}ClNO^+$ $[M+H]^+$ 260.0837, found 260.0842.
IR (film) (cm^{-1}): 3332, 2964, 2923, 2360, 2341, 1627, 1576, 1490, 1475, 1410, 1396, 1339, 1186, 1162, 1107, 1079, 1060, 1026, 1014, 974, 922.

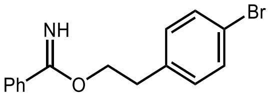

4-bromophenethyl benzimidate (S12)

2-(4-bromophenyl)ethan-1-ol (2.4 g, 12.0 mmol) was subjected to **GP1**, with the following modifications: dry DCE (20 mL) instead of PhMe, and a reaction temperature of 80 °C instead of 110 °C. After filtration, the benzimidate salt was isolated (2.9 g, 53%) as a white solid. A portion of the benzimidate salt (2.2 g, 4.9 mmol) was free-based according to **GP1**; no purification was needed, yielding benzimidate **S12** (1.3 g, 85%) as an off-white solid.

^{1}H NMR (400 MHz, $CDCl_3$): δ = 7.82 (bs, 1H), 7.71 – 7.58 (m, 2H), 7.49 – 7.36 (m, 5H), 7.21 – 7.16 (m, 2H), 4.51 (t, *J* = 6.3 Hz, 2H), 3.08 (t, *J* = 6.7 Hz, 2H).
^{13}C NMR (100 MHz, $CDCl_3$): δ = 168.2, 137.8, 132.7, 131.6, 131.1, 130.9, 128.6, 126.7, 120.4, 66.4, 34.7.
HRMS (ESI-TOF) *m/z*: calc'd for $C_{15}H_{15}BrNO^+$ $[M+H]^+$ 304.0332, found 304.0326.
IR (film) (cm^{-1}): 3331, 3060, 2965, 2896, 2866, 2359, 2341, 1627, 1575, 1485, 1457, 1407, 1394, 1334, 1300, 1292, 1185, 1162, 1106, 1082, 1069, 1059, 1026, 1010, 1001, 973, 921.
MP: 83 – 84 °C.

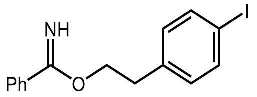

4-iodophenethyl benzimidate (S13)

2-(4-iodophenyl)ethan-1-ol (0.99 g, 4.0 mmol) was subjected to **GP1**. After filtration, the benzimidate salt was isolated (1.4 g, 70%) as a white solid. A portion of the benzimidate salt (0.57 g, 1.1 mmol) was free-based according to **GP1**; no purification was needed, yielding benzimidate **S13** (0.40 g, 100%) as an off-white solid.

^{1}H NMR (400 MHz, $CDCl_3$): δ = 7.82 (bs, 1H), 7.72 – 7.60 (m, 4H), 7.50 – 7.37 (m, 3H), 7.09 – 7.02 (m, 2H), 4.60 – 4.38 (m, 2H), 3.07 (t, J = 6.7 Hz, 2H).
^{13}C NMR (150 MHz, $CDCl_3$): δ = 168.1, 138.4, 137.6, 132.7, 131.2, 131.0, 128.6, 126.7, 91.8, 66.3, 34.8.
HRMS (ESI-TOF) *m/z*: calc'd for $C_{15}H_{15}INO$ $[M+H]^+$ 352.0193, found 352.0176.
IR (film) (cm^{-1}): 3058, 2950, 2888, 2362, 2342, 1633, 1577, 1484, 1447, 1397, 1330, 1297, 1183, 1165, 1080, 1062, 1028, 1000, 976.
MP: 79 – 80 °C.

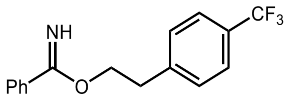

4-(trifluoromethyl)phenethyl benzimidate (S14)

2-(4-(trifluoromethyl)phenyl)ethan-1-ol (0.76 g, 4.0 mmol) was subjected to **GP1**. After filtration, the benzimidate salt was isolated (1.1 g, 62%) as a white solid. A portion of the benzimidate salt (0.72 g, 1.6 mmol) was free-based according to **GP1**; no purification was needed, yielding benzimidate **S14** (0.47 g, 99%) as a white solid.

^{1}H NMR (400 MHz, $CDCl_3$): δ = 7.82 (bs, 1H), 7.72 – 7.56 (m, 2H), 7.50 – 7.36 (m, 5H), 7.22 – 7.15 (m, 2H), 4.51 (t, J = 6.3 Hz, 2H), 3.08 (t, J = 6.7 Hz, 2H).
^{13}C NMR (100 MHz, $CDCl_3$): δ = 168.0, 143.0, 132.6, 131.1, 129.4, 129.0 (q, $^{2}J_{CF}$ = 33.0 Hz), 128.7, 126.7, 125.5 (q, $^{3}J_{CF}$ = 3.7 Hz), 124.43 (q, $^{1}J_{CF}$ = 272.0 Hz), 66.1, 35.1.
^{19}F NMR (376 MHz, $CDCl_3$): δ = –62.4.
HRMS (ESI-TOF) *m/z*: calc'd for $C_{16}H_{15}F_3NO^+$ $[M+H]^+$ 294.1100, found 294.1088.
IR (film) (cm^{-1}): 3337, 2955, 2894, 2359, 2341, 1628, 1617, 1576, 1496, 1448, 1419, 1397, 1324, 1187, 1168, 1153, 1124, 1110, 1086, 1063, 1027, 1017, 1000, 974, 957, 924.
MP: 83 – 84 °C.

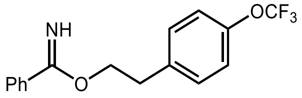

4-(trifluoromethoxy)phenethyl benzimidate (S15)

2-(4-(trifluoromethoxy)phenyl)ethan-1-ol (0.94 g, 4.5 mmol) was subjected to **GP1**. After filtration, the benzimidate salt was isolated (1.4 g, 68%) as a white solid. A portion of the benzimidate salt (0.76 g, 1.7 mmol) was free-based according to **GP1**; no purification was needed, yielding benzimidate **S15** (0.52 g, 99%) as an off-white solid.

^{1}H NMR (400 MHz, $CDCl_3$): δ = 7.83 (bs, 1H), 7.73 – 7.57 (m, 2H), 7.50 – 7.37 (m, 3H), 7.37 – 7.29 (m, 2H), 7.16 (d, J = 7.9 Hz, 2H), 4.62 – 4.42 (m, 2H), 3.13 (t, J = 6.7 Hz, 2H).
^{13}C NMR (150 MHz, $CDCl_3$): δ = 168.2, 148.0, 137.6, 132.7, 131.1, 130.4, 128.6, 126.7, 121.1, 120.6 (q, $^{1}J_{CF}$ = 256.6 Hz), 66.4, 34.6.
^{19}F NMR (376 MHz, $CDCl_3$): δ = –57.9.
HRMS (ESI-TOF) *m/z*: calc'd for $C_{16}H_{15}F_3NO_2^+$ $[M+H]^+$ 310.1049, found 310.1044.
IR (film) (cm^{-1}): 3332, 2966, 2896, 2359, 2342, 1627, 1577, 1507, 1474, 1448, 1399, 1339, 1259, 1211, 1195, 1152, 1108, 1080, 1058, 1027, 1018, 1001, 974, 920.
MP: 61 – 62 °C.

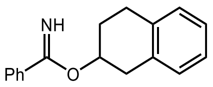

1,2,3,4-tetrahydronaphthalen-2-yl benzimidate (S16)

1,2,3,4-tetrahydronaphthalen-2-ol (0.30 g, 2.0 mmol) was subjected to **GP2 Step 2,** with the following modifications: a reaction temperature of 60 °C instead of 50 °C, and 2 equivalents of Trifluoroethanol benzimidate was used. Final purification was done via column chromatography (silica gel, 1% ethyl acetate/hexanes with 1% Et_3N to 20% ethyl acetate/hexanes with 1% Et_3N), yielding the imidate **S16** (0.27 g, 52%) as a clear oil.

Note: The transimidation is temperature sensitive; at 50 °C the alcohol is not consumed, and at 80 °C, the imidate decomposes into benzamide. Multiple columns may be needed to obtain pure benzimidate.

R_f: 0.26 (20% Ethyl acetate/hexanes)
^{1}H NMR (400 MHz, $CDCl_3$): δ = 7.83 (bs, 1H), 7.70 (d, *J* = 7.2 Hz, 2H), 7.46 – 7.38 (m, 3H), 7.15 – 7.09 (m, 4H), 5.48 (bs, 1H), 3.28 (dd, *J* = 16.8, 5.1 Hz, 1H), 3.16 – 3.00 (m, 2H), 2.91 (td, *J* = 16.7, 6.7 Hz, 1H), 2.25 – 2.09 (m, 2H).
^{13}C NMR (100 MHz, $CDCl_3$): δ =167.2, 136.0, 134.4, 134.3, 130.9, 129.6, 128.7, 128.6, 126.8, 126.0, 126.0, 70.6, 34.6, 27.8, 26.7.
HRMS (ESI-TOF) *m/z*: calc'd for $C_{17}H_{18}NO$ $[M+H]^+$ 252.1383, found 252.1378.
IR (film) cm^{-1}: 3328, 3018, 1630.

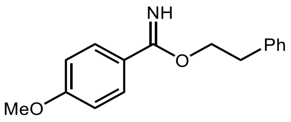

phenethyl 4-methoxybenzimidate (**S17**)

4-methoxybenzonitrile (1 g, 7.5 mmol) was subjected to **GP1**. After filtration, the crude benzimidate salt was isolated as a white solid. A portion of the benzimidate salt (1.0 g, 2.5 mmol) was free-based according to **GP1**. Purification via column chromatography (silica gel, 10% ethyl acetate/hexanes with 1% Et_3N to 25% ethyl acetate/hexanes) yielded benzimidate **S17** (0.52 g, 82%) as a white solid.

R_f: 0.09 (20% Ethyl acetate/hexanes)
^{1}H NMR (400 MHz, $CDCl_3$): δ = 7.67 (bs, 1H), 7.66 (d, *J* = 8.7 Hz, 2H), 7.35 – 7.30 (m, 4H), 7.26 – 7.22 (m, 1H), 6.92 – 6.88 (m, 2H), 4.49 (t, *J* = 6.8 Hz, 2H), 3.84 (s, 3H), 3.13 (t, *J* = 6.8 Hz, 2H).
^{13}C NMR (100 MHz, $CDCl_3$): δ = 167.4, 161.8, 138.7, 129.1, 128.52, 128.46, 126.5, 125.3, 113.8, 66.5, 55.5, 35.3.
HRMS (ESI-TOF) m/z: calc'd for $C_{16}H_{18}NO_2$ $[M+H]^+$ expected 256.1338, found 256.1319.
IR (film) cm^{-1}: 3276, 2996, 2955, 2922, 2835, 1624, 1604, 1509.

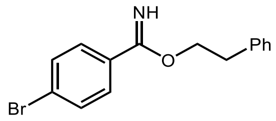

phenethyl 4-bromobenzimidate (**S18**)

4-bromobenzonitrile was subjected to **GP2**, yielding the corresponding TFE-benzimidate (1.43 g, 82%) as a white solid. A portion of the TFE-benzimidate salt (2 reactions at 0.2 g scale) was freebased according to **GP2 Step 2**. Purification via column chromatography (silica gel, 10% ethyl acetate/hexanes with 1% Et_3N) yielded benzimidate **S18** (0.25 g, 65%) as a white solid.

R_f: 0.09 (20% Ethyl acetate/hexanes)
^{1}H NMR (400 MHz, $CDCl_3$): δ = 7.80 (bs, 1H), 7.55 – 7.50 (m, 4H), 7.34 – 7.23 (m, 5H), 4.52 (t, *J* = 6.8 Hz, 2H), 3.11 (t, *J* = 6.8 Hz, 2H).
^{13}C NMR (100 MHz, $CDCl_3$): δ = 167.1, 138.5, 131.8, 129.1, 128.6, 128.4, 126.6, 125.6, 66.9, 35.2.
HRMS (ESI-TOF) m/z: calc'd for $C_{15}H_{15}BrNO$ $[M+H]^+$ expected 304.0337, found 304.0315.
IR (film) cm^{-1}: 3336, 3085, 3032, 2969, 2951, 2931, 2891, 2861, 1639, 1586.
MP: 57 – 59 °C.

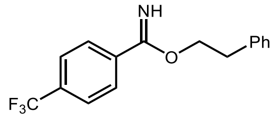

phenethyl 4-(trifluoromethyl)benzimidate (**S19**)

4-trifluoromethylbenzonitrile (0.5 mL, 0.64 mL, 3.7 mmol) was subjected to **GP1**. After filtration, the crude benzimidate salt was isolated. The benzimidate salt was free-based according to **GP1**. Purification via column chromatography (silica gel, 5% ethyl acetate/hexanes with 1% Et_3N), yielded benzimidate **S19** (0.65 g, 59%) as a white solid.

R_f: 0.22 (20% Ethyl acetate/hexanes)
^{1}H NMR (400 MHz, $CDCl_3$): δ = 7.94 (bs, 1H), 7.78 (bs, 2H), 7.66 (d, *J* = 8.3 Hz, 2H), 7.36 – 7.29 (m, 4H), 7.27 – 7.23 (m, 1H), 4.54 (bs, 2H), 3.14 (t, *J* = 6.7 Hz, 2H).
^{13}C NMR (150 MHz, $CDCl_3$): δ = 166.9, 138.5, 136.1, 132.7 (^2J = 32.8 Hz), 129.0, 128.5, 127.1, 126.6, 125.5, 122.9, 67.1, 35.1.
HRMS (ESI-TOF) m/z: calc'd for $C_{16}H_{15}F_3NO$ $[M+H]^+$ expected 294.1106, found 294.1099.
IR (film) cm^{-1}: 3335, 3059, 3026, 2962, 2893, 1638, 1577, 1541.
MP: 62.5 – 63.5 °C.

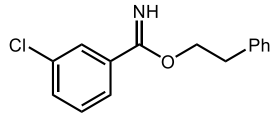

phenethyl 3-chlorobenzimidate (**S20**)

3-chlorobenzonitrile was subjected to **GP2**, yielding the corresponding TFE-benzimidate (0.54 g, 54%) as a white solid. A portion of the TFE-benzimidate salt (0.2 g) was subjected to **GP2 Step**

2. Purification via column chromatography (silica gel, 10% ethyl acetate/hexanes with 1% Et_3N) yielded benzimidate **S20** (0.18 g, 96%) as a white solid.

R_f: 0.15 (20% Ethyl acetate/hexanes)
^{1}H NMR (400 MHz, $CDCl_3$): δ = 7.83 (bs, 1H), 7.63 (bs, 1H), 7.53 – 7.51 (m, 1H), 7.43 (ddd, *J* = 8.0, 2.1, 1.1 Hz, 1H), 7.36 – 7.29 (m, 5H), 7.26 – 7.24 (m, 1H), 4.53 (t, *J* = 6.9 Hz, 2H), 3.12 (t, *J* = 6.8 Hz, 2H).
^{13}C NMR (100 MHz, $CDCl_3$): δ = 166.7, 138.4, 134.7, 131.0, 129.8, 129.1, 128.6, 128.4, 127.2, 126.6, 124.9, 67.0, 35.2.
HRMS (ESI-TOF) m/z: calc'd for $C_{15}H_{15}ClNO$ $[M+H]^+$ expected 260.0842, found 260.0835.
IR (film) cm^{-1}: 3338, 3062, 3029, 2958, 2895, 2859, 1632, 1595, 1566.
MP: 35 – 36 °C.

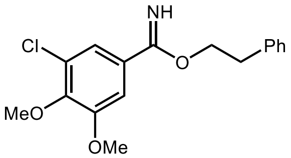

phenethyl 3-chloro-4,5-dimethoxybenzimidate (S21)

3,4-dichloro-5-methoxybenzonitrile (2.0 g, 10.0 mmol) was subjected to GP1, with the following modifications: dry DCE (18 mL) instead of PhMe, and a reaction temperature of 80 °C instead of 110 °C. After filtration, the benzimidate salt was isolated (2.4 g, 57%) as a white solid. A portion of the benzimidate salt (0.52 g, 1.1 mmol) was free-based according to GP1; no purification was needed, yielding benzimidate **S21** (590 mg, 98%) as a yellow oil.

^{1}H NMR (400 MHz, $CDCl_3$): δ = 7.81 – 7.62 (m, 1H), 7.41 – 7.27 (m, 5H), 7.25 – 7.20 (m, 1H), 7.14 – 6.94 (m, 1H), 4.67 – 4.32 (m, 2H), 3.89 (s, 3H), 3.86 (s, 3H), 3.16 – 3.07 (t, *J* = 6.8 Hz, 2H).
^{13}C NMR (100 MHz, $CDCl_3$): δ =153.6, 147.7, 138.5, 129.1, 128.6, 126.6, 120.9, 120.7, 110.9, 109.6, 66.8, 60.9, 56.3, 35.2.
HRMS (ESI-TOF) *m/z*: calc'd for $C_{17}H_{19}ClNO_3^+$ $[M+H]^+$ 320.1048, found 320.1040.
IR (film) cm^{-1}: 3324, 2939, 2833, 2359, 2341, 1670, 1634, 1596, 1566, 1491, 1453, 1405, 1324, 1277, 1235, 1175, 1134, 1077, 1047, 996, 940.
MP: 33 – 34 °C.

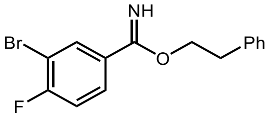

phenethyl 3-bromo-4-fluorobenzimidate (S22)

3-bromo-4-fluorobenzonitrile (2 g, 14.5 mmol) was subjected to GP2 Step 1, yielding the corresponding TFE-benzimidate (1.63 g, 67%) as a white solid. A portion of the TFE-benzimidate salt (0.17 g) was subjected to GP2 Step 2. Purification via column chromatography (silica gel, 10% ethyl acetate/hexanes with 1% Et_3N), yielded benzimidate **S22** (0.12 g, 87%) as a white solid.

R_f: 0.12 (20% Ethyl acetate/hexanes)

^{1}H NMR (400 MHz, $CDCl_3$): δ = 7.89 (bs, 1H), 7.77 (bs, 1H), 7.59 (m, 1H), 7.35 – 7.23 (m, 5H), 7.13 (t, *J* = 8.4 Hz, 1H), 4.49 (bs, 2H), 3.12 (t, *J* = 6.9 Hz, 2H).
^{13}C NMR (100 MHz, $CDCl_3$): δ = 162.0, 159.5, 138.3, 132.7, 130.5, 129.1, 128.7, 127.8, 126.7, 116.5 (d, *J* = 22.8 Hz), 109.4 (d, *J* = 22.1), 66.9, 35.2.
^{19}F NMR (376 MHz, $CDCl_3$): δ = –103.5.
HRMS (ESI-TOF) m/z: calc'd for $C_{15}H_{14}BrFNO$ $[M+H]^+$ expected 322.0243, found 322.0215.
IR (film) cm^{-1}: 3362, 3057, 3026, 2956, 2942, 2883, 1900, 1641, 1597, 1584.
MP: 58 – 58.8 °C.

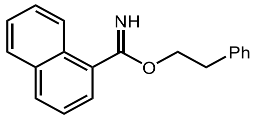

phenethyl 1-naphthimidate (S23)

1-naphthonitrile (0.5 g, 3.3 mmol) was subjected to GP1. After filtration, the crude benzimidate salt was isolated. The benzimidate salt was free-based according to GP1. Purification was done via column chromatography (silica gel, 10% ethyl acetate/hexanes with 1% Et_3N) yielding benzimidate **S23** (0.12 g, 13%) as a clear oil.

R_f: 0.34 (20% Ethyl acetate/hexanes)
^{1}H NMR (400 MHz, $CDCl_3$): δ = 7.91 – 7.85 (m, 3H), 7.62 (bs, 1H), 7.54 – 7.42 (m, 4H), 7.33 – 7.31 (m, 4H), 7.28 – 7.24 (m, 1H), 4.66 (t, *J* = 6.8 Hz, 2H), 3.16 (t, *J* = 6.8 Hz, 2H).
^{13}C NMR (100 MHz, $CDCl_3$): δ = 170.2, 138.7, 133.7, 130.3, 129.8, 129.2, 128.6, 128.5, 127.1, 126.6, 126.3, 125.7 (2C), 125.3. 125.0, 67.1, 35.3.
HRMS (ESI-TOF) m/z: calc'd for $C_{19}H_{17}NONa$ $[M+Na]^+$ expected 298.1208, found 298.1209.
IR (film) cm^{-1}: 3317, 3057, 3027, 2954, 2915, 2893, 1950, 1629.

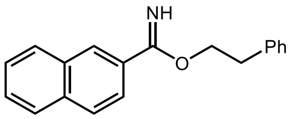

phenethyl 2-naphthimidate (S24)

2-naphthonitrile (0.5 g, 3.3 mmol) was subjected to GP1. After filtration, the crude benzimidate salt was isolated. A portion of the benzimidate salt (0.75 g, 1.8 mmol) was free-based according to GP1. Purification via column chromatography (silica gel, 10% ethyl acetate/hexanes with 1% Et_3N) yielded benzimidate **S24** (0.12 g, 25%) as a white solid with a minor ester impurity (ca. 10%).

R_f: 0.11 (20% Ethyl acetate/hexanes)
^{1}H NMR (400 MHz, $CDCl_3$): δ = 8.21 (bs, 1H), 7.91 – 7.85 (m, 3H), 7.76 (dd, *J* = 8.5, 1.6 Hz, 1H), 7.57 – 7.51 (m, 2H), 7.37 – 7.32 (m, 4H), 7.29 – 7.24 (m, 1H), 4.57 (t, *J* = 6.8 Hz, 2H), 3.19 (t, *J* = 6.9 Hz, 2H).
^{13}C NMR (100 MHz, $CDCl_3$): δ = 167.6, 138.7, 134.5, 132.9, 130.0, 129.2, 129.1, 128.6, 128.4, 127.8, 127.6, 127.3, 126.8, 126.6, 123.6, 66.8, 35.4.
HRMS (ESI-TOF) m/z: calc'd for $C_{19}H_{18}NO$ $[M+H]^+$ expected 276.1388, found 276.1374.
IR (film) cm^{-1}: 3335, 3060, 3031, 2951, 2893, 2854, 1638, 1599, 1552.
MP: 66 – 68 °C.

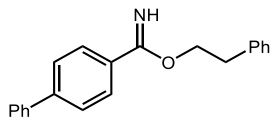

phenethyl [1,1'-biphenyl]-4-carbimidate (S25)

4-phenylbenzonitrile (1 g, 5.6 mmol) was subjected to **GP1**. After filtration, the crude benzimidate salt was isolated. A portion of the benzimidate salt (0.5 g, 1.1 mmol) was free-based according to **GP1**. Final purification via column chromatography (silica gel, 10% ethyl acetate/hexanes with 1% Et_3N), yielded benzimidate **S25** (0.12 g, 36%) as a white solid.

R_f: 0.09 (20% Ethyl acetate/hexanes)
^{1}H NMR (400 MHz, $CDCl_3$): δ = 7.85 (bs, 1H), 7.75 – 7.73 (m, 2H), 7.64 – 7.60 (m, 4H), 7.48 – 7.44 (m, 2H), 7.40 – 7.36 (m, 1H), 7.33 (d, *J* = 4.3 Hz, 4H), 7.27 – 7.23 (m, 1H) 4.56 (t, *J* = 6.2 Hz, 2H), 3.15 (t, *J* = 6.8 Hz, 2H).
^{13}C NMR (100 MHz, $CDCl_3$): δ = 167.3, 143.8, 140.2, 138.7, 129.1, 129.0, 128.6, 128.0, 127.4, 127.31, 127.29, 127.26, 126.5, 66.7, 35.3.
HRMS (ESI-TOF) m/z: calc'd for $C_{21}H_{20}NO$ $[M+H]^+$ expected 302.1545, found 302.1538.
IR (film) cm^{-1}: 3335, 3062, 3030, 2942, 1629, 1607, 1582.
MP: 116 – 118 °C.

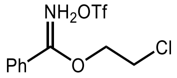

2-chloroethyl benzimidate hydrotriflate salt (S26)

2-chloroethanol (1.2 g, 15 mmol) was subjected to **GP1**. After filtration, the benzimidate salt was isolated (3.1 g, 51%) as a white solid.

Note: The benzimidate salt was found to rapidly tautomerize to the corresponding amide under free-base conditions.

^{1}H NMR (400 MHz, $CDCl_3$): δ = 10.68 (bs, 2H), 8.15 (d, *J* = 7.4 Hz, 2H), 7.78 (t, *J* = 7.5 Hz, 1H), 7.60 (t, *J* = 8.0 Hz, 2H), 4.94 (t, *J* = 4.8 Hz, 2H), 3.99 (t, *J* = 4.8 Hz, 2H).
^{13}C NMR (150 MHz, DMSO-d6): δ = 171.2, 135.7, 129.4, 129.0, 127.2, 72.5, 41.4.
^{19}F NMR (376 MHz, $CDCl_3$): δ = –78.5.
HRMS (ESI-TOF) m/z: calc'd for $C_9H_{11}ClNO$ $[M+H]^+$ expected 184.0524, found 184.0529.
IR (film) cm^{-1}: 1628, 1602, 1507, 1460, 1432, 1376, 1270, 1226, 1167, 1106, 1086, 1027, 1000, 962.
MP: 114 – 115 °C.

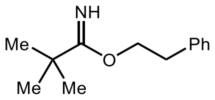

Phenethyl pivalimidate (S27)

2-phenylethan-1-ol (0.82 mL, 8.0 mmol) and pivalonitrile (0.97 mL, 8.8 mmol) were subjected to **GP1**. After filtration, the pivalimidate salt was isolated (680 g, 24%) as a white solid. A portion of the pivalimidate salt (420 mg, 1.2 mmol) was free-based according to **GP1**. Purification via column

chromatography (silica gel, 20% ethyl acetate/hexanes with 1% Et_3N), yielded pivalimidate **S27** (100 mg, 44%) as a clear oil.

Note: The phenethyl pivalimidate hydrotriflate salt took over a week to precipitate out at -15 °C. Alkyl imidates are generally more hydrolytically unstable than their corresponding benzimidates. As such, column chromatography for this substrate was done quickly, in less than 3 minutes. The crude free-based material can be used without any observable difference in reactivity.

R_f: 0.42 (20% Ethyl acetate/hexanes)
^{1}H NMR (400 MHz, $CDCl_3$): δ = 7.30 – 7.20 (m, 5H), 6.90 (Bs, 1H), 4.29 (t, *J* = 6.7 Hz, 2H), 3.00 (t, *J* = 6.7 Hz, 2H), 1.14 (s, 9H).
^{13}C NMR (100 MHz, $CDCl_3$): δ = 179.2, 138.8, 129.1, 128.4, 126.4, 66.5, 37.9, 35.3, 27.8
HRMS (ESI-TOF) *m/z*: calc'd for $C_{13}H_{20}NO$ $[M+H]^+$ 206.1539, found 206.1543.
IR (film) cm^{-1}: 3028, 2958, 2871, 1726, 1639.

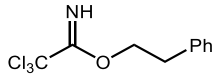

Phenethyl 2,2,2-trichloroacetimidate (S28)

Trichloroacetimidate **S28** was prepared following a literature method.[3]

To a 250 mL round bottom flask containing a stirbar, 2-phenylethan-1-ol (2.2 g, 18 mmol) and CH_2Cl_2 (180 mL) were added trichloroacetonitrile (3.6 mL, 36 mmol) and 1,8-diazabicyclo[5.4.0]undec-7-ene (0.55 mL, 4.5 mmol). The solution was stirred at 23 °C and monitored by TLC until consumption of alcohol. Upon completion, the solution was concentrated and directly loaded onto silica gel (treated with 1% Et_3N in hexanes) and purified, yielding trichloroacetimidate **S28** (4.2 g, 87%) as a light-yellow oil.

Characterization data is consistent with reported literature data.[3]

R_f: 0.66 (20% Ethyl acetate/hexanes)
^{1}H NMR (400 MHz, $CDCl_3$): δ = 8.28 (bs, 1H), 7.34 – 7.26 (m, 4H), 7.25 – 7.21 (m, 1H), 4.50 (t, *J* = 6.9 Hz, 2H), 3.09 (t, *J* = 6.9 Hz, 2H).

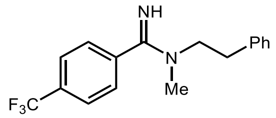

N-methyl-N-phenethyl-4-(trifluoromethyl)benzimidamide **(S29)**

Amidine **S29** was prepared following a literature method.[4]

To a 2-dram vial equipped with stirbar and N-methyl phenethylamine (0.54 g, 4.0 mmol) and PhMe (4.5 mL) at 0 °C, was added $AlMe_3$ (25% w/w in hexanes, 2.7 mL, 6.2 mmol) dropwise. The reaction was stirred and warmed to room temperature for 15 minutes. 4-trifluorobenzonitrile (1.0 g, 6.0 mmol) in PhMe (1.0 mL) was added dropwise, and then brought to 110 °C for 24 h. The reaction was quenched with H_2O, extracted with ethyl acetate, and then purified via column

[4] Chen, H.; Kaga, A.; Chiba, S. *Org. Lett.* **2014**, *16*, 6136–6139.

chromatography (silica gel with 1% Et_3N, 20% ethyl acetate/hexanes to 97% ethyl acetate/3% Et_3N) yielding amidine **S29** (0.31 g, 25%) as a yellow oil.

R_f: 0.29 (97% Ethyl acetate/triethylamine)
^{1}H NMR (400 MHz, $CDCl_3$): δ = 7.56 (d, J = 8.0 Hz, 2H), 7.30 – 7.26 (m, 2H), 7.25 – 7.20 (m, 2H), 7.16 (d, J = 7.9 Hz, 2H), 7.04 (d, J = 6.7 Hz, 2H), 3.42 (t, J = 7.0 Hz, 2H), 2.94 (s, 3H), 2.83 (t, J = 7.2 Hz, 2H).
^{13}C NMR (150 MHz, $CDCl_3$): δ = 168.1, 142.1, 140.1, 138.8, 130.9 (q, $^2J_{CF}$ = 32.7 Hz), 129.0, 128.6, 128.0, 127.1, 126.6, 126.2, 125.6 (q, $^3J_{CF}$ = 3.7 Hz), 123.9 (q, $^1J_{CF}$ = 272.7 Hz), 53.2, 36.1, 34.3.
^{19}F NMR (376 MHz, $CDCl_3$): δ = –62.8.
HRMS (ESI-TOF) *m/z*: calc'd for $C_{17}H_{18}F_3N_2^+$ [M+H]$^+$ 307.1417, found 307.1405.
IR (film) cm^{-1}: 3367, 3027, 2345, 2115, 1685, 1618, 1585, 1570, 1520, 1496, 1478, 1454, 1406, 1364, 1322, 1665, 1123, 1065, 1030.

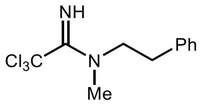

2,2,2-trichloro-*N*-methyl-*N*-phenethylacetimidamide (S30)

Amidine **S30** was prepared following a literature method.[4]

To a 250 mL round bottom flask containing a stirbar, N-methyl-phenethylamine (0.68 g, 5.0 mmol) and CH_2Cl_2 (50 mL) were added trichloroacetonitrile (1.0 mL, 10 mmol) and 1,8-diazabicyclo[5.4.0]undec-7-ene (1.5 mL, 1.0 mmol). The solution was stirred at 23 °C and monitored by TLC until consumption of amine. Upon completion, the solution was concentrated and directly loaded onto silica gel (treated with 1% Et_3N in hexanes) and purified, yielding amidine **S30** (0.8 g, 56%) as an light yellow oil.

R_f: 0.21 (30% Ethyl acetate/hexanes)
^{1}H NMR (400 MHz, $CDCl_3$): δ = 7.68 (bs, 1H), 7.34 – 7.27 (m, 2H), 7.25 – 7.19 (m, 3H), 3.79 – 3.71 (m, 2H), 3.12 (s, 3H), 3.01 – 2.93 (m, 2H).
^{13}C NMR (100 MHz, $CDCl_3$): δ = 179.2, 138.8, 129.14, 129.10, 128.4, 66.5, 37.9, 35.3, 27.8
HRMS (ESI-TOF) *m/z*: calc'd for $C_{11}H_{17}Cl_3N_2^+$ [M+H]$^+$ 279.0217, found 279.0215.
IR (film) cm^{-1}: 3315, 2922, 1709, 1619, 1573, 1522, 1488, 1449, 1426, 1373, 1328, 1310, 1258, 1168, 1071, 1029, 1008.

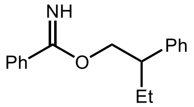

2-phenylbutyl benzimidate (34)

2-phenylbutan-1-ol (0.15 g, 1.0 mmol) was subjected to GP2. The crude reaction mixture was washed with $NaHCO_3$ and purification via column chromatography (silica gel treated with 1% Et_3N), yielded benzimidate **34** (0.034 g, 13%) as a clear oil.

R_f: 0.22 (20% Ethyl acetate/hexanes)
^{1}H NMR (600 MHz, $CDCl_3$): δ = 7.80 (bs, 1H), 7.47 – 7.44 (m, 1H), 7.42 – 7.23 (m, 10H), 4.53 – 4.40 (m, 2H), 3.10 – 3.01 (m, 1H), 2.04 – 1.90 (m, 1H), 1.83 – 1.71 (m, 1H) 0.92 (t, J = 7.4 Hz, 3H).

^{13}C NMR (100 MHz, $CDCl_3$): δ = 167.5, 141.4, 131.8, 129.8, 127.6, 127.0, 127.7, 126.7, 125.6, 66.3, 45.8, 24.5, 10.9.
HRMS (ESI-TOF) *m/z*: calc'd for $C_{17}H_{20}NO^+$ $[M+H]^+$ 254.1539, found 254.1416.

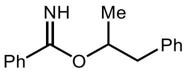

1-phenylpropan-2-yl benzimidate (38)

1-phenyl-2-propanol (0.273 g, 2.0 mmol) was combined with trifluoroethanol benzimidate (hydrochloride salt) (1.16 g, 4.8 mmol) in MeCN (8 mL) and stirred at 60° C for 16h. The solvent was evaporated, and the residue was freebased according to **GP1**. The crude product was purified via column chromatography (silica gel, 5% ethyl acetate/hexanes with 1% Et_3N to yield benzimidate **38** (0.20 g, 0.84 mmol, 42%) as a clear oil.

Note: the transimidation is temperature sensitive; at 50 °C the alcohol is not consumed, and at 80 °C, the imidate decomposes into benzamide. Multiple columns may be needed to obtain pure benzimidate.

R_f: 0.37 (20% Ethyl acetate/hexanes)
^{1}H NMR (400 MHz, $CDCl_3$): δ = 7.75 (bs, 1H), 7.68 (d, J = 7.2 Hz, 2H), 7.47 – 7.38 (m, 3H), 7.30 – 7.27 (m, 4H), 7.25 – 7.20 (m, 1H), 5.40 (bs, 1H), 3.14 (dd, J = 13.6, 6.4 Hz, 1H), 2.94 (dd, J = 13.7, 6.4 Hz, 1H), 1.37 (d, J = 6.2 Hz, 3H).
^{13}C NMR (100 MHz, $CDCl_3$): δ =167.3, 138.3, 133.5, 130.8, 129.7, 128.4, 126.8, 126.4, 72.4, 42.4, 19.3.
HRMS (ESI-TOF) m/z: calc'd for $C_{16}H_{18}NO$ $[M+H]^+$ 240.1383, found 240.1376.
IR (film) cm–1: 3026, 3018, 2926, 1630.

V. Tandem Oxidation Optimization

Solvent Effects with CsI

Benzimidate **3** (0.2 mmol) was subjected to **GP3**, with different solvents listed below. Upon completion, the crude mixture was quenched, concentrated, and quantified via ^{1}H NMR using 1 equiv of 1,2-dichloroethane as an internal standard.

Table S2. Solvent effects with CsI

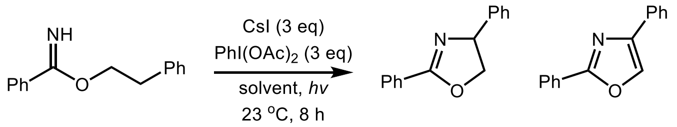

Solvent	ε	Time (h)	Imidate	Oxazoline	Oxazole
Hexanes	1.88	8	53%	28%	7%
1,4-Dioxane	2.25	8	0%	63%	24%
PhH	2.27	8	0%	32%	25%
PhMe	2.38	8	0%	63%	36%
**PhMe**	**2.38**	**24**	**0%**	**0%**	**88%**
p-Xylene	2.57	8	0%	49%	29%
Ethyl acetate	6.02	8	31%	64%	0%
THF	7.5	8	0%	91%	0%
$PhCF_3$	9.04	8	34%	50%	14%
CH_2Cl_2	9.1	8	0%	50%	45%
**CH_2Cl_2**	**9.1**	**24**	**0%**	**10%**	**64%**
MeCN	37.5	8	0%	100%	0%
DMF	38	8	0%	90%	0%

Counter-Ion Effects

Benzimidate **3** (0.2 mmol) was subjected to **GP3**, with different iodide salts listed below. Upon completion, the crude mixture was quenched, concentrated, and quantified via ^{1}H NMR using 1 equiv of 1,2-dichloroethane as an internal standard.

Table S3. Counter-ion effects with metal iodides

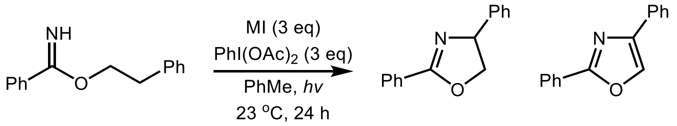

MI	M^+ r_{ion} (pm)[5]	Imidate	Oxazoline	Oxazole
LiI	90	0%	28%	63%
NaI	116	0%	10-30%	65-85%
CuI	77	90%	10%	0%
KI	152	0%	5%	76%
**CsI**	**181**	**0%**	**0%**	**96%**
^{n}Bu$_4$I	494[6]	0%	25%	55%

[5] Shannon, R.D. *Acta Crystallogr. A.* **1976,** A32, 751 – 767.
[6] Poli, I.; Eslava, S.; Cameron, P. *J. Mater. Chem. A,* **2017,** *5,* 22325 – 22333.

Conditions: imidate (0.2 mmol), MI (3 equiv), $PhI(OAc)_2$ (3 equiv), solvent (2 mL), 23 W compact fluorescent light (CFL), 23 °C, 24 h. ^{1}H NMR yields vs DCE standard.
Note: NaI affords a variable range of yields, while CsI affords both more consistent and higher yields

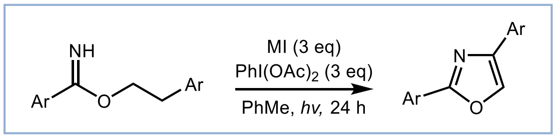

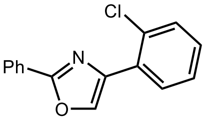

5, NaI: 27%[a]
CsI: 63%

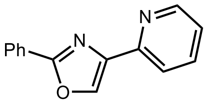

6, NaI: 25%[a]
CsI: 50%

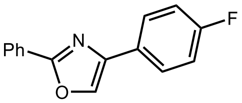

10, NaI: 61%[a]
CsI: 85%

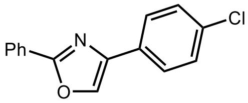

11, NaI: 62%[a]
CsI: 94%

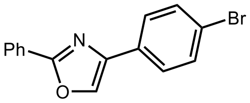

12, NaI: 62%[a]
CsI: 98%

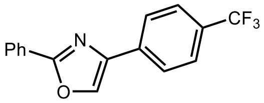

14, NaI: 71%[a]
CsI: 84%

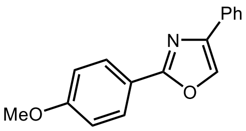

17, NaI: 78%[a]
CsI: 76%

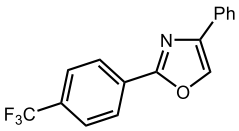

19, NaI: 66%[a]
CsI: 77%

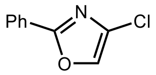

26, NaI: 40%[a]
CsI: 60%[a]

Figure S3. Summary of improved yields with CsI compared to NaI, [a] ^{1}H NMR yields with 1 equiv 1,2-dichloromethane as an internal standard

Light, Thermal, and Oxygen Controls

Benzimidate **3** was subjected to GP3, with different conditions listed below. Upon completion, the crude mixture was quenched, concentrated, and quantified via ^{1}H NMR using 1 equiv of 1,2-dichloroethane as an internal standard.

Table S4. Light and oxygen controls

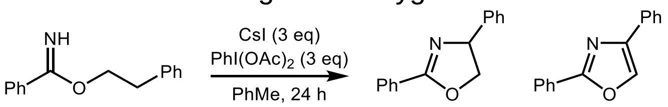

MI	Solvent	Variation	Imidate	Oxazoline	Oxazole
NaI	PhMe	No Degas	0%	9%	75%
NaI	PhMe	Freeze-Pump-Thaw; N_2	0%	12%	74%
NaI	PhMe	Freeze-Pump-Thaw; O_2	0%	14%	58%
**CsI**	**PhMe**	No change	**0%**	**0%**	**98%**
CsI	PhMe	Blue LED	0%	0%	82%
CsI	PhMe	No light, r.t.	15%	75%	12%
CsI	PhMe	No light, 50 °C	0%	50%	35%
CsI	PhMe	Ambient light, r.t.	0%	75%	22%

Oxidant Loading and Catalytic I_2 Studies

Benzimidate **3** was subjected to **GP3**, with different loadings of CsI and $PhI(OAc)_2$ listed below. Upon completion, the resultant crude mixture was concentrated and quantified via ^1H NMR using 1 equiv of 1,2-dichloroethane as an internal standard.

Table S5. Oxidant loading studies

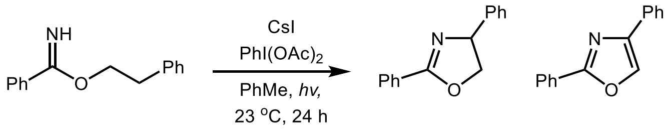

CsI (eq)	$PhI(OAc)_2$ (eq)	Imidate	Oxazoline	Oxazole
1	1	54%	6%	34%
2	2	15%	14%	59%
**3**	**3**	**0%**	**0%**	**98%**

Conditions: imidate (0.2 mmol), solvent (2 mL), 23 W compact fluorescent light (CFL), 23 °C. ^1H NMR yields vs DCE standard.

Intermediate oxazoline **2** was subjected to oxidation conditions which have been reported in the literature for oxazoline to oxazole conversion.

Benzimidate **3** was subjected to conditions reported in the literature for $PhI(OAc)_2$ oxidation with catalytic amounts of I_2 listed below.[7] Upon completion, the resultant crude mixture was concentrated and quantified via ^1H NMR using 1 equiv of 1,2-dichloroethane as an internal standard.

Table S6. Catalytic I_2 conditions

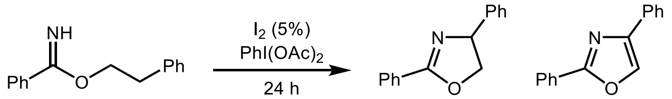

Solvent	$PhI(OAc)_2$ (eq)	Variation	Imidate	Oxazoline	Oxazole
DMF	1.2	50 °C	0%	95%	0%
DMF	2.2	50 °C	0%	84%	0%
MeCN	2.2	50 °C	0%	90%	0%
PhMe	2.2	50 °C	0%	78%	6%
**PhMe**	**2.2**	**23 W CFL, 23 °C**	**0%**	**24%**	**58%**
PhMe	3.0	23 W CFL, 23 °C, 20% I_2	0%	11%	60%

Conditions: imidate (0.2 mmol), solvent (1 mL), I_2 (5 mol%). ^1H NMR yields vs DCE standard.

Time Studies

Benzimidate **3** was subjected to **GP3**, and quenched at different times listed below. Upon completion, the resultant crude mixture was concentrated and quantified via ^1H NMR using 1 equiv of 1,2-dichloroethane as an internal standard.

[7] Stateman, L. M.; Wappes, E. A.; Nakafuku, K. M.; Edwards, K. M.; Nagib, D. A. *Chem. Sci.* **2019**, *10*, 2693 − 2699.

Table S7. Time study of tandem oxidation

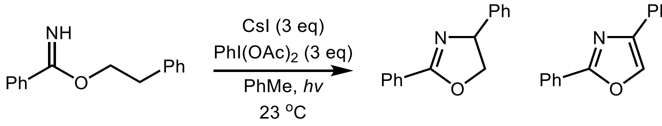

Time (h)	Imidate	Oxazoline	Oxazole
1	76%	23%	5%
2	50%	34%	10%
4	50%	32%	13%
8	0%	34%	54%
10	0%	18%	83%
**12**	**0%**	**6%**	**96% (97%)***
14	0%	0%	94%

Conditions: imidate (0.2 mmol), CsI (3 equiv), $PhI(OAc)_2$ (3 equiv), solvent (2 mL), 23 W compact fluorescent light (CFL), 23 °C. ^{1}H NMR yields vs DCE standard. *denotes isolated yield.

Longer runtimes did not result in a decreased yield of oxazole **3**, indicating the stability of the product under reaction conditions.

Optimization of Trichloroacetimidate Tandem Oxidation

Trichloroacetimidate **S28** was subjected to GP3, with modified conditions listed below. Upon completion, the crude mixture was quenched, concentrated, and quantified via ^{1}H NMR using 1 equiv of 1,2-dichloroethane as an internal standard.

Table S8. Optimization of tandem oxidation for trichloroacetimidates

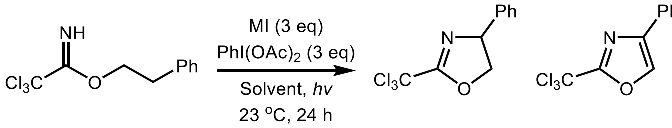

MI	Solvent	Oxazoline	Oxazole
NaI	MeCN	100%	0%
NaI	PhMe	76%	16%
NaI	$PhMe/CHCl_3$ (3:1)	11%	52%
**NaI**	**$PhMe/CHCl_3$ (1:1)**	**10%**	**58%**
NaI	$PhMe/CHCl_3$ (1:3)	19%	50%
NaI	$CHCl_3$	5%	40%
CsI	$PhMe/CHCl_3$ (50:50)	19%	28%

Other oxidants were tried and found to unsuccessfully promote the tandem oxidation, including: $PhI(OTFA)_2$, Dess–Martin periodinane, IBX, N-iodosuccinimide, 1-acetoxy-1,2-benziodoxol-3-(1H)-one, Koser’s reagent.

VI. Tandem Oxidation

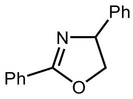

2,4-diphenyl-4,5-dihydrooxazole (2)

Oxazoline **2** was prepared following a literature method.[7]

To a 10 mL round bottom flask and stir bar was added benzimidate 1 (0.4 g, 1.8 mmol), and $PhI(OAc)_2$ (0.7 g, 2.2 mmol). This vial was evacuated and backfilled with N_2 (3x). A degassed stock solution of I_2 (47 mg, 0.018 mmol) in dry DMF (4.5 mL) was added to the flask under N_2. The reaction was heated to 50°C (by placing vial in an aluminum heating block) and stirred for 2 hours. Upon completion, the reaction was quenched with 10% aq. $Na_2S_2O_3$, extracted with EtOAc, and washed with H_2O. The crude was then purified via column chromatography (silica gel treated with 1% Et_3N in hexanes), yielding oxazoline **2** (0.34 g, 85%) as a light yellow oil. Characterization data is consistent with reported literature data.[3]

^{1}H NMR (400 MHz, $CDCl_3$): δ = 8.07 – 8.02 (m, 1H), 7.47 – 7.41 (m, 2H), 7.39 – 7.25 (m, 5H), 5.39 (dd, *J* = 10.2, 8.2 Hz, 1H), 4.80 (dd, *J* = 10.0, 8.4 Hz, 1H), 4.28 (app t, *J* = 8.3 Hz, 1H).

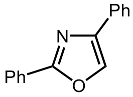

2,4-diphenyloxazole (3)

Benzimidate **1** (45 mg, 0.2 mmol) was subjected to **GP3**. Purification via column chromatography, yielded oxazole **3** (43 mg, 97%) as an off-white solid.

R_f: 0.48 (10% Ethyl acetate/hexanes)
^{1}H NMR (400 MHz, $CDCl_3$): δ = 8.16 – 8.10 (m, 2H), 7.97 (s, 1H), 7.86 – 7.80 (m, 2H), 7.53 – 7.38 (m, 6H), 7.37 – 7.31 (m, 1H).
^{13}C NMR (100 MHz, $CDCl_3$): δ = 162.1, 142.2, 133.6, 131.3, 130.5, 128.9, 128.3, 127.7, 126.7, 125.80
HRMS (ESI-TOF) *m/z*: calc'd for $C_{15}H_{12}NO^+$ $[M+H]^+$ 222.0913, found 222.0920.
IR (film) (cm^{-1}): 2924, 2852, 2360, 2341, 1553, 1487, 1146, 1339, 1290, 1274, 1157, 1123, 1069, 1022, 942, 929.
MP: 94 – 96 °C

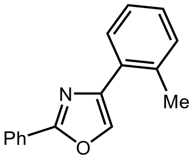

2-phenyl-4-(o-tolyl)oxazole (4)

Benzimidate **S4** (48 mg, 0.2 mmol) was subjected to **GP3**. Purification via column chromatography yielded oxazole **4** (44 mg, 94%) as an off-white solid.

R_f: 0.45 (5% Ethyl acetate/hexanes)
^{1}H NMR (400 MHz, $CDCl_3$): δ = 8.21 – 8.08 (m, 2H), 7.98 – 7.88 (m, 1H), 7.82 (s, 1H), 7.56 – 7.40 (m, 3H), 7.34 – 7.26 (m, 3H), 2.51 (s, 3H).
^{13}C NMR (100 MHz, $CDCl_3$): δ = 161.1, 141.2, 135.8, 135.4, 131.0, 130.6, 130.5, 128.9, 128.9, 128.1, 127.7, 126.7, 126.2, 21.9.
HRMS (ESI-TOF) *m/z*: calc'd for $C_{16}H_{14}NO^+$ $[M+H]^+$ 236.1070, found 236.1067.
IR (film) cm^{-1}: 3177, 3059, 2926, 2359, 2341, 1556, 1488, 1462, 1448, 1378, 1339, 1288, 1262, 1235, 1134, 1111, 1081, 1066, 1043, 1023, 934
M.P 65 – 66 °C.

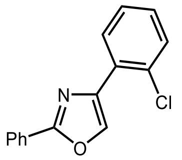

4-(2-chlorophenyl)-2-phenyloxazole (5)

Benzimidate **S5** (52 mg, 0.2 mmol) was subjected to **GP3**. Purification via column chromatography yielded oxazole **5** (33 mg, 63%) as a tan solid.

R_f: 0.45 (5% Ethyl acetate/hexanes)
^{1}H NMR (400 MHz, $CDCl_3$): δ = 8.41 (s, 1H), 8.28 (dd, J = 7.8, 1.7 Hz, 1H), 8.16 – 8.12 (m, 2H), 7.52 – 7.45 (m, 4H), 7.41 – 7.37 (td, J = 7.9, 1.3 Hz, 1H), 7.28 – 7.24 (m, 1H).
^{13}C NMR (100 MHz, $CDCl_3$): δ = 160.9, 138.2, 137.4, 131.7, 130.6, 130.4, 130.1, 130.0, 128.9, 128.8, 127.5, 127.2, 126.7.
HRMS (ESI-TOF) *m/z*: calc'd for $C_{17}H_{12}NO^+$ $[M+H]^+$ 256.0524, found 256.0517.
IR (film) cm^{-1}: 3191, 3045, 1611.
M.P 89 – 91 °C.

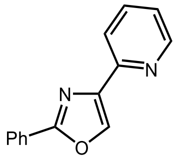

2-phenyl-4-(pyridin-2-yl)oxazole (6)

Benzimidate **S6** (45 mg, 0.2 mmol) was subjected to **GP3**, with the following modifications: addition of K_2HPO_4 (40 mg, 0.2 mmol). Purification via column chromatography yielded oxazole **6** (22 mg, 49%) as a tan solid.

R_f: 0.25 (30% Ethyl acetate/hexanes)
^{1}H NMR (400 MHz, $CDCl_3$): δ = 8.61 (dq, J = 4.8, 0.8 Hz, 1H), 8.31 (s, 1H), 8.01 (dt, J = 7.7, 1.0 Hz, 1H), 7.78 (td, J = 7.7, 1.8 Hz, 1H), 7.52 – 7.45 (m, 3H), 7.23 (ddd, J = 7.6, 4.8, 1.2 Hz, 1H).
^{13}C NMR (100 MHz, $CDCl_3$): δ = 162.2, 151.0, 149.7, 142.4, 137.0, 130.7, 128.9, 127.5, 126.7, 122.9, 120.5.
HRMS (ESI-TOF) *m/z*: calc'd for $C_{15}H_{11}NO^+$ $[M+H]^+$ 223.0866, found 223.0871.
IR (film) cm^{-1}: 3121, 3058, 1607, 1576.
M.P 94 – 95 °C.

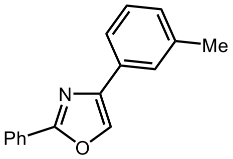

2-phenyl-4-(*m*-tolyl)oxazole (7)

Benzimidate **S7** (48 mg, 0.2 mmol) was subjected to **GP3**. Purification via column chromatography yielded oxazole **7** (44 mg, 94%) as a white solid.

R_f: 0.45 (10% Ethyl acetate in hexanes)
^{1}H NMR (400 MHz, $CDCl_3$): δ = 8.19 – 8.07 (m, 2H), 7.96 (s, 1H), 7.70 – 7.65 (m, 1H), 7.64 – 7.58 (m, 1H), 7.53 – 7.42 (m, 3H), 7.35 – 7.29 (t, H = 7.7 Hz, 1 H), 7.18 – 7.12 (m, 1H), 2.42 (s, 3H).
^{13}C NMR (150 MHz, $CDCl_3$): δ = 162.4, 142.3, 138.6, 133.6, 131.2, 130.5, 129.1, 128.9, 128.8, 127.7, 126.7, 126.5, 122.9, 21.6.
HRMS (ESI-TOF) *m/z*: calc'd for $C_{16}H_{14}NO^+$ $[M+H]^+$ 236.1070, found 236.1067.
IR (film) (cm^{-1}): 3058, 2920, 2857, 2359, 2341, 1616, 1598, 1555, 1488, 1448, 1337, 1275, 1113, 1081, 1060, 1023, 960, 932.
MP: 50 – 51 °C.

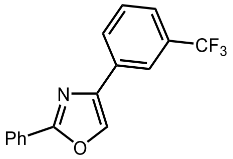

2-phenyl-4-(3-(trifluoromethyl)phenyl)oxazole (8)

Benzimidate **S8** (59 mg, 0.2 mmol) was subjected to **GP3**. Purification via column chromatography yielded oxazole **8** (43 mg, 77%) as a white solid.

R_f: 0.38 (10% Ethyl acetate in hexanes)
^{1}H NMR (400 MHz, $CDCl_3$): δ = 8.17 – 8.09 (m, 3H), 8.03 (s, 1H), 8.03 – 7.98 (m, 1H), 7.62 – 7.52 (m, 2H), 7.52 – 7.46 (m, 3H).
^{13}C NMR (150 MHz, $CDCl_3$): δ = 162.4, 141.0, 134.2, 132.2, 131.4 (q, $^2J_{CF}$ = 32.3 Hz), 130.8, 129.4, 129.0, 126.7, 124.8 (q, $^3J_{CF}$ = 3.8 Hz), 124.22 (q, $^1J_{CF}$ = 272.3 Hz), 122.6 (q, $^3J_{CF}$ = 4.0 Hz).
^{19}F NMR (376 MHz, $CDCl_3$): δ = –62.8.
HRMS (ESI-TOF) *m/z*: calc'd for $C_{16}H_{11}F_3NO^+$ $[M+H]^+$ 290.0787, found 290.0789.
IR (film) (cm^{-1}): 2359, 2341, 1620 ,1558, 1491, 1479, 1448, 1341, 1317, 1284, 1277, 123, 1169, 1157, 1107, 1094, 1060, 1022, 956, 931
MP: 70 – 72 °C.

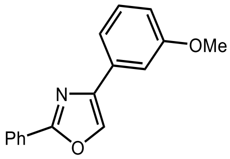

4-(3-methoxyphenyl)-2-phenyloxazole (9)

Benzimidate **S9** (51 mg, 0.2 mmol) was subjected to **GP3**. Purification via column chromatography yielded oxazole **9** (50 mg, 100%) as a white solid.

R_f: 0.28 (10% Ethyl acetate in hexanes)
^{1}H NMR (600 MHz, $CDCl_3$): δ = 8.14 – 8.12 (m, 2H), 7.98 (s, 1H), 7.50 – 7.47 (m, 3H), 7.42 (dd, J = 2.5, 1.4 Hz, 1H), 7.39 (dt, J = 7.6, 1.2 Hz, 1H), 7.34 (t, J = 7.9 Hz, 1H), 6.89 (ddd, J = 8.1, 2.6, 1.0 Hz, 1H), 3.89 (s, 3H).
^{13}C NMR (150 MHz, $CDCl_3$): δ = 162.0, 160.2, 142.1, 133.8, 132.6, 130.6, 129.9, 128.9, 127.6, 126.7, 118.2, 114.2, 111.1, 55.5.
HRMS (ESI-TOF) *m/z*: calc'd for $C_{16}H_{14}NO_2^+$ $[M+H]^+$ 252.1019, found 252.1021.
IR (film) (cm^{-1}): 3061, 2999, 2935, 2833, 2360, 2341, 1609, 1591, 1571, 1555, 1489, 1465, 1447, 1431, 1337, 1320, 1303, 1280, 1245, 1212, 1177, 1079, 1043, 1023, 960, 932.
MP: 67 – 68 °C.

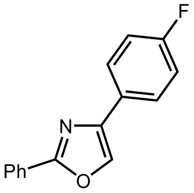

4-(4-fluorophenyl)-2-phenyloxazole (10)

Benzimidate **S10** (49 mg, 0.2 mmol) was subjected to **GP3**. Purification via column chromatography yielded oxazole **10** (41 mg, 85%) as an off-white solid.

R_f: 0.35 (5% Ethyl acetate/hexanes)
^{1}H NMR (400 MHz, $CDCl_3$): δ = 8.12 (m, 2H), 7.92 (s, 1H), 7.80 (dd, J = 8.9, 5.4 Hz, 2H), 7.48 (m, 3H), 7.13 (t, J = 8.8 Hz, 2H).
^{13}C NMR (100 MHz, $CDCl_3$): δ = 162.8 (q, $^1J_{C\text{-}F}$ = 247.2 Hz), 162.1, 141.3, 133.2 (q, $^4J_{C\text{-}F}$ = 1.1 Hz), 130.6, 128.9, 127.6, 127.5 (q, $^3J_{CF}$ = 8.0 Hz), 126.6, 115.9 (q, $^2J_{CF}$ = 21.8 Hz).
^{19}F NMR (376 MHz, $CDCl_3$): δ = –114.68.
HRMS (ESI-TOF) *m/z*: calc'd for $C_{15}H_{11}FNO^+$ $[M+H]^+$ 240.0819, found 240.0830.
IR (film) cm^{-1}: 3132, 3059, 1718, 1610, 1556.
M.P 104 – 105 °C.

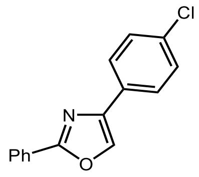

4-(4-chlorophenyl)-2-phenyloxazole (11)

Benzimidate **S11** (42 mg, 0.2 mmol) was subjected to GP3. Purification via column chromatography yielded oxazole **11** (39 mg, 94%) as a white solid.

R$_f$: 0.50 (5% Ethyl acetate/hexanes)
^{1}H NMR (400 MHz, CDCl$_3$): δ = 8.14 – 8.09 (m, 2H), 7.96 (s, 1H), 7.86 – 7.75 (m, 2H), 7.52 – 7.46 (m, 3H), 7.42 – 7.39 (m, 2H).
^{13}C NMR (100 MHz, CDCl$_3$): δ = 162.2, 141.2, 133.9, 133.7, 130.7, 129.8, 129.1, 128.9, 127.5, 127.1, 126.7
HRMS (ESI-TOF) *m/z*: calc'd for $C_{15}H_{10}NNaO^+$ [M+Na]$^+$ 378.0343, found 378.0374
IR (film) (cm^{-1}): 3364, 3120, 3057, 2959, 2923, 2852, 1657, 1609, 1565, 1555, 1486, 1449, 1404, 1340, 1312, 1292, 1271, 1121, 1103, 1068, 191, 1059, 1024, 1013, 942, 932, 829
MP: 129 °C.

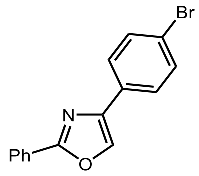

4-(4-bromophenyl)-2-phenyloxazole (12)

Benzimidate **S12** (61 mg, 0.2 mmol) was subjected to GP3. Purification via column chromatography yielded oxazole **12** (59 mg, 98%) as a white solid.

R$_f$: 0.53 (5% Ethyl acetate/hexanes)
^{1}H NMR (400 MHz, CDCl$_3$): δ = 8.14 – 8.08 (m, 2H), 7.97 (s, 1H), 7.73 – 7.68 (m, 2H), 7.59 – 7.53 (m, 2H), 7.52 – 7.45 (m, 3H).
^{13}C NMR (100 MHz, CDCl$_3$): δ = 162.26, 141.25, 133.72, 132.03, 130.69, 130.28, 128.94, 127.47, 127.34, 126.69, 122.08.
HRMS (ESI-TOF) *m/z*: calc'd for $C_{15}H_{11}BrNO^+$ [M+H]$^+$ 300.0019, found 300.0022.
IR (film) (cm^{-1}): 3369, 3181, 3055, 2952, 2853, 1726, 1659, 1606, 1589, 1577
MP: 137 – 138 °C.

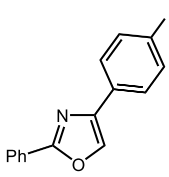

4-(4-iodophenyl)-2-phenyloxazole (13)

Benzimidate **S13** (70 mg, 0.2 mmol) was subjected to GP3. Purification via column chromatography yielded oxazole **13** (64 mg, 93%) as a white solid.

R$_f$: 0.45 (10% Ethyl acetate in hexanes)
^{1}H NMR (400 MHz, $CDCl_3$): δ = 8.12 – 8.10 (m, 2H), 7.98 (s, 1H), 7.78 – 7.75 (m, 2H), 7.59 – 7.56 (m, 2H), 7.50 – 7.46 (m, 3H).
^{13}C NMR (100 MHz, $CDCl_3$): δ = 162.1, 141.2, 137.8, 133.7, 130.7, 130.6, 128.8, 127.4, 127.3, 126.6, 93.4.
HRMS (ESI-TOF) *m/z*: calc'd for $C_{15}H_{11}INO^+$ $[M+H]^+$ 347.9880, found 347.9869.
IR (film) (cm^{-1}): 2360, 2342, 1157, 1473, 1447, 1396, 1338, 1267, 1120, 1100, 1063, 1022, 1005, 940, 932.
MP: 156 °C.

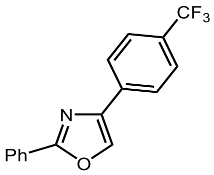

2-phenyl-4-(4-(trifluoromethyl)phenyl)oxazole (14)

Benzimidate **S14** (59 mg, 0.2 mmol) was subjected to GP3. Purification via column chromatography yielded oxazole **14** (49 mg, 84%) as a white solid.

R$_f$: 0.43 (5% Ethyl acetate/hexanes)

^{1}H NMR (400 MHz, $CDCl_3$): δ = 8.16 – 8.10 (m, 2H), 8.05 (s, 1H), 7.98 – 7.92 (d, J = 8.0 Hz, 2H), 7.66-7.72 (d, J = 8.3 Hz, 2H), 7.53 – 7.47 (m, 3H).
^{13}C NMR (100 MHz, $CDCl_3$): δ = 162.5, 141.0, 134.8, 134.6, 130.8, 130.1 (q, $^2J_{CF}$ = 32.4 Hz), 129.0, 127.4, 126.7, 125.9, 125.9 (q, $^3J_{CF}$ = 3.8 Hz), 124.3 (q, $^1J_{CF}$ = 272.0 Hz).
^{19}F NMR (376 MHz, $CDCl_3$): δ = –62.56.
HRMS (ESI-TOF) *m/z*: calc'd for $C_{16}H_{11}F_3NO^+$ $[M+H]^+$ 290.0787, found 290.0789.
IR (film) (cm^{-1}): 2359, 2342, 1620, 1558, 1450, 1415, 1335, 1166, 1156, 1110, 1076, 1065, 943, 930.
MP: 145 – 146 °C.

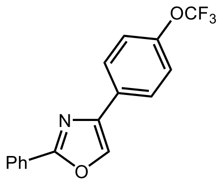

2-phenyl-4-(4-(trifluoromethoxy)phenyl)oxazole (15)

Benzimidate **S15** (62 mg, 0.2 mmol) was subjected to **GP3**. Purification via column chromatography yielded oxazole **15** (57 mg, 94%) as a white solid.

R_f: 0.53 (5% Ethyl acetate/hexanes)
^{1}H NMR (400 MHz, $CDCl_3$): δ = 8.15 – 8.10 (m, 2H), 7.97 (s, 1H), 7.89 – 7.83 (m, 2H), 7.51 – 7.46 (m, 3H), 7.32 – 7.27 (m, 2H).
^{13}C NMR (150 MHz, $CDCl_3$): δ = 162.3, 149.1, 141.0, 133.7, 130.7, 130.1, 129.0, 127.5, 127.2, 126.7, 121.4, 120.7 (q, $^1J_{CF}$ = 257.4 Hz).
^{19}F NMR (376 MHz, $CDCl_3$): δ = –57.8.
HRMS (ESI-TOF) *m/z*: calc'd for $C_{16}H_{11}F_3NO_2^+$ $[M+H]^+$ 306.0736, found 306.0737.
IR (film) (cm^{-1}): 3054, 2983, 2928, 2111, 2029, 2008, 1963, 1574, 1499, 1450
MP: 96 °C.

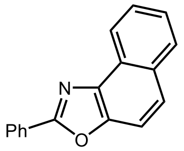

2-phenylnaphtho[1,2-d]oxazole (16)

Benzimidate **S16** (50 mg, 0.2 mmol) was subjected to **GP3**, with the following modifications: additional CsI (208 mg, 0.8 mmol), $PhI(OAc)_2$ (258 mg, 0.8 mmol), and PhMe (3 mL). Purification via column chromatography yielded oxazole **16** (37 mg, 75%) as an off-white solid.

R_f: 0.35 (5% Ethyl acetate/hexanes)
^{1}H NMR (400 MHz, $CDCl_3$): δ = 8.62 (d, *J* = 8.2 Hz, 1H), 8.35 (m, 2H), 7.97 (d, *J* = 8.2 Hz, 1H), 7.80 (d, *J* = 8.8 Hz, 1H), 7.73 (d, *J* = 8.8 Hz, 1H), 7.70 – 7.67 (m, 1H), 7.57 – 7.51 (m, 4H).
^{13}C NMR (100 MHz, $CDCl_3$): δ = 162.4, 148.2, 137.8, 131.4, 131.2, 129.0, 128.7, 127.7, 127.5, 127.1, 126.7, 126.1, 125.5, 122.4, 110.9
HRMS (ESI-TOF) *m/z*: calc'd for $C_{17}H_{12}NO^+$ $[M+H]^+$ 264.0913, found 264.0917.
IR (film) cm^{-1}: 3068, 2924, 1639, 1551.
M.P 133 – 134 °C.

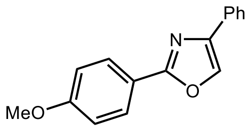

2-(4-methoxyphenyl)-4-phenyloxazole (17)

Benzimidate **S17** (51 mg, 0.2 mmol) was subjected to **GP3**. Purification via column chromatography yielded oxazole **17** (38 mg, 76%) as a white solid.

R_f: 0.50 (20% Ethyl acetate/hexanes)
^{1}H NMR (400 MHz, $CDCl_3$): δ = 8.08 – 8.04 (m, 2H), 7.92 (s, 1H), 7.83 – 7.80 (m, 2H), 7.45 – 7.41 (m, 2H), 7.35 – 7.31 (m, 1H), 7.01 – 6.98 (m, 2H), 3.88 (s, 3H).
^{13}C NMR (100 MHz, $CDCl_3$): δ 162.1, 161.5, 141.9, 133.0, 131.4, 128.8, 128.3, 128.1, 125.7, 120.5, 114.3, 55.5.
HRMS (ESI-TOF) m/z: calc'd for $C_{16}H_{14}NO_2^+$ $[M+H]^+$ expected 252.1025, found 252.1019.
IR (film) cm^{-1}: 3042, 2969, 2920, 2843, 1613, 1592.
MP: 95 – 96 °C.

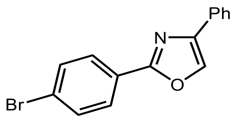

2-(4-bromophenyl)-4-phenyloxazole (18)

Benzimidate **S18** (61 mg, 0.2 mmol) was subjected to GP3**.** Purification via column chromatography yielded oxazole **18** (55 mg, 92%) as a white solid.

R_f: 0.65 (20% Ethyl acetate/hexanes)
^{1}H NMR (400 MHz, $CDCl_3$): δ = 8.01 – 7.97 (m, 3H), 7.82 – 7.80 (m, 2H), 7.64 – 7.60 (m, 2H), 7.46 – 7.41 (m, 2H), 7.37 – 7.32 (m, 1H).
^{13}C NMR (150 MHz, $CDCl_3$): δ 161.2, 142.4, 133.8, 132.2, 131.1, 128.9, 128.4, 128.1, 126.6, 125.8, 125.0.
HRMS (ESI-TOF) m/z: calc'd for $C_{15}H_{11}BrNO^+$ $[M+H]^+$ expected 300.0024, found 299.9998.
IR (film) cm^{-1}: 3120, 3078, 3058, 3026, 2981, 1600, 1551.
MP: 143 – 145 °C.

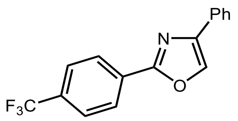

4-phenyl-2-(4-(trifluoromethyl)phenyl)oxazole (19)

Benzimidate **S19** (59 mg, 0.2 mmol) was subjected to GP3**.** Purification via column chromatography yielded oxazole **19** (45 mg, 77%) as a white solid.

R_f: 0.40 (20% Ethyl acetate/hexanes)
^{1}H NMR (400 MHz, $CDCl_3$): δ = 8.25 – 8.23 (m, 2H), 8.02 (s, 1H), 7.84 – 7.82 (m, 2H), 7.76 – 7.73 (m, 2H), 7.47 – 7.43 (m, 2H), 7.38 – 7.34 (m, 1H).
^{13}C NMR (150 MHz, $CDCl_3$): δ = 160.7, 142.7, 134.3, 132.2 (^2J = 32.0 Hz), 130.9, 130.8, 129.0, 128.6, 126.9, 126.0 (^3J = 4.3 Hz), 125.86, 125.85 (^1J = 272.5 Hz), 123.1.
HRMS (ESI-TOF) m/z: calc'd for $C_{16}H_{11}F_3NO^+$ $[M+H]^+$ expected 290.0793, found 290.0822.
IR (film) cm^{-1}: 2981, 2923, 2890, 1659, 1620, 1587, 1563.
MP: 143 – 144 °C.

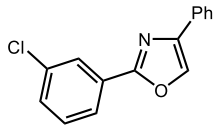

2-(3-chlorophenyl)-4-phenyloxazole (20)

Benzimidate **S20** (52 mg, 0.2 mmol) was subjected to GP3**.** Purification via column chromatography yielded oxazole **20** (59 mg, 89%) as a low-melting yellow solid.

R_f: 0.69 (20% Ethyl acetate/hexanes)
^{1}H NMR (400 MHz, $CDCl_3$): δ = 8.13 – 8.12 (m, 1H), 8.02 – 7.99 (m, 1H), 7.98 (s, 1H), 7.83 – 7.81 (m, 2H), 7.46 – 7.42 (m, 4H), 7.37 – 7.33 (m, 1H).
^{13}C NMR (100 MHz, $CDCl_3$): δ = 160.7, 142.4, 135.0, 133.9, 131.0, 130.5, 130.2, 129.3, 128.9, 128.4, 126.7, 125.8, 124.7.
HRMS (ESI-TOF) m/z: calc'd for $C_{15}H_{11}ClNO^+$ $[M+H]^+$ expected 256.0529, found 256.0526.
IR (film) cm^{-1}: 3123, 3062, 2981, 2881, 1587, 1549.

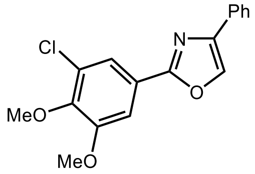

2-(3-chloro-4,5-dimethoxyphenyl)-4-phenyloxazole (21)

Benzimidate **S21** (64 mg, 0.2 mmol) was subjected to GP3**.** Purification via column chromatography yielded oxazole **21** (41 mg, 70%) as a white solid.

R_f: 0.24 (5% Ethyl acetate/hexanes)
^{1}H NMR (400 MHz, $CDCl_3$): δ = 7.96 (s, 1H), 7.85-7.78 (m, 2H), 7.78 – 7.73 (m, 1H), 7.62 – 7.56 (m, 1H), 7.48 – 7.40 (m, 2H), 7.38 – 7.31 (m, 1H), 3.99 (s, 3H), 3.93 (s, 3H).
^{13}C NMR (150 MHz, $CDCl_3$): δ = 160.8, 154.2, 147.5, 142.4, 133.8, 131.0, 129.0, 128.9, 128.4, 125.8, 123.9, 120.5, 109.1, 61.0, 56.5.
HRMS (ESI-TOF) *m/z*: calc'd for $C_{17}H_{15}ClNO_3^+$ $[M+H]^+$ 316.0735, found 316.0735.
IR (film) cm^{-1}: 3055, 2939, 2833, 1549, 1487, 1464, 1447, 1429, 1410.
MP: 96 °C.

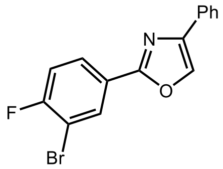

2-(3-bromo-4-fluorophenyl)-4-phenyloxazole (22)

Benzimidate **S22** (64 mg, 0.2 mmol) was subjected to GP3. Purification via column chromatography yielded oxazole **22** (51 mg, 81%) as a white solid.

R_f: 0.65 (20% Ethyl acetate/hexanes)

^{1}H NMR (600 MHz, $CDCl_3$): δ = 8.34 (dd, *J* = 6.5, 2.1 Hz, 1H), 8.04 (ddd, J = 8.6, 4.7, 2.1 Hz, 1H), 7.96 (s, 1H), 7.82 – 7.80 (m, 2H), 7.45 – 7.43 (m, 2H), 7.37 – 7.34 (m, 1H), 7.22 (app t, J = 8.3 Hz, 1H).
^{13}C NMR (150 MHz, $CDCl_3$): δ = 161.3, 159.8 ($^{1}J_{CF}$ = 35 Hz), 142.5, 133.9, 132.0, 130.9, 128.9, 128.5, 127.4 ($^{3}J_{CF}$ = 7.7 Hz), 125.8, 125.3 ($^{4}J_{CF}$ = 4.2 Hz), 117.1 ($^{2}J_{CF}$ = 23.1 Hz), 109.9 ($^{2}J_{CF}$ = 21.8 Hz).
HRMS (ESI-TOF) m/z: calc'd for $C_{15}H_{10}BrFNO^+$ [M+H]$^+$ expected 317.9930, found 317.9915.
IR (film) cm^{-1}: 3145, 3092, 3034, 2981, 1603, 1585, 1557.
MP: 101 – 102 °C.

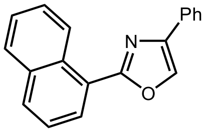

2-(naphthalen-1-yl)-4-phenyloxazole (23)

Benzimidate **S23** (55 mg, 0.2 mmol) was subjected to **GP3**. Purification via column chromatography yielded oxazole **23** (44 mg, 86%) as a yellow solid.

R_f: 0.55 (20% Ethyl acetate/hexanes)
^{1}H NMR (400 MHz, $CDCl_3$): δ = 9.47 – 9.44 (m, 1H), 8.27 (dd, *J* = 7.3, 1.2 Hz, 1H), 8.08 (s, 1H), 8.00 – 7.98 (m, 1H), 7.95 – 7.92 (m, 3H), 7.71 – 7.67 (m, 1H), 7.61 – 7.56 (m, 2H), 7.50 – 7.46 (m, 2H), 7.40 – 7.35 (m, 1H).
^{13}C NMR (100 MHz, $CDCl_3$): δ = 161.9, 142.1, 134.1, 133.3, 131.42, 131.39, 130.4, 128.9, 128.7, 128.3, 128.0, 127.7, 126.5, 126.4, 125.9, 125.1, 124.1.
HRMS (ESI-TOF) m/z: calc'd for $C_{19}H_{14}NO$ [M+H]$^+$ expected 272.1075, found 272.1103.
IR (film) cm^{-1}: 3145, 3048, 2979, 2923, 1587, 1542.
MP: 70 – 72 °C.

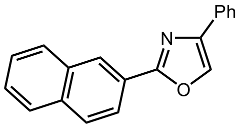

2-(naphthalen-2-yl)-4-phenyloxazole (24)

Benzimidate **S24** (61 mg, 0.2 mmol) was subjected to **GP3**. Purification via column chromatography yielded oxazole **24** (43 mg, 67%; corrected yield, contains ca. 10% inseparable iodinated oxazole) as a white solid.

R_f: 0.61 (20% Ethyl acetate/hexanes)
^{1}H NMR (400 MHz, $CDCl_3$): δ = 8.62 (s, 1H), 8.23 (dd, *J* = 8.5, 1.7 Hz, 1H), 8.02 (s, 1H), 7.98 – 7.94 (m, 2H), 7.89 – 7.86 (m, 3H), 7.57 – 7.54 (m, 2H), 7.48 – 7.44 (m, 2H), 7.38 – 7.34 (m, 1H).
^{13}C NMR (100 MHz, $CDCl_3$): δ = 162.2, 142.4, 134.3, 133.7, 133.2, 131.3, 128.91, 128.89, 128.7, 128.3, 128.0, 127.4, 126.9, 126.6, 125.8, 125.0, 123.7.
HRMS (ESI-TOF) m/z: calc'd for $C_{19}H_{14}NO$ [M+H]$^+$ expected 272.1075, found 272.1049.
IR (film) cm^{-1}: 3059, 2980, 2887, 1609, 1586, 1549.

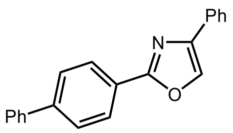

2-([1,1'-biphenyl]-4-yl)-4-phenyloxazole (25)

Benzimidate **S25** (60 mg, 0.2 mmol) was subjected to GP3. Purification via column chromatography yielded oxazole **25** (40 mg, 67%) as a white solid.

R_f: 0.66 (20% Ethyl acetate/hexanes)
^{1}H NMR (600 MHz, $CDCl_3$): δ = 8.21 – 8.19 (m, 2H), 7.99 (s, 1H), 7.86 – 7.84 (m, 2H), 7.73 – 7.71 (m, 2H), 7.67 – 7.65 (m, 2H), 7.49 – 7.44 (m, 4H), 7.41 – 7.38 (m, 1H), 7.37 – 7.34 (m, 1H).
^{13}C NMR (150 MHz, $CDCl_3$): δ = 162.0, 143.2, 142.3, 140.4, 133.6, 131.3, 129.1, 128.9, 128.3, 128.0, 127.6, 127.3, 127.1, 126.5, 125.8.
HRMS (ESI-TOF) m/z: calc'd for $C_{21}H_{16}NO$ $[M+H]^+$ expected 298.1232, found 298.1247.
IR (film) cm^{-1}: 3056, 3038, 2981, 2919, 2850, 1610, 1576.
MP: 161 – 163 °C.

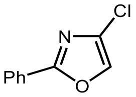

4-chloro-2-phenyloxazole (26)

Benzimidate hydrotriflate **S26** (67 mg, 0.2 mmol) was subjected to GP3, with the following modifications: addition of NaOAc (33 mg, 0.4 mmol) to effect the freebase *in situ*. Analysis of the crude by ^{1}H NMR with 1 equiv of 1,2-dichloroethane as an internal standard gave 60% yield. Purification via column chromatography yielded oxazole **26** (4.9 mg, 27%) as a white solid.

R_f: 0.40 (3% Ethyl acetate/hexanes)
^{1}H NMR (400 MHz, $CDCl_3$): δ = 8.05 – 7.99 (m, 2H), 7.66 (s, 1H), 7.51 – 7.44 (m, 3H).
^{13}C NMR (100 MHz, $CDCl_3$): δ = 134.6, 133.7, 131.2, 130.8, 129.0, 129.0, 126.8, 126.6.
HRMS (ESI-TOF) m/z: calc'd for $C_9H_7ClNO^+$ $[M+H]^+$ expected 180.0211, found 180.0224.
IR (film) cm^{-1}: 2922, 2851, 2360, 2125, 1758, 1724, 1517, 1451, 1236, 1177, 1066, 1041, 1023, 999.

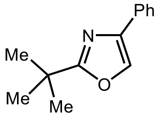

2-(tert-butyl)-4-phenyloxazole (27)

Pivalimidate **S27** (41 mg, 0.2 mmol) was subjected to GP3. Purification via column chromatography yielded oxazole **27** (31 mg, 77%) as a clear yellow oil.

R_f: 0.29 (5% Ethyl acetate/hexanes)
^{1}H NMR (400 MHz, $CDCl_3$): δ = 7.80 (s, 1H), 7.75 – 7.71 (m, 2H), 7.41 – 7.36 (m, 2H), 7.30 – 7.26 (m, 1H), 1.43 (s, 9H).
^{13}C NMR (100 MHz, $CDCl_3$): δ =171.7, 140.4, 132.9, 131.7, 128.8, 127.9, 125.7. 34.0, 28.7.
HRMS (ESI-TOF) *m/z*: calc'd for $C_{13}H_{16}NO$ $[M+H]^+$ 202.1226, found 202.1238.

IR (film) cm^{-1}: 3033, 2970, 1724, 1564.

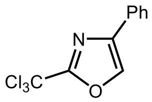

4-phenyl-2-(trichloromethyl)oxazole (28)

Trichloroacetimidate **S28** (53 mg, 0.2 mmol) was subjected to **GP3,** with the following modifications: NaI instead of CsI as the source of iodide, and a 50:50 mixture of PhMe/$CHCl_3$ instead of PhMe as solvent. Purification via column chromatography yielded oxazole **28** (33 mg, 63%) as a yellow solid.

Characterization data is consistent with reported literature data.[3]

R_f: 0.43 (10% Ethyl acetate in hexanes)
^{1}H NMR (400 MHz, $CDCl_3$): δ = 8.01 (s, 1H), 7.80 – 7.74 (m, 2H), 7.47 – 7.40 (m, 2H), 7.40 – 7.37 (m, 1H).

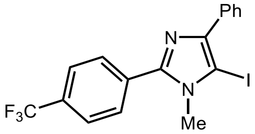

5-iodo-1-methyl-4-phenyl-2-(4-(trifluoromethyl)phenyl)-1H-imidazole (**29**)

Amidine **S29** (61 mg, 0.2 mmol) was subjected to **GP3,** with the following modifications: additional CsI (2 mg, 0.8 mmol) and $PhI(OAc)_2$ (258 mg, 0.8 mmol). Purification via column chromatography yielded imidazole **29** (59 mg, 68%) as a white solid.

R_f: 0.35 (10% Ethyl acetate in hexanes,
^{1}H NMR (400 MHz, $CDCl_3$): δ = 7.99 – 7.92 (m, 2H), 7.77 (q, J = 8.5 Hz, 4H), 7.47 – 7.40 (m, 2H), 7.37 – 7.30 (m, 1H), 3.77 (s, 3H).
^{13}C NMR (150 MHz, $CDCl_3$): δ = 149.6, 144.9, 134.3, 133.8, 131.2 (q, $^2J_{CF}$ = 32.7 Hz), 129.4, 128.4, 127.8, 127.7, 125.8 (q, $^3J_{CF}$ = 3.8 Hz), 124.0 (q, $^1J_{CF}$ = 273.5 Hz), 72.4, 36.5.
HRMS (ESI): m/z calculated for $C_{17}H_{13}F_3IN_2$ $[M+H]^+$: 429.0070, found 429.0063.
IR (film): 2981, 2361, 2342, 2160, 1977, 1616, 1528, 1481, 1459, 1444, 1378, 1325, 1165, 1120, 1106, 1073, 1013, 955, 850.
MP: 157 °C.

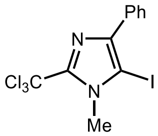

5-iodo-1-methyl-4-phenyl-2-(trichloromethyl)-1H-imidazole (30)

Amidine **S30** (61 mg, 0.2 mmol) was subjected to **GP3,** with the following modifications: additional CsI (2 mg, 0.8 mmol) and $PhI(OAc)_2$ (258 mg, 0.8 mmol). Purification via column chromatography yielded imidazole **30** (25 mg, 32%) as an orange solid.

R_f: 0.28 (10% Ethyl acetate in hexanes)
^{1}H NMR (400 MHz, $CDCl_3$): δ = 8.56 – 8.49 (m, 2H), 7.59 – 7.52 (m, 1H), 7.52 – 7.45 (m, 2H), 3.58 (s, 3H).
^{13}C NMR (150 MHz, $CDCl_3$): δ = 164.5, 158.3, 145.5, 132.8, 129.9, 129.3, 129.0, 111.6, 30.1.
HRMS (ESI): The molecule was not amenable to HRMS analysis and fragmented into a number of species.
IR (film): 3315, 2922, 1709, 1619, 1572, 1522, 1488, 1448, 1426, 1373, 1328, 1310, 1258, 1168, 1071, 1029, 1008.
MP: 99 – 100 °C.

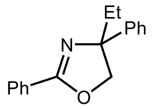

4-ethyl-2,4-diphenyl-4,5-dihydrooxazole (35)

Benzimidate **34** (19 mg, 0.08 mmol) was subjected to GP3**.** Analysis of the crude by ^{1}H NMR with 1 equiv of 1,2-dichloroethane as an internal standard gave 84% yield of oxazoline **35**.

R_f: 0.3 (5% Ethyl acetate/hexanes)
^{1}H NMR (600 MHz, $CDCl_3$): δ = 8.07 – 8.03 (m, 2H), 7.52 – 7.32 (m, 7H), 7.21 – 7.21 (m, 1h) 4.55(d, J = 8.5 Hz, 1H), 4.41(d, J = 8.5 Hz, 1H), 2.08 – 1.92 (m, 2H), 0.92 (t, J = 7.4 Hz, 3H).
^{13}C NMR (100 MHz, $CDCl_3$): δ = 162.6, 146.8, 131.3, 128.4, 128.4, 128.3, 128.0, 126.7, 125.7, 77.8, 35.5, 8.4.
HRMS (ESI-TOF) *m/z*: calc'd for $C_{17}H_{20}NO^+$ $[M+H]^+$252.1383, found 252.1468.

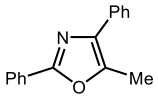

5-methyl-2,4-diphenyloxazole (40)

The two-step protocol outlined in **IX. Mechanistic Studies: Steric Effects of Benzimidates** was used to obtain oxazole **40** in 58% via ^{1}H NMR with 1,2-dichloromethane as internal standard. The crude material was worked up according to GP3. Purification via column chromatography yielded oxazole **40** as a white solid.

R_f: 0.28 (5% Ethyl acetate/hexanes)
^{1}H NMR (400 MHz, $CDCl_3$): δ = 8.10 – 8.07 (m, 2H), 7.76 – 7.74 (m, 2H), 7.49 – 7.43 (m, 2H), 7.35 – 7.31 (m, 1H), 2.62 (s, 3H).
^{13}C NMR (100 MHz, $CDCl_3$): δ = 159.5, 144.1, 136.1, 132.6, 130.1, 128.8, 128.7, 127.8, 127.4, 127.0, 126.3, 12.1.
HRMS (ESI-TOF) m/z: calc'd for $C_{16}H_{14}NO$ $[M+H]^+$ 236.1070, found 236.1065.
IR (film) cm^{-1}: 3089, 2915, 1615, 1597, 1555.
M.P 71 – 73 °C.

VII. One-Pot Optimization

Tandem Oxidation from Benzimidate Hydrotriflate Salt

In the development of a one-pot procedure, we explored the efficiency of the tandem oxidation from the benzimidate hydrotriflate salt. Benzimidate hydrotriflate salt **S2** was subjected to **GP3** with the addition of base described in **Table S9**.

Table S9. Investigation of bases for tandem oxidation

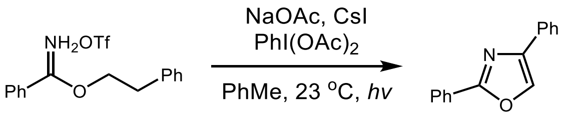

Base	Eq	Yield
None	-	53%
KOAc	1.1	53%
CsOAc	1.1	51%
$NaHCO_3$	1.1	33%
K_2HPO_4	1.1	50%
DBU	1.1	37%
Lutidine	1.1	34%
DTBP	1.1	38%
NaOAc	0.5	67%
NaOAc	1.1	65%
**NaOAc**	**1.5**	**74% (77%)**
NaOAc	2.0	71%

DBU = 1,8-Diazabicyclo[5.4.0]undec-7-ene, DTBP = 2,6-Di-tert-butylpyridine; quantification via ^{1}H NMR with 1 equiv of 1,2-dichloroethane as internal standard, parentheses denotes isolated yield.

One-pot Conversion of Alcohol to 2-Trichloromethyl Oxazole

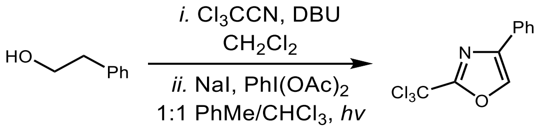

To a 2-dram vial containing a stir bar, 2-phenylethan-1-ol (24.0 μL, 0.2 mmol), CH_2Cl_2 (1 mL), and trichloroacetonitrile (30.0 μL, 0.3 mmol) was added 1,8-diazabicyclo[5.4.0]undec-7-ene (3.0 μL, 0.02 mmol). The reaction was stirred and monitored by TLC. Upon consumption of alcohol, the crude mixture was concentrated was concentrated and immediately subjected to **GP3** with the following modifications: NaI instead of CsI as the iodide source, and a 50:50 mixture of PhMe/$CHCl_3$ instead of PhMe for solvent. After the tandem oxidation was complete, purification via column chromatography yielded oxazole **28** (32 mg, 62%) as a yellow solid.

One-pot Conversion of Alcohol to 2-Phenyl Oxazole

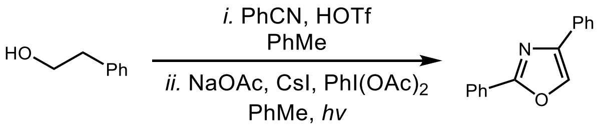

2-phenylethan-1-ol (24.0 μL, 0.2 mmol) was subjected to **GP1** for 24 hours. The crude was reduced under vacuum and immediately subjected to **GP3** with the following modification: addition of NaOAc (33 mg, 0.4 mmol) to effect the freebase *in situ*. After the tandem oxidation was complete, analysis of the crude by ^{1}H NMR with 1 equiv of 1,2-dichloroethane as an internal standard gave 45% yield. Purification via column chromatography yielded oxazole **3** (17.6 mg, 40%) as a white solid.

Modified Procedure 1: The crude imidate hydrotriflate salt from (*i*) was first filtered and residual solvent removed under vacuum. The washed salt was then subjected to **GP3** with the following modification: addition of NaOAc (33 mg, 0.4 mmol) to effect the freebase *in situ*. After the tandem oxidation was complete, purification via column chromatography yielded oxazole **3** (27.3 mg, 62%) as a white solid.

Modified Procedure 2: The crude imidate hydrotriflate salt from (*i*) was first filtered and residual solvent removed under vacuum. The washed salt was then free-based according to **GP1**. The resulting imidate was then subjected to **GP3**. After the tandem oxidation was complete, purification via column chromatography yielded oxazole **3** (35 mg, 78%) as a white solid.

VIII. Post-Synthetic Functionalization

General Procedure for Derivatization of Trichloromethyl Oxazoles (GP4)

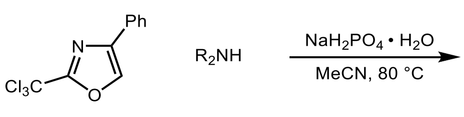

To a 2-dram vial equipped with a stir bar was added oxazole **28** (0.2 mmol, 1 equiv.), $NaH_2PO_4 \cdot H_2O$ (0.6 mmol, 3 equiv.), amine (0.8 mmol, 4 equiv.), and MeCN (0.1 M). The reaction was stirred at 80 °C and progress was monitored by TLC. Upon completion, the reaction was quenched with water and extracted with ethyl acetate. The combined organic layers were dried over Na_2SO_4, concentrated, and then purified via column chromatography (silica gel with ethyl acetate and hexanes).

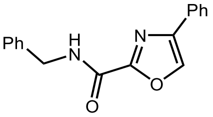

N-benzyl-4-phenyloxazole-2-carboxamide (31)

Oxazole **28** (44 mg, 0.2 mmol) and benzylamine (44 μL, 0.8 mmol) were subjected to **GP4.** Purification via column chromatography (silica gel, hexanes to 20% ethyl acetate/hexanes) yielded oxazole **31** (30 mg, 53%) as a white solid.

R_f: 0.26 (20% Ethyl acetate in hexanes)
^{1}H NMR (400 MHz, $CDCl_3$) δ: 8.04 (s, 1H), 7.74 – 7.71 (m, 2H), 7.44 – 7.31 (m, 9H), 4.67 (d, *J* = 6.1 Hz, 2H).
^{13}C NMR (100 MHz, $CDCl_3$) δ: 155.2, 142.0, 137.3, 136.1, 130.0, 129.04, 129.02, 128.9, 128.2, 128.0, 125.8, 43.8.
HRMS (ESI): m/z calculated for $C_{17}H_{14}N_2O_2Na$ $[M+Na]^+$: 301.0953, found 301.0933.
IR (neat): 3272, 3136, 3110, 3058, 3031, 2917, 1667, 1579, 1526, 1346, 1262, 1204, 1185, 938, 763, 722.
MP: 137 – 139 °C.

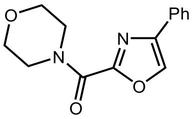

Morpholino(4-phenyloxazol-2-yl)methanone (32)

Oxazole **28** (44 mg, 0.2 mmol) and morpholine (68 μL, 0.8 mmol) were subjected to **GP4.** Purification via column chromatography (silica gel, hexanes to 20% ethyl acetate/hexanes) yielded oxazole **32** (37 mg, 71%) as a brown solid.

R_f: 0.15 (20% Ethyl acetate in hexanes)
^{1}H NMR (400 MHz, $CDCl_3$) δ: 8.02 (s, 1H), 7.75 – 7.73 (m, 2H), 7.45 – 7.41 (m, 2H), 7.38 – 7.34 (m, 1H), 4.38 – 4.35 (m, 2H), 3.85 – 3.79 (m, 6H).

^{13}C NMR (100 MHz, $CDCl_3$) δ: 155.4, 154.5, 141.5, 135.0, 130.2, 129.0, 128.9, 125.8, 67.2, 66.9, 47.7, 43.4.
HRMS (ESI): m/z calculated for $C_{14}H_{14}N_2O_3Na$ $[M+Na]^+$: 281.0902, found 281.0897.
IR (neat): 3140, 3115, 3026, 3018, 2963, 2922, 2853, 1635, 1533, 1430, 1271, 1171, 1110, 1021, 947.
MP: 82 – 84 °C.

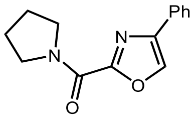

(4-phenyloxazol-2-yl)(pyrrolidin-1-yl)methanone (33)

Oxazole **28** (44 mg, 0.2 mmol) and pyrrolidine (66 μL, 0.8 mmol) were subjected to GP4. Purification via column chromatography (silica gel, 100% hexanes to 20% ethyl acetate/hexanes) yielded oxazole **33** (41 mg, 85%) as a yellow solid.

R_f: 0.15 (20% Ethyl acetate in hexanes)
^{1}H NMR (400 MHz, $CDCl_3$) δ: 8.01 (s, 1H), 7.78 – 7.76 (m, 2H), 7.45 – 7.41 (m, 2H), 7.37– 7.32 (m, 1H), 4.15 (t, J = 6.8 Hz, 2H), 3.72 (t, J = 6.9 Hz, 2H), 2.08 – 2.01 (m, 2H), 1.99 – 1.92 (m, 2H).
^{13}C NMR (100 MHz, $CDCl_3$) δ: 155.6, 154.8, 141.7, 134.9, 130.5, 129.0, 128.7, 125.8, 49.3, 47.4, 26.7, 24.0.
HRMS (ESI): m/z calculated for $C_{14}H_{14}N_2O_2Na$ $[M+Na]^+$: 269.0953, found 265.0955.
IR (neat): 3133, 3105, 3043, 2977, 2923, 2876, 1654, 1540, 1431, 1340, 1305, 1295, 1165, 1115, 1071, 943, 819, 759, 694, 679.
MP: 116 – 119 °C.

IX. Mechanistic Studies

Intermediacy of Oxazoline

Oxazoline **2** (45 mg, 0.2 mmol) was subjected to **GP3**. Upon completion, the crude mixture was quenched, concentrated, and quantified via ^{1}H NMR using 1 equiv of 1,2-dichloroethane as an internal standard.

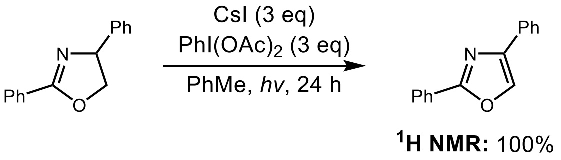

Additionally, the appearance and disappearance of oxazoline **2** under short time points (**Table S7**) and quantitative yield of oxazole **3** from **2** under our normal tandem reaction conditions strongly support oxazolines as an intermediate product en route to oxazoles.

Intermediacy of Di-iodide

2,2-diiodooctyl 2,2,2-trichloroacetimidate **36**[8] (21 mg, 0.04 mmol) was subjected to **GP3**, with the following modifications: NaI instead of CsI, and 1:1 PhMe/$CHCl_3$ instead of PhMe. Upon completion, the crude mixture was quenched, concentrated, and quantified via ^{1}H NMR using 1 equiv of 1,2-dichloroethane as an internal standard.

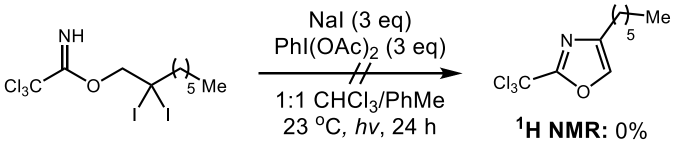

Analysis by ^{1}H NMR showed consumption of starting material but did not show a clear oxazole major product, and TLC confirms a complex mixture. Coupled with the absence of observed di-iodide in previous reactions, a mechanism invoking di-iodination followed by substitution and elimination to the oxazole is unlikely.

Intermolecular HAT Studies

Benzimidate **1** (45 mg, 0.2 mmol) was subjected to **GP3** with the following modifications, NaI was used instead of CsI, and 2,2,2-trifluoroethyl benzimidate (16.2 mg, 0.08 mmol) was added. Upon completion, the crude mixture was quenched, concentrated, and quantified via ^{1}H NMR using 1 equiv of 1,2-dichloroethane as an internal standard.

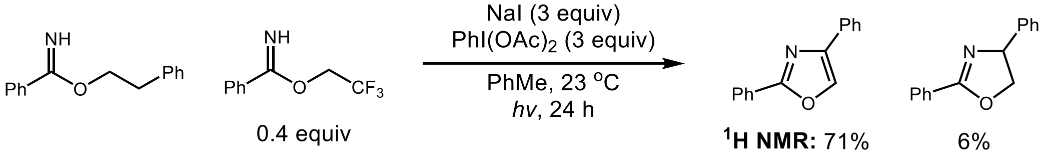

[8] Wappes, E. A.; Vanitcha, A.; Nagib, D. A. *Chem. Sci.*, **2018**, *9*, 4500 – 4504.

To probe if the second HAT event was mediated by residual imidate starting material, the TFE benzimidate was added in sub-stoichiometric quantities to the reaction mixture to observe if it had an effect on the efficiency of the oxazoline to oxazole transformation. The yields obtained are comparable to reactions without additional TFE benzimidate and thus does not support the hypothesis that the second HAT event is mediated by intermolecular imidate HAT.

Trapping of Non-aromatizable Oxazoline via HAT

Benzimidate **34** (19 mg, 0.08 mmol) was subjected to GP3**.** Purification via column chromatography yielded oxazoline **35** (84% via ^{1}H NMR with DCE as internal standard) as a clear oil.

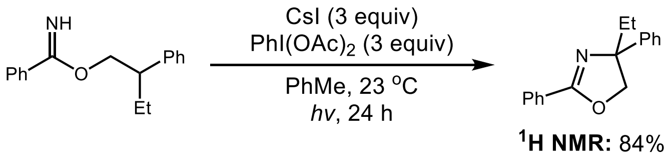

This substrate afforded only oxazoline **35**, with no additional functionalization at the α-oxygen position, indicating that HAT is not efficient at this position.

Deuterated Solvent Experiments

Oxazoline **2** (23 mg, 0.1 mmol) was subjected to GP3 with the following modifications, PhMe-*d8* was used instead of PhMe. Upon completion, the crude mixture was quenched, concentrated, and quantified via ^{1}H NMR using 1 equiv of 1,2-dichloroethane as an internal standard.

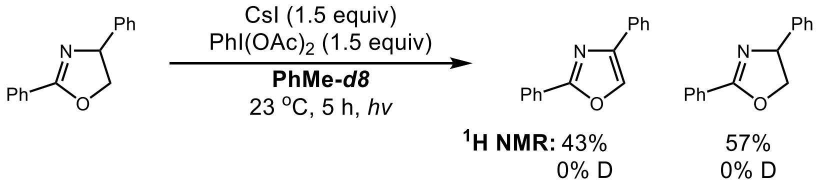

Oxazoline **2** (23 mg, 0.1 mmol) was subjected to GP3 with the following modifications, MeCN-*d3* was used instead of PhMe. Upon completion, the crude mixture was quenched, concentrated, and quantified via ^{1}H NMR using 1 equiv of 1,2-dichloroethane as an internal standard.

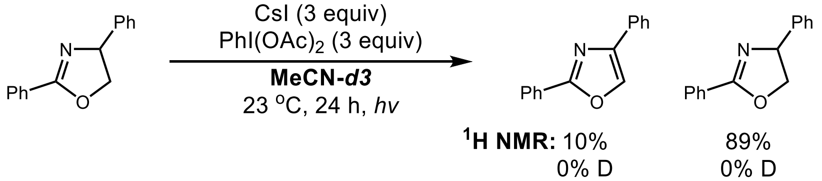

To explore the role of solvent as a hydrogen atom source, and therefore quenching any radical intermediates generated under our reaction conditions and suppressing the second HAT, we employed PhMe-*d8* and MeCN-*d3* to observe any possible deuterium trapping. No deuterium incorporation was observed in either reaction suggesting that solvent C-H bonds are not serving as a hydrogen atom source.

Benzylic Hydrogen Atom Transfer via Other Oxidants

Oxazoline **2** (23 mg, 0.1 mmol) and chloranil (49 mg, 0.2 mmol) were added to an 8 mL vial with stir bar. The vial was degassed and DCE (1 mL) was added. The reaction was then stirred for 15 h at 80 °C. The crude mixture was quenched with 1M NaOH and extracted with CH_2Cl_2, dried over $MgSO_4$ and concentrated. The reaction mixture was quantified via ^{1}H NMR using 1 equiv of 1,2-dichloroethane as an internal standard.

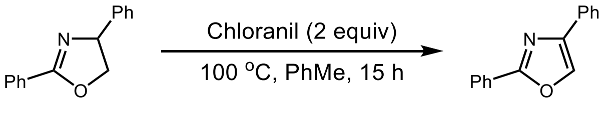

^{1}H NMR: 94%

To probe whether the conversion of the intermediate oxazolines to oxazoles occurs via an intermolecular HAT, we subjected oxazoline **2** to oxidants known to perform HAT at benzylic positions. Chloranil has been shown to oxidize benzylic C-H bonds, and it performs well in the aromatization of the intermediate oxazoline.

Additionally, DDQ at 50 °C in CH_2Cl_2 was found to decompose the oxazoline.

Steric Effects of Secondary Benzimidate

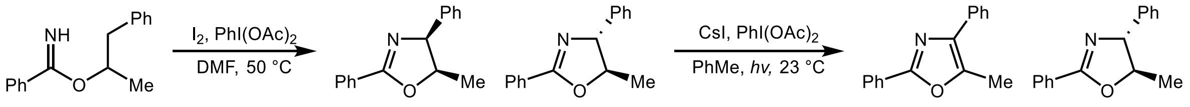

1.4 : 1 — **^{1}H NMR:** 58% — 30%

38 — *syn*/*anti* **39** — **40**

Benzimidate **38** (48 mg, 0.2 mmol) was added to an 8 mL vial and heated at 50 °C for 3 h with $PhI(OAc)_2$ (77 mg, 0.24 mmol) in 1 mL of an I_2 solution (0.01 M in DMF). The solvent was removed and residue was purified by column chromatography to yield a diastereomeric mixture of *syn*/*anti* oxazoline **39** (34 mg, 72%, dr = 1.4:1, *syn*:*anti*) as a clear oil. The mixture of *syn*/*anti* oxazoline **39** (31 mg, 0.13 mmol) was subjected to GP3. The reaction crude was quantified via ^{1}H NMR using 1,2-dichloroethane as an internal standard, yielding 58% oxazole **40** and 30% of *anti* oxazoline **39**.

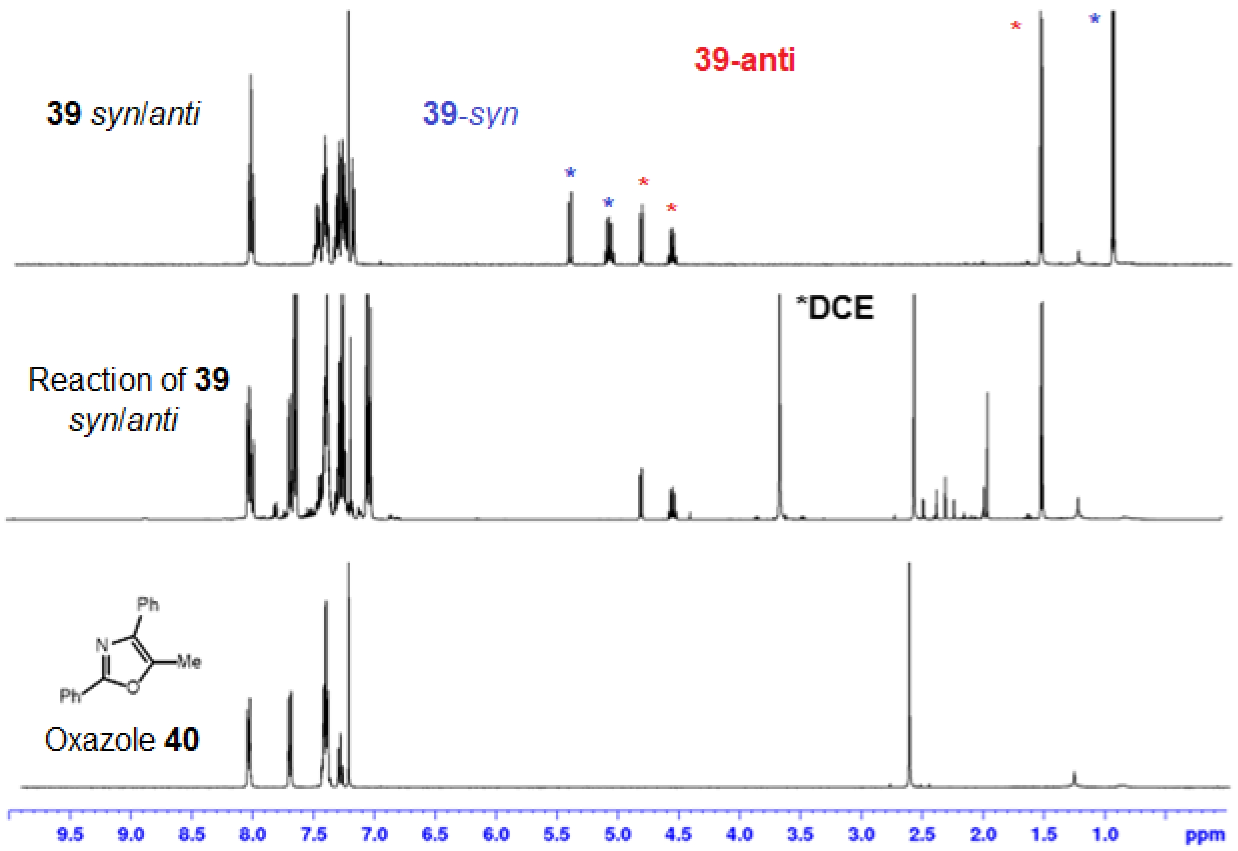

Figure S4. Top spectra is of the mixture of *syn*/*anti* **39** prior to subjection to GP3. Middle spectra is of the crude reaction after subjection to GP3**.** Bottom spectra is of independently synthesized oxazole 40**.**

X. Substrate Limitations

In the course of substrate exploration, the following substrates afforded oxazolines, but not oxazoles.

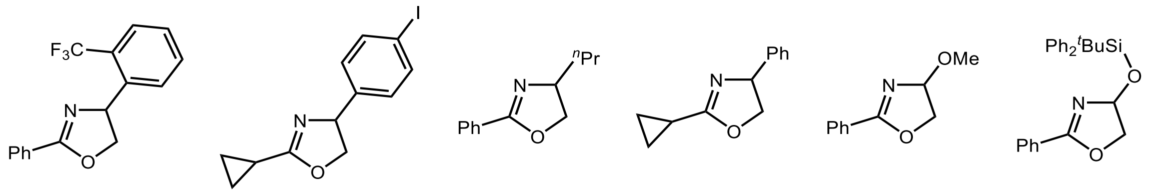

94% 73% 90% 76% 90% 94%

Figure S5. Examples of incomplete cascade reactions

XI. Commercial Availability of Feedstock Chemicals

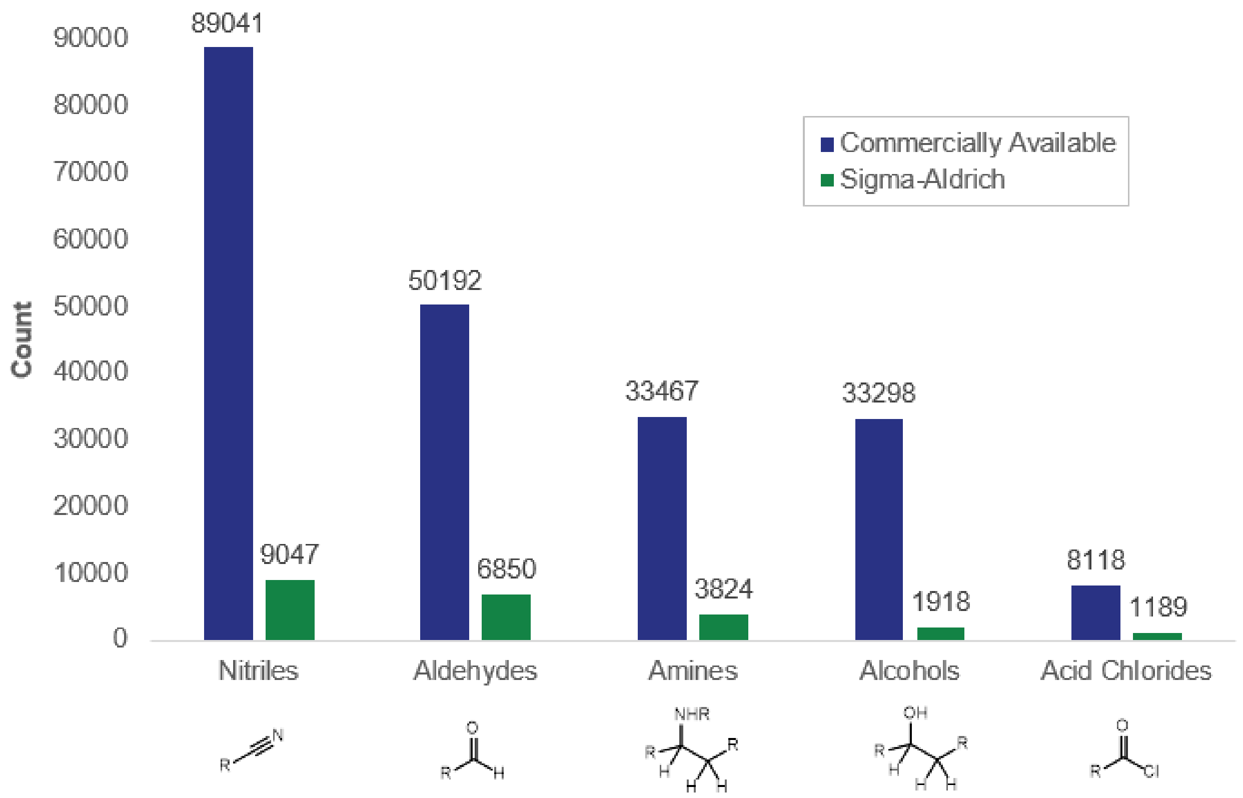

Figure S6. Commercial availability of feedstock chemicals

The above data were collected from Reaxys for the indicated structural motifs on 14 Mar 2019 with the following options: As Drawn, Stereo, Additional Ring Closures, Related Markush, Salts, Charges. The results were filtered by “product for purchase” and “SigmaAldrich”.

XII. Computational Studies

Computational Methods

All calculations were performed using the Gaussian 16 (revision A.03) suite of programs.[9] Geometry optimizations and frequency calculations were performed using the ωB97X-D functional[10] with the 6-311++G(d,p) basis set[11] (Def2-TZVPP for iodine),[12] and the default PCM solvation model for PhMe,[13] and an integration grid of "UltraFine" to help minimize uncertainty in computed free energies. All stationary points were confirmed to have no imaginary frequencies. Transition states were confirmed as first order saddle points with the presence of a single imaginary frequency, and scanned in both directions along their intrinsic reaction coordinate. Reported Gibbs free energies and enthalpies in solution include thermal corrections computed at 298.15 K and 1 atm. Visualization carried out with CYLview.[14]

Oxidation Potentials

The oxidation potentials of oxazole and imidazole were calculated according to the method described in the literature[15] using the B3LYP functional[16] with the 6-311++g(d,p) basis set, and the CPCM solvation model for MeCN.[17]

All calculated $\Delta E°_{1/2}$ are reported versus SCE in MeCN and are summarized in **Table S10**.

---

[9] Gaussian 16, Revision A.03, Frisch, M. J.; Trucks, G. W.; Schlegel, H. B.; Scuseria, G. E.; Robb, M. A.; Cheeseman, J. R.; Scalmani, G.; Barone, V.; Petersson, G. A.; Nakatsuji, H.; Li, X.; Caricato, M.; Marenich, A. V.; Bloino, J.; Janesko, B. G.; Gomperts, R.; Mennucci, B.; Hratchian, H. P.; Ortiz, J. V.; Izmaylov, A. F.; Sonnenberg, J. L.; Williams-Young, D.; Ding, F.; Lipparini, F.; Egidi, F.; Goings, J.; Peng, B.; Petrone, A.; Henderson, T.; Ranasinghe, D.; Zakrzewski, V. G.; Gao, J.; Rega, N.; Zheng, G.; Liang, W.; Hada, M.; Ehara, M.; Toyota, K.; Fukuda, R.; Hasegawa, J.; Ishida, M.; Nakajima, T.; Honda, Y.; Kitao, O.; Nakai, H.; Vreven, T.; Throssell, K.; Montgomery, J. A., Jr.; Peralta, J. E.; Ogliaro, F.; Bearpark, M. J.; Heyd, J. J.; Brothers, E. N.; Kudin, K. N.; Staroverov, V. N.; Keith, T. A.; Kobayashi, R.; Normand, J.; Raghavachari, K.; Rendell, A. P.; Burant, J. C.; Iyengar, S. S.; Tomasi, J.; Cossi, M.; Millam, J. M.; Klene, M.; Adamo, C.; Cammi, R.; Ochterski, J. W.; Martin, R. L.; Morokuma, K.; Farkas, O.; Foresman, J. B.; Fox, D. J. Gaussian, Inc., Wallingford CT, 2016.
[10] ωB97X-D Functional: Chai, J-D., Head-Gordon, M. *Phys. Chem. Chem. Phys.*, **2008,** *10*, 6615 – 6620.
[11] 6-311G Basis Set: Krishnan, R.; Binkley, J. S.; Seeger, R.; Pople, J. A. *J. Chem. Phys.* **1980,** *72*, 650 – 654.
[12] Def2-TZVPP Basis Set for Iodine: (a) Weigend, F.; Ahlrichs, R. *Phys. Chem. Chem. Phys.*, **2005,** 7, 3297 – 3305; (b) Schäefer, A.; Horn, H.; Ahlrichs, R. *J. Chem. Phys.*, **1992,** *97*, 2571 – 2577; (c) Schäefer, A.; Huber, C.; Ahlrichs, R. *J. Chem. Phys.*, **1994,** 100, 5829 – 5835 (d) Eichkorn, K.; Weigend, F.; Treutler, O.; Ahlrichs, R. *Theor. Chem. Acc.*, **1997,** *97*, 119 – 124; (e) Weigend, F.; Furche, F.; Ahlrichs, R. *J. Chem. Phys.* **2003,** *119*, 12753 – 12762; (f) Peterson, K. A.; Figgen, D.; Goll, E.; Stoll, H.; Dolg, M. *J. Chem. Phys.* **2003,** *119*, 11113 – 11123.
[13] PCM Solvation Model: Tomasi, J.; Mennucci, B.; Cammi, R. *Chem. Rev.*, **2005**, *105*, 2999 – 3094.
[14] CYLview: C. Y. Legault, CYLview, 1.0b. (Université de Sherbrooke, 2009); www.cylview.org.
[15] Calculation of Electrochemical Potentials: Roth, H. G.; Romero, N. A.; Nicewicz, D. A. *Synlett*. **2016,** *27*, 714 – 723.
[16] (a) Vosko, S. H.; Wilk, L.; Nusair, M. *Can. J. Phys.* **1980**, *58*, 1200 – 1211; (b) Lee, C.; Yang, W.; Parr, R. G. *Phys. Rev. B*, **1988**, *37*, 785 – 789 (c) Becke, A. D. *J. Chem. Phys.* **1993**, *98*, 5648 – 5652; (d) Stephens, P. J.; Devlin, F. J.; Chabalowski, C. F.; Frisch, M. J. J. Phys. Chem. **1994**, *98*, 11623 – 11627.
[17] CPCM Solvation Model: (a) Klamt, A.; Schüürmann, G. *J. Chem. Soc., Perkin Trans. 2*, **1993**, *0*, 799 – 805 (b) Barone, V.; Cossi, M. *J. Phys. Chem. A*, **1998**, *102*, 1995 – 2001.

Notes: EE = sum of electronic energies, conversion factor: 1 hartree = 627.509 kcal/mol = 27.2114 eV

Table S10. Oxidation potentials of oxazole and imidazole

Structure	$\Delta G°_{Neutral}$ (hartree)	$\Delta G°_{Oxidized}$ (hartree)	$\Delta G°_{1/2}$ (kcal/mol)	$\Delta E°_{1/2}$ Calc. (V)	HOMO (hartree)	HOMO (eV)	LUMO (hartree)	LUMO (eV)
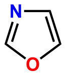	-246.114858	-245.855263	-162.8981989	2.64	-0.27	-7.27	-0.02	-0.63
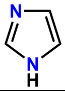	-226.249238	-226.013029	-148.22372734	2.01	-0.24	-6.66	0.00	-0.09

Reaction Modeling

Activation energies ($\Delta G^{\ddagger}$) were calculated as the difference in free energies between the optimized transition state and its preceding ground state. Reaction energies ($\Delta G°$) were calculated as the difference between the free energies of the product(s) and reactant(s). In the case of the bimolecular 2nd HAT reactions, the products and reactants were treated separately to properly account for entropy. These are summarized in **Table S11** and all relevant energies for these calculations are contained in **Table S12**.

Table S11. Activation ($\Delta G^{\ddagger}$) and Reaction ($\Delta G°$) Energies for Hydrogen Atom Transfers (in kcal/mol)

Reaction	Scheme	$\Delta G^{\ddagger}$	$\Delta G°$
1,4 HAT	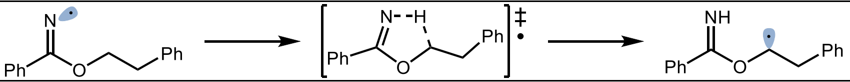	19.4	0.1
1,5 HAT	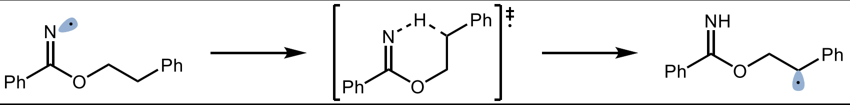	14.3	-6.2
2nd HAT (•OAc)	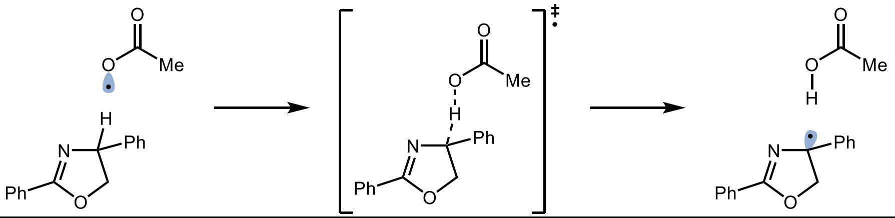	8.3	-33.9
2nd HAT (•Me)	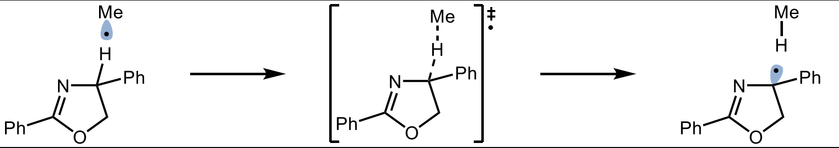	11.9	-30.0
2nd HAT (•I)	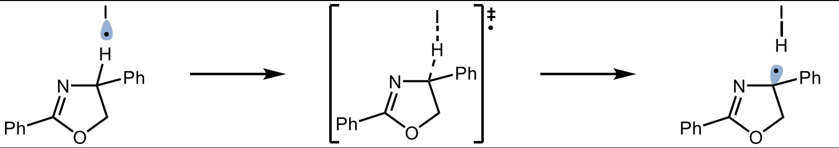	6.0	-5.0

2nd HAT (•Im)	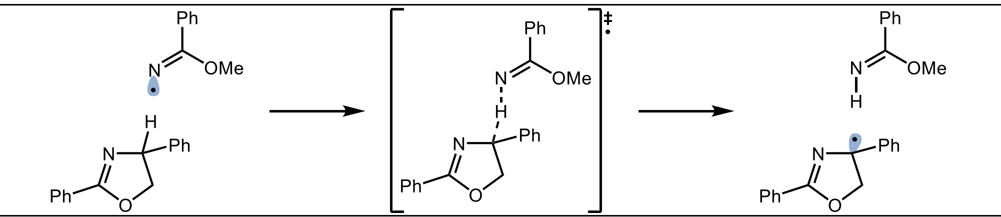	15.9	-22.0
2nd HAT at C-5	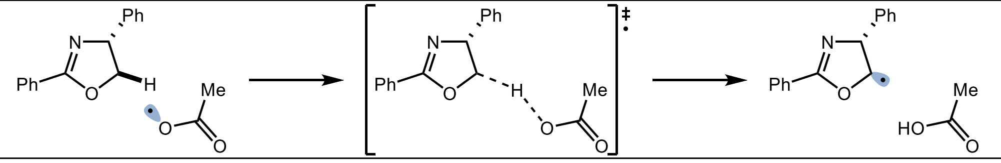	12.4	-12.3

Table S12. Computed Free Energies of Reaction Intermediates and Transition States

Entry	Species	ΔG° (hartree)	ΔG° (kcal/mol)
Intermediate I	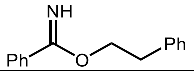	-710.322025	-445733.4636
Intermediate II	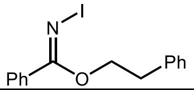	-1007.490338	-632209.2545
Intermediate III	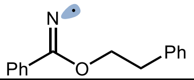	-709.670713	-455324.7594
Transition State IV-TS(1,5)	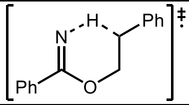	-709.647874	-44531.4278
Intermediate IV	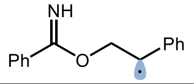	-709.680539	-445330.9253
Intermediate V	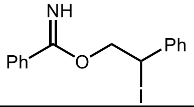	-1007.518019	-632226.6246
Intermediate VI	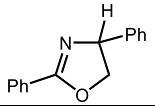	-709.131391	-444986.3300
Transition State VII-TS(OAc)	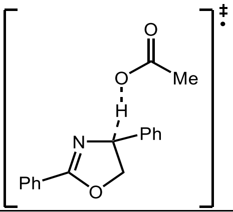	-937.502842	-588291.4709
Intermediate VII	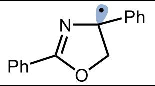	-708.514596	-444599.2856
Intermediate VIII	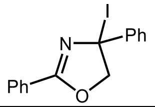	-1006.329815	-631481.0159
Intermediate IX	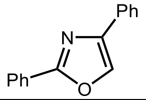	-707.945636	-444242.2581
**Intermediates and Transition States for Alternate Pathways**			
Transition State IV-TS(1,4)	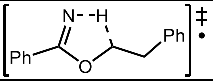	-709.639839	-445305.3857
Intermediate IV(1,4)	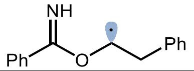	-709.670556	-445324.6609
Transition State VII-TS(Me)	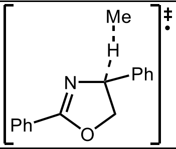	-748.93616	-469964.1808

Transition State VII-TS(I)	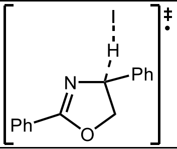	-1006.899787	-631838.6784
Transition State VII-TS(Im)	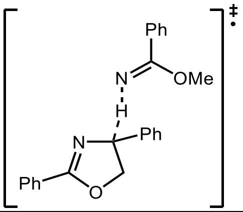	-1148.533376	-720715.0302
Transition State VII-TS(α-O)	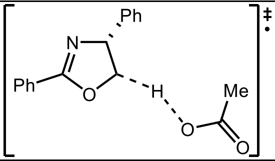	-937.496431	-588287.4479
Intermediate VII(α-O)	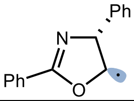	-708.480227	-444577.7188

Bond dissociation free energies were calculated as the difference in free energies between the sum of radical products resulting from hemolytic cleavage of the indicated bond and the non-cleaved starting material. Multiple combinations of common functionals and solvent models were used to provide a range of bond dissociation free energies (**Table S13**).

Table S13. Computed Bond Dissociation Free Energies

		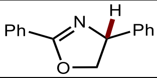	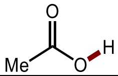	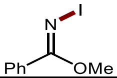	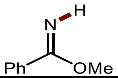
**Functional**	**Solvent**	**BDFE (kcal/mol)**	**BDFE (kcal/mol)**	**BDFE (kcal/mol)**	**BDFE (kcal/mol)**
B3LYP	PCM-PhMe	60.3	94.8	17.7	85.1
ωB97x-D	PCM-PhMe	64.8	98.7	23.0	86.8
M06-2x	SMD-PhMe	65.4	103.8	28.3	88.8

Global and Local Radical Philicities

The global and local radical electrophilicities were calculated according to the method described in the literature.[18]

Geometries for each of the radical species were first optimized using the ωB97X-D functional, 6-311++g(d,p) basis set, and the PCM solvation model for PhMe. From these optimized geometries, electronic energies were then determined at N, N+1, and N-1 electron counts without additional geometry optimization, which are summarized in **Table S15**. The global and local radical electrophilicities, along with the electronic properties from which they are derived, are summarized in **Table S14**.

[18] Calculation of Radical Philicites: De Vleeschouwer, F.; Van Speybroeck, V.; Waroquier, M.; Geerlings, P.; De Proft, F. *Org. Lett.* **2007,** *9*, 2721 – 2724.

Radical	*I* (eV)	*EA* (eV)	*χ* (eV)	*μ* (eV)	*η* (eV)	ω (eV)	ρ_{N+1}	ρ_N	f^+	ω^+_{rc} (eV)
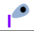	10.30	4.71	7.51	-7.51	5.59	**5.04**	54	53	1	**5.04**
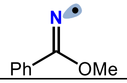	8.86	2.49	5.67	-5.67	6.37	**2.53**	7.914	7.306	0.608	**1.54**
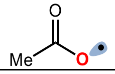	11.24	4.15	7.70	-7.70	7.09	**4.18**	8.836	8.402	0.434	**1.81**
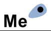	7.91	1.53	4.72	-4.72	6.38	**1.75**	7.471	6.477	0.994	**1.73**
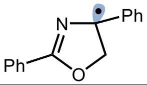	5.05	1.96	3.51	-3.51	3.09	**1.99**	6.044	5.807	0.237	**0.47**

Table S14. Computed global and local electrophilicities; electron densities *ρ* refer to highlighted atom; **key:** *I* = Ionization Energy, *EA* = Electron Affinity, *χ* = Electronegativity, *μ* = Electronic Chemical Potential, *η* = Chemical Hardness, *ω* = Global Electrophilicity, ρ_{N+1} = Electron Population of N+1 Electron Species, ρ_N = Electron Population of N-Electron Species, f^+ = Fukui Index, ω^+_{rc} = Local Electrophilicity

	EE (ωB97X-D, PCM(PhMe)) (hartrees)		
Radical	N Electrons	N+1 Electrons	N-1 Electrons
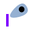	-297.760405	-297.933519	-297.381876
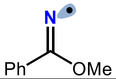	-439.535245	-439.626818	-439.209753
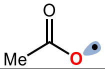	-228.404778	-228.557343	-227.991619
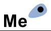	-39.835237	-39.891477	-39.544668
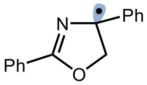	-708.957361	-709.003249	-708.738938

Table S15. Calculated electronic energies of N, N+1, and N-1 electron count species from previously optimized molecular geometries

Optimized Cartesian Coordinates

Note: Oxazole (N electrons), Oxazole (N-1 electrons), Imidazole (N electrons), and Imidazole (N-1 electrons) molecular geometries were all optimized at the B3LYP/6-311++g(d,p)/CPCM(MeCN) level of theory. All other molecular geometries optimized at the ωB97X-D/6-311++g(d,p)/Def2-TZVPP (for I and Cs)/CPCM(PhMe) level of theory, except for $PhI(OAc)_2$, PhI, CsOAc, and CsI which were calculated in the gas phase.

Oxazole (N electrons)

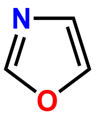

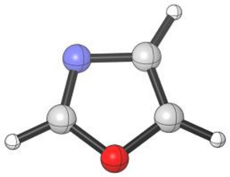

**Total EE (hartree)**	-246.146950
**EE + Zero-Point Energy Correction (hartree)**	-246.088689
**EE + Thermal Enthalpy Correction (hartree)**	-246.084189
**EE + Thermal Free Energy Correction (hartree)**	-246.114858

Cartesian Coordinates

C	1.10535000	0.05493300	0.00000000
C	-0.91874300	0.70670400	-0.00000100
C	-0.93600700	-0.64514800	0.00000100
O	0.36574700	-1.07915300	0.00000100
H	2.17743100	-0.05921500	0.00000000
H	-1.74846900	1.39439600	0.00000400
H	-1.70227200	-1.39997300	-0.00000500
N	0.40624800	1.14272600	0.00000000

Oxazole (N-1 electrons)

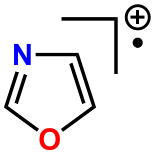

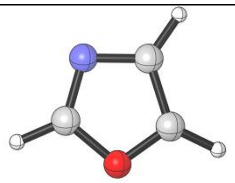

**Total EE (hartree)**	-245.885348
**EE + Zero-Point Energy Correction (hartree)**	-245.828347
**EE + Thermal Enthalpy Correction (hartree)**	-245.823674
**EE + Thermal Free Energy Correction (hartree)**	-245.855263
**Imaginary Frequencies (cm^{-1})**	0
**<S^2>**	0.7501

Cartesian Coordinates

C	1.08999800	0.15561600	0.00000000
C	-0.95469400	0.66680400	0.00000000
C	-0.84021900	-0.76710000	0.00000000
O	0.44549800	-1.05895800	0.00000000
H	2.16974100	0.13769800	-0.00000100
H	-1.86412400	1.24924300	0.00000000
H	-1.56159500	-1.57133000	-0.00000100
N	0.27449700	1.18916100	0.00000000

Imidazole (N electrons)

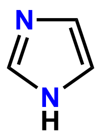

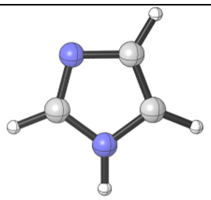

Total EE (hartree)	-226.294102
EE + Zero-Point Energy Correction (hartree)	-226.222991
EE + Thermal Enthalpy Correction (hartree)	-226.218329
EE + Thermal Free Energy Correction (hartree)	-226.249238
Imaginary Frequencies (cm^{-1})	0

Cartesian Coordinates

C	0.99430800	-0.53387200	0.00000000
C	-1.14006400	-0.28537000	0.00000000
C	-0.61370400	0.98075400	0.00000000
H	1.99355000	-0.94094600	0.00000000
H	-2.18035700	-0.57161000	0.00000200
H	-1.06697300	1.95751900	-0.00000200
N	-0.12864200	-1.22601400	0.00000000
N	0.75186800	0.80462200	0.00000000
H	1.44796200	1.53570300	0.00000100

Imidazole (N-1 electrons)

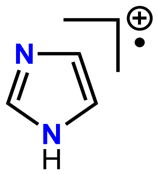

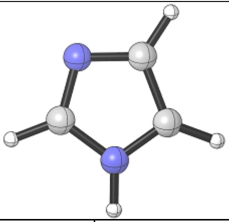

Total EE (hartree)	-226.056427
EE + Zero-Point Energy Correction (hartree)	-225.986070
EE + Thermal Enthalpy Correction (hartree)	-225.982261
EE + Thermal Free Energy Correction (hartree)	-226.013029
Imaginary Frequencies (cm^{-1})	0
$\langle S^2 \rangle$	0.7501

Cartesian Coordinates

C	0.29413900	-1.07980000	0.00000000
C	-1.02436700	0.58785200	0.00000000
C	0.33486700	1.11034000	0.00000000
H	0.68751000	-2.08531000	0.00000000
H	-1.93214400	1.17268500	0.00000000
H	0.70627800	2.12358500	0.00000000
N	-1.00152600	-0.73922100	0.00000000
N	1.11323000	0.03292600	0.00000000
H	2.12859400	0.02275200	-0.00000100

Intermediate I

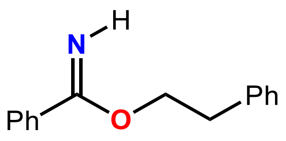

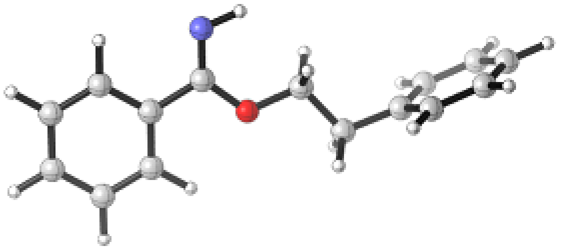

**Total EE (hartree)**	-710.544074
**EE + Zero-Point Energy Correction (hartree)**	-710.276987
**EE + Thermal Enthalpy Correction (hartree)**	-710.261489
**EE + Thermal Free Energy Correction (hartree)**	-710.322025
**Imaginary Frequencies (cm^{-1})**	0

Cartesian Coordinates

C	-1.59941400	0.87674900	0.02713600
N	-1.44212200	2.13625900	0.05752200
H	-0.46690700	2.41897100	0.06519600
O	-0.63434200	-0.06775300	0.00735200
C	0.72624800	0.34735400	0.01707800
H	0.93134400	0.97269100	-0.85986800
H	0.93116300	0.93346000	0.92067600
C	1.59303000	-0.90262900	-0.01078200
H	1.35385400	-1.51818900	0.86025400
H	1.34606800	-1.48345300	-0.90317900
C	-2.95886100	0.26753900	0.00852000
C	-4.07805800	1.10108800	-0.02350000
C	-3.13437900	-1.11620500	0.02310600
C	-5.35410100	0.55733900	-0.04002600
H	-3.92920800	2.17372700	-0.03589500
C	-4.41448300	-1.65795100	0.00772400
H	-2.26939800	-1.76617400	0.04724100
C	-5.52577800	-0.82406400	-0.02396800
H	-6.21787100	1.21199000	-0.06560400
H	-4.54239700	-2.73445900	0.02033900
H	-6.52365500	-1.24821200	-0.03650000
C	3.05602800	-0.53339400	-0.01033000
C	3.74371700	-0.35263000	1.18974400
C	3.73583100	-0.31976600	-1.20947200
C	5.07987200	0.03141100	1.19296600
H	3.22841200	-0.51940000	2.13097300
C	5.07198900	0.06434200	-1.21098300
H	3.21424500	-0.46069200	-2.15146000
C	5.74769900	0.24150400	-0.00858500
H	5.60081300	0.16335500	2.13478500
H	5.58670100	0.22205100	-2.15227200
H	6.79038700	0.53842500	-0.00794900

Intermediate II

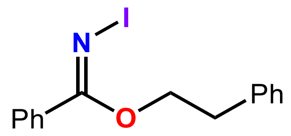

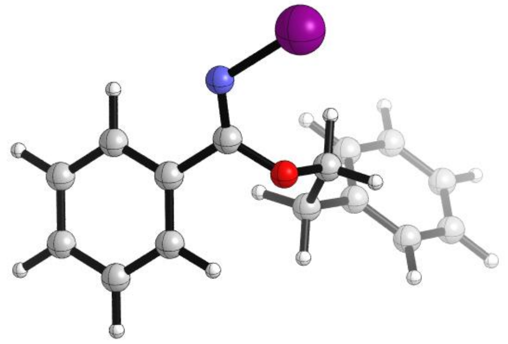

**Total EE (hartree)**	-1007.697171
**EE + Zero-Point Energy Correction (hartree)**	-1007.441082
**EE + Thermal Enthalpy Correction (hartree)**	-1007.423996
**EE + Thermal Free Energy Correction (hartree)**	-1007.490338
**Imaginary Frequencies (cm^{-1})**	0

Cartesian Coordinates

C	1.37743000	-0.36656300	0.27645400
N	2.13575300	0.40860600	-0.38997700
O	0.49875300	0.01899400	1.23626100
C	-0.81509500	0.40433000	0.78984100
H	-1.21701400	1.01298000	1.60031100
H	-0.72995600	1.03778300	-0.09869500
C	-1.72288500	-0.79060400	0.51002300
H	-1.29307500	-1.40430000	-0.28592200
H	-1.78301100	-1.41304900	1.40663500
C	1.50148500	-1.83603600	0.06736000
C	2.09416500	-2.33966300	-1.09282700
C	1.03192600	-2.71853600	1.04058400
C	2.20532200	-3.70965700	-1.27768200
H	2.45849900	-1.64885200	-1.84305200
C	1.15475800	-4.08986900	0.85562600
H	0.57784500	-2.32623600	1.94157900
C	1.73756300	-4.58772800	-0.30375800
H	2.65790600	-4.09481300	-2.18421900
H	0.79398400	-4.76975600	1.61888200
H	1.82748300	-5.65825600	-0.44986200
C	-3.09478800	-0.30880600	0.10884500
C	-3.39312000	-0.04885100	-1.22879600
C	-4.07395000	-0.06543400	1.07208300
C	-4.64160700	0.43930900	-1.59698000
H	-2.64142000	-0.23594000	-1.98983400
C	-5.32393200	0.42223200	0.70822700
H	-3.85654800	-0.26516000	2.11712000
C	-5.61085400	0.67650900	-0.62846500
H	-4.85848300	0.63173600	-2.64185000
H	-6.07546400	0.60112700	1.46918600
H	-6.58591600	1.05460700	-0.91426600
I	2.02313900	2.41475700	-0.05112200

Intermediate III

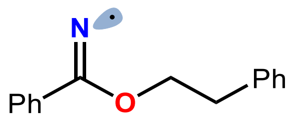

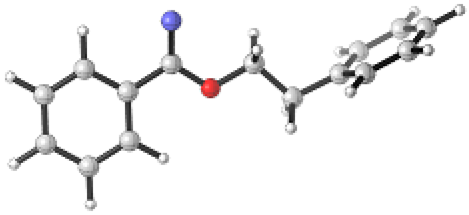

**Total EE (hartree)**	-709.877971
**EE + Zero-Point Energy Correction (hartree)**	-709.624149
**EE + Thermal Enthalpy Correction (hartree)**	-709.608567
**EE + Thermal Free Energy Correction (hartree)**	-709.670713
**Imaginary Frequencies (cm^{-1})**	0
**$<S^2>$**	0.7501

Cartesian Coordinates

C	-1.58825000	0.79078200	0.18730100
N	-1.32717300	1.98030200	0.53164600
O	-0.64012300	-0.12099500	-0.08856800
C	0.72042900	0.31060400	0.05365400
H	0.90543300	1.15116700	-0.62275200
H	0.88537700	0.65243300	1.08038300
C	1.61103000	-0.87333300	-0.28344400
H	1.37135900	-1.70041800	0.39033500
H	1.39185500	-1.20366200	-1.30227800
C	-2.97782500	0.27160300	0.06621100
C	-4.05096900	1.16284600	0.02107500
C	-3.21072100	-1.10157100	0.00896200
C	-5.34799900	0.68030600	-0.07564200
H	-3.86458900	2.22994000	0.05590200
C	-4.51221200	-1.57870100	-0.08802500
H	-2.37510800	-1.78909400	0.04069200
C	-5.58090600	-0.69082100	-0.12967800
H	-6.17889200	1.37523200	-0.11311300
H	-4.69045200	-2.64702200	-0.13119400
H	-6.59519800	-1.06584400	-0.20685300
C	3.06691100	-0.49719700	-0.15511800
C	3.72911700	-0.62445000	1.06587300
C	3.76500400	0.02642400	-1.24311100
C	5.05864100	-0.24036800	1.19739700
H	3.19885500	-1.03259800	1.92111900
C	5.09467400	0.41191900	-1.11612500
H	3.26297200	0.12910100	-2.20047000
C	5.74530900	0.27950800	0.10562800
H	5.55952200	-0.34975500	2.15289700
H	5.62380100	0.81357700	-1.97318300
H	6.78287400	0.57747900	0.20578600

Transition State IV-TS(1,5)

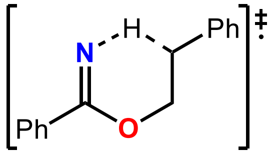	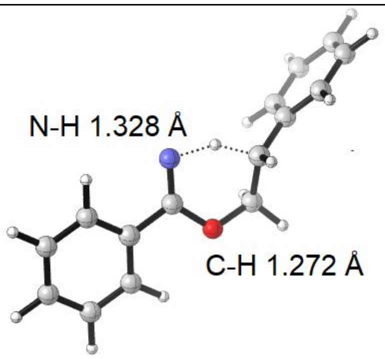

Total EE (hartree)	-709.853471
EE + Zero-Point Energy Correction (hartree)	-709.604410
EE + Thermal Enthalpy Correction (hartree)	-709.589756
EE + Thermal Free Energy Correction (hartree)	-709.647874
Imaginary Frequencies (cm^{-1})	1, -1695.14
<S^2>	0.7503

Cartesian Coordinates

C	-1.23359400	-0.06225500	-0.15321000
N	-0.51019200	-1.10269000	-0.09668000
O	-0.77673700	1.20318000	-0.33924300
C	0.62338300	1.40644700	-0.12792200
H	0.83408700	2.38792500	-0.55653100
H	0.79972600	1.44996000	0.95105600
C	1.44114600	0.31260800	-0.77149600
H	0.69651200	-0.66205700	-0.43347700
H	1.36834600	0.32736000	-1.86029100
C	-2.71673200	-0.11713300	-0.02745600
C	-3.37116300	-1.34558800	-0.13379600
C	-3.45552600	1.04256500	0.20439300
C	-4.75060300	-1.41148300	-0.00422900
H	-2.79005500	-2.24090100	-0.31972500
C	-4.83752000	0.97141000	0.33342000
H	-2.94613300	1.99431100	0.28374600
C	-5.48660300	-0.25324800	0.22980800
H	-5.25402400	-2.36767200	-0.08947900
H	-5.40740000	1.87544600	0.51539800
H	-6.56496700	-0.30642400	0.32944800
C	2.81797900	0.07914900	-0.28364100
C	3.11675300	0.01420300	1.08383600
C	3.85759100	-0.12484200	-1.19871400
C	4.41270600	-0.22864800	1.51712500
H	2.32719800	0.13298200	1.81786900
C	5.15426700	-0.36724600	-0.76509400
H	3.64390000	-0.08700500	-2.26205400
C	5.43809700	-0.41720000	0.59541900
H	4.62231900	-0.27847900	2.57979800
H	5.94510700	-0.51705600	-1.49163400
H	6.44953100	-0.60744200	0.93592100

Intermediate IV

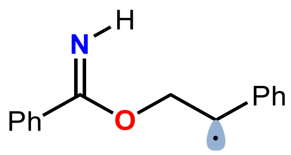

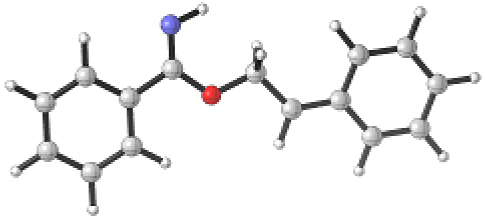

**Total EE (hartree)**	-709.888354
**EE + Zero-Point Energy Correction (hartree)**	-709.635504
**EE + Thermal Enthalpy Correction (hartree)**	-709.620023
**EE + Thermal Free Energy Correction (hartree)**	-709.680539
**Imaginary Frequencies (cm^{-1})**	0
**<S^2>**	0.7512

Cartesian Coordinates

C	-1.67952200	-0.96649600	0.03037400
O	-0.63277200	-0.11215900	0.05898500
C	0.68211400	-0.64847800	0.07800900
H	0.80746400	-1.29039200	0.96374300
H	0.83764100	-1.27682800	-0.81157900
C	1.63829200	0.49084000	0.10418900
H	1.21187300	1.48376000	0.19131200
C	-2.97599000	-0.23390400	-0.00053000
C	-4.16826500	-0.95942500	0.01906100
C	-3.02129000	1.15939000	-0.05019600
C	-5.38803400	-0.29958800	-0.01049300
H	-4.12093500	-2.04059500	0.05715800
C	-4.24524300	1.81745700	-0.08096000
H	-2.09945800	1.72603400	-0.06592400
C	-5.42973100	1.09093600	-0.06078000
H	-6.30932200	-0.87076100	0.00574400
H	-4.27198800	2.90050400	-0.12063900
H	-6.38378600	1.60572800	-0.08413000
C	3.03780600	0.33369700	0.03984800
C	3.65753900	-0.93857400	-0.06519900
C	3.88409900	1.47240900	0.08056200
C	5.03459800	-1.05466500	-0.12541100
H	3.04961700	-1.83623800	-0.09821400
C	5.25744000	1.34322400	0.01922700
H	3.43382700	2.45634500	0.16111000
C	5.84489000	0.08002900	-0.08425700
H	5.48596200	-2.03740500	-0.20533100
H	5.88244300	2.22870200	0.05174000
H	6.92312300	-0.01801600	-0.13209200
H	-0.69385200	-2.60846000	0.05424800
N	-1.63752200	-2.23517400	0.02861500

Transition State IV-TS(1,4)

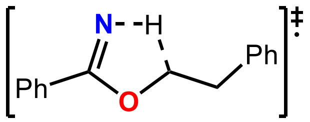

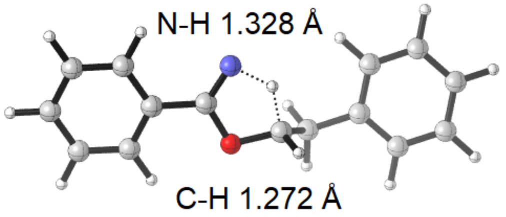

**Total EE (hartree)**	-709.853471
**EE + Zero-Point Energy Correction (hartree)**	-709.604410
**EE + Thermal Enthalpy Correction (hartree)**	-709.589756
**EE + Thermal Free Energy Correction (hartree)**	-709.647874
**Imaginary Frequencies (cm^{-1})**	1, -1695.14
**<S^2>**	0.7617

Cartesian Coordinates

C	-1.37871600	0.24434400	0.34905100
N	-0.73164900	1.33108400	0.51886600
O	-0.69224000	-0.92460500	0.36309200
C	0.66707100	-0.55824500	0.57031900
H	0.39956000	0.75478800	0.64640300
H	1.02253100	-0.88080200	1.54906300
C	1.55223900	-0.86795800	-0.60394700
H	1.55980200	-1.95281800	-0.76622600
H	1.11882800	-0.41447700	-1.50108600
C	-2.83367300	0.13375000	0.12974400
C	-3.61493000	1.29053000	0.10443100
C	-3.43166100	-1.11315400	-0.05164100
C	-4.98302600	1.19801900	-0.10197200
H	-3.14064600	2.25436300	0.24704600
C	-4.80302800	-1.20010800	-0.25762200
H	-2.82291200	-2.00831900	-0.03077900
C	-5.57909400	-0.04721200	-0.28314700
H	-5.58697400	2.09780600	-0.12128700
H	-5.26528800	-2.17038600	-0.39809400
H	-6.64902700	-0.11737300	-0.44391000
C	2.95573800	-0.36129700	-0.37424600
C	3.97551100	-1.22473200	0.02077900
C	3.24201500	0.99681700	-0.51876800
C	5.25825500	-0.74380800	0.26316300
H	3.76596200	-2.28378000	0.13605000
C	4.52183500	1.48043700	-0.27735300
H	2.45503600	1.67949900	-0.82490400
C	5.53427400	0.61016600	0.11454200
H	6.04230500	-1.42878100	0.56630800
H	4.72968300	2.53788900	-0.39678500
H	6.53375300	0.98635500	0.30141400

Intermediate IV (1,4)

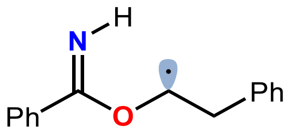

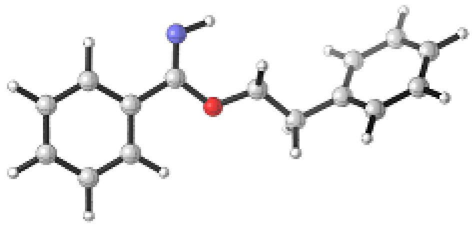

**Total EE (hartree)**	-709.876951
**EE + Zero-Point Energy Correction (hartree)**	-709.624155
**EE + Thermal Enthalpy Correction (hartree)**	-709.608552
**EE + Thermal Free Energy Correction (hartree)**	-709.670556
**Imaginary Frequencies (cm^{-1})**	0
**<S^2>**	0.7544

Cartesian Coordinates

C	-1.55706600	-0.79828600	-0.15418200
O	-0.62546400	0.15165900	0.16514000
C	0.68948700	-0.08096200	-0.11961300
H	0.90520000	-0.54445700	-1.07991500
C	-2.93708400	-0.25832900	-0.06702600
C	-4.01656200	-1.12373000	-0.25593000
C	-3.17527800	1.09115000	0.19491300
C	-5.31567800	-0.64380800	-0.18467400
H	-3.81995100	-2.16941400	-0.45796900
C	-4.47882400	1.56805000	0.26610700
H	-2.34148900	1.76537000	0.34204300
C	-5.55012700	0.70350000	0.07642700
H	-6.14862300	-1.32171600	-0.33221900
H	-4.65626400	2.61795600	0.46941900
H	-6.56643600	1.07726000	0.13220600
C	3.07013700	0.47963700	0.25807900
C	3.86069400	1.17459600	-0.65411600
C	3.61805500	-0.61908300	0.92162100
C	5.17404700	0.78424300	-0.89834800
H	3.44675200	2.03055500	-1.17809100
C	4.92867700	-1.01157900	0.68109700
H	3.01044200	-1.16838800	1.63428800
C	5.71112200	-0.30986000	-0.23121900
H	5.77697900	1.33709100	-1.61014700
H	5.34136100	-1.86512600	1.20741300
H	6.73452900	-0.61451300	-0.41894500
H	-0.31845400	-2.20980400	-0.44076400
N	-1.31298000	-1.99697100	-0.47777000
C	1.63495000	0.87962500	0.50841000
H	1.43298800	0.92527500	1.58497600
H	1.45823100	1.89444100	0.12170300

Intermediate V

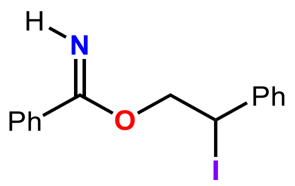

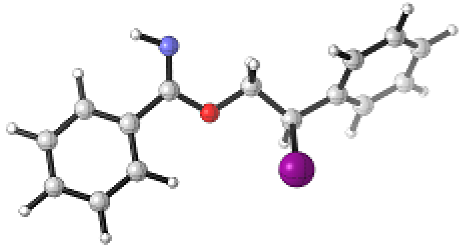

**Total EE (hartree)**	-1007.730364
**EE + Zero-Point Energy Correction (hartree)**	-1007.473415
**EE + Thermal Enthalpy Correction (hartree)**	-1007.456462
**EE + Thermal Free Energy Correction (hartree)**	-1007.521880
**Imaginary Frequencies (cm^{-1})**	0
**<S^2>**	N/A

Cartesian Coordinates

C	-2.05500800	1.43120500	0.35402200
O	-1.04004600	0.75779200	-0.21332600
C	0.27836200	1.16831000	0.11455800
H	0.46860300	2.16366600	-0.30123500
H	0.39092000	1.23080500	1.19924800
C	1.24312400	0.18291600	-0.51713800
H	0.99383400	0.06409900	-1.57043100
C	-3.36538400	0.80215600	0.02762800
C	-4.52919000	1.57088000	0.01049100
C	-3.44001900	-0.56397100	-0.24733100
C	-5.75437100	0.98033400	-0.26783300
H	-4.48008400	2.63902500	0.19358800
C	-4.66833200	-1.15379600	-0.51623400
H	-2.53465500	-1.15876500	-0.24110300
C	-5.82647500	-0.38421500	-0.52741000
H	-6.65226600	1.58708200	-0.28799900
H	-4.72029800	-2.21740700	-0.71875700
H	-6.78367900	-0.84558800	-0.74251900
C	2.67878900	0.59985800	-0.36908600
C	3.22020800	0.93252000	0.87466000
C	3.48724400	0.68772900	-1.50147800
C	4.53925500	1.35062700	0.97907600
H	2.61504200	0.84928100	1.77112000
C	4.80995800	1.10332700	-1.39734700
H	3.07918600	0.42804600	-2.47297200
C	5.33859700	1.43739600	-0.15692600
H	4.94649100	1.60520900	1.95093600
H	5.42598300	1.16545100	-2.28723000
H	6.36971700	1.76130900	-0.07325600
I	0.92453400	-1.81525800	0.28628200
N	-1.83418200	2.45471500	1.07152200
H	-2.69998300	2.81944700	1.45571400

Intermediate VI

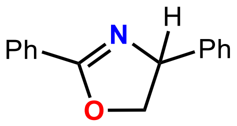

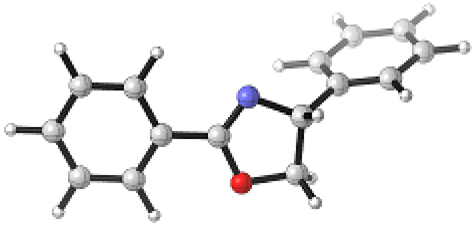

**Total EE (hartree)**	-709.333851
**EE + Zero-Point Energy Correction (hartree)**	-709.088294
**EE + Thermal Enthalpy Correction (hartree)**	-709.074122
**EE + Thermal Free Energy Correction (hartree)**	-709.131391
**Imaginary Frequencies (cm^{-1})**	0

Cartesian Coordinates

C	-0.95745000	0.57847500	-0.20519900
N	-0.01901400	0.03722600	-0.86962000
O	-0.68908300	1.72449000	0.46408900
C	0.72141300	1.95660300	0.31042000
H	1.19964400	1.81199100	1.28177300
H	0.86791000	2.98283100	-0.02417200
C	-2.33597000	0.07225400	-0.09378800
C	-3.27872200	0.73835600	0.68884000
C	-2.69632800	-1.09055200	-0.77743900
C	-4.57369000	0.24350600	0.78566700
H	-2.99526100	1.63967200	1.21780100
C	-3.98955600	-1.58073500	-0.67646300
H	-1.95363000	-1.59710200	-1.38186900
C	-4.93035300	-0.91477500	0.10505400
H	-5.30433000	0.76364300	1.39441400
H	-4.26639500	-2.48384200	-1.20818500
H	-5.94111000	-1.29960400	0.18211900
C	2.40065600	0.13551900	-0.30165400
C	3.65640100	0.55167800	-0.73803400
C	2.31227700	-0.94839600	0.57025000
C	4.80816500	-0.09672200	-0.30471300
H	3.73526200	1.38745100	-1.42696300
C	3.46100100	-1.60089200	1.00060100
H	1.33651600	-1.28918500	0.89912400
C	4.71275400	-1.17525700	0.56683500
H	5.77899500	0.23689000	-0.65407600
H	3.37907100	-2.44728300	1.67361300
H	5.60846300	-1.68613000	0.90189700
C	1.16259000	0.89153600	-0.72767400
H	1.36150000	1.36058100	-1.69705900

Transition State VII-TS(OAc)

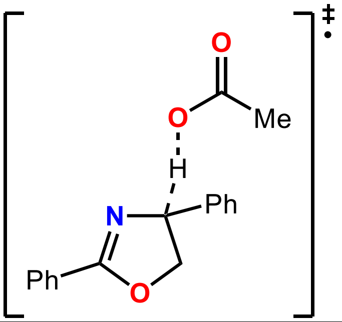

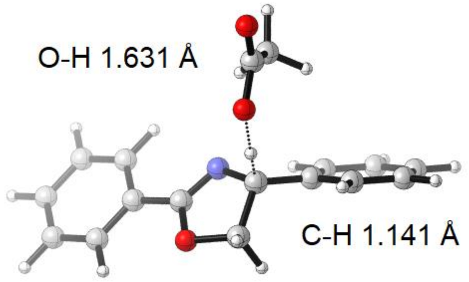

**Total EE (hartree)**	-937.743622
**EE + Zero-Point Energy Correction (hartree)**	-937.451002
**EE + Thermal Enthalpy Correction (hartree)**	-937.431477
**EE + Thermal Free Energy Correction (hartree)**	-937.502842
**Imaginary Frequencies (cm^{-1})**	1, -283.41
**$<S^2>$**	0.7501

Cartesian Coordinates

C	-1.44176100	-0.30622700	0.53007300
N	-0.40797000	-0.15181300	-0.20778700
O	-1.24410900	-0.53619500	1.84250500
C	0.17467600	-0.68957300	2.01218400
H	0.38950700	-1.74832000	2.18021500
H	0.48724000	-0.10261700	2.87408300
C	-2.83554100	-0.25102200	0.07302100
C	-3.88553600	-0.49833900	0.95788000
C	-3.10497600	0.05200100	-1.26341200
C	-5.19744800	-0.44267600	0.50574400
H	-3.67179900	-0.73188800	1.99336000
C	-4.41657900	0.10512900	-1.70897300
H	-2.27942900	0.24373900	-1.93799000
C	-5.46399000	-0.14201400	-0.82530900
H	-6.01245700	-0.63414700	1.19390500
H	-4.62472100	0.34112200	-2.74605200
H	-6.48909400	-0.09877200	-1.17563600
C	1.93074500	-0.94012700	0.13786100
C	3.15019900	-0.83649000	0.80984300
C	1.84734800	-1.72410700	-1.01007500
C	4.26754400	-1.51807800	0.34720900
H	3.22733400	-0.20579600	1.69031100
C	2.96827100	-2.40425800	-1.47419900
H	0.90268400	-1.79135500	-1.53645300
C	4.17861300	-2.30435200	-0.79802800
H	5.21036500	-1.42955800	0.87493100
H	2.89509200	-3.01263500	-2.36876600
H	5.05173800	-2.83311000	-1.16325800
C	0.73740300	-0.19637600	0.66640700
H	1.07923300	0.88220400	0.81450900
O	2.28617800	4.13193600	-0.09460000
C	1.92324400	2.96944700	-0.16874700
O	1.58482700	2.42495100	0.96739300

C	1.85681500	2.15645800	-1.42249900
H	2.60820900	1.36330300	-1.37819500
H	0.87860500	1.67884700	-1.51796900
H	2.05397200	2.79977600	-2.27875400

Transition State VII-TS(Me)

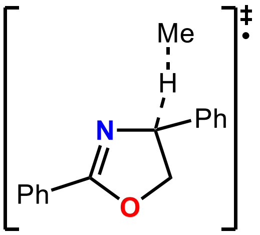

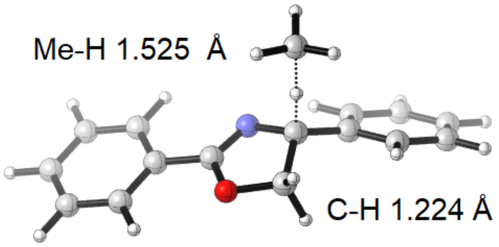

**Total EE (hartree)**	-749.162259
**EE + Zero-Point Energy Correction (hartree)**	-748.888537
**EE + Thermal Enthalpy Correction (hartree)**	-748.871311
**EE + Thermal Free Energy Correction (hartree)**	-748.936160
**Imaginary Frequencies (cm^{-1})**	1, -1231.83
**<S^2>**	0.7627

Cartesian Coordinates

C	-1.09929600	-0.37992000	-0.15469200
N	-0.11752600	0.17767200	0.43779500
O	-0.83774100	-1.47816400	-0.90421800
C	0.59344400	-1.61119900	-0.92159000
H	0.95912400	-1.32706700	-1.91356600
H	0.84357400	-2.65343300	-0.72058700
C	-2.50213800	0.05328200	-0.09730900
C	-3.47950700	-0.59170200	-0.85560800
C	-2.85566200	1.12483400	0.72578800
C	-4.80029900	-0.16506800	-0.79156200
H	-3.20207400	-1.42262100	-1.49213700
C	-4.17497600	1.54683900	0.78512900
H	-2.08712100	1.61419300	1.31178200
C	-5.14959500	0.90296000	0.02676000
H	-5.55729800	-0.66790700	-1.38234700
H	-4.44621300	2.37889900	1.42471200
H	-6.18082900	1.23429600	0.07537600
C	1.04184200	-0.63333400	0.17282400
H	1.21629700	-1.30555900	1.18045900
C	2.32698600	0.09171800	-0.04711800
C	3.45960500	-0.62206100	-0.44921600
C	2.43870500	1.46377700	0.17632300
C	4.67518100	0.02364300	-0.63347100
H	3.39330000	-1.69325000	-0.61611800
C	3.65686100	2.10866000	-0.00641000
H	1.56210200	2.01846500	0.48789600
C	4.77817100	1.39345100	-0.41184400
H	5.54427500	-0.54341700	-0.94826400
H	3.72888700	3.17689100	0.16674900
H	5.72659200	1.89895800	-0.55467400

C	1.38804800	-2.21977800	2.38931100
H	2.27653900	-2.78864900	2.13174900
H	1.50999400	-1.48249600	3.17642000
H	0.45325100	-2.77230300	2.39784300

Transition State VII-TS(I)

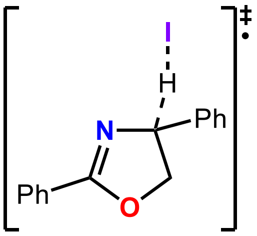

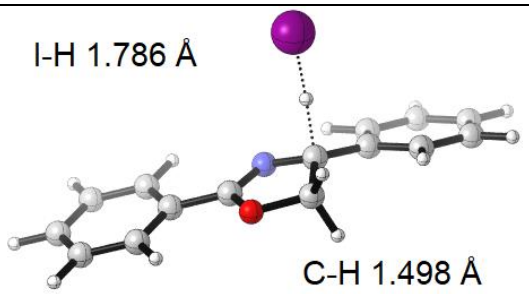

**Total EE (hartree)**	-1007.090383
**EE + Zero-Point Energy Correction (hartree)**	-1006.852084
**EE + Thermal Enthalpy Correction (hartree)**	-1006.835884
**EE + Thermal Free Energy Correction (hartree)**	-1006.899787
**Imaginary Frequencies (cm^{-1})**	1, -707.20
**$<S^2>$**	0.7723

Cartesian Coordinates

C	-1.57504800	-0.58862100	0.51416500
N	-0.58101200	-0.92489400	-0.24252200
O	-1.27653900	-0.22613700	1.77311100
C	0.13508300	-0.42735500	1.92832700
H	0.29700300	-1.28650800	2.58869200
H	0.56864100	0.46841800	2.37473100
C	-2.98031300	-0.56707800	0.12027200
C	-3.97502500	-0.25060000	1.04832800
C	-3.32480800	-0.86581400	-1.20112500
C	-5.30564100	-0.23617900	0.65435300
H	-3.70312800	-0.01688900	2.07005300
C	-4.65581700	-0.84986600	-1.58656600
H	-2.54211900	-1.10365300	-1.91090000
C	-5.64708200	-0.53574500	-0.66033400
H	-6.07745100	0.01013900	1.37401600
H	-4.92261400	-1.08000100	-2.61133600
H	-6.68735900	-0.52264900	-0.96528800
C	0.57160500	-0.71592700	0.50364000
H	0.95675700	0.65947300	0.05280400
C	1.81872600	-1.40270500	0.17549100
C	2.90735700	-1.34347200	1.05518700
C	1.95806200	-2.08163700	-1.04132300
C	4.10322000	-1.96677300	0.73170000
H	2.82342800	-0.80129500	1.99135500
C	3.15654800	-2.70141400	-1.35886000
H	1.11726500	-2.11785400	-1.72293100
C	4.23160200	-2.64778900	-0.47523700
H	4.93911400	-1.91724200	1.41990100
H	3.25501200	-3.22925700	-2.30056400

H	5.16781400	-3.13212500	-0.72806400
I	1.33162700	2.36676700	-0.31260000

Transition State VII-TS(Im)

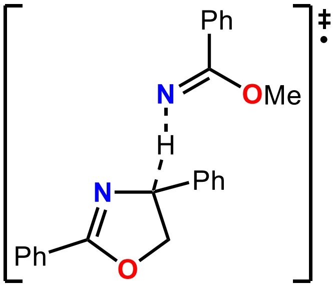

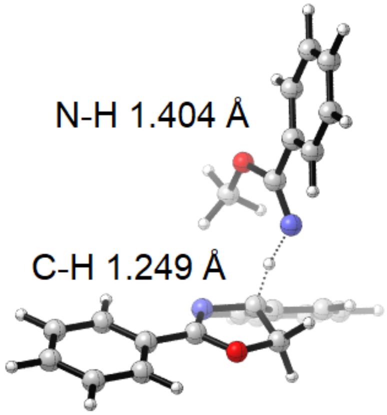

**Total EE (hartree)**	-1148.860556
**EE + Zero-Point Energy Correction (hartree)**	-1148.475489
**EE + Thermal Enthalpy Correction (hartree)**	-1148.451388
**EE + Thermal Free Energy Correction (hartree)**	-1148.533376
**Imaginary Frequencies (cm^{-1})**	1, -1760.98
**<S^2>**	0.7661

Cartesian Coordinates

C	-2.18120300	-0.58036200	-0.51925100
N	-1.69496600	0.33505900	0.23012800
O	-1.89851400	-0.52239100	-1.83655400
C	-1.15658800	0.69330800	-2.03575300
H	-1.78345700	1.39748600	-2.59076300
H	-0.25549000	0.45808400	-2.60384200
C	-3.02354700	-1.69768200	-0.07461800
C	-3.54511000	-2.60672600	-0.99545700
C	-3.30243100	-1.84720100	1.28562700
C	-4.34149700	-3.65684500	-0.55650300
H	-3.32510600	-2.48824300	-2.04904000
C	-4.09846000	-2.89669400	1.71795900
H	-2.88846900	-1.13555100	1.98958100
C	-4.61908300	-3.80316000	0.79784100
H	-4.74520800	-4.36209000	-1.27373000
H	-4.31303900	-3.01101900	2.77423300
H	-5.24033500	-4.62401200	1.13813100
C	-0.86705100	1.15258200	-0.60382000
H	0.30903800	0.76475100	-0.44222500
C	2.28429400	-0.14614200	0.33202200
N	1.56067200	0.16862400	-0.66122900
C	3.51770700	-0.96423000	0.11362500
C	3.62423500	-1.76487900	-1.02406700
C	4.56774600	-0.92443300	1.03049800
C	4.77150000	-2.51416400	-1.24359500
H	2.80068100	-1.79653400	-1.72698600

C	5.71634400	-1.67359100	0.80490000
H	4.48245400	-0.30712300	1.91569100
C	5.82062800	-2.46906100	-0.33051000
H	4.84563200	-3.13850800	-2.12673300
H	6.53095400	-1.63592100	1.51929100
H	6.71632600	-3.05539800	-0.50262500
O	2.12680400	0.17023300	1.62829400
C	1.10069400	1.09104200	1.99290000
H	1.18793600	2.01196700	1.41136700
H	0.11156300	0.65211900	1.84859000
H	1.26232700	1.30314900	3.04786300
C	-0.85240600	2.61358800	-0.32336600
C	-0.08735400	3.46034800	-1.13117800
C	-1.54954100	3.15478700	0.75711800
C	-0.02787800	4.82194700	-0.86747300
H	0.47661500	3.04739600	-1.96173100
C	-1.48630500	4.51824100	1.02086600
H	-2.14096500	2.49717600	1.38284100
C	-0.72674900	5.35580600	0.21148400
H	0.56920100	5.46763500	-1.50173100
H	-2.03557900	4.92837400	1.86126100
H	-0.67902800	6.41888900	0.41885000

Intermediate VII

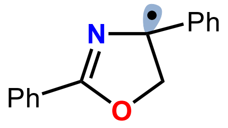

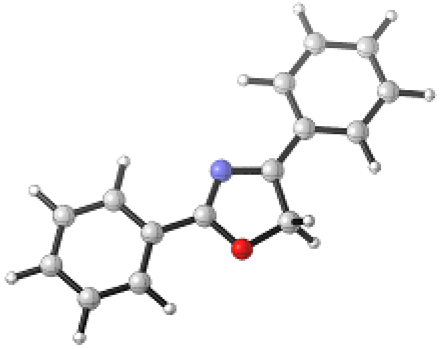

**Total EE (hartree)**	-708.704268
**EE + Zero-Point Energy Correction (hartree)**	-708.472777
**EE + Thermal Enthalpy Correction (hartree)**	-708.458723
**EE + Thermal Free Energy Correction (hartree)**	-708.514596
**Imaginary Frequencies (cm^{-1})**	0
**<S^2>**	0.7519

Cartesian Coordinates

C	-1.05350400	-0.46101800	-0.00002300
N	0.00314800	0.30912300	0.00004200
O	-0.79751500	-1.79204200	-0.00004800
C	0.62891800	-1.93017400	-0.00001500
H	0.93255000	-2.49231400	-0.89067800
H	0.93252400	-2.49223300	0.89070100
C	-2.43759400	-0.02820200	-0.00003500
C	-3.47882100	-0.96366200	0.00020300
C	-2.73854500	1.34037800	-0.00025200

C	-4.79848800	-0.53356300	0.00025900
H	-3.24775300	-2.02166200	0.00040100
C	-4.05862200	1.75996700	-0.00023200
H	-1.92779000	2.05874200	-0.00041400
C	-5.09288500	0.82591600	0.00002600
H	-5.60054900	-1.26283900	0.00047600
H	-4.28509300	2.82007800	-0.00038300
H	-6.12469900	1.15850500	0.00006800
C	2.44113900	-0.04009800	-0.00007900
C	3.50650900	-0.96086800	-0.00021300
C	2.74416800	1.33623900	0.00014300
C	4.82139100	-0.52037900	-0.00009100
H	3.30360000	-2.02689400	-0.00037400
C	4.05934000	1.76487800	0.00021900
H	1.92988400	2.05077500	0.00023700
C	5.10639300	0.84215100	0.00011900
H	5.62931600	-1.24359900	-0.00014800
H	4.27623400	2.82741200	0.00035500
H	6.13494900	1.18414600	0.00022400
C	1.08475500	-0.49617200	-0.00009200

Transition State VII-TS(α-O)

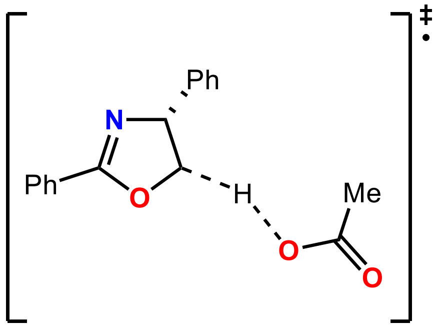

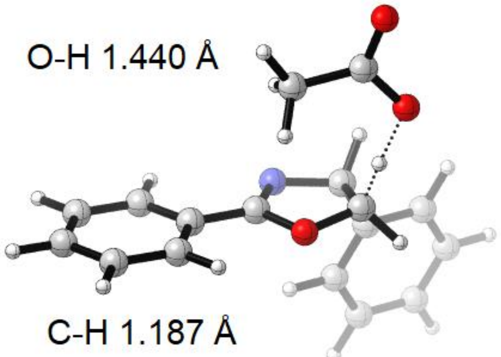

**Total EE (hartree)**	-937.736061
**EE + Zero-Point Energy Correction (hartree)**	-937.444965
**EE + Thermal Enthalpy Correction (hartree)**	-937.425681
**EE + Thermal Free Energy Correction (hartree)**	-937.496431
**Imaginary Frequencies (cm^{-1})**	1, -777.88
**<S^2>**	0.7582

Cartesian Coordinates

C	-0.69067500	-0.44969100	0.01106300
N	0.15705300	-0.37467600	-0.93043700
O	-0.39770800	0.23513100	1.16310500
C	0.73701400	0.99953400	0.87875300
H	1.40920800	1.03079400	1.73608900
H	0.40456400	2.12616200	0.71001800
C	-1.96022600	-1.18431400	0.00291300
C	-2.82841000	-1.12155000	1.09308700
C	-2.29885300	-1.94378800	-1.11902200
C	-4.03095000	-1.81604500	1.05828200

H	-2.56247800	-0.53325000	1.96246600
C	-3.50055400	-2.63400300	-1.14712600
H	-1.61417700	-1.98263200	-1.95759800
C	-4.36777200	-2.57077400	-0.05933100
H	-4.70512100	-1.76687900	1.90530600
H	-3.76335100	-3.22273700	-2.01818800
H	-5.30716100	-3.11137000	-0.08419400
C	2.55104300	-0.36238200	-0.25836100
C	3.78149100	0.27054500	-0.42321700
C	2.51827400	-1.69939100	0.13130600
C	4.96530100	-0.42184500	-0.19779600
H	3.81480700	1.31045000	-0.73334100
C	3.70215700	-2.39284800	0.35359200
H	1.56660800	-2.20617700	0.24674800
C	4.92775100	-1.75602900	0.19192700
H	5.91684100	0.07973800	-0.33304200
H	3.66653100	-3.43505200	0.65026800
H	5.85018000	-2.29900900	0.36373500
C	1.27332200	0.43602500	-0.44807000
H	1.46871500	1.24181100	-1.15969700
O	0.15761600	3.51695300	0.43237800
C	-1.00106700	3.78411500	-0.13591800
O	-1.21943500	4.94404900	-0.41754300
C	-1.94969300	2.64930700	-0.39784300
H	-1.52912200	1.98890300	-1.16107300
H	-2.11030900	2.06270100	0.50893700
H	-2.89781100	3.04901300	-0.75232100

Intermediate VII(α-O)

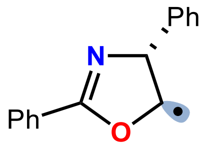

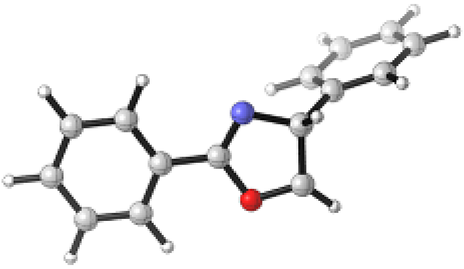

**Total EE (hartree)**	-708.669136
**EE + Zero-Point Energy Correction (hartree)**	-708.437530
**EE + Thermal Enthalpy Correction (hartree)**	-708.423471
**EE + Thermal Free Energy Correction (hartree)**	-708.480227
**Imaginary Frequencies (cm^{-1})**	0
**<S^2>**	0.7550

Cartesian Coordinates

C	-0.90703100	-0.62909300	0.22722100
N	-0.01207700	-0.24173700	1.04092700
O	-0.56044200	-1.63688700	-0.63488500
C	0.71402300	-2.00509100	-0.27640500
H	1.29192500	-2.49430100	-1.04719600
C	-2.27414300	-0.10838600	0.10166600

C	-3.17079400	-0.66487000	-0.81053300
C	-2.67207900	0.96088200	0.90683100
C	-4.45833300	-0.15347100	-0.91467800
H	-2.85984100	-1.49439500	-1.43323000
C	-3.95839300	1.46679100	0.79804600
H	-1.96478700	1.38302300	1.61047100
C	-4.85329100	0.91089300	-0.11256000
H	-5.15358900	-0.58787600	-1.62362100
H	-4.26526000	2.29672300	1.42399700
H	-5.85859100	1.30832900	-0.19571600
C	2.37200400	-0.19314700	0.31387600
C	3.66499800	-0.55016500	0.68707100
C	2.18455600	0.90622900	-0.52185200
C	4.75901400	0.17526000	0.22681200
H	3.81917600	-1.39983000	1.34533000
C	3.27561000	1.63536400	-0.97762200
H	1.17974300	1.19982100	-0.80605800
C	4.56632200	1.27039400	-0.60692000
H	5.76093200	-0.11162700	0.52624000
H	3.11811100	2.49309700	-1.62210700
H	5.41695400	1.84079700	-0.96262000
C	1.19040000	-1.03713200	0.77268300
H	1.48611400	-1.55324200	1.69528800

Intermediate VIII

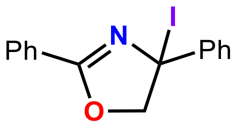

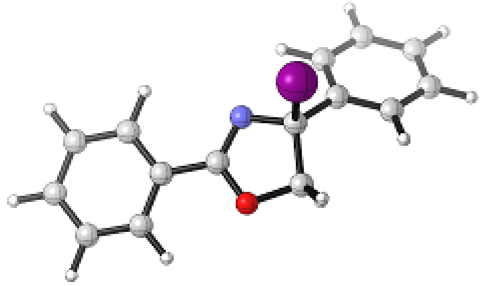

**Total EE (hartree)**	-1006.519329
**EE + Zero-Point Energy Correction (hartree)**	-1006.284573
**EE + Thermal Enthalpy Correction (hartree)**	-1006.268886
**EE + Thermal Free Energy Correction (hartree)**	-1006.329815
**Imaginary Frequencies (cm^{-1})**	0

Cartesian Coordinates

C	1.47230500	0.24629800	0.46898600
N	0.49175200	0.45840400	-0.32578400
O	1.18157800	-0.19421400	1.70931400
C	-0.23987300	-0.07564400	1.83227000
H	-0.45866800	0.84514700	2.38275900
H	-0.62199400	-0.93792000	2.37104500
C	2.89053400	0.42521500	0.14370500
C	3.86916800	0.28207200	1.12784500
C	3.25649500	0.74108000	-1.16645600
C	5.20743700	0.45692900	0.80097700
H	3.57979800	0.03523500	2.14169600

C	4.59415500	0.91556700	-1.48591200
H	2.48532000	0.84223100	-1.92023300
C	5.57047700	0.77408900	-0.50326300
H	5.96740200	0.34526300	1.56545400
H	4.87796600	1.15944000	-2.50299900
H	6.61602300	0.90976900	-0.75605900
C	-1.88482300	0.96689900	0.15273000
C	-2.98288400	0.90056800	1.01208400
C	-1.89156000	1.88428800	-0.89480300
C	-4.07048800	1.74191000	0.82725600
H	-2.99875800	0.17842200	1.82241200
C	-2.98483400	2.72284200	-1.08095800
H	-1.03653600	1.93680200	-1.55659100
C	-4.07526100	2.65555900	-0.22260700
H	-4.91760600	1.68170300	1.50096200
H	-2.98127100	3.43316100	-1.90003600
H	-4.92626700	3.31094100	-0.36916200
C	-0.69967500	0.06839400	0.37104200
I	-1.25787400	-1.95531500	-0.42397400

Intermediate IX

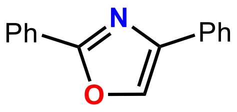

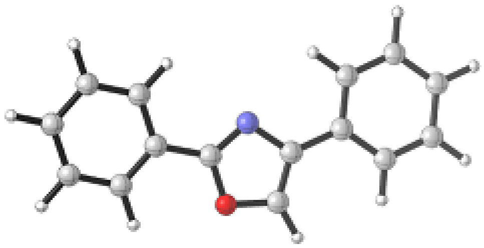

**Total EE (hartree)**	-708.125650
**EE + Zero-Point Energy Correction (hartree)**	-707.903549
**EE + Thermal Enthalpy Correction (hartree)**	-707.889932
**EE + Thermal Free Energy Correction (hartree)**	-707.945636
**Imaginary Frequencies (cm^{-1})**	0

Cartesian Coordinates

C	1.04054600	-0.44820200	0.00006900
N	-0.01133100	0.30424100	-0.00000800
O	0.75670900	-1.76701200	0.00020500
C	-0.60136300	-1.83607700	0.00023000
H	-1.03493300	-2.82066400	0.00048500
C	2.44455700	-0.03800000	-0.00001200
C	3.47014000	-0.98490900	0.00014400
C	2.75725200	1.32320800	-0.00023700
C	4.79570700	-0.57102100	0.00008000
H	3.22959100	-2.04109600	0.00032300
C	4.08333700	1.72952200	-0.00029900
H	1.95409300	2.05023100	-0.00035700
C	5.10540400	0.78455200	-0.00014100
H	5.58866100	-1.31001200	0.00020700
H	4.32104100	2.78707600	-0.00047000
H	6.14105800	1.10497900	-0.00019200

C	-2.47898100	-0.09693500	0.00002400
C	-3.54904000	-0.99630900	-0.00044800
C	-2.74772600	1.27270900	0.00043300
C	-4.85761100	-0.53492200	-0.00048000
H	-3.36290800	-2.06492500	-0.00081400
C	-4.05891800	1.73243700	0.00038500
H	-1.91946000	1.97075500	0.00079100
C	-5.11807500	0.83225000	-0.00006700
H	-5.67730400	-1.24461300	-0.00085000
H	-4.25291900	2.79923300	0.00070400
H	-6.14080900	1.19185200	-0.00010900
C	-1.08864000	-0.57103800	0.00010200

Acetoxy Radical

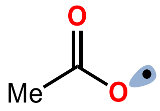

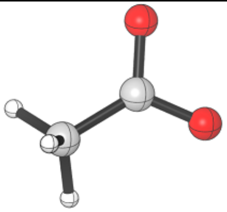

**Total EE (hartree)**	-228.404778
**EE + Zero-Point Energy Correction (hartree)**	-228.356997
**EE + Thermal Enthalpy Correction (hartree)**	-228.351583
**EE + Thermal Free Energy Correction (hartree)**	-228.384751
**Imaginary Frequencies (cm^{-1})**	0
**<S^2>**	0.7576

Cartesian Coordinates

C	0.09718700	-0.00013900	-0.00821600
O	0.81155500	-1.02892900	0.00146800
C	-1.38736100	0.00052800	-0.00315800
H	-1.76229900	0.89450700	-0.50186700
H	-1.76358700	-0.89993400	-0.48967500
H	-1.72752500	0.00733000	1.03565700
O	0.81275200	1.02839900	0.00154900

Acetic Acid

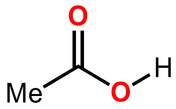

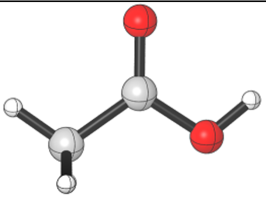

**Total EE (hartree)**	-229.090480
**EE + Zero-Point Energy Correction (hartree)**	-229.028308
**EE + Thermal Enthalpy Correction (hartree)**	-229.022806
**EE + Thermal Free Energy Correction (hartree)**	-229.055544
**Imaginary Frequencies (cm^{-1})**	0

Cartesian Coordinates

C	0.08977900	0.12257200	0.00025600
O	0.78518500	-1.02909100	0.00006100

H	1.72408000	-0.80523600	-0.00076700
C	-1.38962400	-0.11933300	0.00002600
H	-1.91690600	0.83147500	0.00325800
H	-1.66648100	-0.69847000	-0.88319300
H	-1.66653600	-0.70514400	0.87872600
O	0.63042900	1.19883300	-0.00002600

Methyl Radical

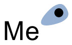	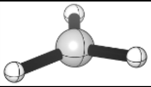
**Total EE (hartree)**	-39.835237
**EE + Zero-Point Energy Correction (hartree)**	-39.805568
**EE + Thermal Enthalpy Correction (hartree)**	-39.801571
**EE + Thermal Free Energy Correction (hartree)**	-39.823665
**Imaginary Frequencies (cm^{-1})**	0
**$\langle S^2 \rangle$**	0.7538

Cartesian Coordinates

C	0.00000000	0.00000000	0.00000000
H	0.00000000	1.08165900	0.00000000
H	0.93674400	-0.54083000	0.00000000
H	-0.93674400	-0.54083000	0.00000000

Methane

CH_4	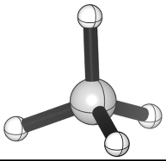
**Total EE (hartree)**	-40.515661
**EE + Zero-Point Energy Correction (hartree)**	-40.470949
**EE + Thermal Enthalpy Correction (hartree)**	-40.467135
**EE + Thermal Free Energy Correction (hartree)**	-40.488266
**Imaginary Frequencies (cm^{-1})**	0

Cartesian Coordinates

C	0.00000000	0.00000000	0.00000000
H	0.62966300	0.62966300	0.62966300
H	-0.62966300	-0.62966300	0.62966300
H	-0.62966300	0.62966300	-0.62966300
H	0.62966300	-0.62966300	-0.62966300

Iodine Atom Radical

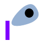	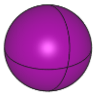
**Total EE (hartree)**	-297.760405
**EE + Zero-Point Energy Correction (hartree)**	-297.760405
**EE + Thermal Enthalpy Correction (hartree)**	-297.758045
**EE + Thermal Free Energy Correction (hartree)**	-297.777908
**Imaginary Frequencies (cm^{-1})**	0
**$\langle S^2 \rangle$**	0.7525

**Cartesian Coordinates**			
I	0.00000000	0.00000000	0.00000000

Hydrogen Iodide

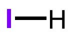	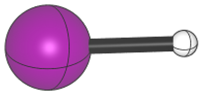
**Total EE (hartree)**	-298.387899
**EE + Zero-Point Energy Correction (hartree)**	-298.382594
**EE + Thermal Enthalpy Correction (hartree)**	-298.379289
**EE + Thermal Free Energy Correction (hartree)**	-298.402721
**Imaginary Frequencies (cm^{-1})**	0

**Cartesian Coordinates**			
I	0.00000000	0.00000000	0.02980100
H	0.00000000	0.00000000	-1.57946500

Hydrogen Atom Radical

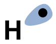	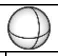
**Total EE (hartree)**	-0.502818
**EE + Zero-Point Energy Correction (hartree)**	-0.502818
**EE + Thermal Enthalpy Correction (hartree)**	-0.500458
**EE + Thermal Free Energy Correction (hartree)**	-0.513472
**Imaginary Frequencies (cm^{-1})**	0
**<S^2>**	0.7500

**Cartesian Coordinates**			
H	0.00000000	0.00000000	0.00000000

Methyl Benzimidate N-Centered Radical

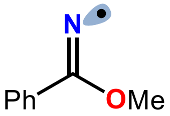	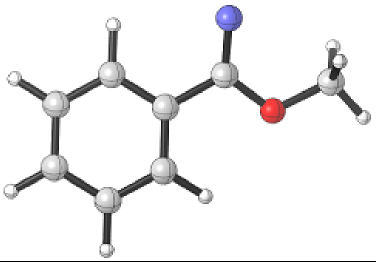
**Total EE (hartree)**	-439.535245
**EE + Zero-Point Energy Correction (hartree)**	-439.391794
**EE + Thermal Enthalpy Correction (hartree)**	-439.381994
**EE + Thermal Free Energy Correction (hartree)**	-439.427262
**Imaginary Frequencies (cm^{-1})**	0
**<S^2>**	0.7501

**Cartesian Coordinates**			
C	1.25109800	0.40703100	0.05636000
C	-0.21460200	0.15471600	0.01788000
C	-1.09958200	1.22718600	-0.10330800
C	-0.70646500	-1.14609700	0.11430200
C	-2.46756600	0.99747800	-0.12182600
H	-0.71239600	2.23587800	-0.18862700

C	-2.07780700	-1.36957000	0.09489700
H	-0.01674500	-1.97562000	0.20483000
C	-2.95896100	-0.30081000	-0.02226400
H	-3.15178600	1.83241600	-0.21880600
H	-2.45762900	-2.38189900	0.17132000
H	-4.02829200	-0.47865400	-0.03876500
N	1.74501600	1.54860500	0.29063500
O	2.00031500	-0.68655800	-0.16574500
C	3.41529900	-0.49955000	-0.09935500
H	3.74173400	0.21619300	-0.85751600
H	3.70703100	-0.14174700	0.89085300
H	3.85196700	-1.47664000	-0.29188800

N-Iodo Methyl Benzimidate

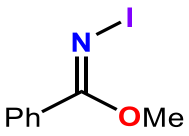

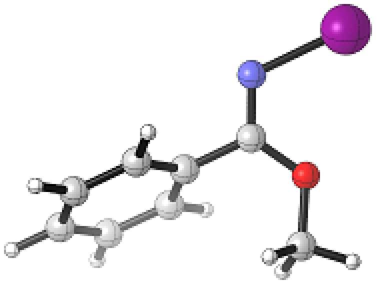

**Total EE (hartree)**	-450.955047
**EE + Zero-Point Energy Correction (hartree)**	-450.809797
**EE + Thermal Enthalpy Correction (hartree)**	-450.798172
**EE + Thermal Free Energy Correction (hartree)**	-450.849184
**Imaginary Frequencies (cm^{-1})**	0

Cartesian Coordinates

C	0.29691300	0.24770600	0.01038400
C	1.75253000	-0.07789000	-0.03796800
C	2.28642500	-0.99750200	0.86093400
C	2.57371400	0.52161200	-0.99305500
C	3.64128500	-1.30324900	0.81601700
H	1.63741600	-1.47048300	1.58822800
C	3.92436100	0.20510200	-1.04090000
H	2.15377700	1.22067600	-1.70808900
C	4.46070000	-0.70241500	-0.13249400
H	4.05518100	-2.01585700	1.51995600
H	4.55826100	0.66373600	-1.79091400
H	5.51646800	-0.94546100	-0.16935500
N	-0.50325200	-0.72214500	-0.18947800
I	-2.55107200	-0.27764900	-0.08318900
O	-0.09219300	1.50932000	0.22257900
C	0.81234500	2.45912200	0.79707800
H	0.18405900	3.27912300	1.13766900
H	1.34897200	2.02679800	1.64296800
H	1.52337000	2.82239700	0.05428600

Methyl Benzimidate

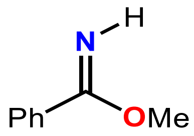	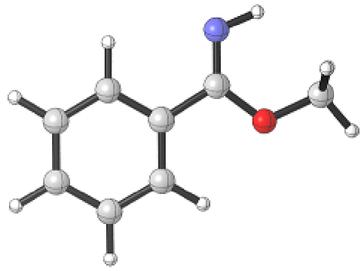

Total EE (hartree)	-440.201248
EE + Zero-Point Energy Correction (hartree)	-440.044572
EE + Thermal Enthalpy Correction (hartree)	-440.034863
EE + Thermal Free Energy Correction (hartree)	-440.079113
Imaginary Frequencies (cm^{-1})	0

Cartesian Coordinates

C	1.22808400	0.41352100	0.00002600
C	-0.23699000	0.14494600	0.00000700
C	-1.12610100	1.22104400	-0.00004900
C	-0.73662100	-1.15743100	0.00004100
C	-2.49496500	0.99663400	-0.00005900
H	-0.72701400	2.22777900	-0.00007600
C	-2.10896700	-1.37893900	0.00004500
H	-0.05096200	-1.99472000	0.00007600
C	-2.99020800	-0.30438600	-0.00000700
H	-3.17829500	1.83829000	-0.00010600
H	-2.48906800	-2.39426100	0.00008600
H	-4.06043200	-0.47888400	-0.00001000
N	1.68168600	1.59924100	0.00010800
O	1.94166200	-0.73342500	-0.00003500
C	3.35670000	-0.64229300	-0.00005700
H	3.71851500	-0.12840300	-0.89593500
H	3.71853800	-0.12857000	0.89589900
H	3.72169600	-1.66684500	-0.00019800
H	2.69633400	1.63976000	0.00010400

PhI(OAc)$_2$

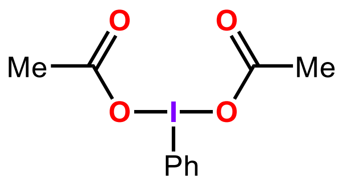	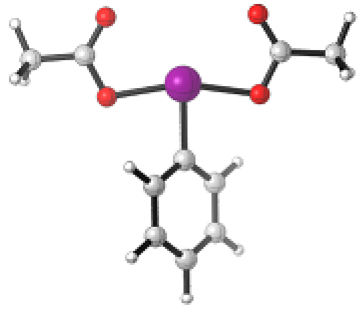

Total EE (hartree)	-986.296037
EE + Zero-Point Energy Correction (hartree)	-986.102248
EE + Thermal Enthalpy Correction (hartree)	-986.084323
EE + Thermal Free Energy Correction (hartree)	-986.151250

Imaginary Frequencies (cm^{-1})	0

Cartesian Coordinates

C	0.00559200	-4.23867900	-0.00002400
C	0.78884700	-3.54423300	-0.91421200
C	0.79086000	-2.15443800	-0.92218300
C	0.00187200	-1.48179600	-0.00003800
C	-0.78532500	-2.15655400	0.92208900
C	-0.77955500	-3.54634200	0.91413900
H	0.00707000	-5.32259000	-0.00000800
H	1.40120700	-4.08206200	-1.62853100
H	1.40918600	-1.61058600	-1.62444100
H	-1.40512500	-1.61435500	1.62433000
H	-1.39045700	-4.08581700	1.62846500
I	-0.00082800	0.61613100	0.00000600
O	2.12502500	0.29806300	0.09502500
C	2.73005300	1.46339500	0.12833700
O	2.11326900	2.51381700	0.10802800
O	-2.12594800	0.29267600	-0.09481700
C	-2.73380600	1.45651600	-0.12833100
O	-2.11956600	2.50844500	-0.10823300
C	4.23227300	1.37094700	0.19236300
H	4.60378800	0.84219500	-0.68773600
H	4.66123200	2.36990100	0.23598000
H	4.52658800	0.79561500	1.07210200
C	-4.23579600	1.36040800	-0.19226000
H	-4.66716900	2.35828500	-0.23668200
H	-4.52877700	0.78359400	-1.07147000
H	-4.60597900	0.83152900	0.68832900

CsI

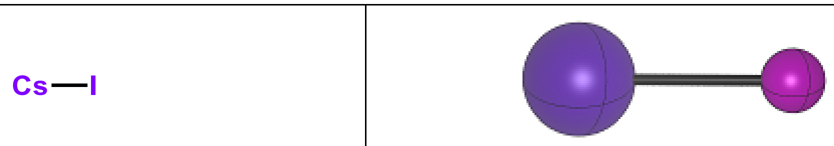

Total EE (hartree)	-318.052659
EE + Zero-Point Energy Correction (hartree)	-318.052393
EE + Thermal Enthalpy Correction (hartree)	-318.048385
EE + Thermal Free Energy Correction (hartree)	-318.079668
Imaginary Frequencies (cm^{-1})	0

Cartesian Coordinates

Cs	0.00000000	0.00000000	1.66485600
I	0.00000000	0.00000000	-1.72768100

PhI

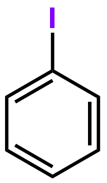

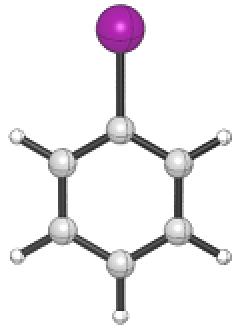

Total EE (hartree)	-529.403162
EE + Zero-Point Energy Correction (hartree)	-529.312450
EE + Thermal Enthalpy Correction (hartree)	-529.305693
EE + Thermal Free Energy Correction (hartree)	-529.344118
Imaginary Frequencies (cm^{-1})	0

Cartesian Coordinates

C	0.00000000	3.32604900	0.00000000
C	0.00000000	2.62861400	1.20206000
C	0.00000000	1.23812700	1.20908300
C	0.00000000	0.55019800	0.00000000
C	0.00000000	1.23812700	-1.20908300
C	0.00000000	2.62861400	-1.20206000
H	0.00000000	4.40989000	0.00000000
H	0.00000000	3.16544600	2.14398300
H	0.00000000	0.69826700	2.14811600
H	0.00000000	0.69826700	-2.14811600
H	0.00000000	3.16544600	-2.14398300
I	0.00000000	-1.54331500	0.00000000

CsOAc

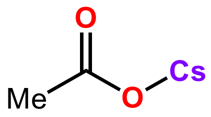

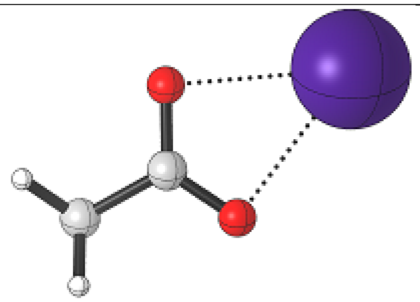

Total EE (hartree)	-248.730424
EE + Zero-Point Energy Correction (hartree)	-248.680425
EE + Thermal Enthalpy Correction (hartree)	-248.673038
EE + Thermal Free Energy Correction (hartree)	-248.714013
Imaginary Frequencies (cm^{-1})	0

Cartesian Coordinates

C	1.95700100	0.00058100	-0.01383400
O	1.36662300	1.11504200	-0.00843000
O	1.36649900	-1.11390400	-0.00845900

C	3.47759300	-0.00038000	0.00547800
H	3.80836800	-0.02154600	1.04839300
H	3.86805300	-0.89009600	-0.48976900
H	3.87007400	0.90613100	-0.45629700
Cs	-1.20034600	-0.00008700	0.00150800

AcOI

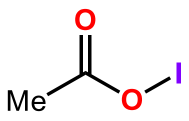

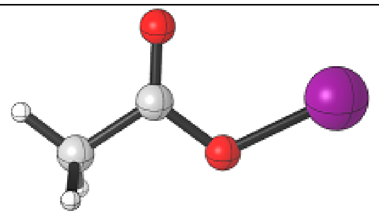

**Total EE (hartree)**	-526.231050
**EE + Zero-Point Energy Correction (hartree)**	-526.180063
**EE + Thermal Enthalpy Correction (hartree)**	-526.173117
**EE + Thermal Free Energy Correction (hartree)**	-526.212701
**Imaginary Frequencies (cm^{-1})**	0

Cartesian Coordinates

C	1.77126300	0.18576400	-0.00026400
C	3.09923000	-0.52672200	-0.00007900
H	3.17642400	-1.16388000	0.88239600
H	3.17577700	-1.16591700	-0.88111900
H	3.89718200	0.21247700	-0.00115300
O	1.60807600	1.37190000	-0.00016400
O	0.76984100	-0.71892700	-0.00016600
I	-1.10369200	-0.02001400	0.00008600
